# Measuring Perceived Social Support After Disaster: Development and Validation of the Perceived Social Support in Disasters Scale (PSSDS-28)

**DOI:** 10.3390/healthcare14162573

**Published:** 2026-08-17

**Authors:** Abdurrahman Oruç, Ekrem Cengiz

**Affiliations:** 1 Department of Property Protection and Security, Civil Defense and Firefighting, Uzumlu Vocational School, Erzincan Binali Yıldırım University, Erzincan 24150, Türkiye; abdurrahman.oruc@erzincan.edu.tr; 2 Department of Business Administration, Faculty of Economics and Administrative Sciences, Gümüşhane University, Gümüşhane 29100, Türkiye

**Keywords:** disaster, perceived social support, scale development, structural validity, measurement invariance

## Abstract

**Background/Objectives:** Measuring perceived social support after disaster remains an unresolved psychometric problem. Most instruments do not jointly capture the source of support (from whom it comes) and its function (how it operates). None has been validated in a disaster-survivor sample with retrospectively framed items. We developed and validated the Perceived Social Support in Disasters Scale (PSSDS-28), a 28-item measure integrating both dimensions. **Methods:** In Phase 1, a PRISMA 2020-guided systematic review; a two-round Delphi with eight experts, cognitive interviews (*n* = 12); and a pilot study (*N* = 150) produced a 31-item form. In Phase 2, face-to-face data were collected from 607 adults affected by the 6 February 2023 Kahramanmaraş earthquakes (Türkiye). Multiple analytic frameworks converged on a five-factor, 28-item structure comprising family–relative, social environment, institutional, emotional, and instrumental–informational support. **Results:** The correlated five-factor model fit the independent validation sample well (CFI = 0.954, RMSEA = 0.076, and SRMR = 0.072) and outperformed a one-factor alternative (ΔCFI = 0.222). Blocks showed strong internal consistency (ordinal α = 0.852–0.949), composite reliability (0.865–0.950), and adequate test–retest stability (ICC = 0.542–0.909). Scalar measurement invariance held across six groupings, with no meaningful differential item functioning. Convergent and discriminant evidence supported construct validity, and common-method variance was negligible. Institutional (M = 3.29) and instrumental–informational support (M = 2.99) scored lowest—a profile a global score would conceal. **Conclusions:** The PSSDS-28 provides promising initial psychometric evidence for a multidimensional post-disaster assessment instrument situated at the perceived–received interface, a target that general-purpose scales are not designed to capture. Criterion-related validity, cross-cultural validity, and cross-disaster replicability remain to be demonstrated.

## 1. Introduction

On 6 February 2023, two earthquakes of moment magnitude 7.8 and 7.5 struck south-eastern Türkiye nine hours apart, damaging an area comparable in size to Germany and affecting approximately 14 million people across eleven provinces. Roughly 50,800 people died in Türkiye, more than 500,000 buildings collapsed or were heavily damaged, and about three million people were still living in tents three months later; the sequence ranks as the fifth deadliest earthquake of the twenty-first century and the third most severe in earthquake-induced displacement [1]. Five months after the event, probable posttraumatic stress disorder was identified in 55.2% of 1100 survivors sampled across the affected provinces [2], alongside disrupted livelihoods, dissolved neighborhoods, and prolonged reliance on formal assistance. Recovery at this scale is a measurement problem as well as a service problem: describing who recovers, and why, requires instruments that can characterize the support environment in which recovery takes place. The present article addresses that measurement problem.

Social support is among the most consistently documented protective resources for psychological adjustment following adversity, and few contexts make this function as salient as large-scale disaster. Catastrophic events such as major earthquakes simultaneously destroy housing, livelihoods, and health infrastructure while disrupting the interpersonal and institutional networks on which survivors ordinarily rely [3]. The resulting vulnerability is not merely material; it is relational. The perception that support is available—that one has access to people and institutions that will help—independently predicts psychological functioning above and beyond objective resource access [4,5]. In disaster contexts, however, this prospective availability appraisal is inseparable from the retrospective evaluation of support already received: survivors simultaneously hold beliefs about ongoing availability and memories of enacted support, and these appraisals may diverge in theoretically meaningful ways [6]. Accurate measurement of this perceived–received interface is therefore a foundational requirement for both disaster research and post-disaster service planning. We therefore state at the outset what the instrument reported here does and does not measure. The PSSDS-28 measures a survivor’s retrospective appraisal of the support they experienced as having been available and provided since a specified disaster, with the response window anchored to a dated event and weighted toward the recent past. It does not measure prospective availability—the belief that support would be forthcoming should a future need arise—which is the target of instruments such as the MSPSS [7]. We use the term perceived–received interface for this target: the items ask about support that was experienced (the received pole), but they elicit a global subjective appraisal rather than an enumeration of discrete helping episodes (the perceived pole). Where the two constructs are known to diverge [6], readers should assume that the PSSDS-28 indexes the former.

Two complementary mechanistic frameworks explain why perceived support matters under extreme stress. The main-effect model holds that believing support is available promotes well-being through belonging, enhanced self-efficacy, and positive affect regardless of current stress level [4]. The stress-buffering model holds that perceived support operates most potently under high stress, attenuating harmful physiological and appraisal responses when demands threaten to exceed resources [8]. Disaster represents precisely the high-stress context in which buffering is hypothesized to be most consequential; however, the strength and direction of buffering depend on contextual parameters—including support type, source, and timing—in ways that aggregate global measures are poorly positioned to detect [9,10].

Theoretical refinements sharpen these measurement requirements. Optimal-matching theory proposes that support is most effective when its type aligns with stressor demands: uncontrollable, loss-related stressors are best served by emotional support, whereas controllable, resource-related stressors are best served by tangible or informational assistance [11]. Disaster is the context in which both demand types coexist—survivors simultaneously grieve irreversible losses and manage urgent resource deficits—so an instrument insensitive to functional type cannot assess whether available support matches situational requirements. Relational regulation theory introduces a further refinement: perceived support arises substantially from the quality of ongoing everyday interaction—the sense of being accompanied and understood—rather than from discrete help-giving transactions [12], shifting the measurement target from transaction to appraisal.

Two considerations are specific to disaster. First, the mobilization–deterioration model [13,14] documents that disaster generates an initial mobilization surge of generous help, typically followed by a deterioration phase in which networks become strained as helpers experience fatigue, relocation, and resource depletion. Whether perceived support is sustained through recovery rather than only mobilized at peak impact is a consequential empirical question. Second, disaster activates unusually broad support providers: beyond interpersonal sources, communal mechanisms (neighborhood solidarity and mutual aid) and formal institutional channels (government agencies and NGOs) become salient. Whether survivors perceive these institutional actors as supportive has both psychological and policy implications [15,16] and is largely unmeasured by existing instruments.

Across these frameworks, a coherent structural account emerges: perceived social support after disaster is organized along a source dimension—family and relatives, wider social environment, and formal institutions—and a function dimension—emotional regulation, instrumental provision, and informational guidance. This separation follows House’s distinction between the provider of support and the function it serves [17], the functional taxonomy of optimal-matching theory [11], and the appraisal focus of relational regulation theory [12]. A measurement instrument adequate to the disaster context should represent both dimensions. A fully crossed matrix would multiply item count prohibitively and impose unrealistic cognitive demands on distressed survivors [18]. The alternative is a hybrid architecture that preserves both organizing principles within a single instrument and remains sensitive to the relative standing of different support channels. The PSSDS-28 adopts this hybrid approach. Three blocks are organized by source (family–relative, social environment, and institutional) and two by function (emotional and instrumental–informational). Source and function are not orthogonal: institutional support is commonly instrumental, and family support is commonly emotional. Both dimensions nonetheless carry distinct informational value for intervention targeting.

Despite the richness of this theoretical landscape, existing instruments have not been designed to capture the source-by-function architecture that disaster theory demands. Table 1 summarizes the structural coverage of six widely used measures against six disaster-theoretic measurement criteria. The pattern is consistent. The MSPSS [7] captures interpersonal sources but differentiates neither function nor institutional and community channels. The ISEL [19] differentiates emotional from tangible functions but specifies no source structure. The SSQ [20] enumerates network members without differentiating either function or institution. The SPS [21] maps six relational provisions without identifying who supplies them. The 2-Way SSS [22] adds a giving–receiving distinction but contains no institutional or disaster-contextualized content. The F-SozU K-3 [23] provides efficient global screening that by design collapses all sources and functions. No existing instrument fully satisfies more than one of the six criteria, and none satisfies more than two even when partial coverage is counted. A detailed narrative comparison is provided in Supplementary Materials S2.

The absence of institutional coverage in existing instruments is not merely a gap in breadth; it is a theoretically consequential blind spot. Lower institutional trust has been associated with more mental health problems and poorer social support in long-term disaster follow-ups [15], and higher institutional trust predicted fewer posttraumatic stress symptoms after adjustment for resilience and interpersonal support among 2311 survivors [16]. Our systematic review confirmed the resulting measurement asymmetry: family, peer, and significant-person categories were operationalized by 20–22 sources each in the Scale evidence set, whereas community–neighborhood and institutional categories were operationalized by only 4–10.

Table 1 is restricted to instruments that measure perceived social support, since those are the instruments the PSSDS-28 would supplement. The newer literature has produced disaster-focused assessment instruments in adjacent domains—community resilience [24], family resilience in disaster scenarios [25], household earthquake preparedness [26], personal social capital after flooding [27], and disaster health literacy [28]—and it is worth stating why these do not close the present gap. None measures perceived support by source and by function: the community and family instruments locate the construct at the collective rather than the individual-appraisal level; the preparedness instrument treats social support as one antecedent of protective behavior rather than as the target construct; the social–capital instrument indexes network resources rather than appraised support; and the health-literacy instrument addresses informational competence rather than informational support received. Our systematic review reached the same conclusion from the opposite direction: among the 25 disaster-context measurement studies retained in the Scale set (2015–2025), we identified no newly developed instrument for perceived social support in disaster survivors, the field being dominated by adaptations of general-purpose measures, most often the MSPSS [29]. A structured comparison against the criteria used in Table 1 is provided in Supplementary Materials S2.4.

This measurement gap motivates the present study. We do not argue that existing instruments should be replaced. We argue instead that the disaster context warrants a targeted instrument representing both source and function, and validated in a disaster survivor sample. The hybrid architecture proposed here uses three source-organized blocks and two function-organized blocks, which together capture the source-by-function landscape without requiring respondents to evaluate each source–function cell independently. We developed and validated the PSSDS-28 in survivors of the 2023 Kahramanmaraş earthquakes, the largest seismic disaster in modern Turkish history. Our aims were to document the development process, test competing structural models, establish reliability and validity evidence, examine measurement invariance, and identify limitations with precision.

## 2. Materials and Methods

### 2.1. Overview and Design

Scale development followed a sequential two-phase design grounded in contemporary standards [18,30,31]. Phase 1 covered theoretical specification, systematic review, item generation, expert review, cognitive interviews, and piloting. Phase 2 covered main-study data collection and psychometric evaluation. Item-reduction decisions used only pilot- and calibration-stage evidence, preserving the validation subsample as an independent test. Reporting follows JARS [32], TOP guidelines [33], the Standards for Educational and Psychological Testing [34], and COSMIN criteria [35]; and the STROBE statement for cross-sectional studies; item-by-item mapping against all five frameworks is provided in Supplementary Materials S21 (Tables S21.1–S21.5). Complete documentation is provided in the Supplementary Materials S1–S21.

### 2.2. Phase 1: Item Development 

Systematic review. A PRISMA 2020-compliant [36] two-stream search was conducted in Web of Science and Scopus; the PRISMA 2020 flow diagram is presented in Supplementary Materials Figure S2.1. The full search strings and the search date (20 December 2025) are given in Supplementary Materials S2.1, and the complete screening criteria, exclude-reason taxonomy and evidence coding scheme are deposited in the OSF repository (OSF Attachments B). The Theory set targeted mechanistic frameworks (buffering, optimal matching, relational regulation, convoy model, social identity, and timing); the Scale set targeted psychometric studies of social support measurement in disaster or mass-trauma contexts. Searches were limited to English-language articles and reviews, with the Scale set restricted to 2015–2025 and the Theory set spanning 1980–2025. Searches returned 489 records (Web of Science 359; Scopus 130). Two-stage deduplication removed 78 records, leaving 411 for title-and-abstract screening; 194 were excluded under a prespecified five-category taxonomy, and 217 remained eligible. Because full-text extraction of all eligible records was not feasible, a prespecified relevance rubric was applied to prioritize a core set of 55 studies (Theory: 30; Scale: 25), of which 46 (83.6%) were extracted at the full-text level and 9 at the abstract level. Stage-by-stage counts, exclusion reasons and the prioritization rubric are reported in Supplementary Materials S2.1–S2.3; the full screening decision rules (OSF Attachments B) and the item–source tracing matrix (OSF Attachments H) are deposited in the OSF repository.

Construct specification. Perceived social support after disaster was defined as a survivor’s subjective retrospective appraisal of the support received and perceived as available across interpersonal, communal, institutional, and functional channels following a disaster event. The construct is situated at the perceived–received interface: items are retrospectively framed, asking survivors to evaluate their actual post-disaster support experience rather than hypothetical future availability. This framing reflects the disaster context specifically, in which the distinction between “what I believe is available” and “what I actually experienced” collapses within the retrospective appraisal window [13]. A preliminary model with seven candidate blocks—family–relative, peer–friend, significant-person, community–neighborhood, institutional, continuity–timing, and functional type—was derived deductively from the review evidence and from House [17], Cutrona and Russell [11], and Lakey and Orehek [12]. This is the definition stated in the Introduction, and it is used in that sense throughout: the construct is bounded to exclude prospective availability appraisal, and the phrase perceived–received interface carries no other meaning in this article.

Expert panel and item restructuring. A three-member advisor panel (scale development, psychometrics, and disaster psychology) evaluated a 43-item pool against item-writing standards [18], CFA requirements [37], and theoretical coherence. The panel identified three structural risks—source–function level confounding, wording inconsistencies, and significant-person heterogeneity—and restructured the pool into a five-factor hybrid model, reducing the set to 28 items and embedding continuity–timing content as an attribute within source-block wording (Supplementary Materials S2.3).

Delphi expert review (two rounds, *n* = 8). Eight experts rated each item on relevance, clarity, and representativeness (4-point scale; I-CVI ≥ 0.78 threshold; [38]). Round 1 (28 items): S-CVI/Average = 0.996 (relevance), 0.946 (clarity), 0.964 (representativeness); two items revised and three added, producing 31 items. Round 2 (31 items): S-CVI/Average reached 1.000, 0.981, and 0.996, respectively; Universal Agreement for relevance = 1.000. All items met threshold on all criteria (Supplementary Materials S3.1).

Cognitive interviews (*n* = 12 survivors). Semi-structured interviews (M = 58 min) with 12 diverse survivors (6 women; ages 22–67; nine provinces) using think-aloud, paraphrasing, confidence-rating, and specific-probing confirmed comprehensibility; five items received minor reformulation (e.g., replacing a double negation and anchoring the response window with an explicit date) (Supplementary Materials S3.5).

Pilot study (*N* = 150). The 31-item form was piloted with 150 survivors meeting main-study eligibility criteria. The pilot target followed the classical minimum ratio of five respondents per item (31 × 5 = 155), which the realized sample approximates; the a priori sample size estimation for the main study, based on multiple classical and model-based methods, is reported in Supplementary Materials S4.3. Block-level α ranged from 0.798 to 0.853; KMO = 0.909. A five-factor oblique EFA recovered a broadly interpretable structure; one item (legal counseling, item 31) remained consistently borderline (Supplementary Materials S4).

### 2.3. Participants

The target population comprised Turkish-literate adults (18+) directly affected by the 6 February 2023 Kahramanmaraş earthquakes (bereavement, injury, housing loss, or property loss). Purposive, multi-site recruitment maximized heterogeneity across province, loss type, and socioeconomic circumstance; fieldwork spanned eight affected provinces (Adana, Adıyaman, Diyarbakır, Gaziantep, Hatay, Kahramanmaraş, Malatya, and Osmaniye). Given the absence of a complete sampling frame, a nonprobability approach was used. Following prespecified data-quality screening (Supplementary Materials S5), 65 of 672 raw respondents (9.67%) were excluded (failure of two or more of the three attention checks, item-pool missingness ≥ 10%, straightlining ≥ 90%, or speeding < mean/3), yielding *N* = 607.

The sample comprised 333 men (54.9%) and 274 women (45.1%; M_age = 39.8, SD = 13.1). Education: primary or below (14.7%), middle school (13.8%), high school (33.1%), university (36.6%), postgraduate (1.8%). Employment: employed (38.1%), student (28.7%), homemaker (18.0%), unemployed (10.0%), retired (5.3%). Housing: permanent (57.8%), rental (24.2%), temporary (9.4%), with relatives or other (8.5%). Most severe loss: no major loss (45.3%), bereavement (22.9%), shelter loss (12.5%), community loss (7.6%), property loss (6.1%), livelihood loss (4.1%), health loss (1.5%). Eleven percent were currently receiving professional psychological support (Supplementary Materials S6).

### 2.4. Measures

PSSDS. The 31-item candidate form (5-point Likert; 1 = strongly disagree, 5 = strongly agree) was administered with recall anchored to “since the 6 February 2023 earthquakes, with particular emphasis on the past month.” All items are positively keyed; higher scores reflect greater perceived support. Three items (4, 5, and 6) were removed after calibration-stage analysis, yielding the retained PSSDS-28 with five blocks: Family–Relative Support (FRS; items 1–3), Social Environment Support (SES; items 7–12), Institutional Support (IS; items 13–18), Emotional Support (ES; items 19–24), and Instrumental–Informational Support (IIS; items 25–31) (Supplementary Materials S20). Terminology is used consistently throughout this article: block denotes one of these five scorable units of the PSSDS-28; factor denotes the corresponding latent variable in a factor-analytic model; and dimension is reserved for the two theoretical organizing axes, source and function. The three terms are not interchangeable, and subscale is not used as a synonym for any of them.

F-SozU K-3 ([23]; Turkish adaptation reported as a first-stage sub-study in [39]). Three items (5-point) assessing general perceived social support; administered as a convergent indicator. Ordinal α = 0.867 [95% CI 0.843, 0.891].

UCLA-6 ([40]; Turkish: [41]). Six items (4-point) assessing loneliness; higher scores indicate greater loneliness. Administered as an inversely keyed convergent indicator. Ordinal α = 0.808 [95% CI 0.780, 0.834].

Demographic and disaster-context form. Eleven items captured sociodemographic and disaster-context variables.

No social desirability measure was included. All PSSDS-28 items are positively keyed, creating a potential acquiescence or social desirability response set that the CMV diagnostics employed here are not designed to detect—those diagnostics target common method variance from shared measurement occasion and format rather than from motivational response tendencies. Future validation studies should include a brief social desirability measure (e.g., [42]) and test whether block scores remain unchanged after partialling out socially desirable responding.

### 2.5. Translation of the PSSDS-28 into English

The PSSDS-28 was developed and psychometrically validated in Turkish; the English version reported here was produced for international dissemination and was not itself administered in the present study. Translation followed the International Test Commission Guidelines for Translating and Adapting Tests [43] and COSMIN recommendations [35]. First, two independent bilingual translators, both native Turkish speakers with academic proficiency in English and familiarity with psychological measurement, produced independent forward translations. Second, the research team reconciled the two forward translations into a single synthesized version, resolving discrepancies through discussion and prioritizing conceptual and functional equivalence over literal correspondence. Third, one additional independent translator, a native English speaker blind to the source items, back-translated the synthesized version into Turkish. Fourth, an expert committee comprising the translators, a bilingual psychometrician, and a disaster-mental-health specialist compared each back-translation against the Turkish source and evaluated semantic, idiomatic, experiential, and conceptual equivalence [44], revising items for which equivalence was not fully achieved. The complete English item set is provided in Supplementary Materials S20. Because all psychometric analyses reported here were conducted on the Turkish administration, the English version should be regarded as a translated form whose cross-language measurement equivalence remains to be established in future English-speaking disaster samples.

### 2.6. Procedure

Data were collected face-to-face between May and June 2026, approximately 39 to 40 months after the 6 February 2023 earthquakes, using standardized paper-and-pencil administration by trained field researchers in quiet, private settings across eight provinces. This timing positions the assessment in the long-term recovery phase; according to the mobilization–deterioration model [13], this phase is characterized by declining informal support availability, which is relevant for interpreting block-level means and test–retest trends. Mean completion time was 11.97 min (SD = 3.84). Items were presented in a fixed order grouped by block (FRS first, then SES, IS, ES, and IIS), with three instructed-response attention-check items distributed through the form, one of them positioned between the IS and ES blocks. This blocked presentation may facilitate comprehension but could inflate within-block covariance through carry-over effects; the CMV diagnostics reported in Results provided limited indirect reassurance, but a randomized item-order administration should be compared against the blocked format in future research. Three instructed-response attention-check items were embedded (e.g., "Please mark ‘strongly disagree’ for this item"), each specifying a different correct response; respondents failing two or more were excluded at the data-screening stage (Supplementary Materials S5.1 and S5.2). Participants provided written informed consent; interviewers paused when distress emerged and directed participants to psychological support resources (ALO 183 crisis line). A volunteer retest subsample (*n* = 89) completed the PSSDS again approximately 21 days later (M = 20.8, SD = 2.3). Ethical approval was granted by the institutional review board; all procedures conformed to the Declaration of Helsinki [45].

Recall window. The recall instruction combined two windows: a long window bounded by a dated event (“since the 6 February 2023 earthquakes”) and a short window emphasizing the present (“with particular emphasis on the past month”). This was a deliberate design decision whose consequences should be stated. The long window preserves the disaster-specific framing that distinguishes the PSSDS-28 from general-purpose support measures; without it, respondents would appraise support in general rather than support in the aftermath of the earthquakes. The short window fixes the appraisal to a current, retrievable state, which is a precondition for the three-week test–retest design reported below and which limits the reconstruction demand placed on respondents 39 to 40 months after the event. The cost is that respondents may have weighted the two windows differently from one another, adding measurement error that the present design cannot quantify. Cognitive interviewing detected no confusion arising from the dual anchor; the explicit date was in fact added to the instruction page as a direct result of that stage, alongside the five item-level revisions (Supplementary Materials Table S3.5). Comprehension without confusion does not, however, establish that respondents weighted the two windows identically. The PSSDS-28 should therefore be regarded as calibrated for the long-term recovery phase, approximately two to four years after the event, in which it was developed. Its use in the acute and intermediate phases is plausible on content grounds but is not established here, and would require the anchor to be re-specified and the structure re-examined at each phase (Section 4.6).

### 2.7. Data Analysis Strategy

All PSSDS indicators were treated as ordinal [46]. Item-level missingness was negligible (0.48% overall; maximum 1.32%). Little’s MCAR test was consistent with data missing completely at random, χ^2^(1933) = 1322.94, *p* = 1.00; diagnostic logistic regression confirmed no demographic predictor of missingness (all *p* > 0.39). No imputation was performed (Supplementary Materials S7).

The cleaned sample (*N* = 607) was split into calibration (*n* = 305) and validation (*n* = 302) subsamples via stratified random allocation by gender × age group. Prespecified equivalence tests (Bonferroni-corrected α = 0.0042) confirmed subsample comparability (all *p* > 0.16) (Supplementary Materials S8).

Calibration analyses used the 31-item form: polychoric EFA (MINRES, oblimin; sensitivity with ULS and promax), parallel analysis, Velicer’s MAP, Kaiser criterion, scree elbow, and EGA (GLASSO + Walktrap; [47]; Leiden sensitivity; bootstrap B = 200). Item reduction triangulated pilot EFA, calibration EFA, and EGA item stability.

Validation analyses (PSSDS-28, *n* = 302) tested four CFA models with WLSMV estimation and theta parameterization: one-factor, correlated five-factor, second-order, and bifactor. Fit was evaluated with CFI, TLI, RMSEA (90% CI), and SRMR against accepted guidelines (CFI/TLI ≥ 0.95; RMSEA ≤ 0.08; SRMR ≤ 0.08). Target-rotated ESEM served as a cross-loading sensitivity check; bootstrap CFA (*B* = 200) quantified sampling variability.

The validation subsample (*n* = 302) yields an observation-to-parameter ratio of approximately 2:1 for the 28-item five-factor model—below the textbook recommendation of 5:1 [48]. This ratio warrants interpretive caution. All four confirmatory models nonetheless converged with no Heywood cases and a positive-definite latent covariance matrix. Bootstrap re-estimation (B = 200) yielded 97.5% convergence for the five-factor model, with narrow fit-index distributions (CFI SD = 0.009, RMSEA SD = 0.005), supporting the numerical stability of the reported estimates.

Full-sample analyses (*N* = 607): (a) ordinal measurement invariance (configural → metric → scalar; nine grouping variables; [49], Δ-fit criteria); (b) block reliability (ordinal α, ω total, CR, AVE; bootstrap *B* = 500); (c) test–retest stability (ICC [2,1], two-way random effects, absolute agreement; *n* = 89; Bland–Altman plots); (d) construct validity (HTMT ratios; fully latent external associations with FDR correction); (e) blockwise IRT (GRM vs. GPCM; M2 fit; S-X^2^; Yen Q3; Mokken *H*); (f) network psychometrics (EBICglasso, γ = 0.50; EGA + bootEGA, *B* = 500; centrality CS stability; split-half replication; Goldbricker redundancy); (g) CMV diagnostics (Harman single-factor; CFA one-factor comparison; common latent factor model; HTMT); and (h) DIF screening (ordinal logistic regression; Holm adjustment; ΔMcFadden R^2^ ≥ 0.010 threshold). A fixed seed (20240315) was used in all resampling. Analyses were conducted in R version 4.5.2 (R Core Team, Vienna, Austria) using lavaan, psych, EGAnet, mirt, mokken, bootnet, qgraph, networktools, mgm, and ancillary packages. During manuscript preparation, the authors used large language model tools for language editing and statistical-code drafting; all AI-assisted output was reviewed and approved by the authors, who accept full responsibility for the content.

## 3. Results

### 3.1. Phase 1 Summary and Item Reduction

Content validity was strong at both Delphi rounds, with S-CVI/Average ≥ 0.946 for all criteria and achieving 1.000 for relevance in Round 2. Cognitive interviews confirmed 26 of 31 items required no revision. Pilot block-level α ranged from 0.798 to 0.853 with KMO = 0.909.

In the calibration subsample, polychoric EFA at *k* = 5 yielded RMSR = 0.043, with RMSEA = 0.124 [0.118, 0.130] and TLI = 0.731; only the residual index met conventional criteria, a pattern consistent with the documented upward bias of RMSEA and downward bias of TLI in polychoric EFA with many items and oblique factors (Supplementary Materials S10.4). Of the retention methods applied, parallel analysis, Velicer’s MAP and the Kaiser criterion indicated *k* = 7 and the automatic scree elbow indicated *k* = 5; these indices were prespecified as diagnostic rather than decisive, and *k* = 5 was retained on theoretical grounds and on the convergent EGA evidence reported next (Supplementary Materials S10.3). EGA recovered five communities exactly matching theoretical blocks. Three family–relative items (4, 5, and 6) converged on removal: pilot EFA flagged item 5 for cross-loading, with items 4 and 6 unflagged at that stage; calibration EFA placed all three toward the social environment factor; bootstrap EGA item stability was 0.390–0.395, the three lowest values in the 31-item pool. Five further items fell below 0.96 in that solution—item 7 (0.645), flagged as near-boundary and monitored, items 25 and 26 (0.910 each), item 27 (0.920), and item 12 (0.955)—while the remaining items ranged from 0.960 to 1.000. None of the five met the convergent removal criterion; all were retained, and all reached 0.965 or above in the 28-item solution (Supplementary Materials Tables S10.18 and S10A.21). No other items showed convergent removal evidence across all three streams. Following removal, 28-item bootstrap EGA recovered five communities in 97.0% (nonparametric) to 98.5% (parametric) of resamples, with mean item stability rising from 0.915 to 0.994 and minimum stability from 0.390 to 0.965. A dissenting signal—calibration EFA losing SES–IS separation after FRS shrank to three items—was recorded transparently; EGA maintained separation under all conditions, suggesting the EFA signal reflected a rotation artifact (Supplementary Materials S10).

### 3.2. Confirmatory Factor Structure (Validation Subsample, N = 302)

Four competing models were estimated (Table 2). All model chi-square values were significant (*p* < 0.001), as is expected at this sample size; model comparison therefore relied on descriptive fit indices and BIC. The one-factor model fit poorly (CFI = 0.732, RMSEA = 0.180, SRMR = 0.194). The correlated five-factor model fit well: χ^2^(340) = 927.73, *p* < 0.001, CFI = 0.954, TLI = 0.949, RMSEA = 0.076 [0.070, 0.082], SRMR = 0.072 (BIC = 24,327.80), outperforming all alternatives with no Heywood cases and 97.5% bootstrap convergence. The second-order (CFI = 0.922, RMSEA = 0.098) and bifactor (CFI = 0.923, RMSEA = 0.101) models were tenable but inferior; bifactor indices indicated a substantial general factor (ECV = 0.497, ω_h = 0.932) alongside meaningful block-specific variance (ω_specific = 0.628–0.792). The correlated five-factor model was retained on grounds of fit, parsimony, interpretability, and the absence of a theoretical reason to impose hierarchical or independence constraints. The RMSEA marginally exceeding 0.06 is partly attributable to the modest observation-to-parameter ratio (~2:1) (Supplementary Materials S11).

**Figure 1:**
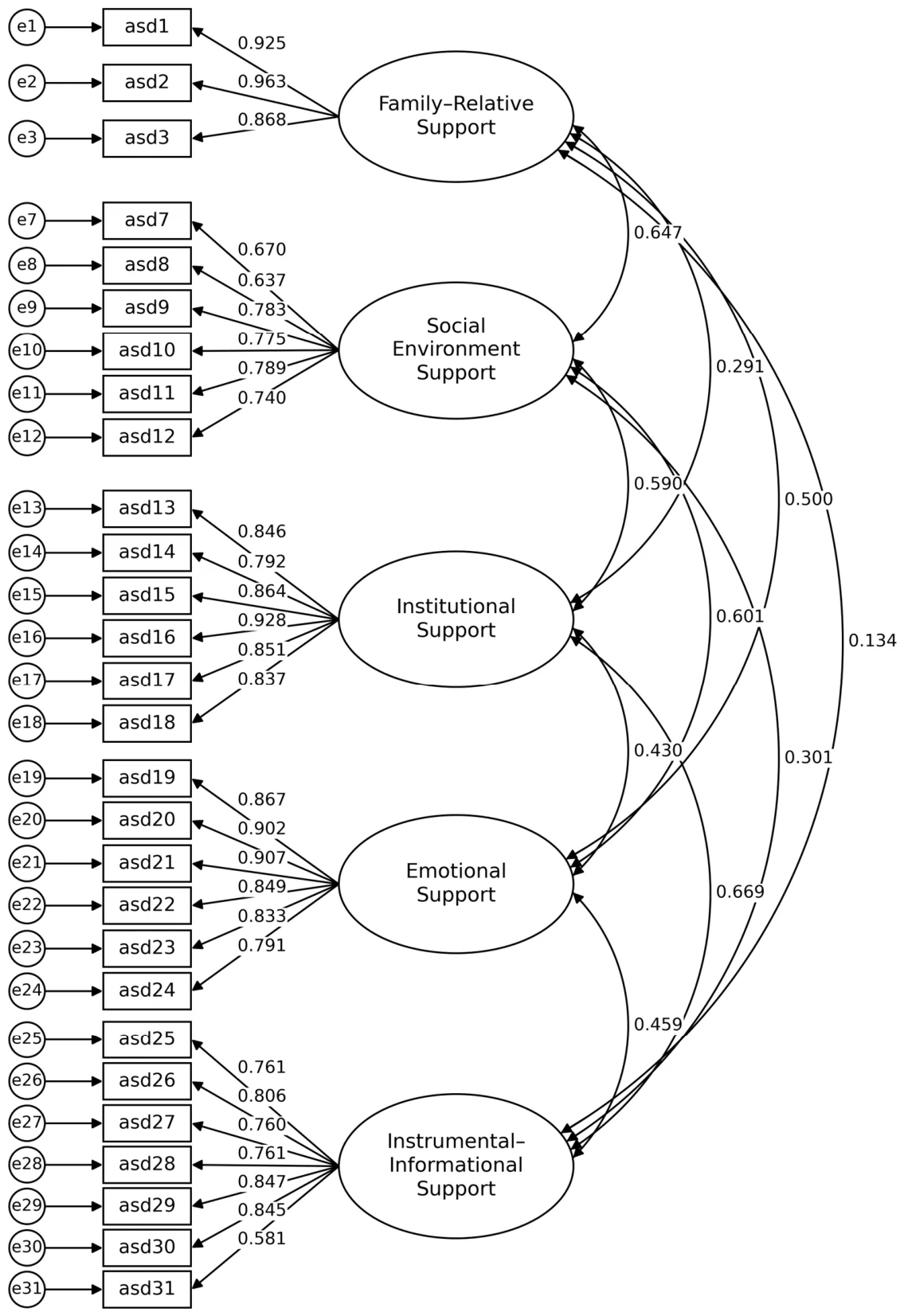
Path diagram of the correlated five-factor CFA model (PSSDS-28; validation subsample, *N* = 302; 28-Item Model B). Note. Standardized WLSMV factor loadings on indicator paths; factor intercorrelations as curved double-headed arrows. All loadings significant at *p* < 0.001. FRS = family–relative support; SES = social environment support; IS = institutional support; ES = emotional support; IIS = instrumental–informational support.

Standardized loadings ranged from 0.581 to 0.963, with only item 31 (legal counseling, λ = 0.581) falling below 0.65 (Figure 1; Table 3). Latent inter-factor correlations in this subsample ranged from 0.134 (FRS–IIS) to 0.669 (IS–IIS); the corresponding full-sample range was 0.137 to 0.611 (Supplementary Materials Tables S11.3 and S14.2). All values fell below 0.70, ruling out block redundancy. Bootstrap CFA (*B* = 200) confirmed narrow fit-index distributions (CFI *SD* = 0.009, RMSEA *SD* = 0.005).

Target-rotated ESEM improved SRMR (0.072 → 0.040) and maintained CFI (0.960), at the cost of a more complex parameter space. Cross-loadings ≥ 0.20 fell from 54.8% in the 31-item set to 39.3% in the 28-item set, confirming through a second method that the three removed items had introduced substantive cross-loading complexity. The highest secondary loading in the 28-item ESEM was 0.378 (item 26 onto SES) (Supplementary Materials S11.7).

### 3.3. Reliability

Internal consistency was consistently high (Table 4). Ordinal α ranged from 0.852 to 0.949; ω total was virtually identical (0.854–0.950), confirming insensitivity to coefficient choice. CR ranged from 0.865 to 0.950; AVE exceeded 0.50 for all blocks (0.518–0.863). Alpha-if-deleted was examined against two reference sets. Against the 31-item administered pool, item 31 was the only item whose removal would raise the coefficient, and the margin was +0.0005 (Supplementary Materials Table S9.2). Within blocks the pattern differs: removing item 31 lowers the IIS coefficient from 0.900 to 0.893, whereas removing item 3 raises the three-item FRS coefficient from 0.949 to 0.951; no other item raises its block coefficient (Supplementary Materials Table S13.2). Neither margin is of a magnitude that would justify deletion.

Test–retest stability (*n* = 89; interval ≈ 21 days) differentiated meaningfully across blocks (Table 4). Stability was interpreted against the benchmarks of Koo and Li [50], where an ICC below 0.50 is poor, 0.50–0.75 moderate, 0.75–0.90 good, and above 0.90 excellent. The emotional and instrumental–informational blocks were excellent (ICC = 0.909 and 0.892) and the institutional block was good (0.859), whereas the social environment and family–relative blocks were moderate (0.607 and 0.542). The FRS ICC of 0.542, while technically moderate, approaches the poor–moderate boundary and warrants cautious interpretation for longitudinal change detection.

Bland–Altman analyses revealed statistically significant small-to-moderate negative mean differences (T2 < T1) in FRS, SES, and IS blocks (mean_diff = −0.588, −0.348, −0.114; all *p* ≤ 0.016). Three non-mutually exclusive explanations are considered. First, perceived family and social support may have genuinely declined, consistent with mobilization–deterioration dynamics [13]. Second, the FRS block’s three-item length limits measurement precision, inflating temporal variability independent of true change. Third, retrospective appraisal of family support may be more susceptible to mood-state fluctuations than appraisal of formal institutional support. The present design cannot disentangle these explanations; the retest-based block-level SEM and MDC_95_ values reported in Section 3.7 provide a concrete standard for judging whether observed changes exceed measurement error. Complete reliability estimates are provided in Supplementary Materials S13.

Item 31 sensitivity. Because the Discussion considers reporting item 31 (legal counseling) as a standalone indicator, we examined what would follow if it were also removed from the IIS composite. Table 5 compares the seven-item IIS used throughout this article with the six-item alternative. Removing item 31 lowered internal consistency (ordinal α 0.900 [0.884, 0.913] → 0.893 [0.873, 0.908]; ω total 0.901 → 0.893) and test–retest stability (ICC = 0.892 → 0.871), widened the standard error of measurement and the minimal detectable change (retest SEM 0.255 → 0.293; MDC_95_ 0.706 → 0.813), left discriminant separation from the institutional block essentially unchanged (HTMT = 0.631 → 0.634), and raised the block mean by 0.09 (2.99 → 3.08). The last of these is substantively the most consequential: item 31 has the highest floor rate in the instrument (38.35%; Supplementary Materials S9.1), so excluding it removes the clearest available signal of unmet need from the composite. All seven items are therefore retained in the IIS composite, and every psychometric property reported in this article refers to the seven-item block (Supplementary Materials S18.6).

### 3.4. Construct Validity

Discriminant validity. All inter-block HTMT ratios fell below 0.85 (Table 6). The three highest pairs—SES–IS (0.612), SES–ES (0.613), IS–IIS (0.631)—marked the least separable combinations but remained below threshold. Each block’s √AVE exceeded the highest HTMT ratio involving that block (0.720–0.929), satisfying the Fornell–Larcker criterion [51].

Convergent validity. Fully latent associations matched predictions (Table 6). All blocks showed positive associations with general perceived support (F-SozU K-3; *r* = 0.216–0.673) and negative associations with loneliness (UCLA-6; *r* = −0.409 to −0.517); all significant after FDR correction (*p*_fdr < 0.001). The gradient was theoretically interpretable: interpersonal blocks (FRS: 0.673; ES: 0.665) correlated most strongly with general perceived support, while institutional and instrumental–informational blocks correlated most modestly (IS: 0.248; IIS: 0.216), reflecting that these tap formal, resource-based content not primarily indexed by everyday-support scales. Loneliness associations were more uniform (−0.409 to −0.517), consistent with loneliness bearing an inverse relationship to perceived support regardless of source.

Known-groups validity. Under scalar invariance, participants not receiving professional psychological support reported higher latent means on FRS (+0.533 *SD*), SES (+0.609 *SD*), and ES (+0.409 *SD*; all *p*_fdr ≤ 0.010). No differences emerged for IS or IIS, indicating the pattern is specific to the interpersonal and emotional blocks—theoretically coherent given that professional help-seeking is more likely when informal support is low. No FDR-corrected differences were observed for age, gender, marital status, education, or employment. Complete construct-validity tables are provided in Supplementary Materials S14.

### 3.5. Measurement Invariance

The correlated five-factor model was tested for ordinal measurement invariance (WLSMV, theta) across nine grouping variables, six of which were feasible (Table 7). For all six—age group, gender, marital status, education, employment, and professional psychological support—configural, metric, and scalar invariance satisfied prespecified Δ-fit criteria. The largest scalar–metric deterioration (employment: ΔCFI = −0.0051, ΔRMSEA = +0.0012, and ΔSRMR = −0.0023) was comfortably within all thresholds. This warrants confidence that latent mean comparisons across these groupings reflect genuine differences rather than measurement artifacts.

For income, loss type, and housing status, the configural model was not estimable because small subgroups produced empty item × response-category cells—a sampling-distribution constraint rather than a psychometric property of the scale, constituting a priority for replication with larger samples. Full invariance results are reported in Supplementary Materials S12.

### 3.6. Supplementary Psychometric Analyses

IRT and Mokken scaling. Block-level GRM outperformed GPCM in all blocks (ΔAIC = −82.9 to −138.0). Item discrimination was generally high (SES: a = 1.37–2.70; IS: 2.38–4.62; ES: 2.33–4.39; IIS: 1.38–2.99). An extreme, imprecise estimate in FRS (item 2: a = 17.55, SE = 8.95) most likely reflects unmodeled local dependence in short, homogeneous blocks [52,53] rather than genuine discriminative power. Global M2 indicated notable misfit in SES, IS, ES, and IIS blocks (RMSEA[M2] = 0.122–0.211), M2 could not be computed for the three-item FRS block, which provides insufficient degrees of freedom for the statistic, so no M2-based statement about FRS fit is possible (Supplementary Materials Table S15.6). Item-level S-X^2^ tests (Bonferroni-corrected) flagged only two items (SES items 10 and 11, both related to community solidarity). Yen’s Q3 residuals exceeded |0.20| for 40 within-block item pairs (FRS 2, SES 7, IS 9, ES 9, IIS 13; Supplementary Materials Table S15.3), interpretable as content proximity rather than structural mis-specification. The pattern of widespread global M2 misfit alongside sparse item-level misflagging is consistent with M2’s known high power to detect minor local dependence in large samples [54]. These results do not invalidate IRT analyses but caution against strong claims based on IRT θ estimates for SES and IIS; CFA-based factor scores are recommended for applied use. Mokken scalability was strong in four blocks (H = 0.534–0.798) and moderate in SES (H = 0.427), with zero monotonicity or IIO violations (Supplementary Materials S15).

**Figure 2:**
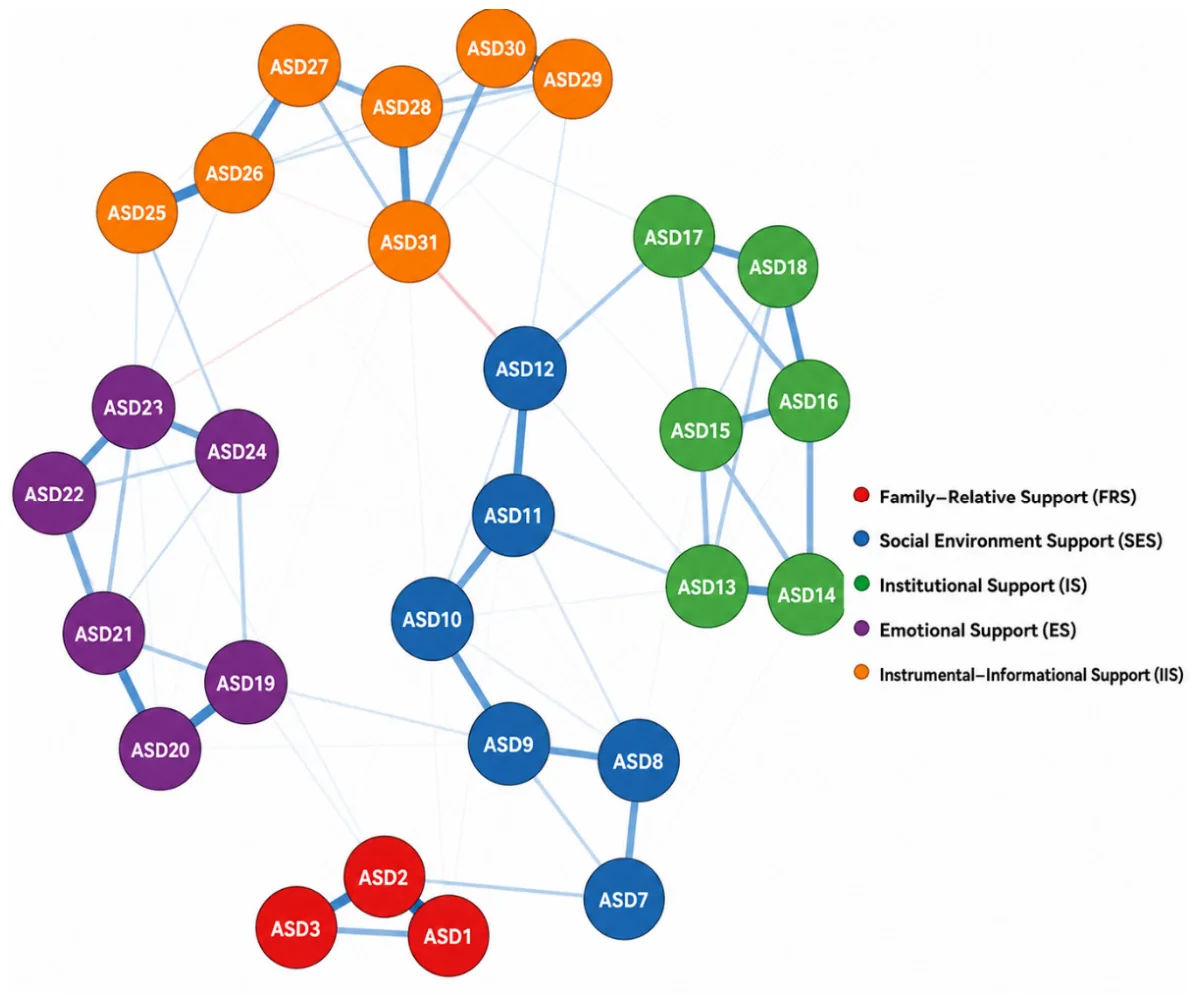
PSSDS-28 primary network (EBICglasso; γ = 0.50; threshold = FALSE; N_cc = 548). Note. Node colors represent CFA block membership (F1–F5). Edge widths are proportional to partial correlation strength.

Network psychometrics. An EBICglasso model (γ = 0.50; *N*_cc = 548) yielded 172 of 378 nonzero edges (sparsity = 0.545). EGA recovered five communities exactly matching CFA blocks (ARI = NMI = 1.000), replicated in all 500 bootstrap resamples (Figure 2). The structure was robust to γ variation (*r* = 0.994 between γ = 0.50 and 1.00) and showed good split-half replication (*r* = 0.884). Centrality stability was acceptable for Strength (CS = 0.440) and strong for Expected Influence (0.595) but low for Closeness (0.206) and Betweenness (0.049); centrality interpretations are restricted accordingly. Goldbricker screening returned no flagged redundant pairs; because the archived output does not distinguish an empty result from a comparison that did not run, this is best read as an absence of evidence for item redundancy rather than as positive evidence of its absence. Mean nodewise R^2^ = 0.538; SWI = 1.041 (Supplementary Materials S16).

Common-method variance. Four complementary diagnostics were employed. Harman’s single-factor test—retained as a widely recognized legacy diagnostic despite acknowledged limitations [55,56]—yielded a first unrotated factor explaining 32.04% of variance, below the 50% threshold. More informatively, in the full sample, the one-factor CFA fit markedly worse than the five-factor model (N_cc = 548; ΔCFI = 0.245, ΔRMSEA = 0.114); the corresponding comparison in the validation subsample is reported in Section 3.2. A common latent factor added to CFA-5 produced negligible improvement (ΔCFI ≈ 0.001; mean |Δλ| = 0.020, maximum = 0.039; mean CLF loading ≈ 0.169). All HTMT ratios remained below 0.85. Together, these results do not support CMV as a plausible alternative explanation (Supplementary Materials S17).

Differential item functioning. DIF screening across six invariance-supported variables (168 item × group combinations) detected no practically meaningful DIF: no combination exceeded ΔMcFadden R^2^ ≥ 0.010. A single statistically significant signal (item 26, education, Holm-adjusted *p* = 0.041) was accompanied by ΔR^2^ = 0.008, consistent with large-sample statistical inflation [57] (Supplementary Materials S18.1).

### 3.7. Score Distributions

Full-sample block means (1–5): FRS *M* = 4.17 (*SD* = 1.10), SES = 3.77 (0.90), IS = 3.29 (1.17), ES = 3.78 (1.03), IIS = 2.99 (1.06). Total *M* = 3.52 (0.76). Alpha-based SEM and MDC_95_ were: FRS = 0.249/0.689; SES = 0.347/0.961; IS = 0.291/0.808; ES = 0.255/0.706; IIS = 0.335/0.928 (Supplementary Materials Table S18.5). Retest-based values, derived from the ICCs in Table 4, are systematically larger where temporal stability is lower: FRS = 0.727/2.015; SES = 0.454/1.259; IS = 0.318/0.880; ES = 0.241/0.668; IIS = 0.251/0.696 (Supplementary Materials Table S13.4). Because the alpha-based figures treat all non-error variance as stable over time, the retest-based values are the appropriate reference for judging whether an observed change exceeds measurement error, and the alpha-based values for judging the precision of a single administration. Neither set constitutes a clinical cut-off score. Item-level descriptive statistics are provided in Supplementary Materials S9.

## 4. Discussion

The PSSDS-28 provides initial validation evidence for a self-report instrument designed to capture perceived social support after disaster along source and function axes. Across a multi-method evaluation, findings converged consistently on a five-block structure—family–relative, social environment, institutional, emotional, and instrumental–informational support. This convergence held across factor-analytic, network-based, item-response-theoretic, and method-variance approaches, making the conclusion unlikely to be an artifact of any single analytic decision.

### 4.1. Theoretical Interpretation of the Factor Structure

Before interpreting each block, the hybrid nature of the structure warrants acknowledgment. The PSSDS-28 does not instantiate a fully crossed source × function matrix (which would require, e.g., separate blocks for “emotional support from family” versus “emotional support from institutions”). Instead, source-organized blocks implicitly carry functional content (family items are predominantly emotional; institutional items are predominantly instrumental) and function-organized blocks implicitly reference multiple sources (emotional support items do not specify whether the person is kin, neighbor, or aid worker). This reflects a deliberate compromise between theoretical comprehensiveness and cognitive accessibility. During item development, the expert panel restructured the pool into a hybrid five-factor model, specifically to avoid source–function-level confounding, rather than imposing a fully crossed matrix that would multiply item count and cognitive demand. Researchers requiring strict source × function separability should supplement the PSSDS-28 with items explicitly crossing both dimensions.

The practical consequences of this design choice are best stated as a scope condition rather than as a caveat. The hybrid architecture is sufficient whenever the research question concerns the standing of a support channel or a support function in its own right—whether perceived institutional support predicts posttraumatic stress independently of interpersonal support, whether emotional and instrumental–informational provision diverge during long-term recovery, or whether survivors in temporary housing perceive institutions as less responsive than survivors in permanent housing. The quantities of interest in such questions are marginal ones, and marginal quantities are precisely what source-organized and function-organized blocks estimate. The architecture becomes insufficient as soon as a question requires a specific cell of the source × function matrix: whether instrumental support is perceived as more available from kin than from formal agencies, whether the buffering value of emotional support depends on who provides it, or whether an intervention that increases institutional instrumental provision leaves institutional emotional provision unchanged. Questions of this second type require either a fully factorial instrument or, more economically, a small set of supplementary items that cross the two dimensions explicitly for the cells of theoretical interest. Table 8 makes this boundary concrete.

The separation of emotional from instrumental–informational support maps onto optimal-matching theory [11]: survivors distinguish being emotionally accompanied from being practically assisted—the contrast the theory treats as most consequential for support–demand fit. Post-disaster, both controllable and uncontrollable demands coexist, and the PSSDS-28’s structure positions researchers to assess whether available support matches this mixed profile.

Institutional support differentiation is theoretically important for a different reason: it captures a channel with no analogue in most existing measures, one with both psychological and policy relevance [15,16]. The mean IS score (3.29) was substantially lower than FRS (4.17) and ES (3.78), echoing the literature’s finding that interpersonal channels are perceived as more available than formal ones [13]. Similarly, IIS attracted the lowest mean (2.99), suggesting systematic shortfalls in tangible resource and information access—findings with direct intervention implications that a single global score would average away.

The distinctness of institutional from instrumental support warrants explicit justification, since the two overlap in practice. The distinction is one of level rather than of content: in House’s typology [17], the provider of support and the function it serves are logically independent, and institutional support is a provider category whose content is not exhausted by tangible provision. Being treated fairly by an agency, being recognized as eligible, and being able to trust that a service will still exist next month are appraisals about an actor rather than about a resource, and the institutional-trust literature shows that such appraisals carry associations with mental health that are not reducible to the resources delivered [15,16]. The present data are consistent with this reading: the two blocks correlate at 0.611 in the full sample and 0.669 in the validation subsample, both below the 0.70 redundancy threshold; their HTMT ratio is 0.631; exploratory graph analysis separates them in every one of 500 bootstrap resamples; Goldbricker screening flags no redundant item pair across them; their means differ (3.29 versus 2.99); their retest stabilities differ (0.859 versus 0.892); and their associations with general perceived support differ (0.248 versus 0.216). Two blocks this consistently separable are not one block measured twice.

The IS–IIS correlation (0.611 in the full sample; 0.669 in the validation subsample) reflects the expected overlap between institutional delivery and instrumental content; the moderate-to-high HTMT values among SES, IS, and ES (0.612–0.631) reflect real-world overlap in post-disaster support. That EGA, Mokken analysis, and bootstrap EGA nevertheless separated these blocks cleanly under every condition suggests the overlap is partial and the distinctions statistically meaningful—exactly what a multidimensional model should find.

The bifactor results showed a substantial general factor (ECV ≈ 0.50, ω_h = 0.932) alongside meaningful block-specific variance (ω_specific = 0.628–0.792). The general factor lends coherence to the total score as a summary indicator; the specific variances and the inferior fit of bifactor and second-order models indicate this general variance does not exhaust block information. The correlated five-factor model’s superiority reflects not that general variance is absent but that forcing it through hierarchical or independence parameterization misrepresents the data.

### 4.2. Measurement Invariance and Practical Comparability

Scalar invariance across six groupings enables the comparative work for which a disaster-assessment tool is most immediately useful: observed group differences in latent means can be attributed to genuine variation rather than differential item interpretation. The inability to test invariance across income, housing, and loss type represents the highest-priority gap for replication with larger, category-balanced samples, as these are substantively the most consequential variables for understanding inequality in support access.

The known-groups finding—lower FRS, SES, and ES among those receiving professional help, with no IS or IIS differences—extends invariance results by demonstrating theoretically ordered group differences. The block specificity suggests professional support-seeking is tied to perceived shortfalls in interpersonal and emotional domains rather than formal or instrumental ones—consistent with theoretical accounts of when individuals seek professional help [12]. However, the cross-sectional design cannot establish whether low perceived support precedes help-seeking or whether help-seeking reduces informal support over time.

### 4.3. Methodological Tensions

Two instructive tensions deserve comment. First, calibration EFA and EGA diverged when FRS was reduced to three items: EGA maintained SES–IS separation; EFA merged them. EGA’s advantage is that its community detection does not involve oblique rotation and is insensitive to factor-space rebalancing. The lesson is that EFA and EGA discrepancies are informative about data geometry rather than resolvable by choosing one as “correct.”

Second, CFA and IRT diverged for FRS: CFA represented it as a strong factor (0.868–0.963), while IRT produced an extreme discrimination estimate (*a* = 17.55, *SE* = 8.95) reflecting local-dependence amplification in short homogeneous blocks [52,53]. This suggests three-item blocks may need testlet or constrained-IRT parameterization when IRT diagnostics are required.

### 4.4. Practical Implications

The block-differentiated profile supports several applied uses unavailable from a single score. In needs assessment, the scale can identify mismatches—abundant emotional support alongside weak institutional responsiveness—providing data-driven maps for intervention resources. Retest-based block-level SEM and MDC_95_ values give evaluators concrete decision rules for interpreting pre–post change. For institutional actors, IS and IIS blocks provide empirical indicators of perceived outreach and service adequacy not captured by satisfaction surveys or objective provision statistics.

Two applied settings show what block-differentiated scoring adds. In post-disaster needs assessment, an instrument that returns a single support score cannot distinguish a community whose informal networks have absorbed the shock but whose formal services are absent from a community in which both have failed; the IS and IIS blocks separate these situations directly, and the two situations call for different responses. Because block-level SEM and MDC_95_ values are available (Section 3.7), field teams can also judge whether a difference between two districts, or between two assessment waves in the same district, exceeds measurement error rather than relying on an unaided comparison of means. In psychosocial service planning, the same profile logic indicates which service to scale: a low ES score alongside an adequate FRS score suggests that emotional needs exceed what kin networks can absorb, so counseling capacity is the binding constraint, whereas a low IIS score alongside an adequate IS score suggests that agencies are present but that tangible provision and information are not reaching survivors—a delivery problem rather than a coverage problem. Neither inference is available from a global score, and neither requires criterion-referenced cut-off values.

The PSSDS-28 is not a clinical diagnostic instrument, and no cut-off scores are proposed. Applications to clinical screening require criterion-referenced calibration in independent samples with identified mental health outcomes (e.g., PTSD diagnosis and clinically significant depression) before any triage function can be responsibly claimed. The known-groups finding that help-seekers reported lower FRS, SES, and ES is consistent with screening use but does not validate one. The cross-sectional design cannot establish whether low support precedes help-seeking, whether help-seeking displaces informal support, or whether both reflect a common third variable. Researchers and practitioners should treat the PSSDS-28 as a research and needs-assessment instrument at this validation stage and report block scores alongside established instruments pending criterion validity evidence.

### 4.5. Limitations and Future Directions

The limitations of this work fall into three groups: structural limitations arising from the hybrid architecture; sampling and generalizability limitations arising from a single event type, a single culture, and a realized sample below target; and evidentiary limitations arising from the absence of criterion-related and discriminant evidence. They are set out in that order and are not repeated in the Conclusions. The first, cutting across all findings, concerns the hybrid rather than fully factorial architecture. The scale does not permit determining whether, for example, instrumental support is perceived as available from family specifically or from institutions specifically—only that instrumental support in general is perceived, and separately, that family support in general is perceived. For research questions requiring cell-level information, the PSSDS-28 would need supplementation with items explicitly crossing source and function. The hybrid design was adopted during expert-panel restructuring as a practical solution to source–function level confounding and item-count constraints, but it represents an incomplete operationalization of the theoretical source-by-function space.

First, the PSSDS-28 was developed and validated exclusively in a Turkish sample affected by a single event type (earthquake). Item wording is disaster-type agnostic, but factor structure, reliability, and validity should be independently established across floods, wildfires, hurricanes, technological disasters, and conflict-related displacement. Cross-cultural adaptation using established protocols is necessary before use outside Turkish-speaking populations. Applicability across event types is not uniform at the item level. Items referring to emotional accompaniment, neighborhood solidarity, and institutional responsiveness are event-agnostic in wording, whereas items referring to shelter provision and to administrative or legal processes presuppose a sudden-onset event with property destruction and a formal relief architecture. Three transfers therefore require content review rather than translation alone: epidemics, in which physical proximity is itself restricted and support delivery changes character; armed conflict and forced displacement, in which state institutions may be a source of threat rather than of support, inverting the meaning of the institutional block; and slow-onset events such as drought, for which a dated event anchor cannot be specified.

Second, the realized sample (*N* = 607) fell short of the a priori target (*N* = 800), and the validation subsample’s observation-to-parameter ratio (~2:1) fell below the 5:1 recommendation [48]. Bootstrap re-estimation nonetheless indicated numerically stable estimates; the three-item FRS block, however, introduced intermittent convergence issues (97.5% bootstrap rate), and its estimates should be interpreted with greater caution. Independent replication with *n* ≥ 500 in a validation subsample remains a priority.

A further sampling limitation concerns who remained reachable. Data were collected 39 to 40 months after the earthquakes, so the sample necessarily excludes survivors who had relocated permanently outside the affected provinces, who had died, or whose functional impairment precluded participation. Because survivors who remain in a disaster region are on average those with more intact local networks and better access to services, this constraint is more likely to bias interpersonal and institutional support estimates upward than downward, which makes the low institutional (3.29) and instrumental–informational (2.99) means more rather than less notable. Multi-site recruitment across eight provinces, with 42.2% of the sample not in permanent housing and 54.7% reporting a major loss, attenuates this constraint without removing it. Prospective cohorts that track displaced survivors are needed to estimate its magnitude.

Third, the cross-sectional design—even supplemented with a three-week retest—cannot test the theoretically central continuity–timing dynamics of the mobilization–deterioration model. Longitudinal designs with acute, intermediate, and long-term measurements are needed to examine whether the five-block profile changes as predicted and whether specific blocks’ protective value is moderated by recovery phase.

Fourth, measurement invariance could not be established for income, loss type, or housing—the variables most relevant to post-disaster socioeconomic inequality research.

The most consequential validity gap is the absence of criterion-related evidence. Both external measures (F-SozU K-3 and UCLA-6) are conceptually proximal, yielding convergent–convergent associations confirming the scale measures something in the support–connection domain but not establishing predictive utility for outcomes of direct relevance. We did not assess PTSD symptoms, depression severity, posttraumatic growth, functional recovery, or help-seeking behavior. Without demonstrating that block scores predict these outcomes incrementally beyond established measures, the practical value proposition remains theoretical. We additionally did not include measures of constructs that should be discriminantly unrelated (e.g., general self-efficacy and trait positive affect), leaving discriminant validity incomplete. Two criterion-validity investigations constitute highest-priority next steps: (1) head-to-head comparison with the MSPSS testing incremental prediction of PTSD and depression and (2) known-groups comparisons using clinically identified groups. Until completed, the PSSDS-28 should be used in conjunction with rather than instead of established measures.

Because the practical value of the PSSDS-28 depends on this evidence, it is worth specifying what would establish it. A first study would administer the PSSDS-28 together with the MSPSS and criterion measures of posttraumatic stress (e.g., the PCL-5 [58]), depressive symptoms (e.g., the PHQ-9 [59]), functional recovery (e.g., WHODAS 2.0 [60]), and posttraumatic growth (e.g., the PTGI [61]), together with a validated measure of help-seeking intention, and would test whether the five blocks explain incremental variance in each criterion beyond the MSPSS total score. The directional predictions are specific enough to be falsified: emotional support should show the strongest inverse association with posttraumatic stress and depressive symptoms, as optimal-matching theory predicts for uncontrollable loss [11]; institutional and instrumental–informational support should show the strongest associations with functional recovery, as the same theory predicts for controllable resource demands; and low family–relative and emotional support should predict help-seeking, extending the known-groups pattern reported here into a prospective test. A second study would compare clinically identified groups—survivors meeting diagnostic criteria for posttraumatic stress disorder or major depression against matched survivors who do not—in order to test known-groups discrimination and to generate the sensitivity and specificity information that any future screening application would require. Both designs are feasible at sample sizes comparable to the present one: detecting an incremental ΔR^2^ of 0.02 for the five blocks over the MSPSS with 80% power at α = 0.05 requires approximately 520 participants, assuming a total model R^2^ of about 0.20. Until this evidence exists, block scores should be interpreted as descriptive indicators of perceived support standing rather than as predictors of clinical outcome.

Fifth, item 31 (legal counseling) presented a retention dilemma throughout analysis. Statistically, it is the weakest item: lowest loading (0.574 in the 31-item model and 0.581 in the 28-item model), highest floor rate (38.35%), and lowest IRT discrimination in IIS. Substantively, it captures a domain—access to legal aid following displacement and bereavement—absent from all existing perceived-support instruments. We resolved this by retaining the item, on both content-validity and psychometric grounds: the sensitivity analysis in Section 3.3 shows that removing it from the composite lowers internal consistency and temporal stability, widens the minimal detectable change, and raises the block mean by masking the very unmet need the item captures. The scoring rule is therefore unambiguous: item 31 contributes to the IIS composite in all applications, and every psychometric property reported here refers to the seven-item block. In applied needs-assessment contexts, we additionally recommend reporting item 31 on its own, as a supplementary descriptive indicator of unmet legal-aid need, because its high floor rate carries information that any composite necessarily averages away. This is an addition to the composite score, not a substitute for it. Future development should consider replacing item 31 with a broader formulation (e.g., “I was able to navigate official administrative procedures and obtain institutional guidance”).

Sixth, the exclusively positive item keying and absence of a social desirability measure preclude separating substantive response variance from acquiescence and socially desirable responding. In the disaster context, endorsing low family or community support may carry social stigma, potentially suppressing low-end responding on interpersonal blocks. Future revisions should explore reverse-keyed items and pair administration with a social desirability screener (e.g., [42]).

A conceptual limitation deserves explicit acknowledgment. The PSSDS-28 items are retrospectively framed—they ask what support survivors received and experienced rather than what they believe would be available in the future. This positions the construct at the perceived–received interface [6] rather than at the purely prospective availability pole emphasized by Cohen and Wills [4] and operationalized by instruments such as the MSPSS. Whether retrospective support appraisals carry the same predictive and buffering properties as prospective availability perceptions is an open question. Future research should administer the PSSDS-28 alongside a prospective availability measure and examine whether the two forms differentially predict PTSD, depression, and recovery outcomes. A related boundary condition concerns the recall instruction itself. Because it combined a long, event-anchored window with a short window emphasizing the past month (Section 2.6), the scores reported here should be read as appraisals formed during long-term recovery rather than as estimates of perceived support at any single earlier point, and the instrument’s calibration should not be assumed to transfer to the acute phase. This is a recall-bias constraint in the strict sense: appraisals formed 39 to 40 months after the event may be reconstructed rather than retrieved, and reconstruction is mood-congruent, so survivors in current distress may recall support as having been lower than it was. Because such a bias would inflate rather than attenuate observed support–distress associations, the criterion-validity studies proposed above should, where possible, separate the measurement occasions for predictor and outcome.

These limitations point to a clear research agenda: cross-disaster and cross-cultural replication with formal invariance testing; longitudinal tracking across recovery phases; larger samples enabling income- and housing-stratified invariance; qualitative re-examination of IIS content; and head-to-head comparison with the MSPSS for incremental validity.

### 4.6. A Validation Roadmap for Prospective Users

The limitations set out above translate into an ordered sequence of studies that prospective users can follow. Two elements of that sequence—cross-cultural adaptation and longitudinal implementation—warrant specific guidance because neither is satisfied by translation or by repeated administration alone.

Cross-cultural adaptation. Adaptation should follow the ITC guidelines [43] and the procedure described by Beaton et al. [44]: independent forward translation; reconciliation; blind back-translation; expert-committee review of semantic, idiomatic, experiential, and conceptual equivalence; and cognitive pretesting with survivors in the target culture. Three points are specific to this instrument. First, the institutional block presumes a response architecture of state agencies and non-governmental organizations; where formal disaster response is delivered by different actors, or where the state is a party to the precipitating event, the referents in the institutional items must be re-specified at the expert-committee stage rather than translated literally. Second, the response window is anchored to a dated event, so the anchor must be replaced with the local event and its date, and the revised anchor must be retested in cognitive interviews. Third, structural equivalence should not be inferred from a successful translation: the adapted form requires its own dimensionality check (exploratory factor analysis or exploratory graph analysis) before confirmatory testing, followed by multi-group confirmatory factor analysis against a reference sample, with configural, metric, and scalar steps evaluated against the ΔCFI ≥ −0.010 and ΔRMSEA ≤ +0.015 criteria applied here [49]. Partial scalar invariance, with a minority of intercepts freed and reported item by item, is an acceptable outcome and still permits latent mean comparison; non-invariance of an entire block indicates that the block measures something different in the target culture and should be reported as such rather than absorbed into a total score. Given the observation-to-parameter constraint documented above, at least 300 respondents per cultural group is advisable.

Longitudinal implementation. Our position on whether the factor structure and measurement invariance should be re-evaluated at different stages of disaster recovery is that they should and that this is an empirical question the instrument is designed to raise rather than a formality. The mobilization–deterioration model [13,14] predicts that informal and formal support availability move in different directions across recovery phases; if they do, the meaning of an item referring to help received from neighbors is not guaranteed to be constant from the acute phase to long-term recovery. A three-wave design spanning the acute (0–3 months), intermediate (6–12 months), and long-term (24 months and beyond) phases would permit longitudinal measurement invariance testing—configural, weak, strong, and strict, with correlated residuals specified for repeated indicators—as a precondition for interpreting change [49]. Only once strong invariance is established can latent change score or second-order growth models test the model’s central prediction that family–relative and social environment support decline while institutional and instrumental–informational support track the trajectory of the formal response. Two design cautions follow from the present findings. The three-item family–relative block showed the weakest retest stability (ICC = 0.542) and is therefore the least suited to change detection in its current form, so longitudinal users should either lengthen it or interpret its change scores against the retest-based MDC_95_ of 2.015 rather than the alpha-based value of 0.689; for a block with an ICC of 0.542 the alpha-based figure substantially understates the change required to exceed measurement error (Supplementary Materials Tables S13.4 and S18.5). And because the present data were collected during long-term recovery only, the instrument’s calibration should be treated as established for that phase and provisional for earlier ones. Table 9 summarizes the full sequence, its priority, and the minimum design required at each step.

## 5. Conclusions

The PSSDS-28 provides promising initial psychometric evidence for an instrument that measures perceived social support after disasters while simultaneously representing support source and function. Its strengths include a replicable five-block structure established across factor-analytic, network, IRT, and method-variance frameworks; strong block-level internal consistency and composite reliability; measurement invariance across six groupings; theoretically coherent external validity evidence; and negligible common-method variance. Its limitations are set out in full in Section 4.5 and are not restated here. Understood as a contextually targeted complement to existing measurement—to be used in conjunction with established scales—the PSSDS-28 provides a foundation for research and assessment that takes seriously both who provides support after disaster and how that support operates. This foundation is initial: criterion-related validity, cross-disaster and cross-cultural replication, longitudinal tracking of support profiles across recovery phases, and head-to-head comparison with the MSPSS remain outstanding tasks that will ultimately determine whether the PSSDS-28’s theoretical advantage translates into an empirical and practical one.

## Figures and Tables

**Table 1:** Structural coverage of widely used perceived social support instruments against disaster-theoretic measurement requirements.

Instrument	IPS	C–N	INST	FD	DSF	VDS
MSPSS	✓	✗	✗	✗	✗	Partial †
ISEL	Imp	✗	✗	✓ ^a^	✗	✗
SSQ/SSQ6	Enum	✗	✗	✗	✗	✗
SPS	Imp	✗	✗	✓ ^b^	✗	✗
2-Way SSS	Imp	✗	✗	✓ ^c^	✗	✗
F-SozU K-3	Glo	✗	✗	Glo	✗	✗
PSSDS-28	✓	✓	✓	✓ ^d^	✓	✓

Note. IPS = interpersonal sources; C–N = community–neighborhood; INST = institutional (government, NGO); FD = functional differentiation; DSF = disaster-specific framing; VDS = validated in disaster survivors. ✓ = explicitly measured; ✗ = not measured; Imp = implicit (inferable from generic wording but not structurally differentiated); Enum = enumerated (respondents list supporters without structural differentiation); Glo = global (single undifferentiated appraisal). ^a^ Appraisal, tangible, belonging, self-esteem. ^b^ Six provisions. ^c^ Emotional, instrumental. ^d^ Emotional, instrumental–informational. † Psychometric properties examined in disaster samples, but the instrument was not designed, framed, or normed for the disaster context.

**Table 2:** Confirmatory factor analysis model fit comparison (validation subsample, *N* = 302; 28-Item Model B).

Model	χ^2^	df	CFI	TLI	RMSEA [90% CI]	SRMR	BIC
One-factor	3761.44	350	0.732	0.710	0.180 [0.175, 0.185]	0.194	26,070.76
Correlated five-factor	927.73	340	0.954	0.949	0.076 [0.070, 0.082]	0.072	24,327.80
Second-order	1339.83	345	0.922	0.914	0.098 [0.092, 0.103]	0.109	24,408.84
Bifactor	1302.65	322	0.923	0.909	0.101 [0.095, 0.106]	0.103	24,432.63

Note. All models estimated with WLSMV and theta parameterization. All chi-square values are significant at *p* < 0.001 (Supplementary Materials Table S11.1). Because nested-model chi-square difference tests under WLSMV require the DIFFTEST procedure, model comparison is based on descriptive fit indices and BIC rather than on chi-square differences; chi-square is reported for completeness and is not used as a comparative criterion, given its sensitivity to sample size and model complexity. The full four-model fit comparison is provided in Supplementary Materials Table S11.1. All four models were estimated on the identical 28-item set. Bifactor indices: ECV = 0.497, ω_h = 0.932.

**Table 3:** PSSDS-28 items, block assignments, and standardized CFA factor loadings (validation subsample, *N* = 302).

Block	Item	Item Content	λ
Family–Relative Support (FRS)	1	My family was there for me when I needed them.	0.925
	2	I relied on my family during difficult times.	0.963
	3	My family supported me throughout my long-term recovery.	0.868
Social Environment Support (SES)	7	My friends were there for me.	0.670
	8	My neighbors looked out for me.	0.637
	9	I found people around me from whom I could seek support.	0.783
	10	Our community acted in solidarity.	0.775
	11	There was mutual aid in our neighborhood/village.	0.789
	12	I connected with others who had experienced the disaster.	0.740
Institutional Support (IS)	13	Government institutions provided support.	0.846
	14	NGOs and aid organizations provided help.	0.792
	15	I was able to reach institutions when needed.	0.864
	16	The services institutions provided met my needs.	0.928
	17	I was able to access health services.	0.851
	18	Institutions acted in a trustworthy manner.	0.837
Emotional Support (ES)	19	I was able to share my feelings.	0.867
	20	There were people who listened to me.	0.902
	21	I found people who understood what I was going through.	0.907
	22	I encountered people who showed empathy.	0.849
	23	I felt that there were people beside me.	0.833
	24	I was helped to achieve emotional relief.	0.791
Instrumental–Informational Support (IIS)	25	My shelter needs were met.	0.761
	26	My basic needs such as food and clothing were met.	0.806
	27	My transportation needs were met.	0.760
	28	I was able to obtain financial support.	0.761
	29	I obtained information about where to apply.	0.847
	30	I was guided about the resources available.	0.845
	31	I was able to obtain legal counseling.	0.581

Note. λ = standardized WLSMV loading; all *p* < 0.001. Items 4, 5, and 6 were removed before confirmatory analysis based on calibration evidence.

**Table 4:** Reliability estimates for the PSSDS-28 blocks (full sample, *N* = 607; retest *n* = 89).

Block	*k*	Ordinal α [95% CI]	ω Total	CR	AVE	ICC [95% CI]
Family–Relative	3	0.949 [0.935, 0.962]	0.950	0.950	0.863	0.542 [0.225, 0.726]
Social Environment	6	0.852 [0.828, 0.875]	0.854	0.865	0.518	0.607 [0.316, 0.767]
Institutional	6	0.937 [0.926, 0.946]	0.938	0.940	0.725	0.859 [0.789, 0.907]
Emotional	6	0.939 [0.927, 0.948]	0.939	0.943	0.735	0.909 [0.865, 0.940]
Instrumental–Informational	7	0.900 [0.884, 0.913]	0.901	0.915	0.607	0.892 [0.840, 0.927]

Note. α and ω total computed from polychoric correlations (bootstrap *B* = 500). CR = composite reliability; AVE = average variance extracted. ICC = ICC(2,1), two-way random-effects, absolute agreement; retest *n* = 89, interval *M* ≈ 21 days. ICC benchmarks follow Koo and Li [50]: <0.50 = poor; 0.50–0.75 = moderate; 0.75–0.90 = good; >0.90 = excellent. The FRS ICC falls in the moderate range; longitudinal change interpretations for this block should be cross-referenced against the retest-based SEM and MDC_95_ values reported in Section 3.7.

**Table 5:** Sensitivity of the instrumental–informational support block to the exclusion of item 31 (full sample, *N* = 607; retest *n* = 87).

Indicator	IIS as Reported (7 Items)	IIS Without Item 31 (6 Items)	Change
Ordinal α [95% CI]	0.900 [0.884, 0.913]	0.893 [0.873, 0.908]	−0.007
ω total	0.901	0.893	−0.008
Mean inter-item correlation	0.561	0.581	+0.020
Corrected item–rest correlation (range)	0.605–0.761	0.632–0.745	—
Test–retest ICC(2,1)	0.892	0.871	−0.021
Retest SEM	0.255	0.293	+0.038
Retest MDC_95_	0.706	0.813	+0.107
HTMT with the institutional block	0.631	0.634	+0.003
Block mean (1–5)	2.99	3.08	+0.09
Block SD	1.06	1.08	+0.02

Note. SEM = standard error of measurement; MDC_95_ = minimal detectable change at the 95% confidence level; HTMT = heterotrait–monotrait ratio. Reliability and HTMT estimates are polychoric-based; test–retest estimates use ICC(2,1), two-way random effects, absolute agreement. Test–retest estimates were computed within the present run with listwise deletion on the paired block scores (*n* = 87); the test–retest analysis underlying Table 4 computes block scores as means over available responses (*n* = 89) and yields SEM = 0.251 and MDC_95_ = 0.696 for the seven-item block (Supplementary Materials Table S13.4). Both columns here are reported from the present run so that they rest on the same subsample. Apart from the two retest quantities, which are retest-based and therefore differ from the alpha-based measurement-error values in Section 3.7, the seven-item column reproduces the values reported in Table 4 and Section 3.7. IIS = instrumental–informational support.

**Table 6:** Construct validity: external correlations, discriminant indices, and HTMT ratios (full sample, *N* = 607).

Block	*r* with F-SozU K-3	*r* with UCLA-6	√AVE	Highest HTMT (Block Pair)
FRS	0.673 ***	−0.409 ***	0.929	0.581 (SES)
SES	0.515 ***	−0.517 ***	0.720	0.613 (ES)
IS	0.248 ***	−0.498 ***	0.851	0.631 (IIS)
ES	0.665 ***	−0.439 ***	0.858	0.613 (SES)
IIS	0.216 ***	−0.460 ***	0.779	0.631 (IS)

Note. External correlations are fully latent; all significant after FDR correction. *** *p*_fdr < 0.001. All HTMT values < 0.85.

**Table 7:** Ordinal measurement invariance results: nested Δ-fit comparisons (full sample, *N* = 607).

Grouping Variable	Comparison	ΔCFI	ΔRMSEA	ΔSRMR	Decision
Age group	Metric − Configural	+0.004	−0.005	+0.001	Supported
	Scalar − Metric	−0.004	+0.001	−0.001	Supported
Gender	Metric − Configural	+0.004	−0.005	+0.001	Supported
	Scalar − Metric	−0.005	+0.002	−0.001	Supported
Marital status	Metric − Configural	+0.003	−0.005	+0.002	Supported
	Scalar − Metric	−0.002	−0.001	−0.002	Supported
Education	Metric − Configural	+0.002	−0.004	+0.004	Supported
	Scalar − Metric	−0.003	−0.001	−0.004	Supported
Employment	Metric − Configural	+0.006	−0.007	+0.002	Supported
	Scalar − Metric	−0.005	+0.001	−0.002	Supported
Psychological support	Metric − Configural	+0.004	−0.005	+0.002	Supported
	Scalar − Metric	−0.003	+0.001	−0.002	Supported

Note. Metric step thresholds: ΔCFI ≥ −0.010, ΔRMSEA ≤ +0.015, ΔSRMR ≤ +0.030; scalar step: ΔCFI ≥ −0.010, ΔRMSEA ≤ +0.015, ΔSRMR ≤ +0.010. Income, loss type, and housing not testable (empty response categories in small subgroups).

**Table 8:** Research questions the hybrid PSSDS-28 architecture can and cannot address.

Question Type	Illustrative Research Question	Addressable with the PSSDS-28?	Requirement If Not Addressable
Channel standing	Do survivors perceive formal institutions as less supportive than their families?	Yes—compare IS and FRS block scores under established scalar invariance	—
Function standing	Is instrumental–informational provision the weakest support function during long-term recovery?	Yes—interpret the IIS block score against its alpha-based SEM and MDC_95_	—
Differential prediction	Does institutional support predict posttraumatic stress beyond interpersonal support?	Yes—enter the five blocks simultaneously as latent predictors	—
Group comparison	Do survivors in temporary housing report lower institutional support?	Partly—established for the six groupings with scalar invariance	Category-balanced samples for income, housing, and loss type
Change detection	Did a psychosocial program raise perceived emotional support?	Yes—compare observed change against the block’s retest-based SEM and MDC_95_	Longitudinal invariance testing (Section 4.6)
Source × function cell	Is instrumental support perceived as more available from kin than from formal agencies?	No	Supplementary crossed items, or a fully factorial instrument
Matching hypothesis	Does emotional support buffer bereavement only when it comes from kin?	No	Provider-specific functional scores derived from crossed items
Provider-specific evaluation	Which agency is perceived as the most responsive?	No—the IS block aggregates across formal providers	Provider-disaggregated items or an agency-specific module

Note. FRS = family–relative support; IS = institutional support; IIS = instrumental–informational support; SEM = standard error of measurement; MDC_95_ = minimal detectable change at the 95% confidence level. Alpha-based and retest-based block-level SEM and MDC_95_ values are both reported in Section 3.7.

**Table 9:** Validation roadmap for the PSSDS-28: priorities, minimum designs, and decision criteria.

Priority	Step	Minimum Design	Decision Criterion	Limitation Addressed
1	Independent structural replication	*n* ≥ 500 in an independent disaster sample; CFA of the correlated five-factor model	CFI ≥ 0.95; RMSEA ≤ 0.08; no Heywood cases	Observation-to-parameter ratio of approximately 2:1
1	Criterion-related validity	*n* ≈ 520; PSSDS-28 and MSPSS administered with clinical and functional criterion measures	Incremental ΔR^2^ over the MSPSS, following the directional pattern set out above	Absence of criterion-related evidence
2	Cross-cultural adaptation	ITC and Beaton procedure; *n* ≥ 300 per culture; multi-group CFA	Scalar, or documented partial scalar, invariance (ΔCFI ≥ −0.010)	Single-culture validation basis
2	Cross-disaster replication	Independent samples from flood, wildfire, epidemic, and conflict-displacement contexts; content review before administration	Recovery of the five-factor structure; item-level content validity in the new context	Single-event-type validation basis
3	Longitudinal calibration	Three waves (0–3, 6–12, and 24+ months); longitudinal invariance testing	Strong invariance established before any change modeling	Continuity–timing dynamics untested
3	Stratified invariance	Category-balanced sampling on income, housing, and loss type	Estimable configural model; scalar invariance	Invariance not testable for these variables
4	Response-set control	Administration with a social desirability screener; trial form containing reverse-keyed items	Block scores unchanged after partialling out socially desirable responding	Uniformly positive item keying
4	Item 31 revision	Cognitive interviews on a broadened administrative-navigation formulation	Higher loading and lower floor rate than the present item	Weakest item retained on content-validity grounds

Note. CFA = confirmatory factor analysis; ITC = International Test Commission; MSPSS = Multidimensional Scale of Perceived Social Support. Priority 1 denotes steps required before the PSSDS-28 is used beyond descriptive research; priority 4 denotes refinements of the instrument itself.

## Data Availability

The anonymized data, analysis scripts, codebooks, outputs, appendices, and study instruments are archived on OSF will be publicly accessible upon acceptance. https://osf.io/eh53j/overview?view_only=e0e48ce7962c45778c63f30fffcfd254 (accessed on 6 March 2026).

## Supplementary Materials

The following supporting information can be downloaded at https://www.mdpi.com/article/10.3390/healthcare14162573/s1. Section S1: Theoretical Framework and Construct Specification; Section S2: Item Development—Systematic Review and Initial Pool; Section S3: Content Validity—Two-Round Delphi and Cognitive Interviews; Section S4: Pilot Study; Section S5: Main Study Data Quality Control Protocol; Section S6: Main Sample Demographic and Background Characteristics; Section S7: Missing Data Analyses and Handling; Section S8: Cross-Validation Architecture and Subsample Equivalence; Section S9: Item-Level Descriptive Analyses; Section S10: Exploratory Analyses (Calibration Subsample); Section S11: Confirmatory Analyses (Validation Subsample); Section S12: Measurement Invariance; Section S13: Reliability Analyses; Section S14: Validity Evidence; Section S15: Item Response Theory Analyses; Section S16: Network Psychometrics; Section S17: Common Method Variance Assessment; Section S18: Additional Analyses; Section S19: Consolidated Robustness Summary; Section S20: PSSDS-28 User Guide and Scoring; Section S21: Mapping to Reporting and Measurement Standards.
