# Supplementary material for: Measuring Perceived Social Support After Disaster: Development and Validation of the Perceived Social Support in Disasters Scale (PSSDS-28)
Source: Healthcare (Basel). 2026 Aug 17;14(16):2573. doi: 10.3390/healthcare14162573 (PMC13512528; doi:10.3390/healthcare14162573)
Supplement: Supplementary file 1 [file healthcare-14-02573-s001.zip › healthcare-4447903-supplementary.pdf]

# Supplementary Material

## Measuring Perceived Social Support After Disaster: Development and Validation of the Perceived Social Support in Disasters Scale (PSSDS-28)

## Preface and Organization

This supplementary document provides complete methodological detail, extended statistical output, and all supporting tables and figures referenced in the main manuscript. Section numbering (S1–S19) parallels the manuscript's development and analysis sequence. The supplement is organized into five thematic parts:

- **Part I (S1–S4):** Construct specification, item development, and content validity
- **Part II (S5–S8):** Main study design, data quality, and cross-validation architecture
- **Part III (S9–S11):** Item-level analyses, exploratory analyses (calibration), and confirmatory analyses (validation)
- **Part IV (S12–S17):** Psychometric evaluation (measurement invariance, reliability, validity, IRT, network psychometrics, common method variance)
- **Part V (S18–S19):** Additional and sensitivity analyses
- **S20: PSSDS-28 user guide and scoring (item sets, scoring rules, percentile references)**
- **S21: Mapping to reporting and measurement standards (AERA–APA–NCME, JARS–Quant, TOP, STROBE, COSMIN)**

All item-level indicators were treated as ordinal and analyzed with polychoric-correlation–based methods unless otherwise noted. Analyses were conducted in R 4.5.2 using the following packages:

*lavaan* (v0.6.20), *semTools* (v0.5.7), *psych*, *EGAnet* (v2.3.0), *mirt* (v1.42), *mokken* (v3.1.1), *bootnet* (v1.6), *qgraph* (v1.9.8), *networktools* (v1.6.0), *mgm* (v1.2.15), *igraph* (v2.2.1), *naniar*, and *irr*. No imputation was performed at any stage. A fixed random seed (set.seed = 20240315) was used in all resampling procedures.

The Open Science Framework repository containing anonymized data, analysis scripts, codebooks, and supplementary materials is available at: [**https://osf.io/eh53j/overview?view_only=e0e48ce7962c45778c63f30fffcfd254**].

## PART I: CONSTRUCT SPECIFICATION, ITEM DEVELOPMENT, AND CONTENT VALIDITY

### S1. Theoretical Framework and Construct Specification

### S1.1 Conceptual Rationale

Perceived social support was conceptualized as a multidimensional construct integrating source-based and function-based facets of the support experience in the aftermath of a large-scale disaster. The conceptual architecture drew on several complementary theoretical traditions.

The *main-effect* and *stress-buffering* models (Cohen & Wills, 1985; Wheaton, 1985) provided the foundational mechanistic framework. Under the main-effect model, perceived support promotes well-being regardless of current stress level, operating through processes of belonging, identity affirmation, and positive affect. Under the buffering model, support attenuates the adverse impact of stressors specifically when stress is high—a condition prototypically met in post-disaster settings. Empirical evidence on the buffering hypothesis in disaster samples is not uniform (Norris et al., 2002), and there is evidence that temporal parameters of support moderate its protective function (Lam, 2024; Ma et al., 2025).

*Relational regulation theory* (Lakey & Orehek, 2011) contributed the insight that perceived support operates primarily through everyday interactions and shared activities—not exclusively through crisis-contingent assistance. This implies that an adequate measurement instrument must capture regulation-relevant content (e.g., feeling heard, being able to share emotions, experiencing relief) rather than only concrete help-giving.

*Optimal-matching theory* (Cutrona & Russell, 1990) established that the effectiveness of support depends on the fit between the type of support provided and the type of need experienced. Disasters create diverse simultaneous needs (shelter, food, medical care, financial, legal, informational, emotional), meaning that a measure limited to a single support type underestimates the full resource landscape.

The *post-disaster mobilization–deterioration model* (Norris & Kaniasty, 1996; Kaniasty & Norris, 2008) highlighted that support networks first expand immediately after disaster (mobilization) and then contract over time as network members experience fatigue, relocation, and resource depletion (deterioration). This temporal dynamic implies that measurement instruments should capture continuity and timing rather than merely cross-sectional availability.

*Community-resilience approaches (Leykin et al., 2013)* and research on institutional trust in disaster-affected populations (Thoresen et al., 2018; Yang & Bae, 2022) contributed the argument that disaster support extends beyond dyadic interpersonal channels to encompass neighborhood solidarity, collective efficacy, and institutional trust—channels that existing instruments (e.g., MSPSS; Zimet et al., 1988) systematically underrepresent.

The measurement gap motivating scale development was documented empirically through a systematic review: across 25 disaster-context scale studies retained in the *Scale set* (see S2.1), community/neighborhood and institutional support dimensions were represented by only 4–10 sources respectively, whereas family, peer, and significant-other support accounted for 20–22 sources each. By contrast, the *Theory set* documented strong mechanistic justification for both the community and institutional dimensions. This asymmetry—sparse measurement representation but strong theoretical justification—constituted the operationalized rationale for developing new dimension-specific items beyond what existing scales could offer.

### S1.2 Construct Definition

Perceived social support in disaster contexts was formally defined as: *a disaster survivor's subjective appraisal of the availability, accessibility, adequacy, and continuity of support received across interpersonal, communal, institutional, and functional channels following a disaster event.*

This definition deliberately integrates:

1. **Source diversity** (who provides support): family/relatives, peers/friends, community, institutions
2. **Functional diversity** (how support operates): emotional regulation, instrumental provision, informational guidance
3. **Temporal considerations** (when support is available): early-phase mobilization, sustained continuity over recovery
4. **Appraisal orientation** (subjective evaluation rather than objective receipt): consistent with the perceived-support vs. received-support distinction (Haber et al., 2007)

Relation to the definition used in the main text. The four facets listed above describe the conceptual space from which items were generated. The construct as operationalized in the final instrument is narrower, and the main text states it in that narrower form: the PSSDS-28 measures a survivor's retrospective appraisal of support experienced as having been available and provided since a specified disaster, and does not measure prospective availability—the belief that support would be forthcoming should a future need arise. The term availability in this section therefore refers to support appraised as having been accessible during the recovery period, not to anticipated future access. Continuity is likewise represented as an attribute embedded within source-block item wording rather than as a separate scored facet (see S2.3).

### S1.3 Initial Dimension Model

On the basis of the construct definition and the systematic review evidence, a seven-dimension preliminary model was specified deductively:

**Table S1.3. Preliminary seven-dimension model and theoretical anchors.**

| Dimension | Label | Theoretical Anchor |
|---|---|---|
| D1 | Family–Relative Support | Zimet et al. (1988); Kaniasty & Norris (2008) |
| D2 | Peer/Friend Support | Laksmita et al. (2020) |
| D3 | Special/Significant Person | Lakey & Orehek (2011) |
| D4 | Community/Neighborhood Support | Leykin et al. (2013) |
| D5 | Institutional Service Support | Thoresen et al. (2018); Yang & Bae (2022) |
| D6 | Continuity–Timing | Lam (2024); Ma et al. (2025) |
| D7 | Functional Type/Quality | Lakey & Orehek (2011); Cutrona & Russell (1990) |

This model was subsequently restructured by a three-member advisor panel into a five-factor hybrid model (see S2.3) that consolidated D1–D2 into Family–Relative and Social Environment source blocks, merged D3 into the source structure, absorbed D6 (timing) as an attribute embedded in source-block items, and split D7 into distinct Emotional and Instrumental–Informational function blocks.

## S2. Item Development: Systematic Review and Initial Pool

### S2.1 Systematic Review Protocol

A two-stream systematic search was conducted in Web of Science and Scopus in compliance with the PRISMA 2020 reporting framework (Page et al., 2021). Two complementary literature streams were defined:

- **Theory set:** Studies examining mechanisms, models, and theoretical frameworks of social support (buffering, main effect, optimal matching, relational regulation, convoy model, social identity, collective-level buffering, timing).
- **Scale set:** Studies examining social support measurement in disaster or mass-trauma contexts, with explicit psychometric content (scale development, adaptation, factor analysis, reliability, measurement invariance, IRT/Rasch).

**Search parameters:**

**Table S2.1a. Search parameters by stream (Theory set and Scale set).**

| Parameter | Theory Set | Scale Set |
|---|---|---|
| Databases | WoS + Scopus | WoS + Scopus |
| Language | English | English |
| Publication type | Article, Review | Article, Review |
| Date range | 1980–2025 | 2015–2025 |
| COVID/pandemic | Included (theoretical) | Excluded (Scopus) |

The WoS Theory Set search query targeted theoretical terms (e.g., "buffering hypothesis," "relational regulation theory," "optimal matching") intersected with social support terminology and filtered against scale/psychometrics content. The WoS Scale Set query intersected perceived social support terminology with disaster context terms and psychometric terms. The complete search strings are given below.

Search strings and search date. All four searches were executed on 20 December 2025; the record counts reported below reflect each database as indexed on that date. Queries were written in the native syntax of each database (Web of Science: TS/TI with NEAR/n proximity; Scopus: TITLE-ABS-KEY/TITLE with W/n proximity). Because TITLE-ABS-KEY and TS also search indexed keywords, which databases revise over time, re-execution at a later date yields a larger record set; the search date should therefore be treated as part of the search specification.

*Web of Science — Theory set (n = 204)*

```
TS=("social support" OR "perceived social support") AND TS=("buffering hypothesis" OR "stress-buffer*" OR ("stress" NEAR/3 buffer*) OR ("main effect" NEAR/2 model*) OR "optimal matching" OR ("support" NEAR/2 deterioration) OR ("support" NEAR/2 mobilization) OR "relational regulation theor*" OR ("social" NEAR/1 convoy) OR ("convoy" NEAR/2 model*)) AND TI=(theor* OR model* OR framework* OR hypothes* OR conceptual* OR "stress-buffer*" OR buffering) NOT TS=(scale* OR questionnaire* OR instrument* OR psychometr* OR validat* OR reliab* OR "factor analy*" OR EFA OR CFA OR "measurement invariance" OR IRT OR "item response theory" OR Delphi OR "cognitive interview*") AND DT=(Article OR Review) AND LA=(English)
```

The Theory-set publication window (1980–2025) was applied through the database interface rather than within the query string.

*Web of Science — Scale set (n = 155)*

```
TS=(("perceived social support" OR (perceived NEAR/3 "social support") OR "perceived support" OR "Multidimensional Scale of Perceived Social Support" OR MSPSS)) AND TS=(disaster* OR "post-disaster" OR earthquake* OR flood* OR wildfire* OR hurricane* OR typhoon* OR "mass trauma" OR "natural hazard*" OR "humanitarian crisis") AND TS=(scale* OR questionnaire* OR instrument* OR measure* OR psychometr* OR validat* OR reliab* OR "factor analy*" OR EFA OR CFA OR "measurement invariance" OR IRT OR "item response theory") AND PY=(2015-2025) AND DT=(Article OR Review) AND LA=(English)
```

*Scopus — Theory set (n = 97)*

```
SRCTYPE(j) AND (DOCTYPE(ar) OR DOCTYPE(re)) AND LANGUAGE(english) AND PUBYEAR > 1979 AND PUBYEAR < 2026 AND TITLE-ABS-KEY("social support" OR "perceived social support") AND TITLE-ABS-KEY("buffering hypothesis" OR "stress-buffer*" OR (stress W/3 buffer*) OR ("main effect" W/2 model*) OR "optimal matching" OR (support W/2 deterioration) OR (support W/2 mobilization) OR "relational regulation theor*" OR (social W/1 convoy) OR (convoy W/2 model*)) AND TITLE(theor* OR model* OR framework* OR hypothes* OR conceptual* OR "stress-buffer*" OR buffering) AND NOT TITLE-ABS-KEY(scale* OR questionnaire* OR instrument* OR psychometr* OR validat* OR reliab* OR "factor analy*" OR EFA OR CFA OR "measurement invariance" OR IRT OR "item response theory" OR Delphi OR "cognitive interview*")
```

*Scopus — Scale set (n = 33)*

```
SRCTYPE(j) AND (DOCTYPE(ar) OR DOCTYPE(re)) AND PUBYEAR > 2014 AND PUBYEAR < 2026 AND TITLE-ABS-KEY("perceived social support" OR (perceived W/3 "social support") OR "perceived support" OR "Multidimensional Scale of Perceived Social Support" OR MSPSS) AND TITLE-ABS-KEY(disaster* OR "post-disaster" OR earthquake* OR flood* OR wildfire* OR hurricane* OR typhoon* OR "mass trauma" OR "natural hazard*" OR "humanitarian crisis") AND TITLE-ABS-KEY(validat* OR psychometr* OR reliab* OR "factor analy*" OR EFA OR CFA OR "measurement invariance" OR IRT OR "item response theory" OR adapt* OR translat* OR "cross-cultural") AND NOT TITLE-ABS-KEY(covid* OR coronavirus* OR pandemic*)
```

The English-language limit for the Scopus Scale-set query was applied through the database interface.

Search yields and selection. Stage-by-stage counts are reported in Table S2.1 and exclusion reasons in Table S2.2.

**Table S2.1.** PRISMA 2020 stage counts for the two-stream review.

| Stage | Count | Theory set | Scale set |
|---|---|---|---|
| Records identified | 489 | 301 | 188 |
| Web of Science | 359 | 204 | 155 |
| Scopus | 130 | 97 | 33 |
| Duplicates removed | 78 | — | — |
| Records screened (title/abstract) | 411 | — | — |
| Records excluded at screening | 194 | — | — |
| Records eligible after screening | 217 | — | — |
| Prioritized core set (Core55) | 55 | 30 | 25 |
| extracted at full-text level (Level A) | 46 (83.6%) | — | — |
| extracted at abstract level (Level B) | 9 (16.4%) | — | — |

*Note.* Stream-level counts are given where the two streams were searched separately; deduplication and screening were performed on the combined pool, so stream-level counts are not defined for those stages.

**Table S2.2.** Exclusion reasons at title-and-abstract screening (n = 194).

| Exclude-reason code | n | % of exclusions |
|---|---|---|
| Not social-support focused | 89 | 45.9 |
| Not a disaster context | 42 | 21.6 |
| No theory, model, or framework content | 35 | 18.0 |
| Empirical only, no psychometric content | 24 | 12.4 |
| Not a scale or psychometric study | 4 | 2.1 |
| Total | 194 | 100.0 |

*Note.* Codes were prespecified and applied hierarchically within each stream; the full decision-rule set is given in OSF Attachment B. Percentages may not sum to 100 because of rounding.

Prioritization of the core set. Full-text extraction of all 217 eligible records was not feasible within the resources of the project. A prespecified relevance rubric was therefore applied to rank eligible records, weighting contributions of direct methodological relevance to scale development (illustrative weights: MSPSS-focused measurement +10; buffering or stress-buffering theory +8; measurement invariance +8; item response theory or Rasch modelling +7). The 55 highest-ranked records (Theory: 30; Scale: 25) formed the Core55 set used for extraction. This is a purposive prioritization rather than an exhaustive synthesis, and it is reported as such: the review was conducted to inform item generation, not to estimate a population effect.

Complete record set. A supplementary data file lists all 411 deduplicated records carried into screening, with database, stream, publication year, authors, title, source, DOI, screening decision, exclusion reason where applicable, and Core55 membership. It is provided as a supplementary data file (PRISMA_screened_records_411.csv) deposited in the OSF repository, and permits direct audit of the retrieved evidence base. Counts reconcile with the chain reported above: 411 screened, 217 included, 194 excluded, 55 prioritized for extraction.

Deduplication was conducted in R with a quality-scoring rule (DOI presence +4, abstract presence +2, year/source/author +1 each) to retain the most complete record for each duplicate group. Screening applied prespecified include/exclude rules (see OSF Attachment B for the full screening criteria and exclude-reason taxonomy). The Core55 was selected using a rubric weighting theoretical and psychometric relevance (e.g., buffering/stress-buffering theory +8; MSPSS-focused measurement +10; measurement invariance +8; IRT/Rasch +7).

Full-text retrieval was achieved for 46 of 55 Core55 studies (83.6%); 9 were coded at abstract level (Level B). Extraction used standardized templates separately for Theory and Scale studies (OSF Attachment D–E).

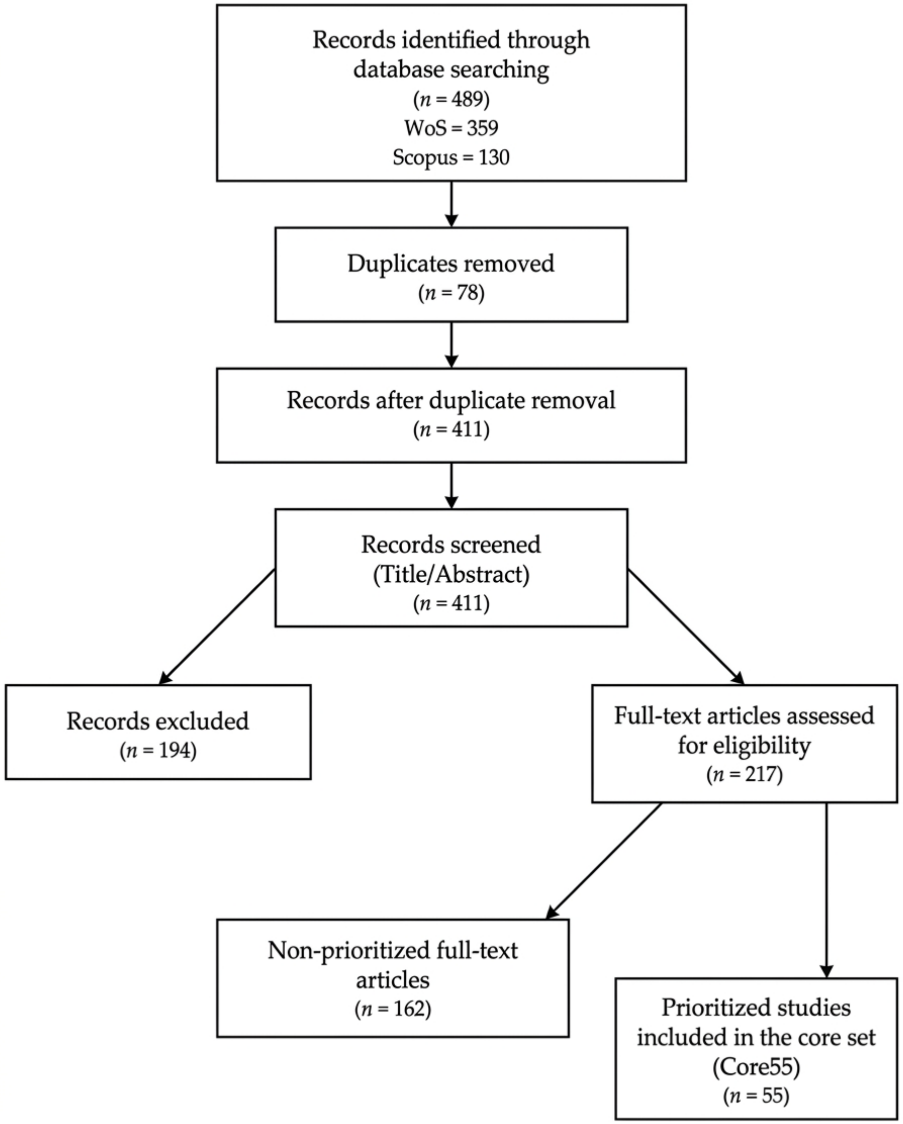


**Figure S2.1.** PRISMA 2020 flow diagram for the two-stream systematic review.

*Note.* Boxes show record counts at each screening stage. Identification, screening, eligibility, and inclusion phases are shown separately for the Theory set (left column) and Scale set (right column), then combined into the Core55 priority set. See OSF Attachments for full PRISMA counts.

### S2.2 Evidence Density by Dimension

Dimension evidence was coded as M (measured/operationalized) or R (rationale/gap-indicated) at the A (full-text) or B (abstract-only) level. The resulting evidence counts revealed a systematic asymmetry: family, peer, and significant-person dimensions were heavily represented in the Scale set, whereas community, institutional, and continuity–timing dimensions had sparse Scale-set representation but strong Theory-set justification. This asymmetry constituted an empirical index of the measurement gap motivating item development.

**Table S2.3.** Evidence density by dimension across the Core55 (Theory + Scale; Level A and B)

| Dimension | Scale A | Scale B | Theory A | Theory B | Total |
|---|---|---|---|---|---|
| Family–Relative (D1) | 22 | 3 | 3 | 1 | 29 |
| Peer/Friend (D2) | 22 | 3 | 7 | 2 | 34 |
| Special/Significant Person (D3) | 20 | 3 | 3 | 2 | 28 |
| Community/Neighborhood (D4) | 4 | 0 | 3 | 0 | 7 |
| Institutional Service (D5) | 10 | 0 | 3 | 1 | 14 |
| Continuity–Timing (D6) | 4 | 0 | 10 | 2 | 16 |
| Functional Type/Quality (D7) | 10 | 1 | 24 | 6 | 41 |

*Note.* M = source operationalizes/measures the dimension; R = source provides theoretical rationale or identifies a measurement gap for the dimension. A = full-text extraction; B = abstract-level extraction. The low Scale-set counts for D4–D6 relative to their Theory-set justification documents the measurement gap addressed by scale development. These counts reproduce OSF Attachment F, Table F1.

**Note.** The complete evidence documentation — screening criteria and the exclude-reason taxonomy (OSF Attachment B), the Core55 bibliography (OSF Attachment C), the scale and theory extraction records (OSF Attachments D and E), the dimension evidence counts (OSF Attachment F), the global dimension × evidence matrix (OSF Attachment G), the item–source tracing matrix (OSF Attachment H) and the dimension model synthesis notes (OSF Attachment J) — is deposited in the OSF repository.

### S2.3 Item Pool Development and Advisor Panel Restructuring

From the Core55 evidence, a 43-item preliminary pool was developed deductively across the seven-dimension model. Each item was linked to at least one Core55 source through an item-tracing matrix (OSF Attachment H), with linkage type (M or R) and evidence level (A or B) documented for full traceability.

A three-member advisor academic panel (expertise in scale development, psychometrics, and disaster psychology) evaluated the pool against item-writing standards (DeVellis & Thorpe, 2022), factor-analytic requirements (Brown, 2015), and theoretical coherence. The panel identified three structural risks:

1. **Source–function confounding:** Dimensions D1–D5 (source) and D7 (function type) co-existed at the same model level, risking high inter-factor correlations and discriminant validity threats in CFA.
2. **Item wording inconsistencies:** Inconsistent verb tense, redundant "after the disaster" phrases, and parenthetical examples across items.
3. **D3 (Special/Significant Person) heterogeneity:** The four items (religious leader, therapist, teacher/mentor, general significant other) represented four structurally distinct sub-sources with different institutional status, risking low internal consistency.

After deliberation, the panel restructured the pool into a five-factor hybrid model (source factors × function factors), reduced items to 28, removed all reverse-keyed items, standardized verb tense (past tense, completed action), and embedded timing content as an attribute within source-block items rather than as a separate factor. Three new Emotional Support items were added to reach the minimum viable block size.

**Table S2.4.** Seven-factor to five-factor restructuring: factor disposition decisions

| Original factor | Disposition | New assignment |
|---|---|---|
| D1 Family–Relative | Retained (3 core family items retained; 3 relative-focused items, asd4–asd6, monitored) | F1: Family–Relative Support |
| D2 Peer/Friend | Merged with D4 | F2: Social Environment Support |
| D3 Special/Significant Person | Dissolved; items redistributed or removed | Therapist → F3; Others removed |
| D4 Community/Neighborhood | Merged with D2 | F2: Social Environment Support |
| D5 Institutional Service | Retained; reverse-keyed item reformulated | F3: Institutional Support |
| D6 Continuity–Timing | Dissolved; timing embedded in source-block items | F1–F3 items |
| D7 Functional Type | Split into Emotional and Instrumental–Informational | F4 + F5 |

### S2.4 Disaster-Focused Assessment Instruments in Adjacent Domains

Table 1 in the main text compares the PSSDS-28 with instruments that measure perceived social support, because those are the instruments it would supplement. A separate and more recent literature has produced disaster-focused assessment instruments in adjacent domains. These are not competitors to the PSSDS-28 in the sense that Table 1 uses, but their existence raises a reasonable question about whether the measurement gap identified here has already been closed elsewhere. Table S2.5 evaluates five such instruments against three of the six criteria applied in main-text Table 1 — source structure, functional differentiation, and whether perceived support is the target construct — together with each instrument's target construct and level of measurement, which are the dimensions on which these instruments differ from the PSSDS-28.

**Table S2.5.** Disaster-focused instruments in adjacent domains, evaluated against the criteria used in main-text Table 1.

| Instrument | Target construct | Level | Source structure | Function differentiation | Measures perceived support? |
|---|---|---|---|---|---|
| CCRAM-28/10 | Community resilience (leadership, collective efficacy, preparedness, place attachment, social trust) | Community | ✗ | ✗ | ✗ — community capacity, not individual appraisal |
| WFRQ-9 | Family resilience in disaster scenarios | Family | ✗ | ✗ | ✗ — family functioning, not received or available support |
| HPE-Q | Household earthquake preparedness (7 factors, one labelled social support) | Household | Imp | ✗ | Partial — support treated as an antecedent of protective behaviour |
| brPSCS-10 | Personal social capital after flooding | Individual | Imp (bonding/bridging) | ✗ | ✗ — network resources, not appraised support |
| DIS-HL | Disaster health literacy (access, understand, appraise, apply) | Individual | ✗ | ✗ | ✗ — informational competence, not informational support received |
| PSSDS-28 | Perceived social support after disaster | Individual | ✓ (3 source blocks) | ✓ (2 function blocks) | ✓ |

*Note.* Criteria follow main-text Table 1: ✓ = explicitly measured; ✗ = not measured; Imp = implicit (inferable from wording but not structurally differentiated). CCRAM = Conjoint Community Resiliency Assessment Measure; WFRQ-9 = Walsh Family Resilience Questionnaire Short Version; HPE-Q = Household Preparedness for Earthquake Hazards Questionnaire; brPSCS-10 = Brazilian Personal Social Capital Scale 10; DIS-HL = Disaster Health Literacy instrument. Instruments were identified through a targeted supplementary search of PubMed (2016–2026) using disaster, psychometric, and psychosocial-assessment terms; this search was conducted to answer a specific question about adjacent instrument families and does not replace the systematic review reported in S2.1.

Two conclusions follow. First, none of these instruments measures perceived social support organized by source and by function, so none closes the gap that motivated the PSSDS-28: the community and family instruments operate at a collective level, the preparedness instrument treats support as an antecedent of behaviour, the social-capital instrument indexes network resources, and the health-literacy instrument addresses competence rather than received support. Second, and consistent with this, the Scale set of the systematic review reported in S2.1 (25 disaster-context measurement studies, 2015–2025) contained no newly developed instrument for perceived social support in disaster survivors. The disaster measurement literature in this domain consists predominantly of

adaptations of general-purpose instruments, most often the MSPSS. The measurement asymmetry documented in S2.2 is therefore not an artefact of the search strategy but a property of the field.

## S3. Content Validity: Two-Round Delphi and Cognitive Interviews

### S3.1 Expert Panel Composition

Eight experts participated in both Delphi rounds. Expertise areas included disaster psychology (n = 3), psychometrics and scale development (n = 1), statistics (n = 1), disaster management and emergency response (n = 2), and sociology (n = 1). All experts held doctoral degrees and had demonstrated peer-reviewed publication activity in their respective domains.

### S3.2 Delphi Procedure and Rating Criteria

Experts evaluated each item on three criteria rated on a 4-point Likert scale (1 = not relevant, 2 = somewhat relevant, 3 = quite relevant, 4 = highly relevant):

- **Relevance (Rel):** Does the item measure perceived social support after disaster?
- **Clarity (Cla):** Is the item language clear and unambiguous?
- **Representativeness (Rep):** Does the item represent the assigned dimension?

Categories 3 and 4 were coded as acceptable (binary). For n = 8 experts, the accepted I-CVI threshold was ≥ .78 (Lynn, 1986), corresponding to at least 7 of 8 experts rating the item acceptable. The S-CVI/Average (mean of item I-CVIs) was calculated at the item, factor, and scale level. Universal agreement (S-CVI/UA) was also calculated as the proportion of items with I-CVI = 1.00.

### S3.3 Round 1 Results (28-Item Form)

**Table S3.1.** Delphi Round 1: Item-level CVI results (n = 28 items; panel n = 8)

| No. | Block | Short label | Rel (n/8; I-CVI) | Cla (n/8; I-CVI) | Rep (n/8; I-CVI) | R1 Decision |
|---|---|---|---|---|---|---|
| 1 | FRS | Family present | 8/8 (1.00) | 8/8 (1.00) | 8/8 (1.00) | Retain |
| 2 | FRS | Family trust | 8/8 (1.00) | 8/8 (1.00) | 8/8 (1.00) | Retain |
| 3 | FRS | Family long-term | 8/8 (1.00) | 8/8 (1.00) | 8/8 (1.00) | Retain |
| 4 | FRS | Relative sharing | 8/8 (1.00) | 7/8 (0.88) | 6/8 (0.75) | Revise (relative focus) |
| 5 | FRS | Relative help | 8/8 (1.00) | 8/8 (1.00) | 8/8 (1.00) | Retain |
| 6* | FRS | Relative contact | — | — | — | Add (panel proposal) |
| 7 | SES | Friends present | 8/8 (1.00) | 8/8 (1.00) | 8/8 (1.00) | Retain |

| | | | | | | |
|---|---|---|---|---|---|---|
| 8 | SES | Neighbor care | 8/8 (1.00) | 7/8 (0.88) | 8/8 (1.00) | Retain |
| 9 | SES | Environment support | 8/8 (1.00) | 8/8 (1.00) | 7/8 (0.88) | Retain |
| 10 | SES | Community solidarity | 8/8 (1.00) | 8/8 (1.00) | 8/8 (1.00) | Retain |
| 11 | SES | Mutual aid | 8/8 (1.00) | 7/8 (0.88) | 8/8 (1.00) | Retain |
| 12 | SES | Peer connection | 7/8 (0.88) | 7/8 (0.88) | 7/8 (0.88) | Retain |
| 13 | IS | Government support | 8/8 (1.00) | 8/8 (1.00) | 8/8 (1.00) | Retain |
| 14 | IS | NGO support | 8/8 (1.00) | 8/8 (1.00) | 8/8 (1.00) | Retain |
| 15 | IS | Institution access | 8/8 (1.00) | 7/8 (0.88) | 8/8 (1.00) | Retain |
| 16 | IS | Service adequacy | 8/8 (1.00) | 7/8 (0.88) | 7/8 (0.88) | Retain |
| 17 | IS | Health access | 8/8 (1.00) | 8/8 (1.00) | 8/8 (1.00) | Retain |
| 18 | IS | Institutional trust | 8/8 (1.00) | 6/8 (0.75) | 7/8 (0.88) | Revise (clarify target) |
| 19 | ES | Emotion sharing | 8/8 (1.00) | 8/8 (1.00) | 8/8 (1.00) | Retain |
| 20 | ES | Being listened to | 8/8 (1.00) | 8/8 (1.00) | 8/8 (1.00) | Retain |
| 21 | ES | Being understood | 8/8 (1.00) | 8/8 (1.00) | 8/8 (1.00) | Retain |
| 22 | ES | Empathy | 8/8 (1.00) | 7/8 (0.88) | 8/8 (1.00) | Retain |
| 23 | ES | Not lonely | 8/8 (1.00) | 8/8 (1.00) | 7/8 (0.88) | Retain |
| 24 | ES | Emotional relief | 8/8 (1.00) | 7/8 (0.88) | 8/8 (1.00) | Retain |
| 25 | IIS | Shelter | 8/8 (1.00) | 8/8 (1.00) | 8/8 (1.00) | Retain |
| 26 | IIS | Basic needs | 8/8 (1.00) | 8/8 (1.00) | 8/8 (1.00) | Retain |
| 27* | IIS | Transportation | — | — | — | Add (panel proposal) |
| 28 | IIS | Finance | 8/8 (1.00) | 7/8 (0.88) | 8/8 (1.00) | Retain |
| 29 | IIS | Information | 8/8 (1.00) | 8/8 (1.00) | 7/8 (0.88) | Retain |
| 30 | IIS | Guidance | 8/8 (1.00) | 7/8 (0.88) | 8/8 (1.00) | Retain |
| 31* | IIS | Legal counseling | — | — | — | Add (panel proposal) |

*Note.* Items marked * were proposed for addition by the panel in Round 1 and therefore not rated in Round 1; they appear in Round 2. FRS = Family–Relative Support; SES = Social Environment Support; IS = Institutional Support; ES = Emotional Support; IIS = Instrumental–Informational Support. (R) = item revised in Round 1; (New) = item added in Round 1.

**Round 1 scale-level CVI summary (28-item form):**

- S-CVI/Ave (Relevance) = 27.875/28 = .996
- S-CVI/Ave (Clarity) = 26.500/28 = .946
- S-CVI/Ave (Representativeness) = 27.000/28 = .964
- S-CVI/UA (Relevance): 27/28 = .964; (Clarity): 17/28 = .607; (Rep): 21/28 = .750

**Post–Round 1 revisions and additions:**

Two items were revised based on panel feedback:

- **Item 4** (Relevance 1.00, Clarity 0.88, Representativeness 0.75 — below threshold): Changed from *"I shared my experiences with my family"* to *"I shared my experiences with my relatives"* to strengthen representativeness of the relative-specific content within the family–relative block.
- **Item 18** (Relevance 1.00, Clarity 0.75 — below threshold, Representativeness 0.88): Changed from *"Authorities/institutions acted in a trustworthy manner"* to *"Institutions acted in a trustworthy manner"* to resolve the dual-target ambiguity (individual authority vs. institutional structure).

Three items were added on panel recommendation:

- **Item 6** (*"I stayed in contact with my relatives"*): To balance the family–relative block (3 family + 3 relative items) and capture communication continuity.
- **Item 27** (*"My transportation needs were met"*): To fill a critical gap in the instrumental block (shelter, food, finance were covered; mobility was absent).
- **Item 31** (*"I was able to obtain legal counseling"*): To capture the specialized informational–legal support channel salient in Turkish post-earthquake context (property, insurance, temporary housing rights).

These changes yielded the 31-item candidate form for Round 2.

### S3.4 Round 2 Results (31-Item Form)

**Table S3.2.** Delphi Round 2: Item-level CVI results (n = 31 items; panel n = 8)

| No. | Block | Short label | Rel (n/8; I-CVI) | Cla (n/8; I-CVI) | Rep (n/8; I-CVI) | R2 Decision |
|---|---|---|---|---|---|---|
| 1 | FRS | Family present | 8/8 (1.00) | 8/8 (1.00) | 8/8 (1.00) | Accepted |
| 2 | FRS | Family trust | 8/8 (1.00) | 8/8 (1.00) | 8/8 (1.00) | Accepted |
| 3 | FRS | Family long-term | 8/8 (1.00) | 8/8 (1.00) | 8/8 (1.00) | Accepted |
| 4 | FRS | Relative sharing (R) | 8/8 (1.00) | 8/8 (1.00) | 8/8 (1.00) | Accepted ✓ |
| 5 | FRS | Relative help | 8/8 (1.00) | 8/8 (1.00) | 8/8 (1.00) | Accepted |
| 6 | FRS | Relative contact (New) | 8/8 (1.00) | 7/8 (0.88) | 8/8 (1.00) | Accepted ✓ |
| 7 | SES | Friends present | 8/8 (1.00) | 8/8 (1.00) | 8/8 (1.00) | Accepted |
| 8 | SES | Neighbor care | 8/8 (1.00) | 8/8 (1.00) | 8/8 (1.00) | Accepted |
| 9 | SES | Environment support | 8/8 (1.00) | 8/8 (1.00) | 8/8 (1.00) | Accepted |
| 10 | SES | Community solidarity | 8/8 (1.00) | 8/8 (1.00) | 8/8 (1.00) | Accepted |
| 11 | SES | Mutual aid | 8/8 (1.00) | 8/8 (1.00) | 8/8 (1.00) | Accepted |
| 12 | SES | Peer connection | 8/8 (1.00) | 7/8 (0.88) | 8/8 (1.00) | Accepted |
| 13 | IS | Government support | 8/8 (1.00) | 8/8 (1.00) | 8/8 (1.00) | Accepted |
| 14 | IS | NGO support | 8/8 (1.00) | 8/8 (1.00) | 8/8 (1.00) | Accepted |
| 15 | IS | Institution access | 8/8 (1.00) | 8/8 (1.00) | 8/8 (1.00) | Accepted |

| | | | | | | |
|---|---|---|---|---|---|---|
| 16 | IS | Service adequacy | 8/8 (1.00) | 7/8 (0.88) | 8/8 (1.00) | Accepted |
| 17 | IS | Health access | 8/8 (1.00) | 8/8 (1.00) | 8/8 (1.00) | Accepted |
| 18 | IS | Institutional trust (R) | 8/8 (1.00) | 8/8 (1.00) | 8/8 (1.00) | Accepted ✓ |
| 19 | ES | Emotion sharing | 8/8 (1.00) | 8/8 (1.00) | 8/8 (1.00) | Accepted |
| 20 | ES | Being listened to | 8/8 (1.00) | 8/8 (1.00) | 8/8 (1.00) | Accepted |
| 21 | ES | Being understood | 8/8 (1.00) | 8/8 (1.00) | 8/8 (1.00) | Accepted |
| 22 | ES | Empathy | 8/8 (1.00) | 8/8 (1.00) | 8/8 (1.00) | Accepted |
| 23 | ES | Not lonely | 8/8 (1.00) | 8/8 (1.00) | 7/8 (0.88) | Accepted |
| 24 | ES | Emotional relief | 8/8 (1.00) | 7/8 (0.88) | 8/8 (1.00) | Accepted |
| 25 | IIS | Shelter | 8/8 (1.00) | 8/8 (1.00) | 8/8 (1.00) | Accepted |
| 26 | IIS | Basic needs | 8/8 (1.00) | 8/8 (1.00) | 8/8 (1.00) | Accepted |
| 27 | IIS | Transportation (New) | 8/8 (1.00) | 8/8 (1.00) | 8/8 (1.00) | Accepted ✓ |
| 28 | IIS | Finance | 8/8 (1.00) | 8/8 (1.00) | 8/8 (1.00) | Accepted |
| 29 | IIS | Information | 8/8 (1.00) | 8/8 (1.00) | 8/8 (1.00) | Accepted |
| 30 | IIS | Guidance | 8/8 (1.00) | 7/8 (0.88) | 8/8 (1.00) | Accepted |
| 31 | IIS | Legal counseling (New) | 8/8 (1.00) | 8/8 (1.00) | 8/8 (1.00) | Accepted ✓ |

*Note.* (R) = revised in Round 1; (New) = added in Round 1 and rated for the first time in Round 2. ✓ = revision/addition successful (high consensus confirmed). All 31 items met I-CVI ≥ .78 on all three criteria. FRS = Family–Relative Support; SES = Social Environment Support; IS = Institutional Support; ES = Emotional Support; IIS = Instrumental–Informational Support.

**Round 2 scale-level CVI summary (31-item form):**

- S-CVI/Ave (Relevance) = 31.000/31 = **1.000**
- S-CVI/Ave (Clarity) = 30.400/31 = **.981**
- S-CVI/Ave (Representativeness) = 30.880/31 = **.996**
- S-CVI/UA (Relevance): 31/31 = **1.000**; (Clarity): 26/31 = **.839**; (Rep): 30/31 = **.968**

**Table S3.3.** Round 1 vs. Round 2 CVI comparison

| **Criterion** | **R1 S-CVI/Ave (k=28)** | **R2 S-CVI/Ave (k=31)** | **Δ** |
|---|---|---|---|
| Relevance | .996 | 1.000 | +.004 ↑ |
| Clarity | .946 | .981 | +.035 ↑ |
| Representativeness | .964 | .996 | +.032 ↑ |
| UA Relevance | .964 | 1.000 | +.036 ↑ |
| UA Clarity | .607 | .839 | +.232 ↑ |

| | | | |
|---|---|---|---|
| UA Representativeness | .750 | .968 | +.218 ↑ |

*Note.* Δ = Round 2 minus Round 1. All criteria improved from Round 1 to Round 2; the largest gains were in Universal Agreement for Clarity and Representativeness, confirming that targeted revisions and additions were effective. S-CVI/Ave = scale-level content validity index (average method); UA = universal agreement index.

**Table S3.4.** Factor-level S-CVI/Ave (Round 2, 31-item form)

| Block | k | Relevance | Clarity | Representativeness | Decision |
|---|---|---|---|---|---|
| Family–Relative Support | 6 | 1.000 | .980 | 1.000 | ✓ Accepted |
| Social Environment Support | 6 | 1.000 | .980 | 1.000 | ✓ Accepted |
| Institutional Support | 6 | 1.000 | .980 | 1.000 | ✓ Accepted |
| Emotional Support | 6 | 1.000 | .980 | .980 | ✓ Accepted |
| Instrumental–Informational Support | 7 | 1.000 | .983 | 1.000 | ✓ Accepted |
| **Overall** | **31** | **1.000** | **.981** | **.996** | **✓ Accepted** |

### S3.5 Cognitive Interview Findings

Twelve disaster survivors (6 women, 6 men; age range 22–67; education: primary through university; 9 provinces) participated in semi-structured cognitive interviews averaging 58 minutes (range 45–75 min). The think-aloud, paraphrasing, confidence-rating, recall-probing, and specific-probing techniques (Boateng et al., 2018) were applied to each item. Participants provided written informed consent; psychological first-aid referral information was provided at the start and end of each interview.

**Table S3.5.** Cognitive interview revisions: items requiring minor wording changes

| Item No. | Original wording | Revised wording | Revision type | Rationale |
|---|---|---|---|---|
| 6 | "I maintained regular contact with my relatives" | "I stayed in contact with my relatives" | Minor | "Regular" was abstract (5/12 participants uncertain about frequency threshold); revision preserves continuity meaning with greater flexibility |
| 12 | "I formed bonds with others who experienced the disaster" | "I communicated with others who experienced the disaster" | Minor | "Bonds" was interpreted variably (emotional vs. social network); behavioral formulation increases precision |

| | | | | |
|---|---|---|---|---|
| 16 | "The services provided by institutions were sufficient" | "The services provided by institutions met my needs" | Minor | "Sufficient" was evaluatively loaded and subject-relative (6/12 participants); person-centered formulation reduces implicit judgment |
| 23 | "I did not feel lonely" | "I felt that there were people beside me" | Minor | Double-negation confusion (4/12 participants); positive formulation eliminates reverse-coding comprehension burden |
| 27 | "Transportation support was provided" | "My transportation needs were met" | Minor | Ambiguous referent of "transportation support" (5/12 participants); need-centered formulation encompasses modes and providers |
| Instruction | "…in the period after the disaster…" | "…from the 6 February 2023 earthquakes through today…with emphasis on the past month" | Minor | Temporal reference was vague (6/12 participants); explicit date + 1-month anchoring standardizes recall window |

*Note.* 26 of 31 items required no revision (clear, answerable, and not emotionally distressing for all 12 participants). Emotional burden was noted for 3 participants on items referencing loss; the instruction page and debrief form were strengthened accordingly.

## S4. Pilot Study

### S4.1 Sample and Data Quality

The 31-item candidate form was piloted in N = 150 disaster survivors meeting the same eligibility criteria as the main study. After listwise deletion, the EFA subsample comprised n = 130. Item-level missingness ranged from 0.00% to 2.00%; straightlining was absent (0 cases); low-variance response patterns (item-level SD < .50) were detected in 2 cases (1.33%). No formal exclusion was applied in the pilot; these indicators were noted for the main-study exclusion protocol.

**Table S4.1.** Pilot study: Factor-level reliability (Cronbach's α and McDonald's ω)

| Block | k | Ordinal α (polychoric) | McDonald's ω (polychoric) |
|---|---|---|---|
| Family–Relative Support | 6 | .809 | .815 |
| Social Environment Support | 6 | .842 | .847 |
| Institutional Support | 6 | .811 | .811 |
| Emotional Support | 6 | .798 | .802 |
| Instrumental–Informational Support | 7 | .853 | .856 |

**Note.** All coefficients exceed the .70 acceptability threshold. Both α and ω were computed from the polychoric inter-item correlation matrix (ordinal approach), consistent with the main study reliability analyses (S13). ω coefficients were computed under a single-factor model assumption for each block. These values were recomputed from the raw pilot data for this supplement. The corresponding scale-level coefficient for the full 31-item pilot form is ordinal α = .935 (mean polychoric inter-item correlation = .318); the main-study equivalents are α = .944 and mean IIC = .352. Both scale-level coefficients are deposited in the OSF

repository (item-level analysis output). The full-scale coefficient differs from the block-level values above because it reflects all 31 items jointly rather than each block separately.

### S4.2 Pilot EFA Results

**Table S4.2.** Pilot EFA: KMO and Bartlett sphericity test (N = 130)

| Statistic | Value |
|---|---|
| KMO (overall) | .909 |
| Bartlett $\chi^2$ | 1940.72 |
| df | 465 |
| p | < .001 |

*Note.* KMO = .909 indicates excellent sampling adequacy. The Bartlett test confirms that the correlation matrix is significantly non-spherical, supporting factor analysis.

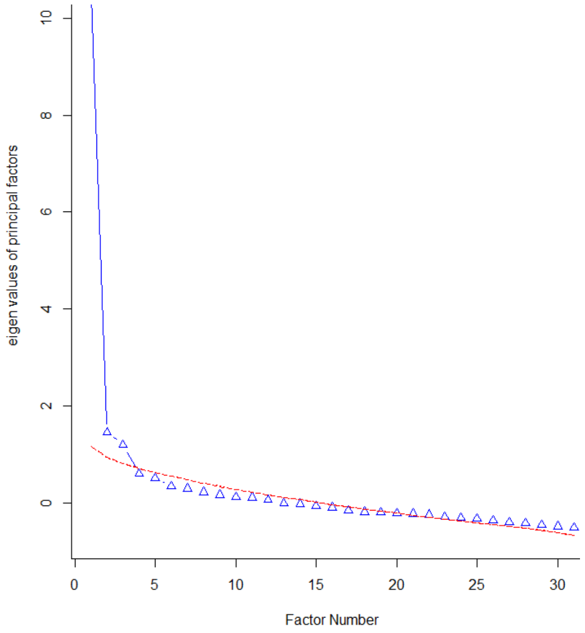


**Figure S4.1.** Parallel analysis scree plot (pilot study, N = 130, polychoric eigenvalues).

*Note.* Blue line = observed eigenvalues; red/grey line = simulated eigenvalues from parallel analysis. Intersection suggests 3 factors under parallel analysis; theoretical k = 5 was retained as the primary solution.

Parallel analysis suggested k = 3; the theoretically specified k = 5 solution was evaluated as the primary model. Under PAF extraction + Promax rotation in the five-factor solution, seven items were flagged as potential monitoring candidates: two for cross-loading alone (items 2 and 5), one for cross-loading with a borderline secondary loading (item 23), three for low primary loading combined with cross-loading (items 15, 17 and 19; item 19 additionally showed $h^2$ = .293), and one for low loading, cross-loading and low communality together (item 31). The issue codes are listed item by item in Table S4.3. These items were carried forward to the main study without revision because pilot N (130) fell below the recommended item-to-person ratio (1:5), making the instability likely attributable to small-sample estimation rather than structural problems.

**Table S4.3.** Pilot EFA: Items flagged for monitoring (PAF + Promax, k = 5, n = 130)

| Item code (pilot / main study) | Block | Item text (English) | Issue(s) | Pilot $h^2$ | Main study outcome |
|---|---|---|---|---|---|
| asd2 | FRS | Relied on family in difficult times | Cross-loading (PA3 = .705, PA1 = .518) | .596 | Retained — CITC = .545 in main study |
| asd5 | FRS | Relatives helped me | Cross-loading (PA5 = .497, PA1 = .409) | .461 | Retained — CITC = .579 |
| asd15 | IS | Was able to reach institutions when needed | Low loading + cross-loading (primary = .310) | .488 | Retained — CITC = .615 |
| asd17 | IS | Accessed health services | Low loading + cross-loading | .396 | Retained — CITC = .636 |
| asd19 | ES | Was able to share emotions | Low loading + cross-loading + $h^2$ = .293 | .293 | Retained — CITC = .670 |
| asd23 | ES | Felt that there were people beside me | Cross-loading | .506 | Retained — CITC = .638 |
| asd31 | IIS | Was able to obtain legal counseling | Low loading + cross-loading ($h^2$ = .333) | .333 | Monitor — CITC = .345; floor rate = 38.35% |

*Note.* CITC = corrected item–total correlation (polychoric) in the main study (N = 607). Item codes (asd2, asd5, etc.) correspond to the variable naming convention used throughout the main study dataset and all other supplement sections; the "Q" numbering used in the pilot data collection phase (Q1–Q31) maps directly onto asd1–asd31. Six of seven pilot-flagged items showed acceptable-to-strong performance in the main study; asd31 remained the only item satisfying multiple monitoring criteria across both studies. The pilot N/item ratio (130/31 ≈ 4.2) falls below the recommended 5:1, which accounts for the instability of pilot EFA solutions. FRS = Family–Relative Support; IS = Institutional Support; ES = Emotional Support; IIS = Instrumental–Informational Support.

**S4.3 Target Sample Size Rationale**

**Table S4.4.** A priori sample size estimation: multiple methods

| Method | Type | Criterion | Minimum N | Recommended N |
|---|---|---|---|---|
| N:p = 5:1 | Classical | Minimum ratio | 155 | 155 |
| N:p = 10:1 | Classical | Recommended ratio | 310 | 310 |
| RMSEA close-fit power analysis | Modern | Close-fit, power = .80 | 60 | 60 |
| semPower RMSEA power analysis | Modern | RMSEA = .05, power = .80 | 75 | 75 |
| Monte Carlo (ordinal + polychoric EFA) | Modern | Conv ≥ 95%, Tucker ≥ .95, Structure ≥ 80% | NA | NA |

*Note.* The Monte Carlo criterion was not met within the simulated range (N = 150–500) due to low convergence rates in polychoric + PAF + Promax; results are interpreted as showing improving recovery trends rather than a precise minimum. Pilot $h^2$ values: mean = .495, SD = .099, minimum = .293; these moderate communalities favored conservative planning. The final target was N = 800 (2 × 400 for EFA and CFA subsamples); the realized cleaned sample was N = 607. The pilot sample was planned separately and on a simpler basis: the classical minimum ratio of five respondents per item (31 items × 5 = 155) was used as the target, and the realized pilot sample (N = 150) approximates it. The pilot was intended for item screening and preliminary reliability estimation rather than for definitive dimensionality testing, so the more demanding criteria in the table above were reserved for the main study.

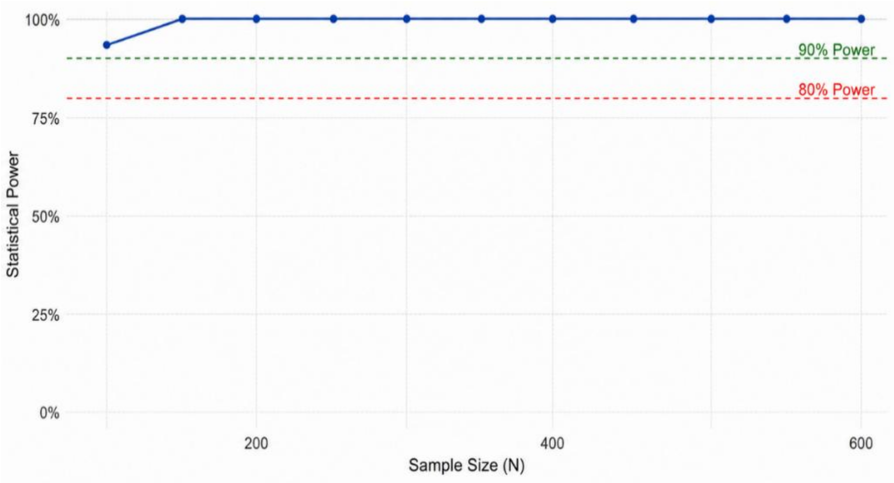


**Figure S4.2.** CFA power curve: statistical power as a function of sample size (RMSEA = .05, df = 424, α = .05).

*Note.* Power exceeds .99 for the realized validation subsample (n = 302). The curve was computed with a close-fit RMSEA power analysis (RMSEA = .05, df = 424, α = .05).

### S4.4 Project Timeline and Ethical Approval Coverage

All phases of the project involving human participants were conducted after institutional ethical approval was granted on 29 April 2026 by the Gümüşhane University Scientific Research and Publication Ethics Board (document number E-76551937-108.99-420974). The approval covered the expert review, cognitive interviewing, pilot, and main study phases as a single protocol. The systematic review and item generation preceded this period and did not involve human participants. Table S4.5 reports the dates of each phase.

**Table S4.5.** Project timeline for phases involving human participants.

| **Phase** | **Participants** | **Dates (2026)** | **Duration** |
|---|---|---|---|
| Ethical approval granted | — | 29 April | — |
| Delphi Round 1 | 8 experts | 1–3 May | 3 days |
| Delphi Round 2 | 8 experts | 5–7 May | 3 days |
| Cognitive interviews | 12 survivors | 10–14 May | 5 days |
| Pilot study | 150 survivors | 16–25 May | 10 days |
| Main study data collection | 672 respondents (607 retained) | 27 May – 16 June | 21 days |
| Test–retest administration | 89 volunteers | ≈ 21 days after individual T1 | — |

*Note.* Delphi rounds were conducted electronically, which permitted a short turnaround between rounds. Cognitive interviews and the pilot study were carried out by the trained field research team across the eight sampled provinces, operating in parallel across sites. Item-reduction decisions taken between the pilot and the main study were confined to the pilot exploratory factor analysis and block-level reliability estimates, which were computed on the pilot dataset as it closed; the substantive item revisions had already been completed at the cognitive interview stage. All main-study analyses reported in this work used data collected from 27 May 2026 onward, and no pilot participant was included in the main sample.

## PART II: MAIN STUDY DESIGN, DATA QUALITY, AND CROSS-VALIDATION ARCHITECTURE

### S5. Main Study Data Quality Control Protocol

#### S5.1 Variable Range and Format Checks

Eighty-six variables were verified against the study codebook (11 demographic/context variables, 3 attention-check items, 1 completion-time variable, 31 PSSDS candidate items [asd1–asd31], 3 F-SozU K-3 items [SD1–SD3], 6 UCLA-6 items [UCLA1–UCLA6], and 31 retest items [rt_asd1–rt_asd31]). No out-of-range integer codes, non-integer values, or missing expected columns were detected, except that one participant (ID 456) answered all six UCLA items with the value 5 (outside the valid 1–4 range; 6 out-of-range cells); those six cells were set to missing before further analysis. The minimum observed age was 18 years, satisfying the eligibility criterion.

**Table S5.1.** Variable range and format check summary

| Check | Result |
|---|---|
| Total variables checked (n) | 86 |
| Out-of-range: Age < 18 (n participants) | 0 |
| Out-of-range: UCLA > 4 (n participants) | 1 |
| Out-of-range: UCLA > 4 (n observations) | 6 |
| Non-integer values (n) | 0 |
| Missing expected columns (n) | 0 |

*Note.* The single UCLA out-of-range participant (ID 456) answered UCLA1–UCLA6 with a value of "5"; the valid range is 1–4. These cells were set to missing at this stage.

#### S5.2 Data Quality Exclusion Protocol

Four prespecified exclusion rules were applied to the raw dataset (N = 672). Rules were applied to the 40 primary ordinal items (PSSDS asd1–asd31, F-SozU K-3 SD1–SD3, UCLA-6 UCLA1–UCLA6) for the missingness criterion, and to the 31 PSSDS items only for the straightlining criterion (to avoid misclassifying genuine high- or low-support responders using scales with different response formats). The retest block (rt_asd1–rt_asd31) was excluded from all quality-control scans because its high structural missingness (~85.5%) reflected planned incomplete participation rather than data quality failure.

**Table S5.2.** Data quality screening and exclusions (raw dataset N = 672)

| Criterion | Rule | Threshold | Flagged n (%) | Passed n (%) |
|---|---|---|---|---|
| Attention checks | Failed ≥ 2 of 3 instructional checks (ACI1 = 1, ACI2 = 5, ACI3 = 3) | ≥ 2 fails | 7 (1.04) | 665 (98.96) |
| Missingness (40 ordinal items) | ≥ 10% missing across 40 primary ordinal items (asd1–asd31, SD1–SD3, UCLA1–UCLA6) | ≥ .10 | 3 (0.45) | 669 (99.55) |

| | | | | |
|---|---|---|---|---|
| Straightlining (31 PSSDS items only) | Max identical-response proportion ≥ .90 AND ≥ 25/31 asd items answered | ≥ .90 | 57 (8.48) | 615 (91.52) |
| Speeding | Completion time < mean/3 (cutoff = 3.99 min; mean = 11.97 min) | < 3.99 min | 2 (0.30) | 670 (99.70) |
| **Excluded (any rule)** | — | — | 65 (9.67) | — |
| **Retained** | — | — | — | 607 (90.33) |

*Note.* Four participants met two criteria simultaneously (IDs 304, 350, 382, 664); all remaining exclusions were attributable to a single rule. Straightlining was assessed on PSSDS items only to avoid misclassifying genuine extreme responders on external scales with different response formats. mean(TIME) = 11.97 min; speeding cutoff = 3.99 min. max_same_prop = maximum proportion of identical responses across asd1–asd31.

**Table S5.3.** Participants meeting multiple exclusion criteria

| ID | n rules failed | fail_ACI | fail_missing | fail_straightline | fail_time |
|---|---|---|---|---|---|
| 304 | 2 | TRUE | TRUE | FALSE | FALSE |
| 350 | 2 | TRUE | FALSE | TRUE | FALSE |
| 382 | 2 | TRUE | TRUE | FALSE | FALSE |
| 664 | 2 | TRUE | TRUE | FALSE | FALSE |

*Note.* All four participants who met multiple criteria failed the attention-check criterion (≥ 2 failed); participants 304, 382, and 664 additionally failed the missingness criterion; participant 350 additionally failed the straightlining criterion.

## S6. Main Sample Demographic and Background Characteristics

All demographic and context variables had complete data (0 missing cells) in the retained sample (N = 607).

**Table S6.1.** Main study sample: demographic and background characteristics (N = 607)

| Characteristic | n | % |
|---|---|---|
| **Age group** | | |
| 18–40 years | 432 | 71.2 |
| 41–60 years | 146 | 24.1 |
| 61+ years | 29 | 4.8 |
| **Gender** | | |
| Women | 274 | 45.1 |
| Men | 333 | 54.9 |
| **Marital status** | | |

| | | |
|---|---|---|
| Single | 290 | 47.8 |
| Married | 271 | 44.6 |
| Widowed | 33 | 5.4 |
| Divorced | 13 | 2.1 |
| **Education level** | | |
| Primary school or below | 89 | 14.7 |
| Middle school | 84 | 13.8 |
| High school | 201 | 33.1 |
| University | 222 | 36.6 |
| Postgraduate | 11 | 1.8 |
| **Employment status** | | |
| Employed | 231 | 38.1 |
| Unemployed | 61 | 10.0 |
| Retired | 32 | 5.3 |
| Student | 174 | 28.7 |
| Homemaker | 109 | 18.0 |
| **Monthly household income** | | |
| No regular income | 204 | 33.6 |
| Below minimum wage | 54 | 8.9 |
| Minimum wage | 127 | 20.9 |
| 2× minimum wage | 119 | 19.6 |
| 3× minimum wage | 62 | 10.2 |
| 4× minimum wage or above | 41 | 6.8 |
| **Province during earthquake** | | |
| Adana | 77 | 12.7 |
| Adıyaman | 79 | 13.0 |
| Diyarbakır | 67 | 11.0 |
| Gaziantep | 80 | 13.2 |
| Hatay | 86 | 14.2 |
| Kahramanmaraş | 75 | 12.4 |
| Malatya | 76 | 12.5 |
| Osmaniye | 67 | 11.0 |
| **Type of loss (most severe)** | | |
| Bereavement (close relative) | 139 | 22.9 |
| Health loss (injury/disability) | 9 | 1.5 |

| | | |
|---|---|---|
| Shelter loss (homelessness) | 76 | 12.5 |
| Livelihood loss | 25 | 4.1 |
| Property loss | 37 | 6.1 |
| Community loss (social network dissolution) | 46 | 7.6 |
| No major loss | 275 | 45.3 |
| **Current housing status** | | |
| Permanent housing | 351 | 57.8 |
| Temporary housing | 57 | 9.4 |
| With relatives | 25 | 4.1 |
| Rental | 147 | 24.2 |
| Other | 27 | 4.4 |
| **Professional psychological support** | | |
| Yes | 67 | 11.0 |
| No | 540 | 89.0 |

*Note.* Valid percentages are reported (no missing values for any variable). Columns may not sum exactly to 100% due to rounding. Minimum wage reference = 28,075 TRY (applicable at time of data collection).

**Continuous variables:**

**Table S6.2. Continuous demographic variables (full sample, N = 607).**

| Variable | N | Mean | Median | SD | Min | Max |
|---|---|---|---|---|---|---|
| Age (years) | 607 | 39.8 | 38 | 13.1 | 18 | 78 |
| Household size (persons) | 607 | 4.0 | 4 | 1.4 | 1 | 10 |

## S7. Missing Data Analyses and Handling

### S7.1 Scope and Definition

Missing data assessment focused on the primary analytic variables. The retest block (rt_asd1–rt_asd31) was excluded from the primary missing data assessment because its ~85.5% missingness was structural (only a volunteer subsample completed the retest). No imputation was applied at any stage.

**Table S7.1.** Missing data overview by variable set (retest block excluded)

| Variable set | N total | Variables | Overall missingness (%) | Complete cases (n) |
|---|---|---|---|---|
| 40-item primary ordinal set (asd1–asd31, SD1–SD3, UCLA1–UCLA6) | 607 | 40 | 0.482 | 507 (83.5%) |
| Demographic/context set | 607 | 11 | 0.000 | 607 (100%) |

*Note*. Retest items (rt_asd1–rt_asd31) excluded from this table; their high missingness (~85.5%) is structural. Overall missingness was computed as (total missing cells / total cells) × 100.

**Table S7.2.** Item-level missingness: 40-item primary ordinal set (sorted by number missing)

| Item | Missing n | Missing % | Block |
|---|---|---|---|
| asd30 | 8 | 1.318 | IIS |
| asd5 | 7 | 1.153 | FRS |
| UCLA2 | 7 | 1.153 | External |
| UCLA4 | 7 | 1.153 | External |
| UCLA5 | 6 | 0.988 | External |
| asd22 | 5 | 0.824 | ES |
| UCLA3 | 5 | 0.824 | External |
| UCLA6 | 5 | 0.824 | External |
| asd4 | 4 | 0.659 | FRS |
| asd23 | 4 | 0.659 | ES |
| asd25 | 4 | 0.659 | IIS |
| asd27 | 4 | 0.659 | IIS |
| SD2 | 4 | 0.659 | External |
| asd6 | 3 | 0.494 | FRS |
| asd9 | 3 | 0.494 | SES |
| asd11 | 3 | 0.494 | SES |
| asd14 | 3 | 0.494 | IS |
| asd17 | 3 | 0.494 | IS |
| asd24 | 3 | 0.494 | ES |
| asd28 | 3 | 0.494 | IIS |
| UCLA1 | 3 | 0.494 | External |
| asd8 | 2 | 0.329 | SES |
| asd13 | 2 | 0.329 | IS |
| asd18 | 2 | 0.329 | IS |

| | | | |
|---|---|---|---|
| asd20 | 2 | 0.329 | ES |
| asd29 | 2 | 0.329 | IIS |
| asd31 | 2 | 0.329 | IIS |
| SD1 | 2 | 0.329 | External |
| SD3 | 2 | 0.329 | External |
| asd1 | 1 | 0.165 | FRS |
| asd3 | 1 | 0.165 | FRS |
| asd10 | 1 | 0.165 | SES |
| asd16 | 1 | 0.165 | IS |
| asd19 | 1 | 0.165 | ES |
| asd21 | 1 | 0.165 | ES |
| asd26 | 1 | 0.165 | IIS |
| asd2 | 0 | 0.000 | FRS |
| asd7 | 0 | 0.000 | SES |
| asd12 | 0 | 0.000 | SES |
| asd15 | 0 | 0.000 | IS |

*Note.* Items asd2, asd7, asd12, and asd15 had no missing values. Maximum item-level missingness was 1.318% (asd30). FRS = Family–Relative Support; SES = Social Environment Support; IS = Institutional Support; ES = Emotional Support; IIS = Instrumental–Informational Support.

### S7.2 Missing Data Mechanism Diagnostics

**Table S7.3.** Little's MCAR test (40-item primary ordinal set)

| Test | $\chi^2$ | df | p | Conclusion |
|---|---|---|---|---|
| Little's MCAR | 1322.94 | 1933 | 1.00 | MCAR not rejected |

*Note.* p ≥ .05 indicates that the MCAR hypothesis is not rejected. This test is treated as a descriptive diagnostic, not a confirmatory proof of mechanism.

**Table S7.4.** Diagnostic logistic regression: missingness predicted by demographic variables

| Predictor | B | SE | z | p |
|---|---|---|---|---|
| Intercept | −1.729 | 0.719 | −2.406 | .016* |
| Age | 0.009 | 0.010 | 0.851 | .395 |
| Gender | 0.113 | 0.224 | 0.505 | .614 |
| Marital status | −0.110 | 0.206 | −0.536 | .592 |

| | | | | |
|---|---|---|---|---|
| Education | −0.065 | 0.119 | −0.544 | .587 |

*Note.* Outcome = any missing value across 40 primary ordinal items (1 = at least one missing, 0 = complete). No demographic predictor significantly predicted missingness (all p > .39). This analysis was treated as a pragmatic diagnostic screening. * p < .05.

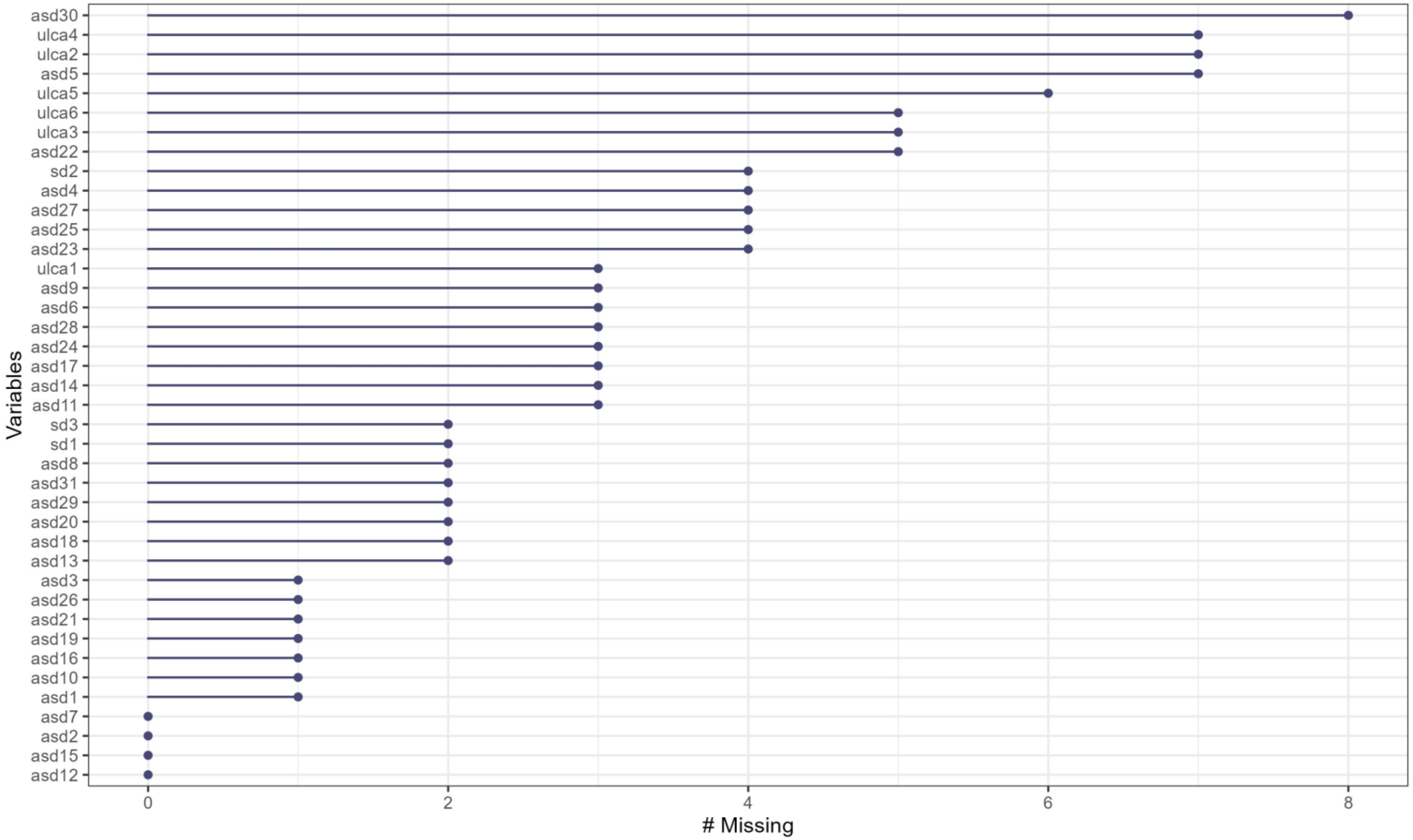


**Figure S7.1.** Item-level missingness rates across the 40 primary ordinal items.

*Note.* Bars show the number of missing responses per item in the cleaned dataset (N = 607). The highest missingness occurred for asd30 (n = 8, 1.318%). Most items had 0–3 missing responses.

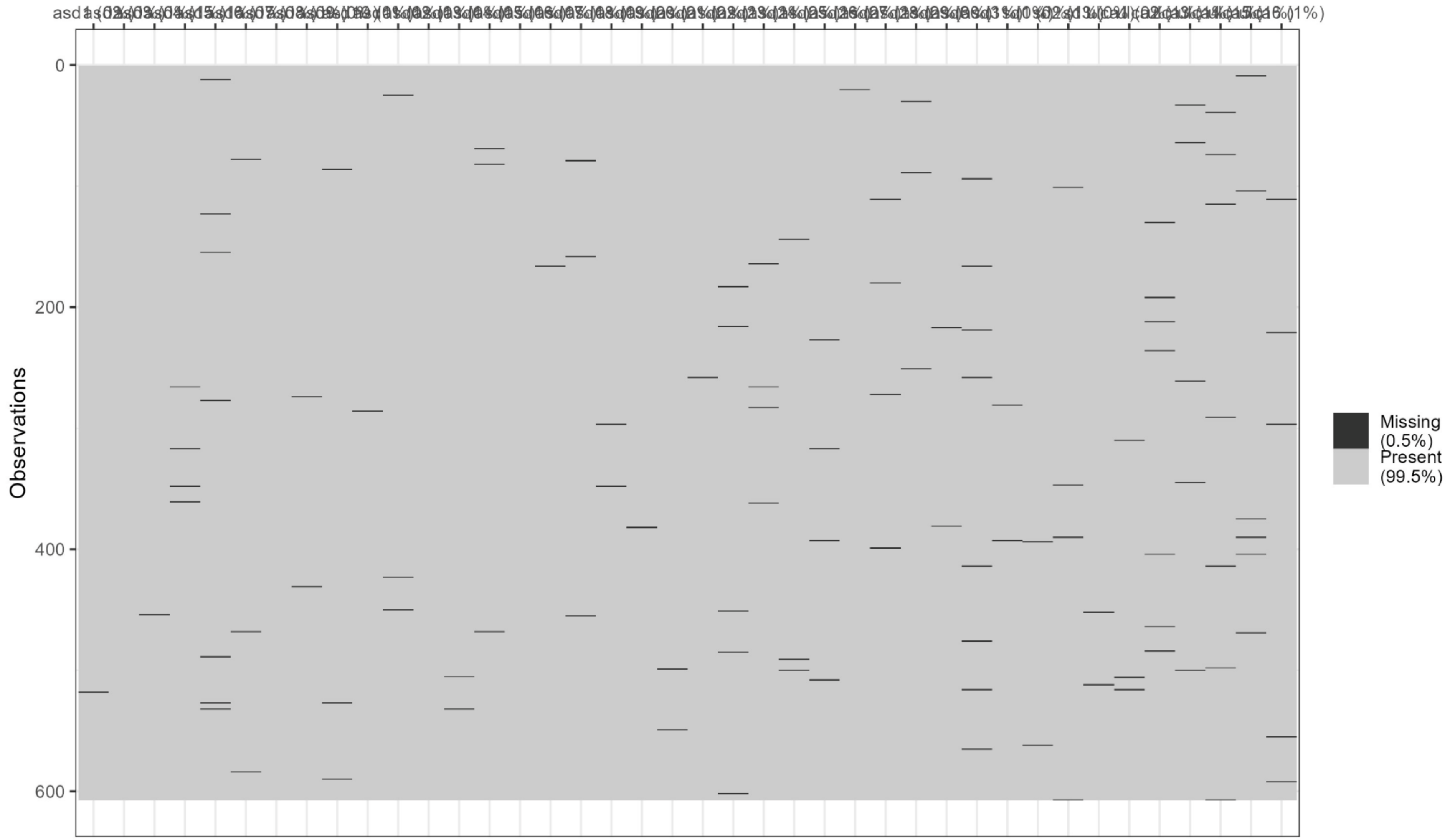


**Figure S7.2.** Missing data heatmap across the 40 primary ordinal items and 607 participants.

*Note.* Rows = participants (observations); columns = items. Grey = observed response; dark marks = missing. The sparse, non-systematic distribution of missing cells is consistent with MCAR.

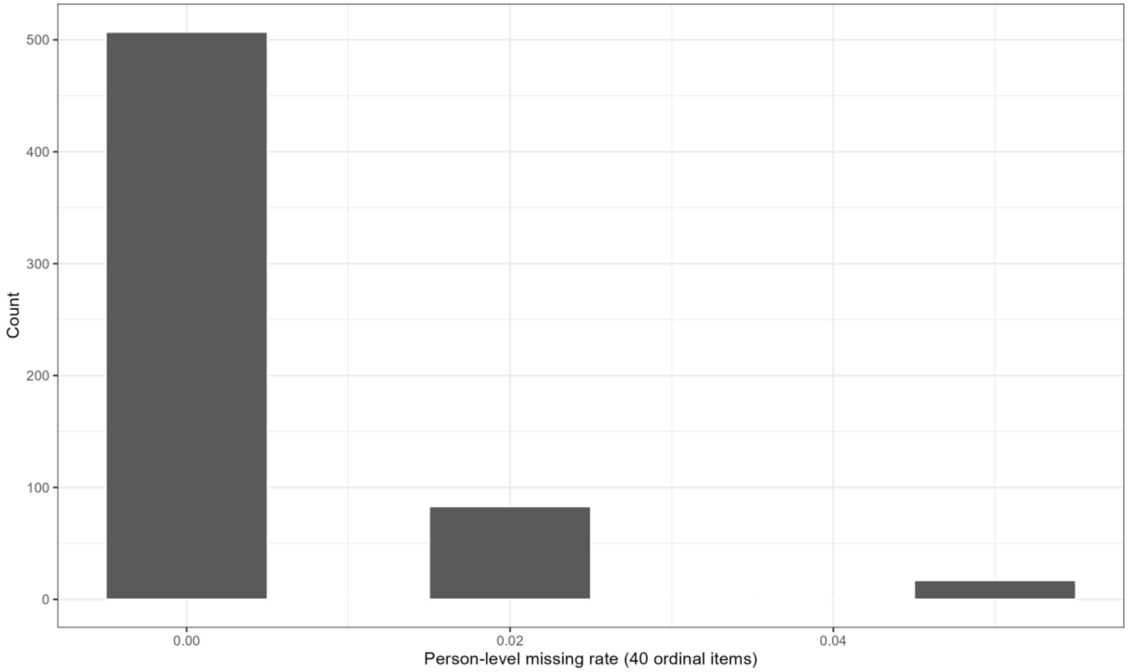


**Figure S7.3.** Person-level missingness rate distribution (40 primary ordinal items).

*Note.* Histogram of the proportion of missing responses per participant. The distribution is heavily concentrated at zero: the majority of participants (n ≈ 507) had no missing values; a small number had 1–2 missing items.

## S8. Cross-Validation Architecture and Subsample Equivalence

### S8.1 Stratified Split Design

To minimize overfitting and provide an independent test of the measurement model, the cleaned dataset (N = 607) was split into calibration (n = 305) and validation (n = 302) subsamples using stratified random allocation based on the gender × age group interaction. This ensured that gender and age distributions were approximately matched across subsamples.

**Table S8.1.** Stratified split cell counts by subsample (gender × age group)

| Stratum (Gender_AgeGroup) | Calibration n | Validation n | Total n |
|---|---|---|---|
| Men_18–40 | 125 | 124 | 249 |
| Women_18–40 | 92 | 91 | 183 |
| Women_41–60 | 38 | 38 | 76 |
| Men_41–60 | 35 | 35 | 70 |
| Women_61+ | 8 | 7 | 15 |
| Men_61+ | 7 | 7 | 14 |
| **Total** | **305** | **302** | **607** |

*Note.* Stratum labels use gender code (Women/Men) and age group. The close match in cell counts across subsamples confirms that stratified allocation achieved the intended balance.

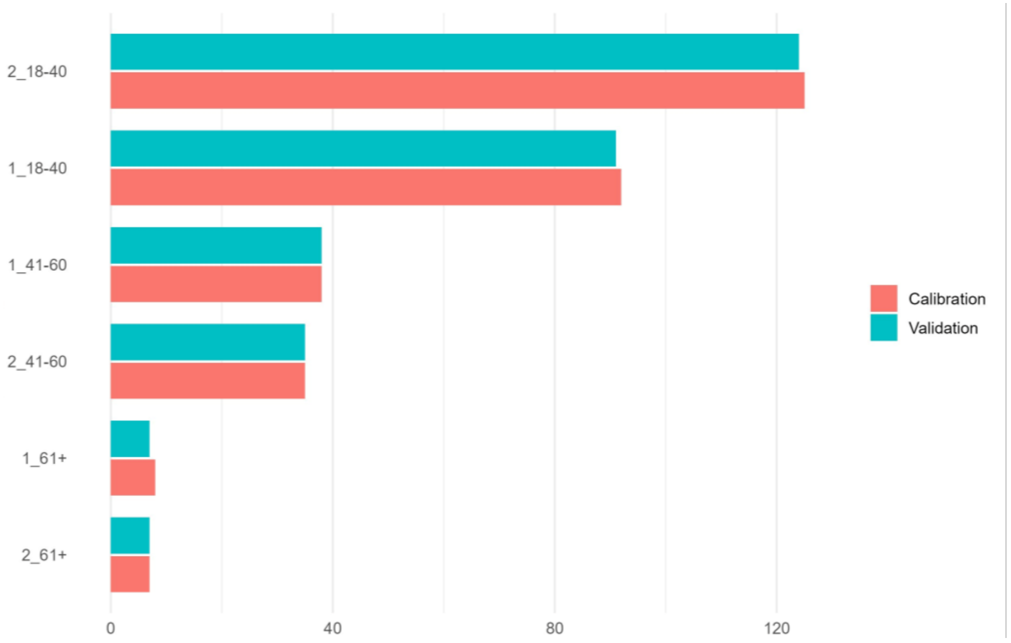


**Figure S8.1.** Stratified split balance: calibration vs. validation counts across gender × age group strata.

*Note.* Grouped bar chart showing calibration (dark) and validation (light) cell counts for each gender × age stratum. Near-equal bar heights across all strata confirm successful balance.

**S8.2 Subsample Equivalence Tests**

Prespecified equivalence tests were applied across 12 demographic and contextual variables using Bonferroni-corrected alpha (α_Bonf = .0042; k = 12). No comparison reached Bonferroni-corrected significance.

**Table S8.2.** Subsample equivalence tests: calibration vs. validation (N = 607)

| Variable | Test | Statistic | p | Effect size | Value |
|---|---|---|---|---|---|
| Loss type | Chi-square | $\chi^2(6) = 9.19$ | .163 | Cramér's V | .123 |
| Housing | Chi-square | $\chi^2(4) = 3.76$ | .439 | Cramér's V | .079 |
| PSSDS total score | Welch t | t(602.27) = 0.76 | .450 | Cohen's d | .061 |
| Marital status | Chi-square | $\chi^2(3) = 2.61$ | .456 | Cramér's V | .066 |
| Province | Chi-square | $\chi^2(7) = 6.02$ | .537 | Cramér's V | .100 |
| Monthly income | Wilcoxon | W = 45054.5 | .634 | Cliff's δ | −0.022 |
| Employment | Chi-square | $\chi^2(4) = 1.83$ | .767 | Cramér's V | .055 |
| Education | Wilcoxon | W = 46496.0 | .831 | Cliff's δ | +0.010 |
| Household size | Welch t | t(604.99) = 0.12 | .908 | Cohen's d | .009 |
| Psychological support | Chi-square | $\chi^2(1) = 0.01$ | .931 | Cramér's V | .004 |
| Gender | Chi-square | $\chi^2(1) = 0.00$ | .958 | Cramér's V | .002 |
| Age | Welch t | t(604.00) = −0.01 | .991 | Cohen's d | −.001 |

*Note.* Bonferroni-corrected significance threshold: α_Bonf = .0042 (k = 12). No comparison reached corrected significance. Effect sizes for the two Wilcoxon comparisons are reported as Cliff's δ, derived from the rank-sum statistic as $\delta = 2W/(n_1 n_2) - 1$ with $n_1 = 305$ and $n_2 = 302$; both are negligible ($|\delta| < .03$). For two independent groups Cliff's δ is algebraically identical to the rank-biserial correlation. PSSDS total score comparisons used the full 31-item applied form.

**Table S8.3.** PSSDS total score descriptives by subsample (applied 31-item form)

| Subsample | N | Mean | SD | Min | Max |
|---|---|---|---|---|---|
| Calibration | 305 | 110.04 | 22.32 | 36 | 150 |
| Validation | 302 | 108.63 | 23.64 | 41 | 151 |

*Note.* Values are for the 31-item applied form total score. The Welch t-test comparison yielded t(602.27) = 0.76, p = .450, Cohen's d = .061, confirming negligible mean difference.

## PART III: ITEM-LEVEL ANALYSES, EXPLORATORY ANALYSES, AND CONFIRMATORY ANALYSES

### S9. Item-Level Descriptive Analyses (PSSDS-31 administered pool; Full Sample, N = 607)

#### S9.1 Descriptive Statistics and Floor/Ceiling Effects

**Table S9.1.** Descriptive statistics and floor/ceiling rates for the 31 administered items (full sample, N = 607)

| Item code | Block | N | Mean | SD | Median | Skewness | Kurtosis | Floor % (1) | Ceiling % (5) |
|---|---|---|---|---|---|---|---|---|---|
| asd1 | FRS | 606 | 4.243 | 1.169 | 5 | −1.581 | 1.503 | 6.27 | 60.23 |
| asd2 | FRS | 607 | 4.198 | 1.179 | 5 | −1.418 | 0.946 | 5.44 | 58.48 |
| asd3 | FRS | 606 | 4.087 | 1.225 | 5 | −1.291 | 0.633 | 7.43 | 52.64 |
| asd4 | FRS | 603 | 3.798 | 1.295 | 4 | −0.886 | −0.330 | 9.45 | 39.30 |
| asd5 | FRS | 600 | 3.670 | 1.337 | 4 | −0.689 | −0.715 | 10.50 | 36.67 |
| asd6 | FRS | 604 | 3.825 | 1.268 | 4 | −0.853 | −0.333 | 8.11 | 40.89 |
| asd7 | SES | 607 | 3.748 | 1.296 | 4 | −0.815 | −0.431 | 9.72 | 37.40 |
| asd8 | SES | 605 | 3.565 | 1.320 | 4 | −0.585 | −0.800 | 10.74 | 31.07 |
| asd9 | SES | 604 | 3.838 | 1.218 | 4 | −0.904 | −0.129 | 7.12 | 37.75 |
| asd10 | SES | 606 | 3.842 | 1.215 | 4 | −0.850 | −0.229 | 6.60 | 39.11 |
| asd11 | SES | 604 | 3.768 | 1.272 | 4 | −0.758 | −0.504 | 7.95 | 38.74 |
| asd12 | SES | 607 | 3.847 | 1.259 | 4 | −0.963 | −0.095 | 8.73 | 39.70 |
| asd13 | IS | 605 | 3.443 | 1.376 | 4 | −0.473 | −1.006 | 13.72 | 29.09 |
| asd14 | IS | 604 | 3.392 | 1.326 | 4 | −0.429 | −0.916 | 13.08 | 25.33 |
| asd15 | IS | 607 | 3.137 | 1.369 | 3 | −0.178 | −1.156 | 17.46 | 20.43 |
| asd16 | IS | 606 | 3.152 | 1.374 | 3 | −0.243 | −1.141 | 18.48 | 19.80 |
| asd17 | IS | 604 | 3.316 | 1.409 | 4 | −0.354 | −1.152 | 16.39 | 26.49 |
| asd18 | IS | 605 | 3.321 | 1.433 | 4 | −0.370 | −1.161 | 17.85 | 27.77 |
| asd19 | ES | 606 | 3.650 | 1.377 | 4 | −0.713 | −0.727 | 12.87 | 37.13 |
| asd20 | ES | 605 | 3.805 | 1.215 | 4 | −0.952 | 0.063 | 8.60 | 34.71 |
| asd21 | ES | 606 | 3.802 | 1.239 | 4 | −0.908 | −0.148 | 8.25 | 36.14 |
| asd22 | ES | 602 | 3.847 | 1.165 | 4 | −0.930 | 0.087 | 6.31 | 35.22 |
| asd23 | ES | 603 | 3.871 | 1.153 | 4 | −0.975 | 0.235 | 6.30 | 35.66 |
| asd24 | ES | 604 | 3.690 | 1.213 | 4 | −0.695 | −0.370 | 7.78 | 31.79 |
| asd25 | IIS | 603 | 3.434 | 1.393 | 4 | −0.495 | −1.015 | 14.93 | 28.86 |
| asd26 | IIS | 606 | 3.343 | 1.362 | 4 | −0.396 | −1.038 | 14.69 | 24.75 |
| asd27 | IIS | 603 | 2.960 | 1.431 | 3 | −0.083 | −1.316 | 24.88 | 17.41 |

| | | | | | | | | | |
|---|---|---|---|---|---|---|---|---|---|
| asd28 | IIS | 604 | 2.772 | 1.407 | 3 | 0.123 | −1.295 | 27.32 | 14.07 |
| asd29 | IIS | 605 | 3.015 | 1.373 | 3 | −0.119 | −1.227 | 20.33 | 16.36 |
| asd30 | IIS | 599 | 2.935 | 1.379 | 3 | −0.090 | −1.261 | 23.20 | 14.19 |
| asd31 | IIS | 605 | 2.460 | 1.432 | 2 | 0.463 | −1.179 | 38.35 | 12.07 |

*Note.* Floor % = percentage of valid responses in category 1 ("strongly disagree"); Ceiling % = percentage of valid responses in category 5 ("strongly agree"). No item showed |Skewness| > 2 or |Kurtosis| > 7, indicating acceptable univariate distributional behavior. Item asd31 showed the most pronounced floor effect (38.35%), consistent with IRT and network analyses. Item-level screening preceded item reduction, so all 31 administered items are reported here; asd4, asd5 and asd6 were removed after the calibration-stage analyses described in S10.9 and do not form part of the PSSDS-28. FRS = Family–Relative Support; SES = Social Environment Support; IS = Institutional Support; ES = Emotional Support; IIS = Instrumental–Informational Support.

### S9.2 Corrected Item–Total Correlations and Internal Consistency Contribution

**Table S9.2.** Item-level psychometric indicators for the 31 administered items: corrected item–total correlation, alpha-if-deleted, and mean inter-item correlation (polychoric; full sample, N = 607)

| Item | Block | CITC (polychoric) | Cohen's d (27%) | Alpha if deleted | Mean IIC |
|---|---|---|---|---|---|
| asd1 | FRS | .518 | 1.309 | (no increase) | .318 |
| asd2 | FRS | .545 | 1.370 | (no increase) | .334 |
| asd3 | FRS | .537 | 1.414 | (no increase) | .329 |
| asd4 | FRS | .508 | 1.298 | (no increase) | .312 |
| asd5 | FRS | .579 | 1.646 | (no increase) | .354 |
| asd6 | FRS | .572 | 1.455 | (no increase) | .350 |
| asd7 | SES | .561 | 1.430 | (no increase) | .343 |
| asd8 | SES | .528 | 1.360 | (no increase) | .323 |
| asd9 | SES | .620 | 1.635 | (no increase) | .378 |
| asd10 | SES | .586 | 1.553 | (no increase) | .358 |
| asd11 | SES | .574 | 1.521 | (no increase) | .351 |
| asd12 | SES | .569 | 1.561 | (no increase) | .348 |
| asd13 | IS | .619 | 2.241 | (no increase) | .378 |
| asd14 | IS | .564 | 1.784 | (no increase) | .345 |
| asd15 | IS | .615 | 2.016 | (no increase) | .375 |
| asd16 | IS | .672 | 2.173 | (no increase) | .409 |
| asd17 | IS | .636 | 2.119 | (no increase) | .388 |
| asd18 | IS | .606 | 2.044 | (no increase) | .370 |
| asd19 | ES | .670 | 1.868 | (no increase) | .408 |

| | | | | | |
|---|---|---|---|---|---|
| asd20 | ES | .636 | 1.646 | (no increase) | .388 |
| asd21 | ES | .665 | 1.748 | (no increase) | .405 |
| asd22 | ES | .629 | 1.556 | (no increase) | .383 |
| asd23 | ES | .638 | 1.546 | (no increase) | .388 |
| asd24 | ES | .642 | 1.650 | (no increase) | .391 |
| asd25 | IIS | .537 | 1.678 | (no increase) | .329 |
| asd26 | IIS | .558 | 1.920 | (no increase) | .342 |
| asd27 | IIS | .545 | 2.045 | (no increase) | .334 |
| asd28 | IIS | .470 | 1.483 | (no increase) | .289 |
| asd29 | IIS | .557 | 1.978 | (no increase) | .341 |
| asd30 | IIS | .559 | 2.000 | (no increase) | .342 |
| asd31 | IIS | .345 | 1.177 | +.0005 (increase) | .214 |

*Note.* CITC = corrected item–total correlation (polychoric, computed from the smoothed polychoric correlation matrix). Cohen's d = upper vs. lower 27% group discrimination index. "Alpha if deleted" = whether removing the item would increase the ordinal alpha of the 31-item administered pool (α = .944; complete-case N = 540), not that of the item's own block; all items are coded as "(no increase)" except asd31, which showed a negligible increase of +.0005 upon deletion. Block-level alpha-if-deleted values, computed on the 28-item form, are reported separately in Table S13.2. Mean IIC = mean polychoric inter-item correlation between the item and the other 30 items of the administered pool. The shaded row (asd31) indicates the only item with a positive pool-level alpha-if-deleted value. CITC is likewise computed against the remaining 30 items of the administered pool. FRS = Family–Relative Support; SES = Social Environment Support; IS = Institutional Support; ES = Emotional Support; IIS = Instrumental–Informational Support.

**S10. Exploratory Analyses — Calibration Subsample (n = 305)**

Input data: calibration_sample.xlsx (n = 305, asd1–asd31). This section comprises two parallel analysis lines: Part A (S10.1–S10.9) covers the exploratory analyses of the administered 31-item pool, Part B (S10.10–S10.18) covers the 28-item exploratory re-analyses following the item reduction decision, and Part C (S10.19) compares the two solutions. The polychoric correlation matrix was computed using pairwise complete observations and smoothed; EFA and EGA model estimations were conducted using complete-case observations. The primary number of factors/dimensions was fixed at k = 5 based on the theoretical model; statistical retention criteria were used for diagnostic purposes only. MINRES (primary) and ULS (sensitivity) were used for EFA extraction, while oblimin (primary) and promax (sensitivity) were applied for rotation. For EGA, GLASSO (EBIC regularization), cor_auto correlations, and the Walktrap (primary) and Leiden (sensitivity) algorithms for community detection were applied. Bootstrap stability analyses with B = 200 replications were performed for both methods.

**PART A: 31-Item Pool Analyses (asd1–asd31)**

**S10.1 Missing Data and Analysis N (31-Item Pool)**

Missingness in the calibration subsample (n = 305) was negligible across the 31 PSSDS items. Of 305 participants, 273 (89.5%) had complete data on all 31 items; 25 (8.2%) had one missing item; and 7 (2.3%) had two missing items. Item-level missingness ranged from 0% (asd1, asd2, asd7, asd8, asd12, asd15, asd16, asd19, asd26, asd28, and others) to 1.31% (asd5, asd30). EFA and EGA model estimates were based on complete-case observations (N = 273).

**Table S10.1.** Item-level missingness in the calibration subsample (31-item pool)

| Item | Missing n | Missing % |
|---|---|---|
| asd1 | 0 | 0.00 |
| asd2 | 0 | 0.00 |
| asd3 | 1 | 0.33 |
| asd4 | 3 | 0.98 |
| asd5 | 4 | 1.31 |
| asd6 | 1 | 0.33 |
| asd7 | 0 | 0.00 |
| asd8 | 0 | 0.00 |
| asd9 | 2 | 0.66 |
| asd10 | 1 | 0.33 |
| asd11 | 3 | 0.98 |
| asd12 | 0 | 0.00 |
| asd13 | 2 | 0.66 |
| asd14 | 1 | 0.33 |
| asd15 | 0 | 0.00 |
| asd16 | 0 | 0.00 |
| asd17 | 1 | 0.33 |
| asd18 | 1 | 0.33 |
| asd19 | 0 | 0.00 |
| asd20 | 1 | 0.33 |
| asd21 | 1 | 0.33 |
| asd22 | 3 | 0.98 |
| asd23 | 3 | 0.98 |
| asd24 | 1 | 0.33 |
| asd25 | 3 | 0.98 |

| | | |
|---|---|---|
| asd26 | 0 | 0.00 |
| asd27 | 1 | 0.33 |
| asd28 | 0 | 0.00 |
| asd29 | 1 | 0.33 |
| asd30 | 4 | 1.31 |
| asd31 | 1 | 0.33 |

*Note.* Missingness percentages computed over n = 305. All items had observed responses in the full 1–5 range. EFA and EGA model estimates used complete-case observations (N = 273).

### S10.2 EFA: Factorability and Sampling Adequacy (31-Item Pool)

**Table S10.2.** EFA sampling adequacy (calibration subsample, polychoric, 31-item pool)

| Statistic | Value |
|---|---|
| N (pairwise, for polychoric) | 305 |
| N (complete-case, for model estimation) | 273 |
| Number of items (p) | 31 |
| KMO (overall) | .816 |
| KMO (minimum item MSA) | .661 (asd28) |
| KMO (maximum item MSA) | .931 (asd12) |
| Bartlett $\chi^2$ | 8748.41 |
| Bartlett df | 465 |
| Bartlett p | < .001 |

*Note.* No item fell below the .50 MSA unacceptability threshold. KMO = Kaiser-Meyer-Olkin; MSA = measure of sampling adequacy.

### S10.3 Factor/Dimension Retention Evidence (31-Item Pool, Diagnostic)

Five statistical retention methods were applied. Parallel analysis, Velicer's MAP, and Kaiser's criterion (eigenvalue > 1) suggested k = 7; the automatic scree elbow supported k = 5, matching the theoretical model. The Hull method was unavailable due to a software constraint. These indicators were used diagnostically; the theoretical k = 5 was retained as the primary solution.

**Table S10.3.** Factor retention methods comparison (31-item pool, calibration subsample)

| Method | Suggested k | Note |
|---|---|---|
| Parallel Analysis (PA) | 7 | — |
| Velicer's MAP | 7 | Minimum MAP = .0232 at k = 7; k = 5 vs. k = 7 Δ = .0052 |
| Scree (automatic elbow) | 5 | Matches theoretical k |
| Kaiser criterion (eigenvalue > 1) | 7 | — |
| Hull method | NA | EFAtools::N_FACTORS unavailable |

**Table S10.4.** Velicer MAP values across candidate k (31-item pool)

| k | MAP |
|---|---|
| 1 | .0716 |
| 2 | .0510 |
| 3 | .0345 |
| 4 | .0298 |
| 5 | .0284 |
| 6 | .0267 |
| 7 | .0232 |
| 8 | .0241 |

*Note.* MAP reaches its minimum at k = 7 (.0232); the difference between k = 5 and k = 7 is small (Δ = .0052).

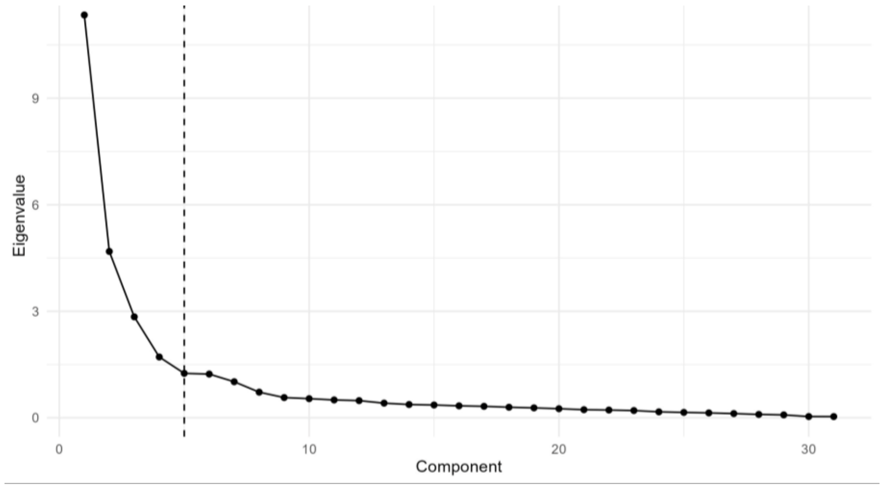


**Figure S10.1.** Scree plot (polychoric eigenvalues; 31-item pool; calibration subsample).

*Note.* Dashed line indicates the automatic elbow point (k = 5), which matches the theoretical k.

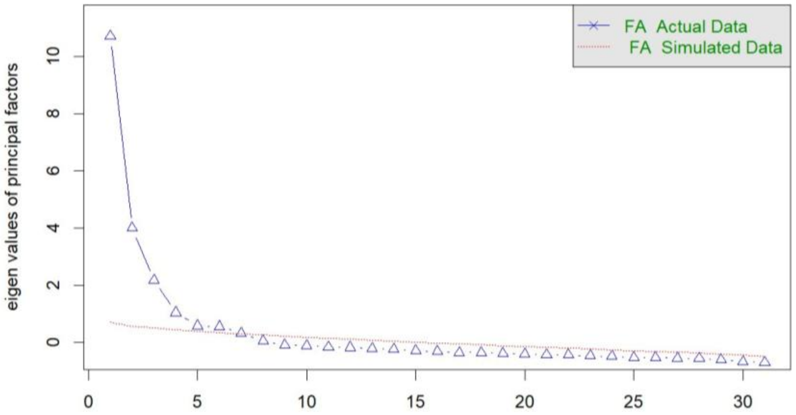


**Figure S10.2.** Parallel analysis (polychoric; 31-item pool; calibration subsample).

*Note.* Observed eigenvalues (blue) clearly exceed simulated eigenvalues (red) through the first five components; separation diminishes markedly from the sixth component onward.

### S10.4 EFA Model Fit: k = 1–8 Comparison and Primary k = 5 Solution (31-Item Pool)

**Table S10.5.** EFA model fit comparison across candidate k values (polychoric, MINRES; calibration subsample; 31-item pool)

| k | Rotation | RMSR | RMSEA | RMSEA [90% CI] | TLI | BIC |
|---|---|---|---|---|---|---|
| 1 | none | .170 | .199 | [.194, .204] | .322 | 3177.02 |
| 4 | oblimin | .051 | .137 | [.132, .143] | .675 | 352.87 |
| 5 | oblimin | .043 | .126 | [.120, .131] | .726 | 31.32 |
| 6 | oblimin | .033 | .118 | [.112, .124] | .760 | −146.42 |
| 7 | oblimin | .025 | .111 | [.105, .117] | .786 | −259.39 |
| 8 | oblimin | .021 | .109 | [.102, .115] | .794 | −275.67 |

*Note.* The k = 5 solution was retained as primary on theoretical grounds. Known inflation properties of these indices in polychoric EFA with large item counts and oblique factors account for the failure to meet conventional thresholds. RMSR (.043) is at an acceptable level.

The primary k = 5 solution was re-estimated on complete-case observations (N = 273). Model fit: RMSEA = .124 [.118, .130], TLI = .731, RMSR = .043.

**Table S10.6.** Primary k = 5 EFA fit summary (polychoric; MINRES + oblimin; complete-case N = 273; 31-item pool)

| Statistic | Value |
|---|---|
| Extraction | MINRES |
| Rotation | Oblimin |
| N (complete-case) | 273 |
| RMSR | .0428 |
| RMSEA | .1236 |
| RMSEA [90% CI] | [.118, .130] |
| TLI | .7314 |
| $\chi^2$ | 1656.02 |
| df | 320 |
| p | < .001 |

### S10.5 Primary k = 5 EFA Solution: Loading Pattern and Theoretical Alignment (31-Item Pool)

**Table S10.7.** Primary k = 5 EFA pattern matrix (calibration subsample; MINRES + oblimin; N = 273; 31-item pool)

| Item | Theoretical Block | F1raw | F2raw | F3raw | F4raw | F5raw | h² |
|---|---|---|---|---|---|---|---|
| asd1 | FRS | .032 | .037 | −.042 | .895 | .019 | .852 |
| asd2 | FRS | .017 | .079 | −.018 | .939 | .009 | .964 |

| | | | | | | | |
|---|---|---|---|---|---|---|---|
| asd3 | FRS | .039 | −.010 | .049 | .876 | .029 | .800 |
| asd4 | FRS | −.164 | .095 | −.035 | .413 | .482 | .621 |
| asd5 | FRS | −.028 | −.059 | .081 | .332 | .601 | .614 |
| asd6 | FRS | −.037 | .088 | −.016 | .256 | .560 | .566 |
| asd7 | SES | .098 | −.077 | .125 | .142 | .635 | .553 |
| asd8 | SES | .148 | .111 | .056 | −.115 | .614 | .502 |
| asd9 | SES | .189 | .202 | .055 | −.056 | .519 | .520 |
| asd10 | SES | .304 | .223 | −.121 | .050 | .364 | .442 |
| asd11 | SES | .486 | .286 | −.285 | −.063 | .291 | .489 |
| asd12 | SES | .522 | .266 | −.140 | −.051 | .170 | .445 |
| asd13 | IS | .840 | −.046 | −.014 | .039 | −.009 | .680 |
| asd14 | IS | .904 | −.040 | −.043 | .133 | −.078 | .758 |
| asd15 | IS | .730 | −.041 | .178 | −.053 | .085 | .705 |
| asd16 | IS | .825 | −.002 | .105 | −.028 | .025 | .777 |
| asd17 | IS | .733 | .009 | .126 | −.021 | .049 | .665 |
| asd18 | IS | .757 | .018 | .135 | −.005 | −.029 | .677 |
| asd19 | ES | −.032 | .660 | .137 | .136 | .120 | .706 |
| asd20 | ES | −.112 | .800 | .063 | −.038 | .173 | .755 |
| asd21 | ES | .011 | .883 | −.009 | .005 | .006 | .789 |
| asd22 | ES | .046 | .848 | −.039 | −.011 | −.024 | .692 |
| asd23 | ES | .023 | .856 | .058 | .124 | −.154 | .772 |
| asd24 | ES | .010 | .745 | .105 | .072 | .004 | .682 |
| asd25 | IIS | −.019 | .210 | .503 | .037 | .065 | .398 |
| asd26 | IIS | .021 | .251 | .525 | −.056 | .091 | .472 |
| asd27 | IIS | .062 | .159 | .713 | .012 | −.005 | .662 |
| asd28 | IIS | .020 | .013 | .846 | −.033 | −.079 | .725 |
| asd29 | IIS | .108 | .025 | .737 | .012 | .044 | .659 |
| asd30 | IIS | .109 | .021 | .742 | .015 | .081 | .683 |
| asd31 | IIS | .003 | −.064 | .794 | −.009 | .034 | .608 |

*Note.* F1raw–F5raw = raw (unaligned) factor labels from psych::fa output; alignment to theoretical blocks was performed post hoc using the Hungarian algorithm. FRS = Family–Relative Support; SES = Social Environment Support; IS = Institutional Support; ES = Emotional Support; IIS = Instrumental–Informational Support.

**Table S10.8.** Theoretical vs. empirical factor alignment after Hungarian algorithm (31-item EFA; calibration subsample)

| Item | Theoretical Block | Empirical Block (aligned) | Match | Primary Loading |
|---|---|---|---|---|
| asd1 | FRS | F1 | ✓ | .895 |
| asd2 | FRS | F1 | ✓ | .939 |
| asd3 | FRS | F1 | ✓ | .876 |
| asd4 | FRS | F2 | ✗ | .482 |
| asd5 | FRS | F2 | ✗ | .601 |
| asd6 | FRS | F2 | ✗ | .560 |
| asd7 | SES | F2 | ✓ | .635 |
| asd8 | SES | F2 | ✓ | .614 |
| asd9 | SES | F2 | ✓ | .519 |
| asd10 | SES | F2 | ✓ | .364 |
| asd11 | SES | F3 | ✗ | .486 |
| asd12 | SES | F3 | ✗ | .522 |
| asd13 | IS | F3 | ✓ | .840 |
| asd14 | IS | F3 | ✓ | .904 |
| asd15 | IS | F3 | ✓ | .730 |
| asd16 | IS | F3 | ✓ | .825 |
| asd17 | IS | F3 | ✓ | .733 |
| asd18 | IS | F3 | ✓ | .757 |
| asd19 | ES | F4 | ✓ | .660 |
| asd20 | ES | F4 | ✓ | .800 |
| asd21 | ES | F4 | ✓ | .883 |
| asd22 | ES | F4 | ✓ | .848 |
| asd23 | ES | F4 | ✓ | .856 |
| asd24 | ES | F4 | ✓ | .745 |
| asd25 | IIS | F5 | ✓ | .503 |
| asd26 | IIS | F5 | ✓ | .525 |
| asd27 | IIS | F5 | ✓ | .713 |
| asd28 | IIS | F5 | ✓ | .846 |

| | | | | |
|---|---|---|---|---|
| asd29 | IIS | F5 | ✓ | .737 |
| asd30 | IIS | F5 | ✓ | .742 |
| asd31 | IIS | F5 | ✓ | .794 |

*Note.* ✗ = item's empirical primary factor did not match its theoretical block. 26/31 items (83.9%) aligned with their theoretical blocks. Five items misaligned: asd4, asd5, asd6 (theoretical FRS → empirical SES direction); asd11, asd12 (theoretical SES → empirical IS direction). This misalignment was a key input to the item reduction triangulation (see S10.9).

**Table S10.9.** Item flagging summary from primary k = 5 EFA (31-item pool; calibration subsample)

| Item | Primary Loading | Secondary Loading | Difference | $h^2$ | Cross-loading Flag |
|---|---|---|---|---|---|
| asd10 | .364 | .304 | .060 | .442 | TRUE |
| asd4 | .482 | .413 | .069 | .621 | TRUE |
| asd11 | .486 | .291 | .195 | .489 | FALSE |
| asd25 | .503 | .210 | .293 | .398 | FALSE |
| All other 27 items | — | — | — | — | FALSE |

*Note.* Cross-loading flagged when secondary loading ≥ .30 AND primary–secondary difference < .20. No item met the low primary loading (< .30) or low communality ($h^2$ < .30) criteria. One near-threshold case is worth noting because Table S10A.8 rounds it: asd29 has a secondary loading of .2996, which appears as .300 in the pattern matrix but falls below the ≥ .30 flagging criterion, so the item is correctly unflagged.

**Table S10.10.** Factor intercorrelation matrix (phi; primary k = 5 EFA; raw factor order; 31-item pool)

| | F1raw | F2raw | F3raw | F4raw | F5raw |
|---|---|---|---|---|---|
| F1raw | 1.000 | .265 | .452 | .110 | .338 |
| F2raw | .265 | 1.000 | .350 | .438 | .460 |
| F3raw | .452 | .350 | 1.000 | .040 | .148 |
| F4raw | .110 | .438 | .040 | 1.000 | .462 |
| F5raw | .338 | .460 | .148 | .462 | 1.000 |

*Note.* Oblique (oblimin) rotation factor correlations (.04–.46) support oblique rotation and suggest factors are related but distinguishable.

**Table S10.11.** Variance explained (primary k = 5 EFA; raw factor order; 31-item pool)

| Indicator | F1raw | F2raw | F3raw | F4raw | F5raw |
|---|---|---|---|---|---|
| SS loadings | 4.990 | 4.872 | 4.080 | 3.295 | 2.994 |
| Proportion of total variance | .161 | .157 | .132 | .106 | .097 |
| Cumulative variance | .161 | .318 | .450 | .556 | .653 |
| Proportion of common variance | .247 | .241 | .202 | .163 | .148 |
| Cumulative (common variance) | .247 | .487 | .689 | .852 | 1.000 |

*Note.* Five factors together account for 65.3% of total variance.

### S10.6 EFA Sensitivity and Bootstrap Stability (31-Item Pool)

**Table S10.12.** EFA sensitivity: extraction × rotation alternatives (k = 5; 31-item pool; calibration subsample)

| Extraction | Rotation | RMSR | RMSEA | TLI | Congruence with Primary |
|---|---|---|---|---|---|
| MINRES | Promax | .0428 | .1236 | .7314 | .994 |
| ULS | Oblimin | .0428 | .1236 | .7314 | 1.000 |
| ULS | Promax | .0428 | .1236 | .7314 | .994 |

*Note.* All sensitivity alternatives produced nearly identical fit indices and very high congruence with the primary solution (≥ .994). Note that MINRES and ULS are the same minimum-residual criterion in psych::fa, so identical fit across those two rows is expected; the informative comparison is between the oblimin and promax rotations.

**Table S10.13.** Bootstrap EFA stability (B = 200; 31-item pool; calibration subsample; k = 5)

| B | Converged | % Converged | Mean | SD | Min | P05 | Median | P95 | Max | ≥ .85 | ≥ .90 | ≥ .95 |
|---|---|---|---|---|---|---|---|---|---|---|---|---|
| 200 | 200 | 100 | .859 | .082 | .718 | .744 | .868 | .970 | .980 | .530 | .395 | .185 |

*Note.* Congruence = Tucker's congruence coefficient between bootstrap-reestimated and original loading matrices (after Hungarian alignment). The bimodal distribution (concentrations near .75 and .95) suggests that the boundary items asd4, asd5, asd6, and asd7 contribute to loading instability in a subset of bootstrap resamples.

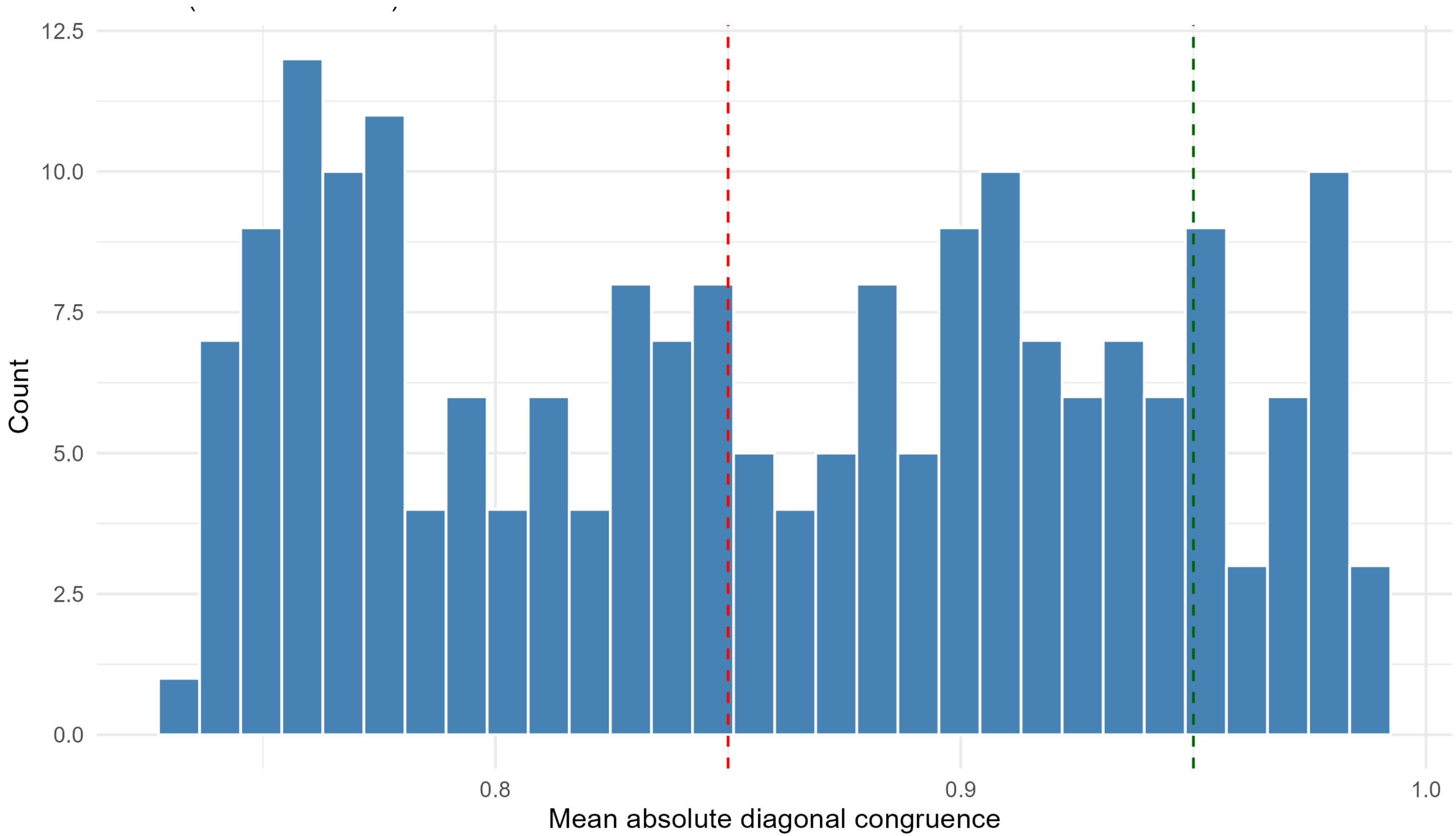


**Figure S10.3.** Bootstrap EFA stability: distribution of Tucker congruence coefficients (k = 5; B = 200; 31-item pool).

*Note.* Dashed red line = mean (.859); dashed green line = median (.868). Bimodal pattern indicates that some bootstrap resamples yielded loading patterns noticeably different from the primary solution.

### S10.7 Exploratory Graph Analysis: Primary Community Structure (31-Item Pool)

**Table S10.14.** EGA community assignments and theoretical block correspondence (31-item pool; GLASSO + Walktrap; calibration subsample)

| EGA Community | Items | Theoretical Block | Correspondence |
|---|---|---|---|
| C1 | asd1, asd2, asd3, asd4, asd5, asd6 | FRS | Full (6/6) |
| C2 | asd7, asd8, asd9, asd10, asd11, asd12 | SES | Full (6/6) |
| C3 | asd13, asd14, asd15, asd16, asd17, asd18 | IS | Full (6/6) |
| C4 | asd19, asd20, asd21, asd22, asd23, asd24 | ES | Full (6/6) |
| C5 | asd25, asd26, asd27, asd28, asd29, asd30, asd31 | IIS | Full (7/7) |

*Note.* The Leiden sensitivity algorithm reproduced this solution exactly (alignment ratio = 1.000). Unlike EFA, EGA placed all six FRS items (including asd4, asd5, asd6) in C1, preserving theoretical block membership that was obscured in EFA's oblique rotation solution.

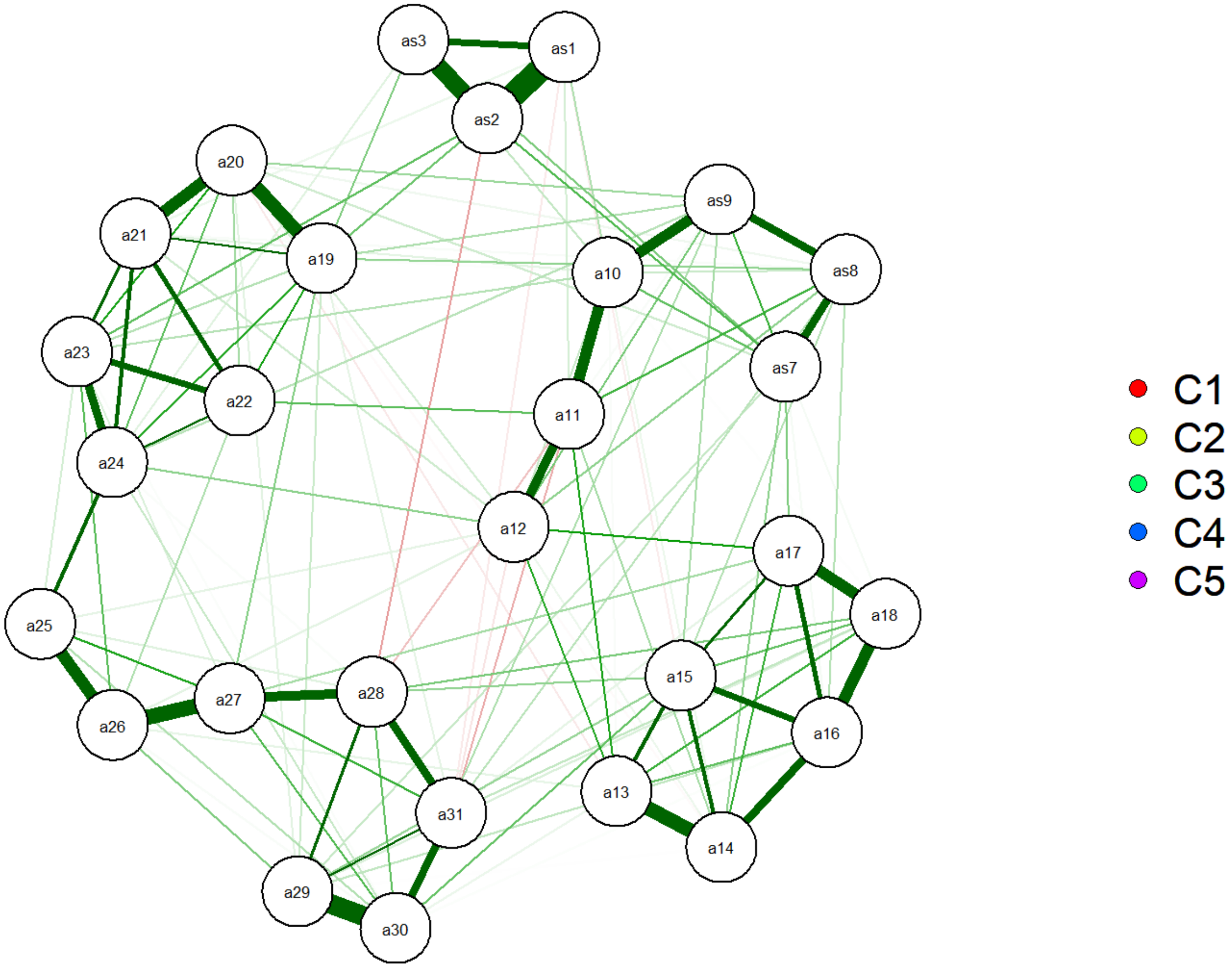


**Figure S10.4.** Primary EGA network (GLASSO + Walktrap; calibration subsample; 31-item pool).

*Note.* Node colors represent Walktrap communities. Five CFA blocks emerge as visually distinct, densely interconnected clusters.

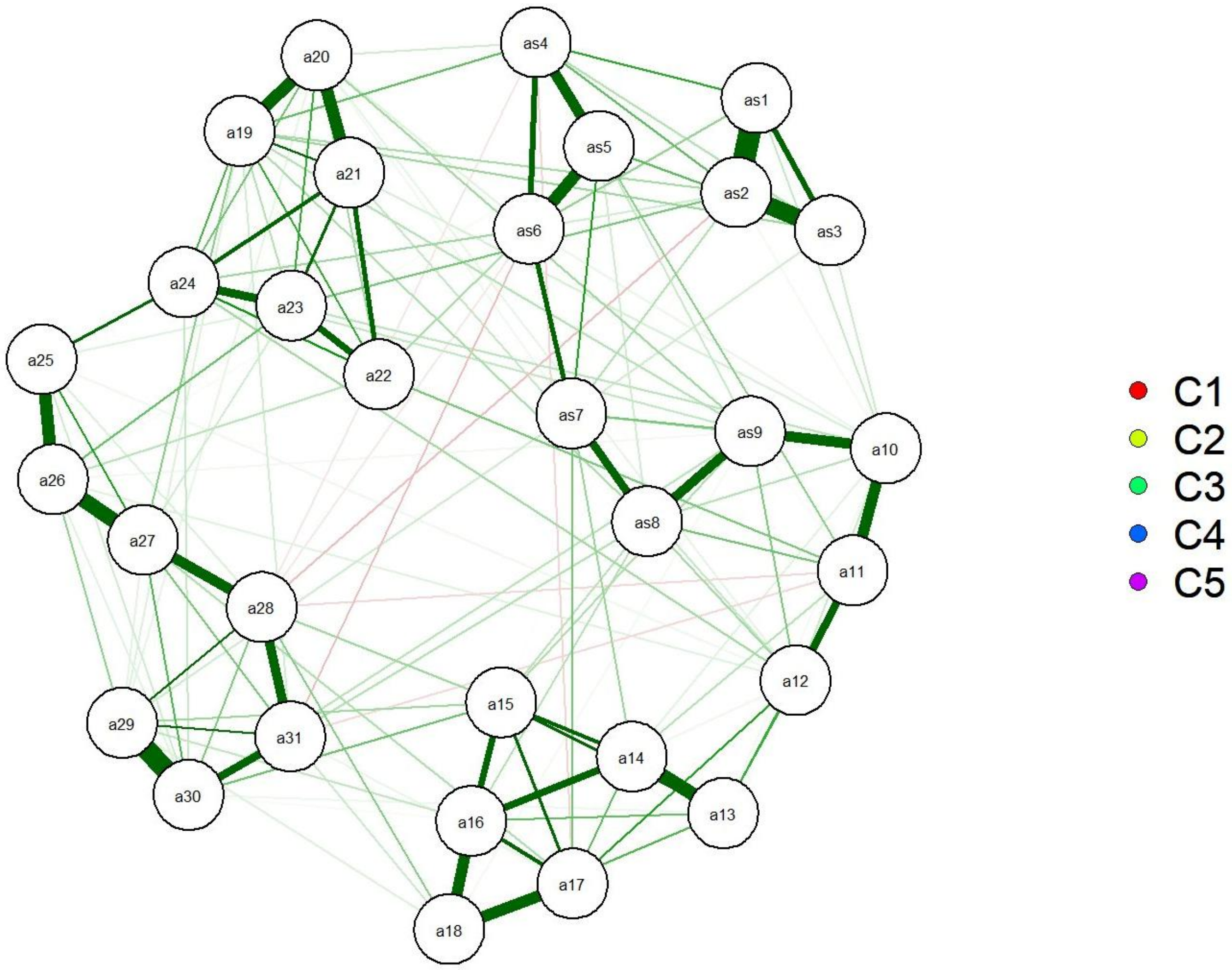


**Figure S10.5.** Sensitivity EGA network (GLASSO + Leiden; calibration subsample; 31-item pool).

*Note.* Community structure exactly replicates the Walktrap solution.

### S10.8 EGA Bootstrap Stability (31-Item Pool)

**Table S10.15.** Bootstrap dimension distribution — nonparametric bootstrap (B = 200; 31-item pool)

| k | n | Proportion |
|---|---|---|
| 4 | 2 | .010 |
| 5 | 114 | .570 |
| 6 | 75 | .375 |
| 7 | 9 | .045 |

**Table S10.16.** Structural stability summary — nonparametric bootstrap (B = 200; 31-item pool)

| k (primary) | Resamples | Valid | P(k = k_primary) | Mean Item Stability | Median | Min |
|---|---|---|---|---|---|---|
| 5 | 200 | 200 | .570 | .915 | .995 | .390 |

**Table S10.17.** Parametric bootstrap summary (EGAnet::bootEGA; B = 200; 31-item pool)

| Indicator | Value |
|---|---|
| Model | GLASSO (EBIC) |
| Correlation | cor_auto |
| Algorithm | Walktrap |
| Bootstrap type | Parametric |
| Bootstrap resamples | 200 |
| Frequency k = 5 | .720 |
| Frequency k = 6 | .275 |
| Frequency k = 7 | .005 |
| Median dimension count | 5 |
| 95% CI (median) | [4.09, 5.91] |

**Table S10.18.** EGA bootstrap item stability — ten lowest-stability items (nonparametric B = 200; 31-item pool)

| Item | Stability | Theoretical Block |
|---|---|---|
| asd5 | .390 | FRS |
| asd6 | .390 | FRS |
| asd4 | .395 | FRS |
| asd7 | .645 | SES |
| asd25 | .910 | IIS |
| asd26 | .910 | IIS |
| asd27 | .920 | IIS |
| asd12 | .955 | SES |
| asd8 | .960 | SES |
| asd11 | .980 | SES |

*Note.* Remaining 21 items had stability values of .985–1.000. Items asd4, asd5, asd6 (the three lowest-stability items) are identical to the FRS items that EFA empirically placed in the SES direction. Item asd7 (the first SES item) also showed reduced stability, likely due to its network proximity to the FRS–SES boundary.

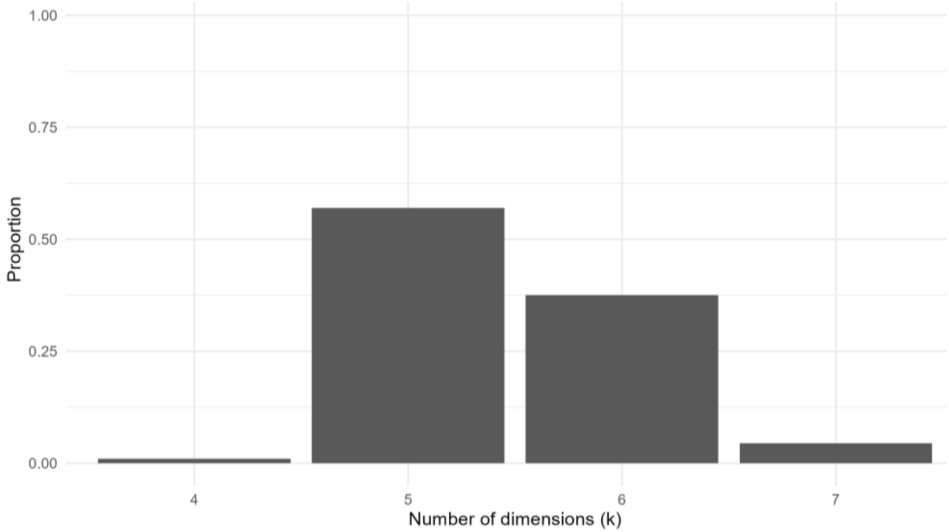


**Figure S10.6.** Bootstrap EGA dimension distribution (nonparametric B = 200; 31-item pool).

*Note.* Bars show dimension count frequency across 200 bootstrap resamples.

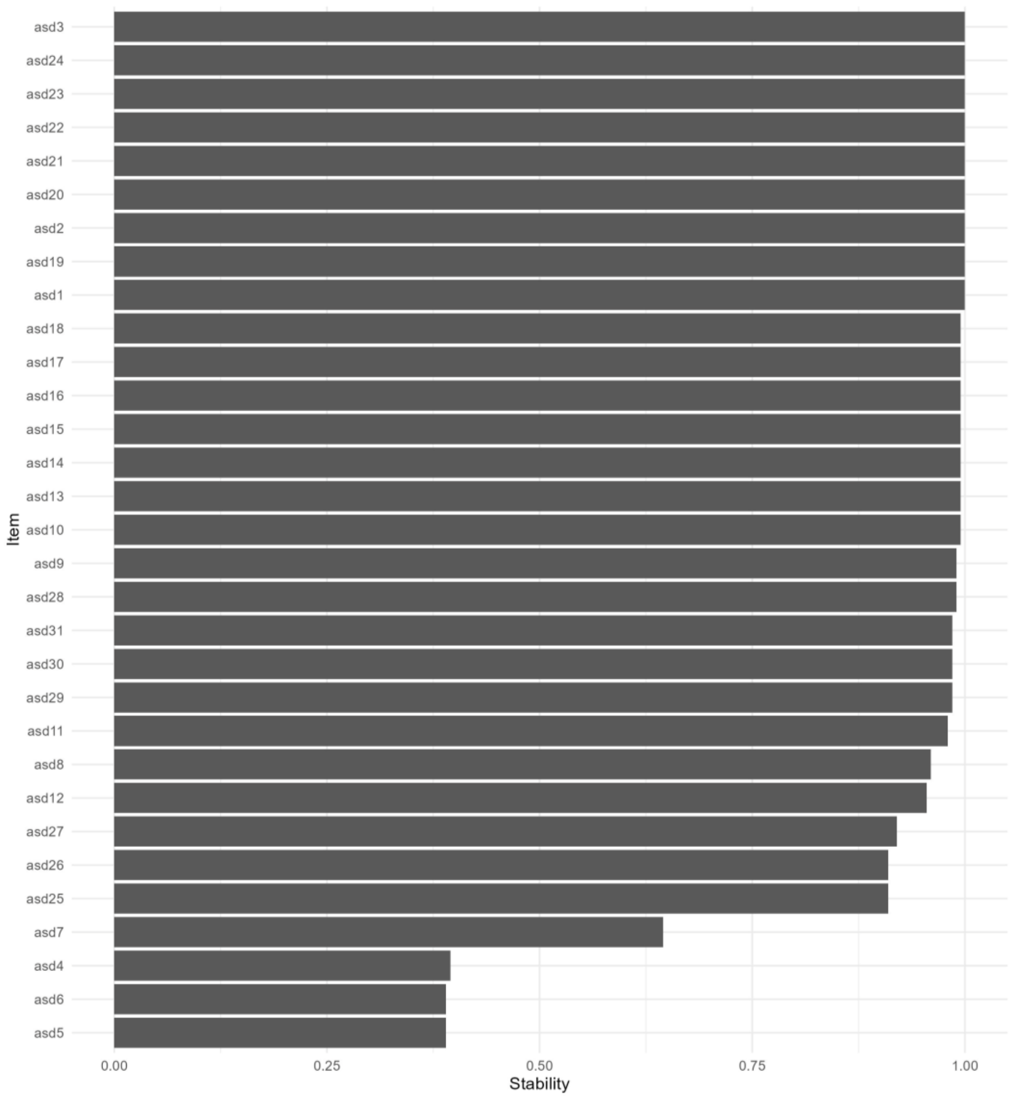


**Figure S10.7.** Bootstrap EGA item stability (nonparametric B = 200; 31-item pool).

*Note.* asd4, asd5, asd6, and asd7 are visually isolated as markedly lower-stability items.

### S10.9 Item Reduction Triangulation: Pilot EFA, Calibration EFA, and Calibration EGA

All three analysis streams operated exclusively on pilot and calibration data; the validation subsample was kept blind to all item-reduction decisions.

**Table S10.19.** Three-source triangulation for item reduction decision

| Item | Theoretical Block | Pilot EFA Signal | Calibration EFA Signal | EGA Bootstrap Stability | Triangulation Note |
|---|---|---|---|---|---|
| asd2 | FRS | Cross-loading (PA3 = .705) | Theoretically aligned; clean loading (.939) | 1.000 | Pilot-limited; calibration clear |
| asd4 | FRS | No flag ($h^2$ = .361) | Misaligned to SES; cross-loading (F1 = .413, F2 = .482) | .395 | Convergent boundary signal (2/3 sources) |
| asd5 | FRS | Cross-loading (PA5 = .497) | Misaligned to SES (F2 = .601) | .390 | Convergent signal (3/3 sources) |
| asd6 | FRS | No flag ($h^2$ = .616) | Misaligned to SES (F2 = .560) | .390 | Convergent boundary signal (2/3 sources) |
| asd7 | SES | No flag | Theoretically aligned (.635) | .645 | Near-boundary; monitor |
| asd11 | SES | No flag ($h^2$ = .690) | Misaligned to IS (F3 = .486) | .980 | EFA-limited; EGA robust |
| asd12 | SES | No flag | Misaligned to IS (F3 = .522) | .955 | EFA-limited; EGA robust |
| asd15 | IS | Low loading + cross | Theoretically aligned (.730) | 1.000 | Pilot-limited; calibration clear |
| asd17 | IS | Low loading + cross | Theoretically aligned (.733) | 1.000 | Pilot-limited; calibration clear |
| asd19 | ES | Low + cross + $h^2$ = .293 | Theoretically aligned (.660) | 1.000 | Pilot-limited; calibration clear |
| asd23 | ES | Cross-loading | Theoretically aligned (.856) | 1.000 | Pilot-limited; calibration clear |
| asd31 | IIS | Low + cross ($h^2$ = .333) | Theoretically aligned (.794) | .985 | Pilot-limited; calibration clear; main-study monitor |

*Note.* "Convergent signal" = consistent evidence across ≥ 2 of 3 independent analysis streams. Items asd11 and asd12 showed EFA misalignment but high EGA stability, suggesting the discrepancy is specific to EFA's oblique rotation. Items asd4, asd5, and asd6 were recommended for removal based on convergent evidence; this recommendation was subsequently confirmed in validation-subsample CFA and ESEM (S11). Items with pilot-only signals (asd2, asd15, asd17, asd19, asd23) were retained; their signals were attributable to small-sample EFA instability ($n$ = 130; $p/n \approx 4.2$, below the recommended 5:1 ratio). FRS = Family–Relative Support; SES = Social Environment Support; IS = Institutional Support; ES = Emotional Support; IIS = Instrumental–Informational Support.

**PART B: 28-Item Set Analyses (asd1–asd3, asd7–asd31; asd4/5/6 Removed)**

*Scope note.* Following the convergent item-reduction recommendation from the three-source triangulation (S10.9), items asd4, asd5, and asd6 were removed. The 28-item set was re-analyzed in the same calibration subsample (n = 305) using identical analytic procedures. The F1 (Family–Relative Support) block reduced from 6 to 3 items (asd1–asd3); Blocks F2–F5 remained unchanged.

**S10.10 Missing Data and Analysis N (28-Item Set)**

Missingness remained negligible. Of 305 participants, 276 (90.5%) provided complete data on all 28 items—three more than in the 31-item analyses (N = 273), because the removed items (asd4, asd5, asd6) each carried small amounts of missing data. Maximum item-level missingness was 1.31% (asd30, n = 4).

**Table S10A.1.** Item-level missingness in the calibration subsample (28-item set)

| Item | Missing n | Missing % | Block |
|---|---|---|---|
| asd1 | 0 | 0.00 | FRS |
| asd2 | 0 | 0.00 | FRS |
| asd3 | 1 | 0.33 | FRS |
| asd7 | 0 | 0.00 | SES |
| asd8 | 0 | 0.00 | SES |
| asd9 | 2 | 0.66 | SES |
| asd10 | 1 | 0.33 | SES |
| asd11 | 3 | 0.98 | SES |
| asd12 | 0 | 0.00 | SES |
| asd13 | 2 | 0.66 | IS |
| asd14 | 1 | 0.33 | IS |
| asd15 | 0 | 0.00 | IS |
| asd16 | 0 | 0.00 | IS |
| asd17 | 1 | 0.33 | IS |
| asd18 | 1 | 0.33 | IS |
| asd19 | 0 | 0.00 | ES |
| asd20 | 1 | 0.33 | ES |
| asd21 | 1 | 0.33 | ES |
| asd22 | 3 | 0.98 | ES |
| asd23 | 3 | 0.98 | ES |
| asd24 | 1 | 0.33 | ES |
| asd25 | 3 | 0.98 | IIS |

| | | | |
|---|---|---|---|
| asd26 | 0 | 0.00 | IIS |
| asd27 | 1 | 0.33 | IIS |
| asd28 | 0 | 0.00 | IIS |
| asd29 | 1 | 0.33 | IIS |
| asd30 | 4 | 1.31 | IIS |
| asd31 | 1 | 0.33 | IIS |

*Note.* Missingness percentages computed over n = 305. EFA and EGA model estimates used complete-case observations (N = 276). FRS = Family–Relative Support; SES = Social Environment Support; IS = Institutional Support; ES = Emotional Support; IIS = Instrumental–Informational Support.

**Table S10A.2.** Person-level missing data distribution (28-item set; calibration subsample)

| Missing Items | n | % |
|---|---|---|
| 0 | 276 | 90.49 |
| 1 | 27 | 8.85 |
| 2 | 2 | 0.66 |

*Note.* Total N = 305. No participant had more than two missing items.

### S10.11 EFA: Factorability and Sampling Adequacy (28-Item Set)

Compared to the 31-item pool (KMO = .816), overall sampling adequacy improved in the 28-item set (KMO = .841), consistent with the three removed items contributing somewhat less to the overall factor-analytic signal. The polychoric matrix was positive definite.

**Table S10A.3.** EFA sampling adequacy (28-item set; calibration subsample; polychoric)

| Statistic | Value |
|---|---|
| N (pairwise, for polychoric) | 305 |
| N (complete-case, for model estimation) | 276 |
| Number of items (p) | 28 |
| KMO (overall) | .841 |
| KMO (minimum item MSA) | .719 (asd28) |
| KMO (maximum item MSA) | .948 (asd12) |
| Polychoric matrix positive definite | Yes |
| Bartlett $\chi^2$ | 7757.33 |
| Bartlett df | 378 |

| | |
|---|---|
| Bartlett p | < .001 |

*Note.* No item fell below the .50 MSA threshold. KMO = .841 indicates "very good" sampling adequacy. The improvement from .816 (31-item) reflects that item removal did not degrade factorability.

### S10.12 Factor/Dimension Retention Evidence (28-Item Set, Diagnostic)

Statistical retention indicators shifted slightly relative to the 31-item analyses. Parallel analysis, MAP, and Kaiser's criterion now suggested k = 6 (vs. k = 7 for the 31-item pool); the automatic scree elbow pointed to k = 4 (vs. k = 5 for the 31-item pool). The theoretical k = 5 was retained as the primary solution.

**Table S10A.4.** Factor retention methods comparison (28-item set; calibration subsample)

| Method | Suggested k | Note |
|---|---|---|
| Parallel Analysis (PA) | 6 | — |
| Velicer's MAP | 6 | Minimum MAP = .0241 at k = 6; Δ (k = 5 vs. k = 6) = .0036 |
| Scree (automatic elbow) | 4 | One below theoretical k |
| Kaiser criterion (eigenvalue > 1) | 6 | — |
| Hull method | NA | Same software constraint as in 31-item analysis |

*Note.* Statistical indicators suggest a slightly lower k than in the 31-item pool (k = 6 vs. k = 7 for PA/MAP/Kaiser; k = 4 vs. k = 5 for scree). The MAP value at k = 5 vs. k = 6 difference is small (Δ = .0036), and k = 7 shows MAP increasing again, indicating a "shallow valley" around k = 6–7. These are diagnostic outputs; the theoretical k = 5 was maintained as primary.

**Table S10A.5.** Velicer MAP values across candidate k (28-item set; calibration subsample)

| k | MAP |
|---|---|
| 1 | .0745 |
| 2 | .0555 |
| 3 | .0370 |
| 4 | .0307 |
| 5 | .0286 |
| 6 | .0241 |
| 7 | .0252 |
| 8 | .0283 |

*Note.* MAP reaches its minimum at k = 6 (.0241); the k = 5–k = 6 difference is small (Δ = .0036).

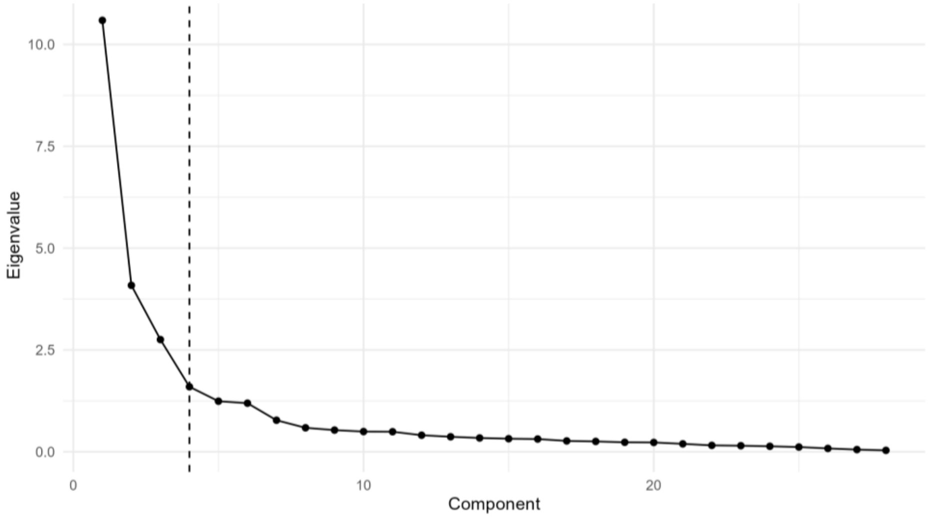


**Figure S10A.1.** Scree plot (polychoric eigenvalues; 28-item set; calibration subsample). *Note.* Dashed line = automatic elbow (k = 4); theoretical k = 5 is one step above.

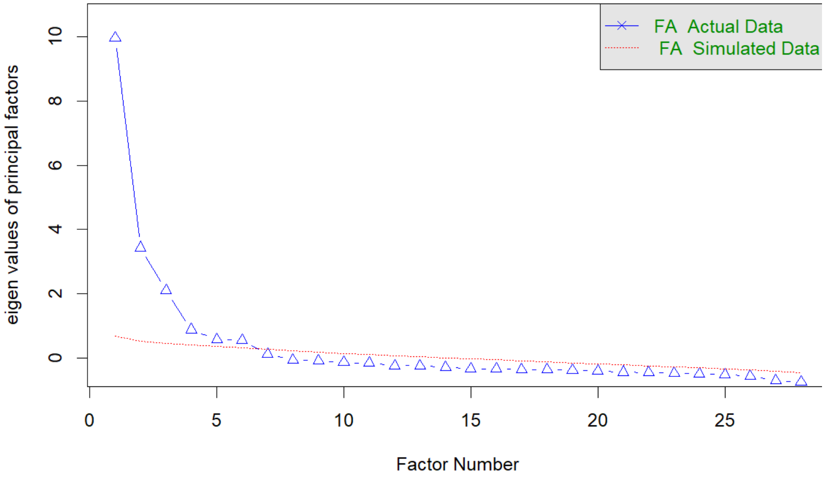


**Figure S10A.2.** Parallel analysis (polychoric; 28-item set; calibration subsample). *Note.* Observed eigenvalues exceed simulated values through the first six components; the separation weakens at the seventh.

### S10.13 EFA Model Fit: k = 1–7 Comparison and Primary k = 5 Solution (28-Item Set)

Global fit improved modestly relative to the 31-item solution. The k = 5 RMSEA decreased from .124 to .118 and TLI increased from .731 to .778. Both indices remained below conventional acceptability thresholds (RMSEA ≤ .08; TLI ≥ .90); this was expected given the known inflation properties of these indices in short, oblique polychoric EFA structures.

**Table S10A.6.** EFA model fit comparison across candidate k values (polychoric, MINRES; calibration subsample; 28-item set)

| k | Rotation | RMSR | RMSEA | RMSEA [90% CI] | TLI | BIC |
|---|---|---|---|---|---|---|
| 1 | none | .167 | .206 | [.202, .212] | .333 | 2892.35 |
| 3 | oblimin | .067 | .157 | [.151, .163] | .613 | 825.04 |
| 4 | oblimin | .049 | .129 | [.123, .135] | .739 | 88.99 |
| 5 | oblimin | .040 | .120 | [.114, .126] | .773 | −86.47 |
| 6 | oblimin | .027 | .110 | [.104, .117] | .808 | −230.93 |
| 7 | oblimin | .022 | .105 | [.098, .112] | .825 | −275.67 |

*Note.* k = 2 failed to converge and was excluded. The k = 5 solution is retained as primary on theoretical grounds. BIC favors more complex solutions but model selection was theory-driven.

**Table S10A.7.** Primary k = 5 EFA fit summary (polychoric; MINRES + oblimin; complete-case N = 276; 28-item set)

| Statistic | Value |
|---|---|
| Extraction | MINRES |
| Rotation | Oblimin |
| N (complete-case) | 276 |
| RMSR | .040 |
| RMSEA | .118 |
| RMSEA [90% CI] | [.111, .125] |
| TLI | .778 |
| BIC | −194.68 |
| $\chi^2$ | 1199.18 |
| df | 248 |
| p | < .001 |

*Note.* Compared to the 31-item primary solution (RMSEA = .124, TLI = .731), the 28-item solution shows modest global fit improvement. RMSR (.040) remains acceptable in both solutions.

### S10.14 Primary k = 5 EFA Solution: Loading Pattern and Theoretical Alignment (28-Item Set)

The pattern matrix revealed a key structural consequence of the FRS block reduction: the SES (asd7–asd12) and IS (asd13–asd18) blocks, which were clearly separated in the 31-item solution, merged into a single large raw factor (F1raw). This occurred because reducing F1/FRS from 6 to 3 items altered the balance of the factor space, making it harder for the oblique rotation to distinguish SES and IS loadings. Conversely, the FRS block (now 3 items: asd1–asd3) and the ES block (asd19–asd24) emerged as highly clean, well-separated factors. The IIS block split into two sub-clusters (asd25–asd27 in F4raw; asd28–asd31 in F5raw).

**Table S10A.8.** Primary k = 5 EFA pattern matrix (calibration subsample; MINRES + oblimin; N = 276; 28-item set)

| Item | Theoretical Block | F1 | F2 | F3 | F4 | F5 |
|---|---|---|---|---|---|---|
| asd1 | FRS | −.033 | −.026 | **.919** | .036 | −.053 |
| asd2 | FRS | −.042 | .033 | **.973** | .000 | −.002 |
| asd3 | FRS | .019 | −.012 | **.921** | −.041 | .086 |
| asd7 | SES | .349 | .169 | .348 | −.068 | .021 |
| asd8 | SES | .408 | .346 | .132 | −.138 | −.005 |
| asd9 | SES | .377 | .322 | .180 | .022 | −.094 |
| asd10 | SES | .374 | .193 | .246 | .112 | −.296 |
| asd11 | SES | **.499** | .193 | .053 | .134 | −.475 |
| asd12 | SES | **.517** | .167 | .017 | .170 | −.318 |
| asd13 | IS | **.808** | −.148 | .026 | .128 | −.121 |
| asd14 | IS | **.843** | −.126 | .099 | .032 | −.066 |
| asd15 | IS | **.834** | .041 | −.068 | −.043 | .153 |
| asd16 | IS | **.880** | .033 | −.050 | −.045 | .098 |
| asd17 | IS | **.790** | .040 | −.018 | −.007 | .088 |
| asd18 | IS | **.787** | .028 | −.045 | .005 | .108 |
| asd19 | ES | .043 | **.789** | .152 | −.062 | .138 |
| asd20 | ES | −.023 | **.970** | −.019 | −.111 | .075 |
| asd21 | ES | −.002 | **.900** | −.014 | .001 | −.024 |
| asd22 | ES | −.010 | **.767** | −.045 | .141 | −.128 |
| asd23 | ES | −.080 | **.705** | .087 | .228 | −.063 |
| asd24 | ES | −.019 | **.683** | .064 | .207 | −.041 |
| asd25 | IIS | −.044 | .038 | .075 | **.737** | .018 |
| asd26 | IIS | −.006 | .059 | −.019 | **.837** | −.027 |
| asd27 | IIS | .087 | .043 | .022 | **.693** | .251 |

| | | | | | | |
|---|---|---|---|---|---|---|
| asd28 | IIS | .127 | .053 | −.068 | .421 | **.548** |
| asd29 | IIS | .254 | .111 | .037 | .300 | **.493** |
| asd30 | IIS | .263 | .101 | .053 | .334 | **.466** |
| asd31 | IIS | .184 | .093 | .011 | .190 | **.645** |

*Note.* F1–F5 are raw (unaligned) psych::fa output labels. Bold = highest absolute loading per item. The FRS block (asd1–asd3) shows very high, clean loadings on F3 (.919–.973). The IS block (asd13–asd18) shows high loadings on F1 (.787–.880), but several SES items (asd7–asd12) also load on F1 (.349–.517), reflecting the SES–IS merger in this solution. The IIS block splits across F4 (asd25–asd27) and F5 (asd28–asd31).

**Table S10A.9.** Theoretical vs. empirical factor alignment after Hungarian algorithm (28-item EFA; calibration subsample)

| Item | Theoretical Block | Empirical Block (aligned) | Match | Primary Loading |
|---|---|---|---|---|
| asd1 | FRS | F1 | ✓ | .919 |
| asd2 | FRS | F1 | ✓ | .973 |
| asd3 | FRS | F1 | ✓ | .921 |
| asd7 | SES | F2 | ✓ | .349 |
| asd8 | SES | F2 | ✓ | .408 |
| asd9 | SES | F2 | ✓ | .377 |
| asd10 | SES | F2 | ✓ | .374 |
| asd11 | SES | F2 | ✓ | .499 |
| asd12 | SES | F2 | ✓ | .517 |
| asd13 | IS | **F2** | ✗ | .808 |
| asd14 | IS | **F2** | ✗ | .843 |
| asd15 | IS | **F2** | ✗ | .834 |
| asd16 | IS | **F2** | ✗ | .880 |
| asd17 | IS | **F2** | ✗ | .790 |
| asd18 | IS | **F2** | ✗ | .787 |
| asd19 | ES | F4 | ✓ | .789 |
| asd20 | ES | F4 | ✓ | .970 |
| asd21 | ES | F4 | ✓ | .900 |
| asd22 | ES | F4 | ✓ | .767 |
| asd23 | ES | F4 | ✓ | .705 |

| | | | | |
|---|---|---|---|---|
| asd24 | ES | F4 | ✓ | .683 |
| asd25 | IIS | **F3** | ✗ | .737 |
| asd26 | IIS | **F3** | ✗ | .837 |
| asd27 | IIS | **F3** | ✗ | .693 |
| asd28 | IIS | F5 | ✓ | .548 |
| asd29 | IIS | F5 | ✓ | .493 |
| asd30 | IIS | F5 | ✓ | .466 |
| asd31 | IIS | F5 | ✓ | .645 |

*Note.* ✗ = mismatch with theoretical block. 19/28 items (67.9%) aligned; 9 misaligned (vs. 26/31 = 83.9% for the 31-item solution). The apparent deterioration is a rotation artifact: in the raw EFA solution, SES items (asd7–asd12) and IS items (asd13–asd18) loaded on the same large factor (F1raw); the Hungarian algorithm could assign this merged factor to only one theoretical block (SES), causing all IS items to be labeled "misaligned." Similarly, IIS sub-cluster asd25–asd27 was assigned to a separate raw factor (F4raw) that the algorithm maps to F3/IS — not because these items structurally belong there, but because F4raw was the remaining unassigned factor. Critically, EGA (reported in S10.16) correctly recovered all five theoretical communities even in this 28-item set, confirming that the EFA alignment deterioration is a rotation artifact rather than a true structural failure.

**Table S10A.10.** Communalities ($h^2$) and uniqueness ($u^2$) — primary k = 5 EFA (28-item set; ascending order)

| **Item** | **$h^2$** | **$u^2$** | **Theoretical Block** |
|---|---|---|---|
| asd7 | .376 | .624 | SES |
| asd8 | .377 | .624 | SES |
| asd9 | .443 | .557 | SES |
| asd10 | .475 | .525 | SES |
| asd12 | .492 | .508 | SES |
| asd11 | .569 | .432 | SES |
| asd25 | .573 | .427 | IIS |
| asd22 | .656 | .344 | ES |
| asd29 | .658 | .342 | IIS |
| asd24 | .665 | .335 | ES |
| asd31 | .667 | .333 | IIS |
| asd18 | .668 | .332 | IS |
| asd17 | .668 | .332 | IS |
| asd13 | .671 | .329 | IS |
| asd30 | .672 | .328 | IIS |

| | | | |
|---|---|---|---|
| asd14 | .698 | .302 | IS |
| asd23 | .708 | .292 | ES |
| asd28 | .712 | .288 | IIS |
| asd26 | .724 | .276 | IIS |
| asd15 | .738 | .262 | IS |
| asd27 | .739 | .261 | IIS |
| asd19 | .768 | .232 | ES |
| asd16 | .786 | .214 | IS |
| asd21 | .796 | .204 | ES |
| asd3 | .823 | .177 | FRS |
| asd1 | .836 | .164 | FRS |
| asd20 | .844 | .156 | ES |
| asd2 | .965 | .035 | FRS |

*Note.* No item fell below the $h^2 < .30$ threshold (minimum = .376 for asd7). The lowest communalities are systematically in the SES block (asd7–asd12; $h^2$ = .376–.569), reflecting cross-block loading spread in the merged SES–IS factor. The highest communalities are in FRS (.823–.965) and ES (.656–.844) blocks.

**Table S10A.11.** Item flagging summary (primary k = 5 EFA; 28-item set)

| Item | Primary Loading | Secondary Loading | Difference | $h^2$ | Cross-loading Flag |
|---|---|---|---|---|---|
| asd7 | .349 | .348 | .001 | .376 | TRUE |
| asd9 | .377 | .322 | .055 | .443 | TRUE |
| asd8 | .408 | .346 | .062 | .377 | TRUE |
| asd30 | .466 | .334 | .132 | .672 | TRUE |
| asd11 | .499 | .475 | .024 | .569 | TRUE |
| asd12 | .517 | .318 | .199 | .492 | TRUE |
| asd28 | .548 | .421 | .127 | .712 | TRUE |
| All other 21 items | — | — | — | — | FALSE |

*Note.* Seven items (25.0%) received cross-loading flags in the 28-item solution, compared to two (6.5%) in the 31-item solution. This increase is the direct consequence of the SES–IS merger in F1raw and the IIS split across F4raw/F5raw — both rotation-space artifacts of the FRS block reduction, not item-quality failures. No item met the low primary loading (< .30) or low communality ($h^2 < .30$) criteria.

**Table S10A.12.** Factor intercorrelation matrix (phi; primary k = 5 EFA; raw factor order; 28-item set)

| | F1 | F2 | F3 | F4 | F5 |
|---|---|---|---|---|---|
| F1 | 1.000 | .308 | .180 | .430 | .178 |
| F2 | .308 | 1.000 | .482 | .433 | .067 |
| F3 | .180 | .482 | 1.000 | .159 | −.102 |
| F4 | .430 | .433 | .159 | 1.000 | .262 |
| F5 | .178 | .067 | −.102 | .262 | 1.000 |

*Note.* Values range from −.102 to .482. The relatively high F2–F3 correlation (.482) reflects the SES–IS proximity in the merged F1raw factor, partially captured across two raw factors in this solution. The negative F3–F5 correlation (−.102) is an artefact of factor orientation rather than an inverse substantive relationship: three SES items (asd10, asd11, asd12) carry negative loadings on F5raw in this solution, which reverses the sign of correlations involving that factor.

**Table S10A.13.** Variance explained (primary k = 5 EFA; raw factor order; 28-item set)

| Indicator | F1 | F2 | F3 | F4 | F5 |
|---|---|---|---|---|---|
| SS loadings | 5.777 | 4.892 | 3.195 | 2.925 | 1.977 |
| Proportion of total variance | .206 | .175 | .114 | .105 | .071 |
| Cumulative variance | .206 | .381 | .495 | .600 | .670 |
| Proportion of common variance | .308 | .261 | .170 | .156 | .105 |
| Cumulative (common variance) | .308 | .569 | .739 | .895 | 1.000 |

*Note.* Five factors account for 67.0% of total variance — comparable to the 65.3% from the 31-item solution.

### S10.15 EFA Sensitivity and Bootstrap Stability (28-Item Set)

**Table S10A.14.** EFA sensitivity: extraction × rotation alternatives (k = 5; 28-item set; calibration subsample)

| Extraction | Rotation | RMSR | RMSEA | TLI | Congruence with Primary |
|---|---|---|---|---|---|
| MINRES | Promax | .040 | .118 | .778 | .968 |
| ULS | Oblimin | .040 | .118 | .778 | 1.000 |
| ULS | Promax | .040 | .118 | .778 | .968 |

*Note.* All alternatives produced identical fit indices and very high congruence with the primary solution (≥ .968), confirming that the loading pattern is stable across extraction and rotation choices.

**Table S10A.15.** Bootstrap EFA stability (B = 200; 28-item set; calibration subsample; k = 5)

| B | Converged | % Converged | Mean | SD | Min | P05 | Median | P95 | Max | ≥ .85 | ≥ .90 | ≥ .95 | ≥ .98 |
|---|---|---|---|---|---|---|---|---|---|---|---|---|---|
| 200 | 200 | 100 | .858 | .076 | .732 | .746 | .858 | .976 | .988 | .525 | .360 | .155 | .045 |

*Note.* Despite removing asd4/5/6, the mean congruence (.858, SD = .076) is nearly identical to the 31-item solution (.859, SD = .082). The bimodal distribution pattern persists, concentrated near .73–.80 and .90–.99, indicating that the SES–IS merged factor and IIS split continue to generate loading instability in a subset of bootstrap resamples.

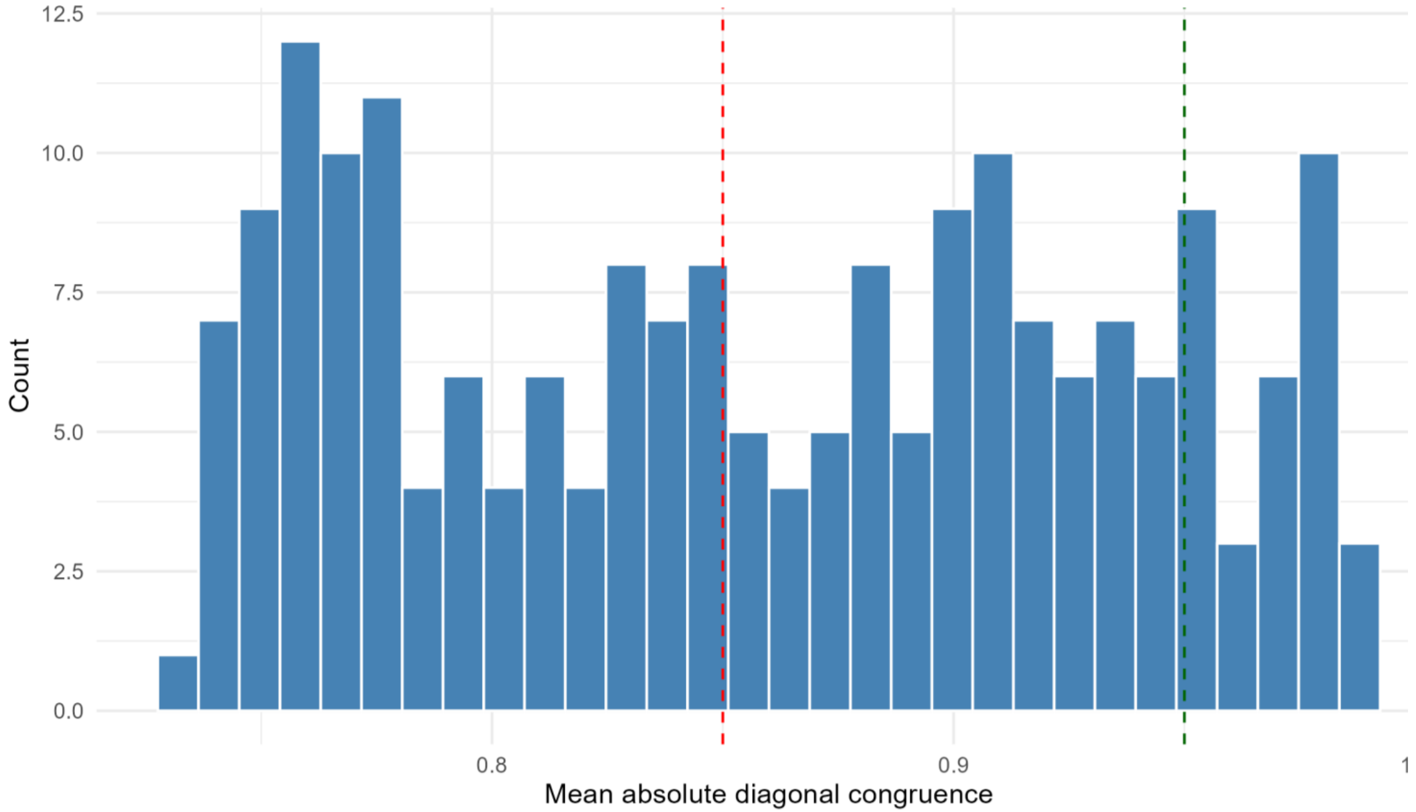


**Figure S10A.3.** Bootstrap EFA stability: distribution of Tucker congruence coefficients (k = 5; B = 200; 28-item set).

*Note.* Dashed red line = mean (.858); dashed green line = median (.858). The bimodal distribution — compared to the 31-item solution — shows that removing asd4/5/6 did not fully resolve EFA's loading instability; the SES–IS merged factor remains the primary source of variance.

### S10.16 Exploratory Graph Analysis: Primary Community Structure (28-Item Set)

In marked contrast to the EFA results, EGA in the 28-item set produced a complete, exact match between the five detected communities and the five theoretical blocks. Unlike EFA's oblique rotation, which merged SES and IS into a single factor, the network-based community detection maintained distinct communities for all five theoretical blocks — confirming that the SES–IS separation is a real structural feature of the data that EFA's rotation approach failed to recover in the 28-item context.

**Table S10A.16.** EGA community assignments and theoretical block correspondence (28-item set; GLASSO + Walktrap; calibration subsample)

| EGA Community | Items | Theoretical Block | Correspondence |
|---|---|---|---|
| C1 | asd1, asd2, asd3 | FRS | Full (3/3) |
| C2 | asd7, asd8, asd9, asd10, asd11, asd12 | SES | Full (6/6) |
| C3 | asd13, asd14, asd15, asd16, asd17, asd18 | IS | Full (6/6) |
| C4 | asd19, asd20, asd21, asd22, asd23, asd24 | ES | Full (6/6) |
| C5 | asd25, asd26, asd27, asd28, asd29, asd30, asd31 | IIS | Full (7/7) |

*Note.* 28/28 items (100%) aligned with theoretical blocks — a marked improvement over the 31-item EGA (which correctly placed asd4/5/6 in FRS community but showed those items had low stability) and a complete divergence from the 28-item EFA (67.9% alignment). EGA correctly separated SES and IS into distinct communities even though EFA merged them.

**Table S10A.17.** Walktrap vs. Leiden community agreement (28-item set; calibration subsample)

| k (Walktrap) | k (Leiden) | Post-alignment agreement |
|---|---|---|
| 5 | 5 | 1.000 |

*Note.* The Leiden sensitivity algorithm reproduced the Walktrap solution exactly.

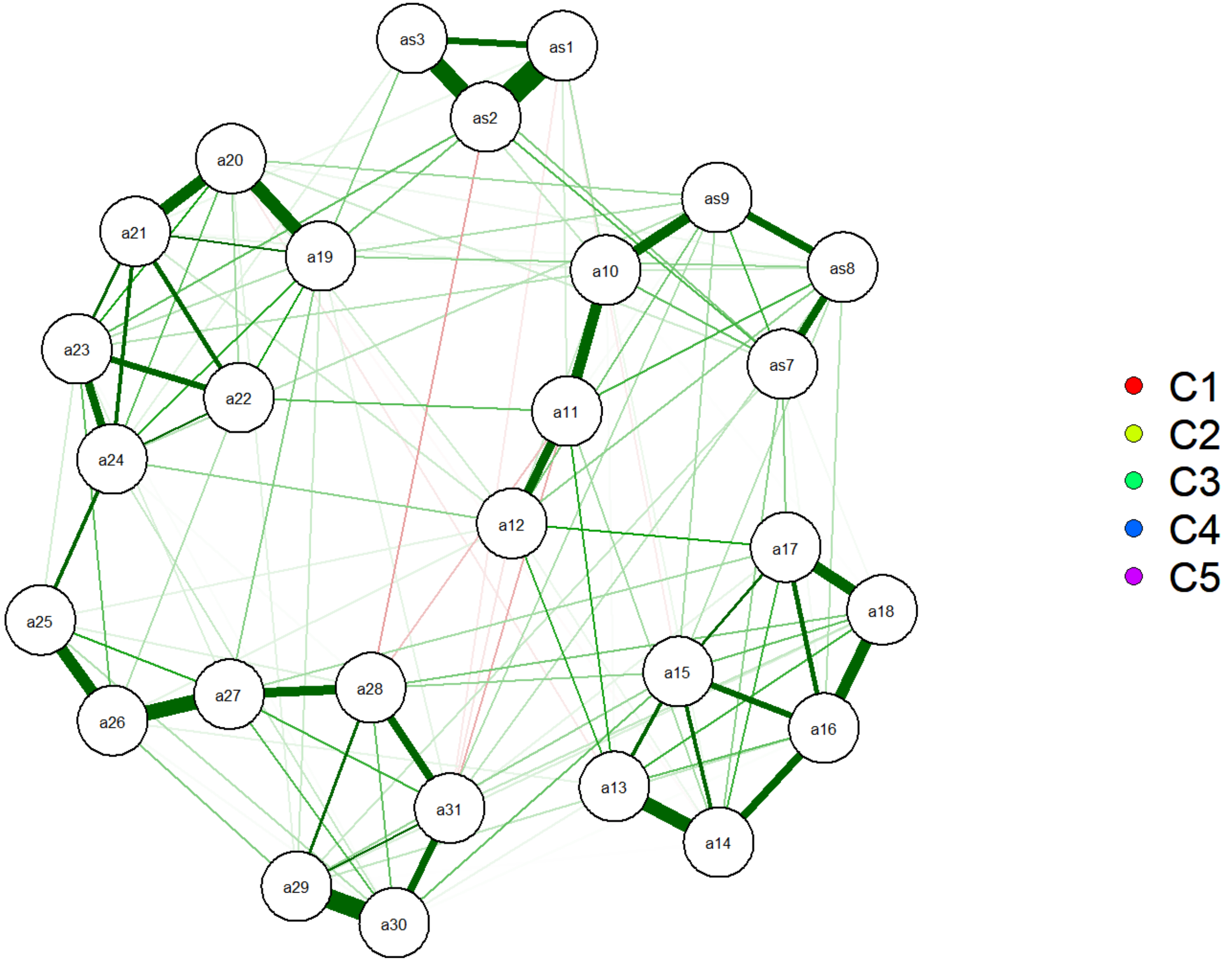


**Figure S10A.4.** Primary EGA network (GLASSO + Walktrap; calibration subsample; 28-item set).

*Note.* Node colors represent Walktrap communities. Five distinct, densely interconnected clusters are visible, each corresponding to a theoretical block. Note the clear separation between the SES (C2) and IS (C3) communities — a separation that EFA's oblique rotation failed to recover in this 28-item set.

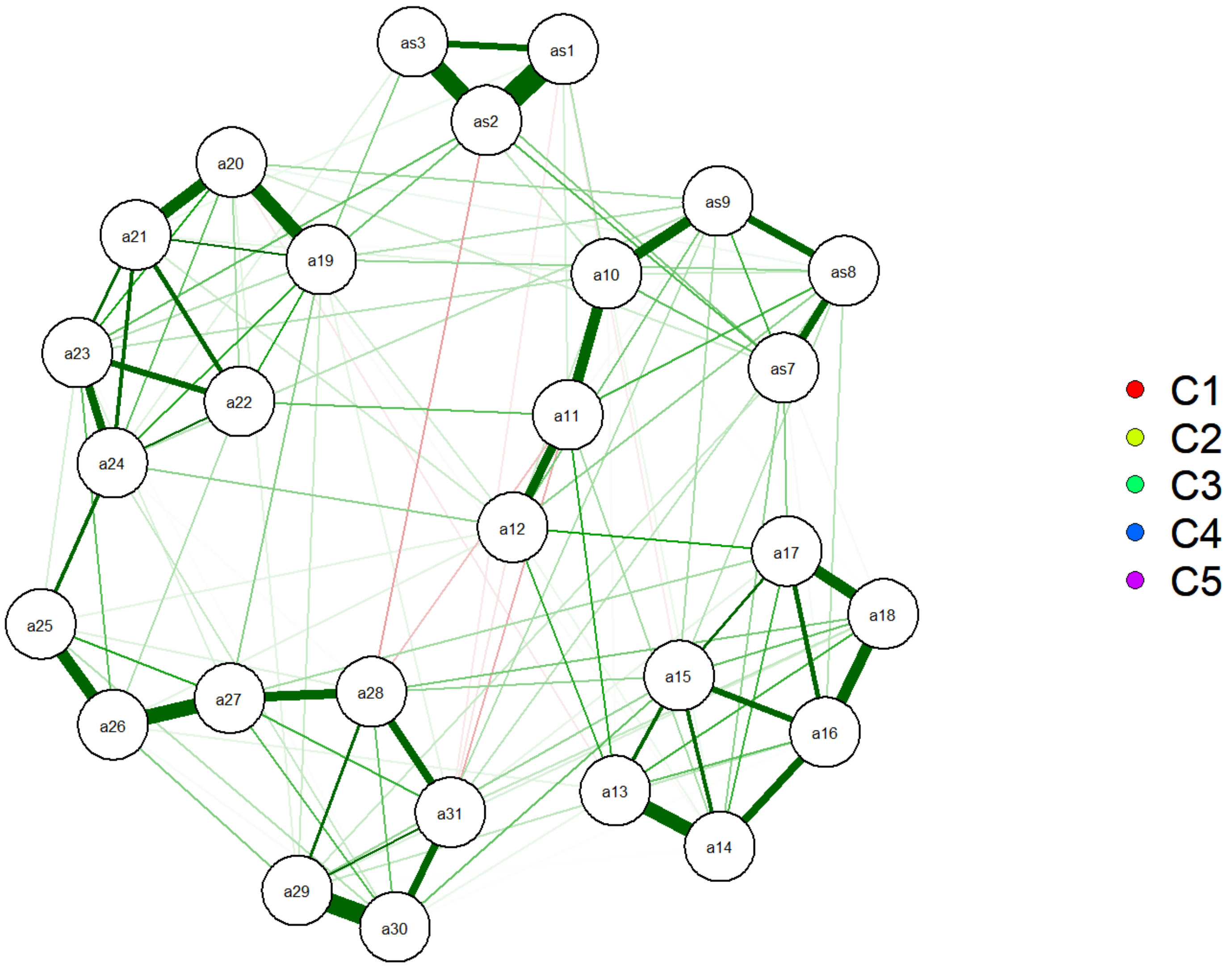


**Figure S10A.5.** Sensitivity EGA network (GLASSO + Leiden; calibration subsample; 28-item set).

*Note.* Community structure exactly replicates the Walktrap solution.

### S10.17 EGA Bootstrap Stability (28-Item Set)

Bootstrap results for the 28-item set showed a dramatic improvement relative to both the 31-item pool EGA and the 28-item EFA bootstraps. The five-dimensional solution was reproduced in 97.0–98.5% of bootstrap resamples, and mean item stability reached .994.

**Table S10A.18.** Bootstrap dimension distribution — nonparametric bootstrap (B = 200; 28-item set)

| k | n | Proportion |
|---|---|---|
| 4 | 1 | .005 |
| 5 | 194 | .970 |
| 6 | 5 | .025 |

**Table S10A.19.** Structural stability summary — nonparametric bootstrap (B = 200; 28-item set)

| k (primary) | Resamples | Valid | P(k = k_primary) | Mean Item Stability | Median | Min |
|---|---|---|---|---|---|---|
| 5 | 200 | 200 | .970 | .994 | .998 | .965 |

*Note.* Compared to the 31-item EGA bootstrap (P(k = 5) = .570; mean item stability = .915), the 28-item EGA shows markedly higher structural stability and item-level consistency.

**Table S10A.20.** Parametric bootstrap summary (EGAnet::bootEGA; B = 200; 28-item set)

| Indicator | Value |
|---|---|
| Model | GLASSO (EBIC) |
| Correlation | cor_auto |
| Algorithm | Walktrap |
| Unidimensionality method | Louvain |
| Bootstrap resamples | 200 (parametric) |
| Frequency k = 5 | .985 |
| Frequency k = 6 | .015 |
| Median dimension count | 5 |
| 95% CI (median) | [4.76, 5.24] |

**Table S10A.21.** EGA bootstrap item stability — lowest 6 items (nonparametric B = 200; 28-item set)

| Item | Stability | Theoretical Block |
|---|---|---|
| asd25 | .965 | IIS |
| asd26 | .965 | IIS |
| asd27 | .970 | IIS |
| asd12 | .985 | SES |
| asd1 | .995 | FRS |
| asd2 | .995 | FRS |

*Note.* All 28 items achieved stability ≥ .965 — substantially higher than the lowest values observed in the 31-item solution (.390 for asd5/asd6). The three lowest-stability items (asd25, asd26, asd27) are the first three IIS items, the same items that EFA isolated in a separate sub-cluster (F4raw); however, at .965 these values are very high and do not constitute a structural concern in the EGA framework.

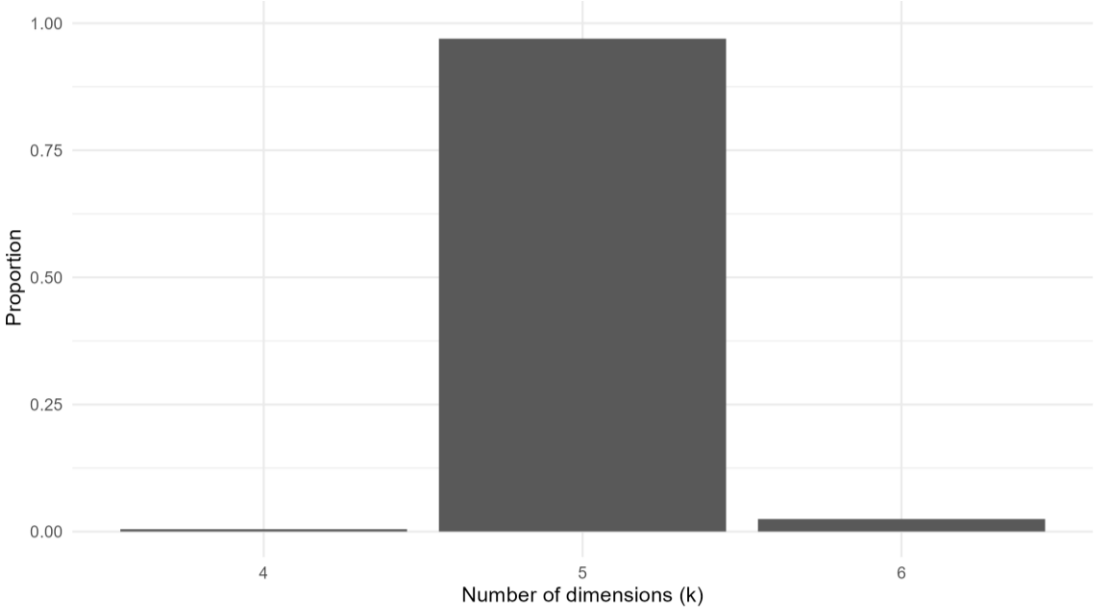


**Figure S10A.6.** Bootstrap EGA dimension distribution (nonparametric B = 200; 28-item set).

*Note.* Nearly all mass is concentrated at k = 5 (97.0%).

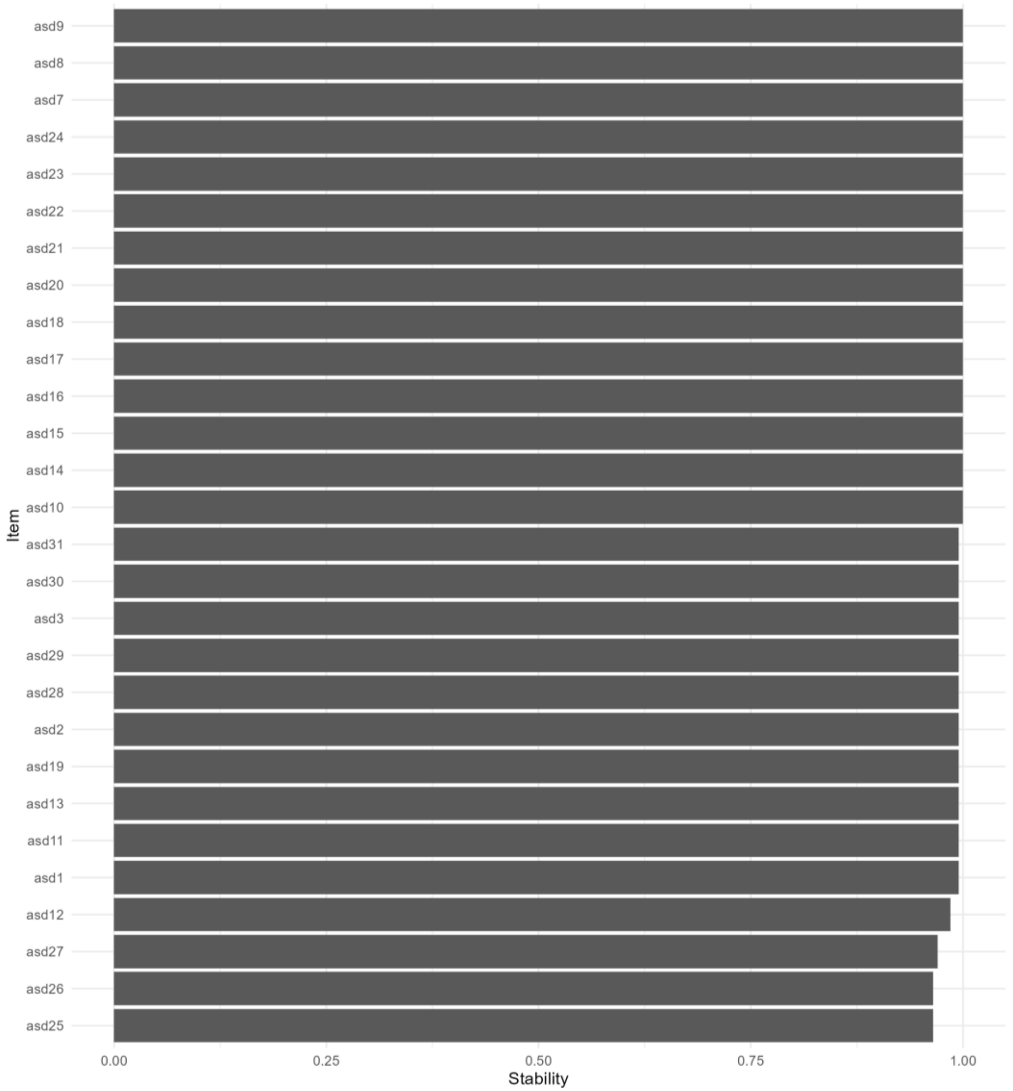


**Figure S10A.7.** Bootstrap EGA item stability (nonparametric B = 200; 28-item set).

*Note.* All items show stability ≥ .965; the pronounced low-stability cluster observed for asd4–asd7 in the 31-item solution is absent.

### S10.18 EFA–EGA Convergence (28-Item Set)

**Table S10A.22.** EFA–EGA item-level alignment summary (28-item set)

| k (EFA empirical) | k (EGA) | Alignment ratio | Items (n) |
|---|---|---|---|
| 5 | 5 | .679 | 28 |

*Note.* 19/28 items (67.9%) showed matching assignments between EFA's Hungarian-aligned empirical factors and EGA's Walktrap communities.

**Table S10A.23.** EFA × EGA cross-tabulation (item counts; aligned labels; 28-item set)

| EFA Factor | EGA C1 (FRS) | EGA C2 (SES) | EGA C3 (IS) | EGA C4 (ES) | EGA C5 (IIS) |
|---|---|---|---|---|---|
| F1 (empirical) | 0 | 6 | 6 | 0 | 0 |
| F2 (empirical) | 0 | 0 | 0 | 6 | 0 |
| F3 (empirical) | 3 | 0 | 0 | 0 | 0 |
| F4 (empirical) | 0 | 0 | 0 | 0 | 3 |
| F5 (empirical) | 0 | 0 | 0 | 0 | 4 |

*Note.* EGA C1–C5 labels were aligned to EFA empirical factors via the Hungarian algorithm; EGA labels in this table correspond to theoretical blocks as follows: C1 = FRS, C2 = SES, C3 = IS, C4 = ES, C5 = IIS. The critical discordance is in EFA F1 (12 items): EGA correctly separates these 12 items into SES (6 items, C2) and IS (6 items, C3), while EFA's oblique rotation merges them. For IIS: EFA splits 7 items into F4 (asd25–asd27, 3 items) and F5 (asd28–asd31, 4 items), while EGA correctly unifies all 7 as C5.

**Table S10A.24.** Discordant items: detailed EFA–EGA comparison (28-item set)

| Item | EFA Factor (aligned) | EGA Behavior | Theoretical Block | EGA Bootstrap Stability |
|---|---|---|---|---|
| asd13 | F1 (merged with SES items) | EGA assigns to IS community (C3) — correctly separated | IS | .985+ |
| asd14 | F1 (merged with SES items) | EGA assigns to IS community (C3) — correctly separated | IS | .985+ |
| asd15 | F1 (merged with SES items) | EGA assigns to IS community (C3) — correctly separated | IS | .985+ |
| asd16 | F1 (merged with SES items) | EGA assigns to IS community (C3) — correctly separated | IS | .985+ |
| asd17 | F1 (merged with SES items) | EGA assigns to IS community (C3) — correctly separated | IS | .985+ |
| asd18 | F1 (merged with SES items) | EGA assigns to IS community (C3) — correctly separated | IS | .985+ |
| asd25 | F4 (IIS sub-cluster) | EGA merges with asd28–asd31 into single IIS community (C5) | IIS | .965 |
| asd26 | F4 (IIS sub-cluster) | EGA merges with asd28–asd31 into single IIS community (C5) | IIS | .965 |
| asd27 | F4 (IIS sub-cluster) | EGA merges with asd28–asd31 into single IIS community (C5) | IIS | .970 |

*Note.* For IS items (asd13–asd18): EFA cannot separate SES and IS in the 28-item rotation space, but EGA correctly recovers them as two distinct communities. For IIS items (asd25–asd27): EFA splits them from asd28–asd31 into a separate sub-factor, while EGA unifies all seven IIS items. In both cases, EGA's behavior is theoretically correct; the discordance reflects EFA's rotation-space limitation rather than true item misplacement. All nine discordant items showed high EGA bootstrap stability (≥ .965), confirming that EGA's assignments are structurally robust.

**PART C: Comparison of 31-Item and 28-Item Calibration Solutions**

**S10.19 Comparative Summary: 31-Item vs. 28-Item EFA and EGA Results**

**Table S10B.1.** Comparative summary of 31-item and 28-item calibration solutions across all key indicators

| Indicator | 31-Item (asd4/5/6 included) | 28-Item (asd4/5/6 removed) | Direction |
|---|---|---|---|
| Complete-case N | 273 | 276 | ≈ |
| KMO (overall) | .816 | .841 | ↑ improvement |
| EFA Primary Solution | | | |
| RMSEA (k = 5) | .124 [.118, .130] | .118 [.111, .125] | ↑ modest improvement |
| TLI (k = 5) | .731 | .778 | ↑ modest improvement |
| RMSR (k = 5) | .043 | .040 | ≈ minimal change |
| Theoretical–empirical alignment | 26/31 (83.9%) | 19/28 (67.9%) | ↓ apparent deterioration* |
| Cross-loading flagged items | 2/31 (6.5%) | 7/28 (25.0%) | ↓ apparent deterioration* |
| EFA bootstrap mean congruence | .859 (SD = .082) | .858 (SD = .076) | ≈ no meaningful change |
| EFA bootstrap ≥ .95 congruence | 18.5% | 15.5% | ≈ slight decrease |
| EGA (Walktrap) | | | |
| k detected | 5 | 5 | = unchanged |
| Theoretical block alignment | Partial (asd4/5/6/7 boundary) | Full (5/5 blocks exact) | ↑ marked improvement |
| EGA bootstrap P(k = 5) — nonparametric | .570 | .970 | ↑ marked improvement |
| EGA bootstrap P(k = 5) — parametric | .720 | .985 | ↑ marked improvement |
| Mean item stability | .915 | .994 | ↑ marked improvement |
| Minimum item stability | .390 (asd5, asd6) | .965 (asd25, asd26) | ↑ marked improvement |
| EFA–EGA item alignment | 83.9% (26/31) | 67.9% (19/28) | ↓ apparent decrease† |

*Note.* ↑ = improvement (28-item vs. 31-item); ↓ = deterioration; ≈ = negligible change. Asterisked (*) EFA deteriorations are rotation artifacts, not genuine structural failures: when the FRS block shrinks from 6 to 3 items, EFA's oblique rotation loses the balance needed to cleanly separate SES and IS — yet EGA continues to separate them correctly. The EGA evidence clearly dominates: structural stability improved substantially on every EGA indicator. Dagger (†) EFA–EGA alignment decrease reflects the same rotation artifact: EGA's communities are theoretically correct (100% block match), while EFA's five raw factors merge SES+IS and split IIS; the lower alignment number reflects EFA's rotation-space limitation, not an EGA problem.

The overarching conclusion from this comparison is that removing asd4, asd5, and asd6 produced clear, unambiguous improvements in EGA's structural stability and item-level consistency — confirming that these items were genuine sources of network-level structural instability. The EFA global fit also improved modestly. The apparent EFA alignment deterioration is a methodologically expected consequence of reducing a short block's item count in an oblique rotation framework, and is contradicted by the EGA evidence from the same data. This asymmetric pattern — clear EGA improvement, modest EFA global improvement, but EFA rotation-space deterioration — is exactly what motivated the pre-specified triangulation strategy: using EGA as an independent, rotation-free check on EFA findings.

*Transition to S11:* The calibration-based exploratory analyses (31-item: S10.1–S10.9; 28-item: S10.10–S10.18; comparison: S10.19) provided the evidence base for the item-reduction recommendation. Item removal decisions were made exclusively on calibration data; the validation subsample was not consulted at any stage before the confirmatory analyses reported in S11. Section S11 presents competitive CFA model tests, ESEM analyses, and all validation-subsample confirmatory results for both Model A (31 items) and Model B (28 items).

## S11. Confirmatory Analyses — Validation Subsample (N = 302)

### S11.1 Model Comparison: 31-Item Model A and 28-Item Model B

**Table S11.1.** Competing CFA model fit comparison: Model A (31 items) and Model B (28 items); validation subsample (N = 302)

| Model | k | $\chi^2$ | df | p | CFI | TLI | RMSEA | RMSEA [90% CI] | SRMR | BIC |
|---|---|---|---|---|---|---|---|---|---|---|
| **Model A (31 items)** | | | | | | | | | | |
| One-factor | 31 | 4375.71 | 434 | < .001 | .719 | .699 | .174 | [.169, .178] | .195 | 29002.57 |
| Correlated 5-factor | 31 | 1138.41 | 424 | < .001 | .949 | .944 | .075 | [.070, .080] | .075 | 27053.79 |
| Second-order | 31 | 1618.26 | 429 | < .001 | .915 | .908 | .096 | [.091, .101] | .111 | 27134.31 |
| Bifactor | 31 | 1612.69 | 403 | < .001 | .914 | .901 | .100 | [.095, .105] | .105 | 27009.73 |
| **Model B (28 items)** | | | | | | | | | | |
| One-factor | 28 | 3761.44 | 350 | < .001 | .732 | .710 | .180 | [.175, .185] | .194 | 26070.76 |
| Correlated 5-factor | 28 | 927.73 | 340 | < .001 | .954 | .949 | .076 | [.070, .082] | .072 | 24327.80 |
| Second-order | 28 | 1339.83 | 345 | < .001 | .922 | .914 | .098 | [.092, .103] | .109 | 24408.84 |
| Bifactor | 28 | 1302.65 | 322 | < .001 | .923 | .909 | .101 | [.095, .106] | .103 | 24432.63 |

*Note.* All models estimated with WLSMV and theta parameterization (lavaan; ordered = TRUE). BIC values for models with different numbers of items are not directly comparable across Model A and Model B. The correlated five-factor model had the lowest BIC within each set and was the only model meeting the CFI ≥ .95 criterion (Model B: CFI = .954). No model met the stringent RMSEA ≤ .06 criterion; this is attributable partly to the relatively low observation-to-parameter ratio (~2:1) and the known sensitivity of RMSEA to model complexity in ordinal CFA. No Heywood cases (negative residual variances) were observed in any model.

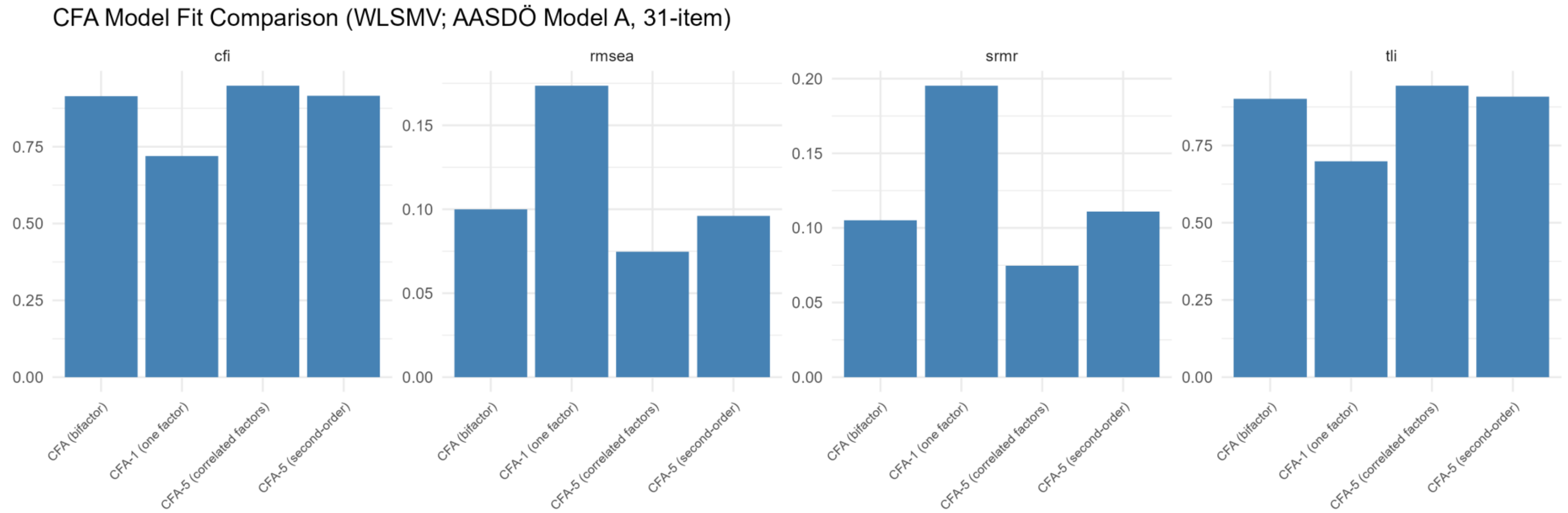


**Figure S11.1.** Four competing CFA models: CFI, TLI, RMSEA, and SRMR comparison (Model A, validation subsample).

*Note.* Bar charts comparing the four model types on each fit index. The correlated five-factor model (CFA-5) clearly outperforms the one-factor, second-order, and bifactor alternatives on CFI and SRMR.

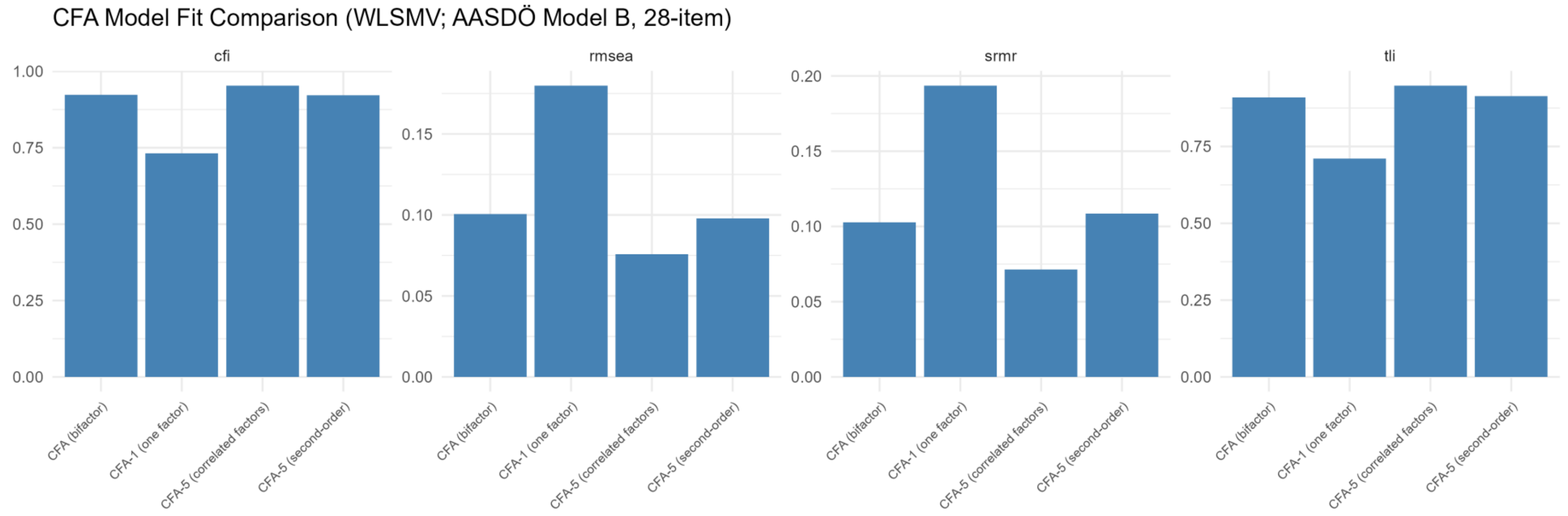


**Figure S11.2.** Four competing CFA models: CFI, TLI, RMSEA, and SRMR comparison (Model B, validation subsample).

*Note.* Same format as Figure S11.1 but for the 28-item set.

### S11.2 Primary CFA Model (5-Factor Correlated): Standardized Loadings

Standardized loadings for both Model A (31 items) and Model B (28 items) are reported below. The Model B column of this table constitutes the main manuscript's Table 3.

**Table S11.2.** Standardized CFA loadings: Model A (31 items) and Model B (28 items), correlated five-factor model; validation subsample (N = 302)

| Block | Item | Item content (English) | λ Model A | λ Model B |
|---|---|---|---|---|
| **Family–Relative Support** | | | | |
| | asd1 | My family was there for me when I needed them. | .898 | .925 |
| | asd2 | I relied on my family during difficult times. | .925 | .963 |
| | asd3 | My family supported me throughout my long-term recovery. | .850 | .868 |
| | asd4 | I shared my experiences with my relatives. | .845 | — |
| | asd5 | My relatives helped me. | .908 | — |

| | | | | |
|---|---|---|---|---|
| | asd6 | I stayed in contact with my relatives. | .877 | — |
| **Social Environment Support** | | | | |
| | asd7 | My friends were there for me. | .715 | .670 |
| | asd8 | My neighbors looked out for me. | .645 | .637 |
| | asd9 | I found people around me from whom I could seek support. | .769 | .783 |
| | asd10 | Our community acted in solidarity. | .772 | .775 |
| | asd11 | There was mutual aid in our neighborhood/village. | .774 | .789 |
| | asd12 | I connected with others who had experienced the disaster. | .732 | .740 |
| **Institutional Support** | | | | |
| | asd13 | Government institutions provided support. | .852 | .846 |
| | asd14 | NGOs and aid organizations provided help. | .790 | .792 |
| | asd15 | I was able to reach institutions when needed. | .863 | .864 |
| | asd16 | The services institutions provided met my needs. | .928 | .928 |
| | asd17 | I was able to access health services. | .851 | .851 |
| | asd18 | Institutions acted in a trustworthy manner. | .835 | .837 |
| **Emotional Support** | | | | |
| | asd19 | I was able to share my feelings. | .870 | .867 |
| | asd20 | There were people who listened to me. | .899 | .902 |
| | asd21 | I found people who understood what I was going through. | .906 | .907 |
| | asd22 | I encountered people who showed empathy. | .852 | .849 |
| | asd23 | I felt that there were people beside me. | .835 | .833 |
| | asd24 | I was helped to achieve emotional relief. | .790 | .791 |
| **Instrumental–Informational Support** | | | | |
| | asd25 | My shelter needs were met. | .766 | .761 |
| | asd26 | My basic needs such as food and clothing were met. | .808 | .806 |
| | asd27 | My transportation needs were met. | .759 | .760 |
| | asd28 | I was able to obtain financial support. | .762 | .761 |
| | asd29 | I obtained information about where to apply. | .847 | .847 |
| | asd30 | I was guided about the resources available. | .844 | .845 |
| | asd31 | I was able to obtain legal counseling. | .574 | .581 |

*Note.* λ = standardized WLSMV factor loading (std.all); all loadings $p < .001$. "—" = item not included in Model B. Items asd4, asd5, and asd6 were removed prior to confirmatory analyses on the basis of calibration-subsample exploratory evidence (see S10.4). Item asd31 consistently showed the lowest loading within its block across both models; see S11.3 and S11.5 for bifactor and residual correlation analyses.

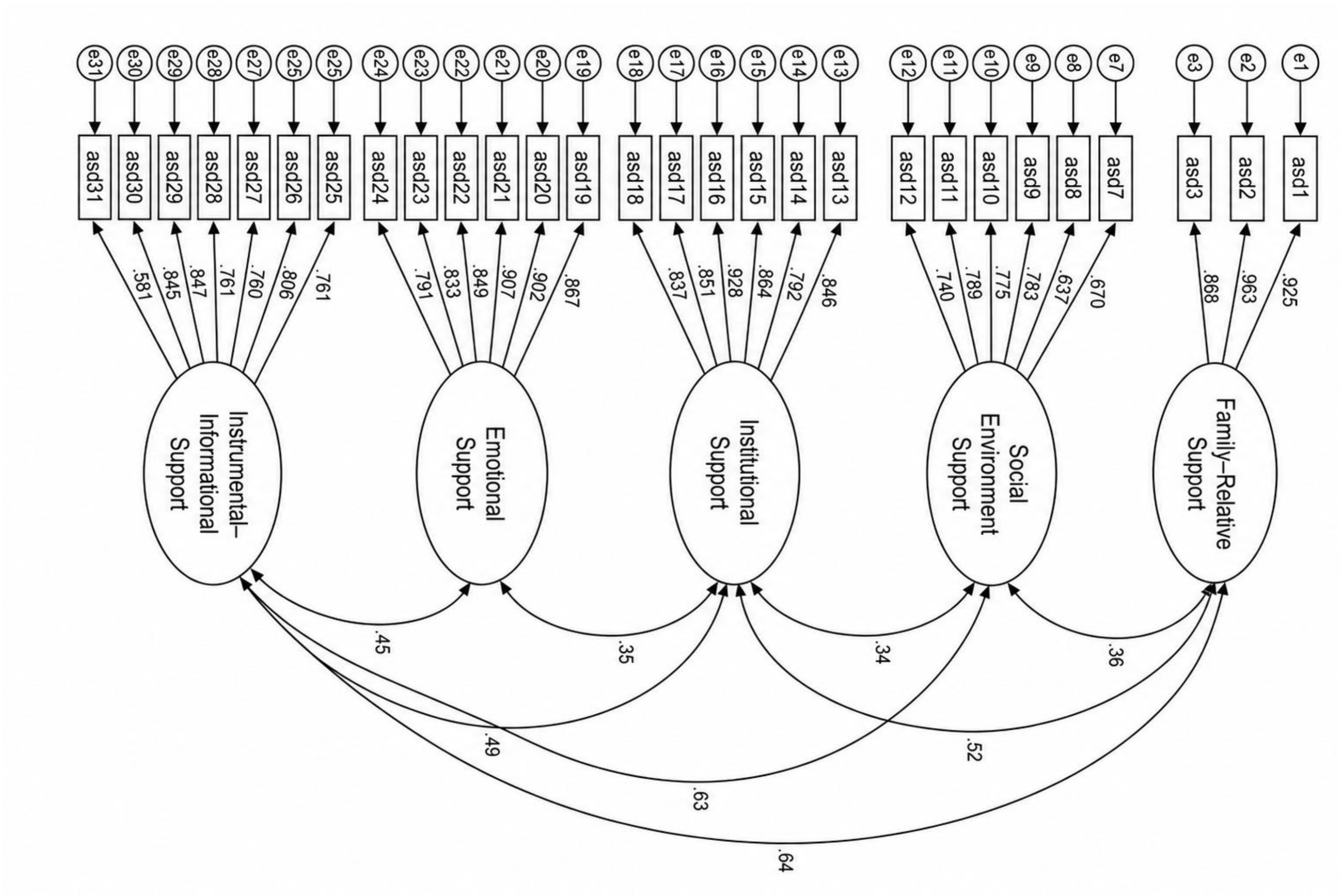


**Figure S11.3.** Path diagram: Model B correlated five-factor CFA (validation subsample, N = 302).

*Note.* Standardized loadings are shown on each path. Circles = latent factors (F1–F5); rectangles = observed indicators (asd1–asd31 minus asd4/5/6). Factor intercorrelations shown as curved double-headed arrows.

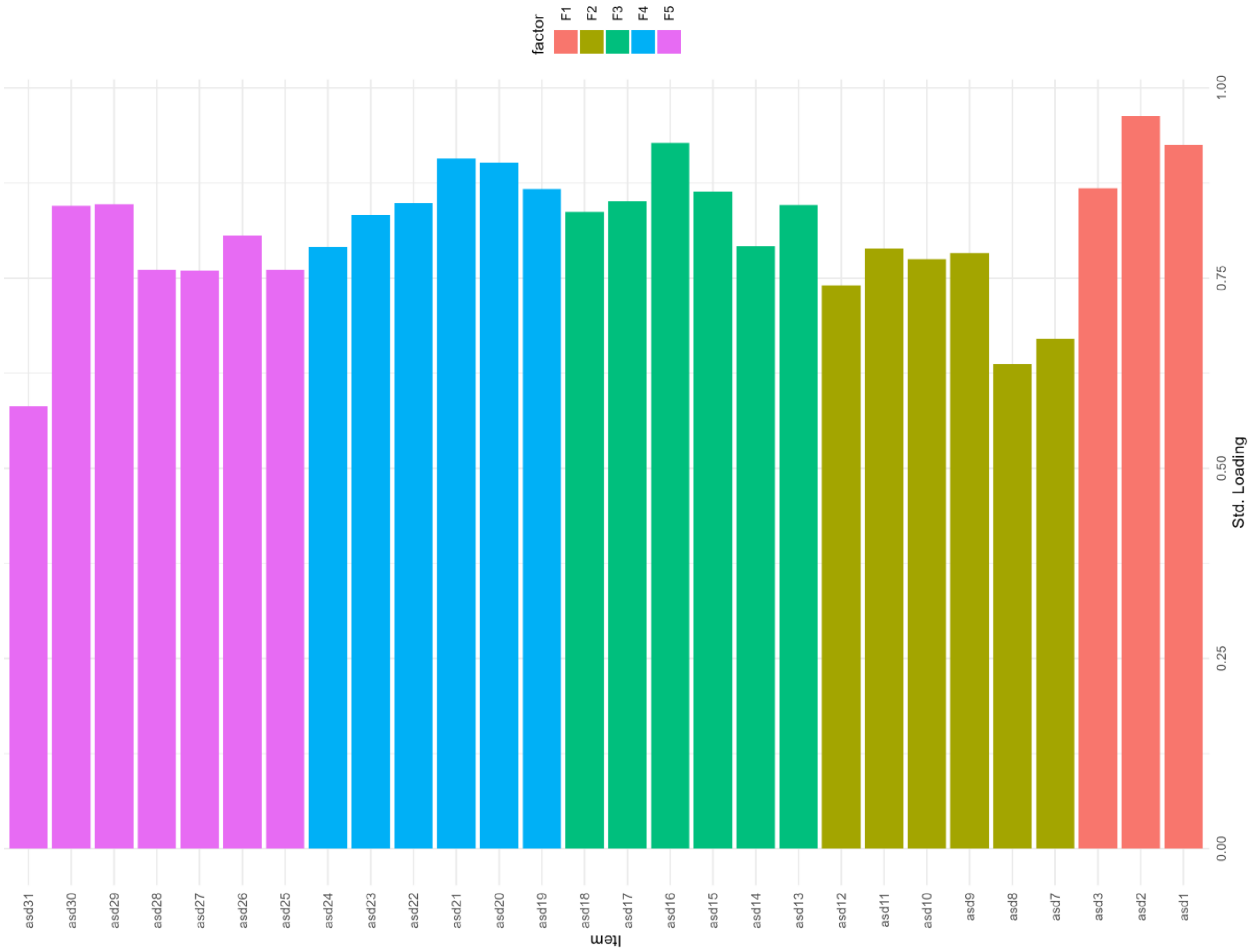


**Figure S11.4.** Standardized factor loadings by block (Model B, CFA-5; validation subsample).

*Note.* Items sorted from highest to lowest loading within each block. The consistent pattern of high loadings within blocks is evident, with asd31 as an outlier within the IIS block.

### S11.3 Factor Intercorrelations and Second-Order Structure

**Table S11.3.** CFA-5 factor intercorrelations: Model A (31 items) and Model B (28 items); validation subsample (N = 302)

| | FRS | SES | IS | ES | IIS |
|---|---|---|---|---|---|
| **FRS** | 1.000 | | | | |
| **SES** | .691 (.647) | 1.000 | | | |
| **IS** | .341 (.291) | .588 (.590) | 1.000 | | |
| **ES** | .493 (.500) | .600 (.601) | .429 (.430) | 1.000 | |
| **IIS** | .174 (.134) | .300 (.301) | .669 (.669) | .459 (.459) | 1.000 |

*Note.* Values outside parentheses = Model A (31 items); values in parentheses = Model B (28 items). The pattern of inter-factor relationships was highly similar across both models. The highest inter-factor correlations were SES–FRS (.691/.647) and IS–IIS (.669/.669); the lowest was FRS–IIS (.174/.134). Changes were confined to correlations involving FRS; IS, ES, IIS inter-correlations were virtually identical across models, confirming that item removal affected only F1-related relationships. FRS = Family–Relative Support; SES = Social Environment Support; IS = Institutional Support; ES = Emotional Support; IIS = Instrumental–Informational Support.

**Table S11.4.** Second-order model: standardized general factor loadings on primary factors; Model A and Model B

| Primary factor | λ (Model A) | λ (Model B) |
|---|---|---|
| FRS | .623 | .576 |
| SES | .840 | .773 |
| IS | .748 | .783 |
| ES | .693 | .682 |
| IIS | .628 | .669 |

*Note.* Second-order factor (GEN) is a single higher-order construct representing general post-disaster perceived social support. Removing FRS items asd4/5/6 led to FRS's general factor loading decreasing (.623 → .576) and IS's loading increasing (.748 → .783), reflecting redistributed variance in the second-order structure. All second-order loadings were statistically significant ($p < .001$).

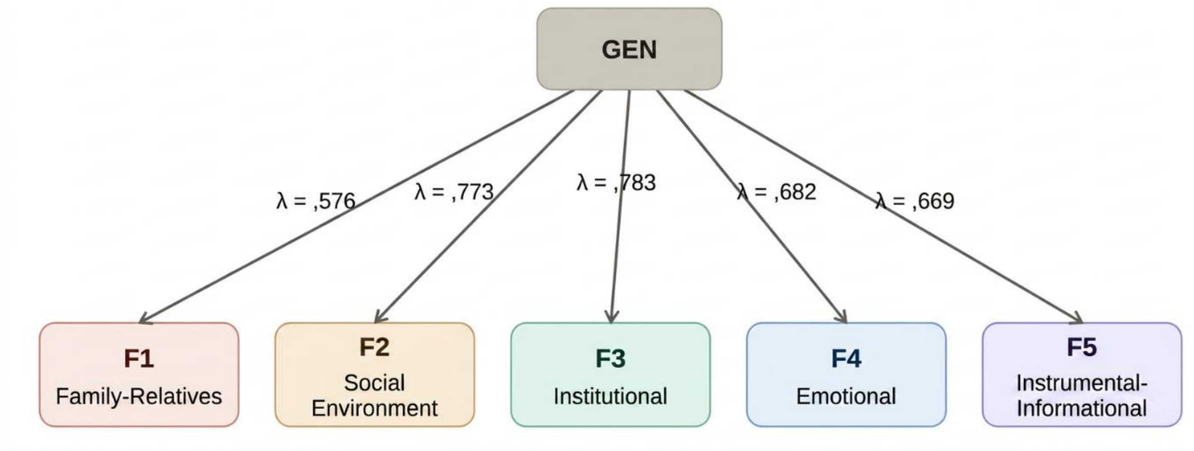


**Figure S11.5.** Second-order model path diagram: Model B (28 items; validation subsample).

*Note.* GEN = general perceived social support factor. Standardized second-order loadings shown on each path from GEN to F1–F5.

**S11.4 Bifactor Model Analysis**

**Table S11.5.** Bifactor model indicators: Model A (31 items) and Model B (28 items)

| Indicator | Model A | Model B |
|---|---|---|
| ECV (explained common variance) | .490 | .497 |
| ω_hierarchical | .938 | .932 |
| mean\|g\| | .570 | .572 |
| mean\|s\| | .575 | .572 |
| ω_specific: FRS | .845 | .792 |
| ω_specific: SES | .528 | .628 |
| ω_specific: IS | .749 | .715 |
| ω_specific: ES | .783 | .791 |
| ω_specific: IIS | .800 | .774 |

*Note.* ECV = ratio of general factor variance to total common variance; ω_h = hierarchical omega (reliability of the general factor). ECV ≈ .49 indicates the general factor explains approximately half of all common variance, providing support for a general dimension alongside specific factors. The decrease in ω_specific for FRS (Model A: .845 → Model B: .792) reflects the reduction of FRS from 6 to 3 items. The increase in ω_specific for SES (.528 → .628) is noteworthy: after removing the boundary items (asd4/5/6), SES-specific variance became more reliably estimated. FRS = Family–Relative Support; SES = Social Environment Support; IS = Institutional Support; ES = Emotional Support; IIS = Instrumental–Informational Support.

**Table S11.6.** Bifactor model: item-level general (g) and specific (s) standardized loadings (Model B; 28 items)

| **Item** | **Block** | **g** | **s** | **g²** | **s²** | **Unique variance** |
|---|---|---|---|---|---|---|
| asd1 | FRS | .534 | .759 | .285 | .576 | .140 |
| asd2 | FRS | .554 | .788 | .307 | .621 | .072 |
| asd3 | FRS | .512 | .694 | .263 | .482 | .256 |
| asd7 | SES | .532 | .329 | .283 | .108 | .609 |
| asd8 | SES | .449 | .545 | .201 | .297 | .502 |
| asd9 | SES | .613 | .453 | .376 | .206 | .419 |
| asd10 | SES | .584 | .558 | .342 | .311 | .347 |
| asd11 | SES | .617 | .491 | .380 | .241 | .379 |
| asd12 | SES | .587 | .427 | .345 | .183 | .472 |
| asd13 | IS | .691 | .458 | .477 | .210 | .313 |
| asd14 | IS | .563 | .595 | .317 | .353 | .329 |
| asd15 | IS | .630 | .623 | .397 | .388 | .215 |
| asd16 | IS | .736 | .552 | .541 | .305 | .154 |
| asd17 | IS | .691 | .482 | .477 | .232 | .291 |
| asd18 | IS | .641 | .544 | .411 | .296 | .292 |
| asd19 | ES | .576 | .652 | .332 | .425 | .243 |
| asd20 | ES | .569 | .721 | .324 | .520 | .156 |
| asd21 | ES | .605 | .679 | .366 | .461 | .172 |
| asd22 | ES | .590 | .610 | .348 | .371 | .280 |
| asd23 | ES | .604 | .558 | .365 | .311 | .324 |
| asd24 | ES | .588 | .501 | .345 | .251 | .404 |
| asd25 | IIS | .603 | .386 | .364 | .149 | .487 |
| asd26 | IIS | .629 | .437 | .396 | .191 | .413 |
| asd27 | IIS | .549 | .480 | .302 | .231 | .467 |
| asd28 | IIS | .461 | .649 | .213 | .421 | .366 |
| asd29 | IIS | .544 | .672 | .296 | .452 | .251 |
| asd30 | IIS | .537 | .673 | .288 | .454 | .258 |
| asd31 | IIS | .230 | .686 | .053 | .471 | .476 |

*Note.* g = general factor standardized loading; s = specific factor standardized loading. Item asd31 showed the lowest general factor loading (g = .230) across all 28 items, indicating that it shares relatively little variance with the general post-disaster perceived support dimension. Its specific loading (s = .686) was substantial, suggesting it measures something specific to the IIS block that is not shared with the general factor. FRS = Family–Relative Support; SES = Social Environment Support; IS = Institutional Support; ES = Emotional Support; IIS = Instrumental–Informational Support.

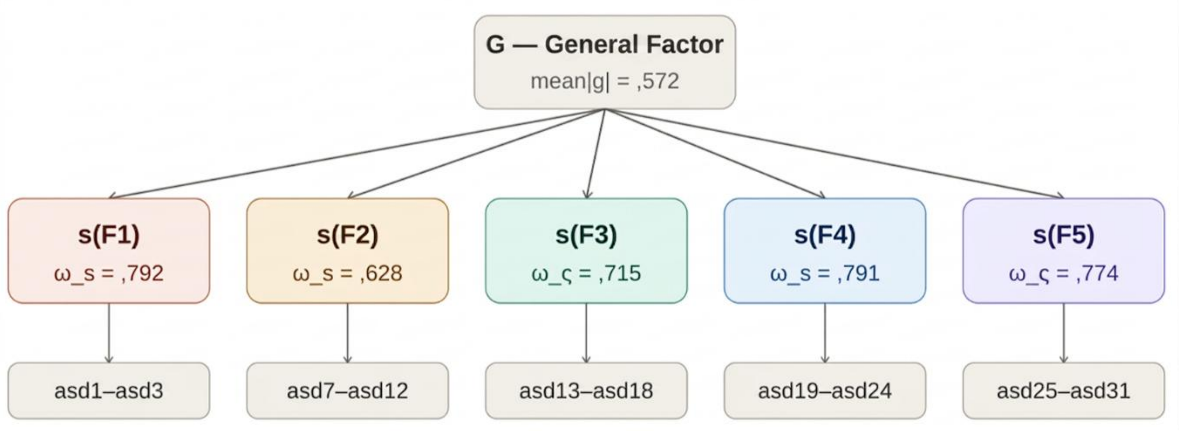


**Figure S11.6.** Bifactor model path diagram (Model B; 28 items; validation subsample).

*Note.* GEN = general factor; F1–F5 = specific factors. Each item is connected to both GEN and its specific factor. The low g loading for asd31 is visually apparent from the thin path from GEN to asd31.

### S11.5 Local Fit: Standardized Residual Correlations

**Table S11.7.** Highest 15 standardized residual correlations: Model B, CFA-5 (SAFE method; validation subsample, N = 302)

| Rank | Item i | Item j | Residual (*r*) | \|*r*\| |
|---|---|---|---|---|
| 1 | asd1 | asd31 | −.252 | .252 |
| 2 | asd23 | asd31 | −.248 | .248 |
| 3 | asd26 | asd31 | −.223 | .223 |
| 4 | asd2 | asd31 | −.218 | .218 |
| 5 | asd25 | asd30 | −.217 | .217 |
| 6 | asd28 | asd31 | +.207 | .207 |
| 7 | asd24 | asd25 | +.185 | .185 |
| 8 | asd23 | asd25 | +.181 | .181 |
| 9 | asd25 | asd29 | −.179 | .179 |
| 10 | asd10 | asd31 | −.177 | .177 |
| 11 | asd9 | asd14 | −.175 | .175 |
| 12 | asd9 | asd12 | −.175 | .175 |
| 13 | asd1 | asd25 | +.174 | .174 |
| 14 | asd20 | asd31 | −.173 | .173 |

| | | | | |
|---|---|---|---|---|
| 15 | asd24 | asd26 | +.166 | .166 |

*Note.* Residual r = observed polychoric correlation − model-implied correlation (SAFE method). Positive residuals indicate underestimation of the observed relationship; negative residuals indicate overestimation. Items asd1, asd2, asd23, asd26, asd28, asd10, asd20 all pair with asd31 among the highest residuals, providing independent corroborating evidence for asd31's anomalous behavior in the measurement model. Overall mean absolute residual = .056; median = .040; 43.9% of item pairs had |r| > .05; 17.7% had |r| > .10.

### S11.6 Discriminant Validity: HTMT Ratios

**Table S11.8.** HTMT ratios (polychoric; Model B)

| | **F1** | **F2** | **F3** | **F4** | **F5** |
|---|---|---|---|---|---|
| F1 | 1.000 | .663 | .289 | .503 | .182 |
| F2 | .663 | 1.000 | .594 | .615 | .312 |
| F3 | .289 | .594 | 1.000 | .433 | .696 |
| F4 | .503 | .615 | .433 | 1.000 | .459 |
| F5 | .182 | .312 | .696 | .459 | 1.000 |

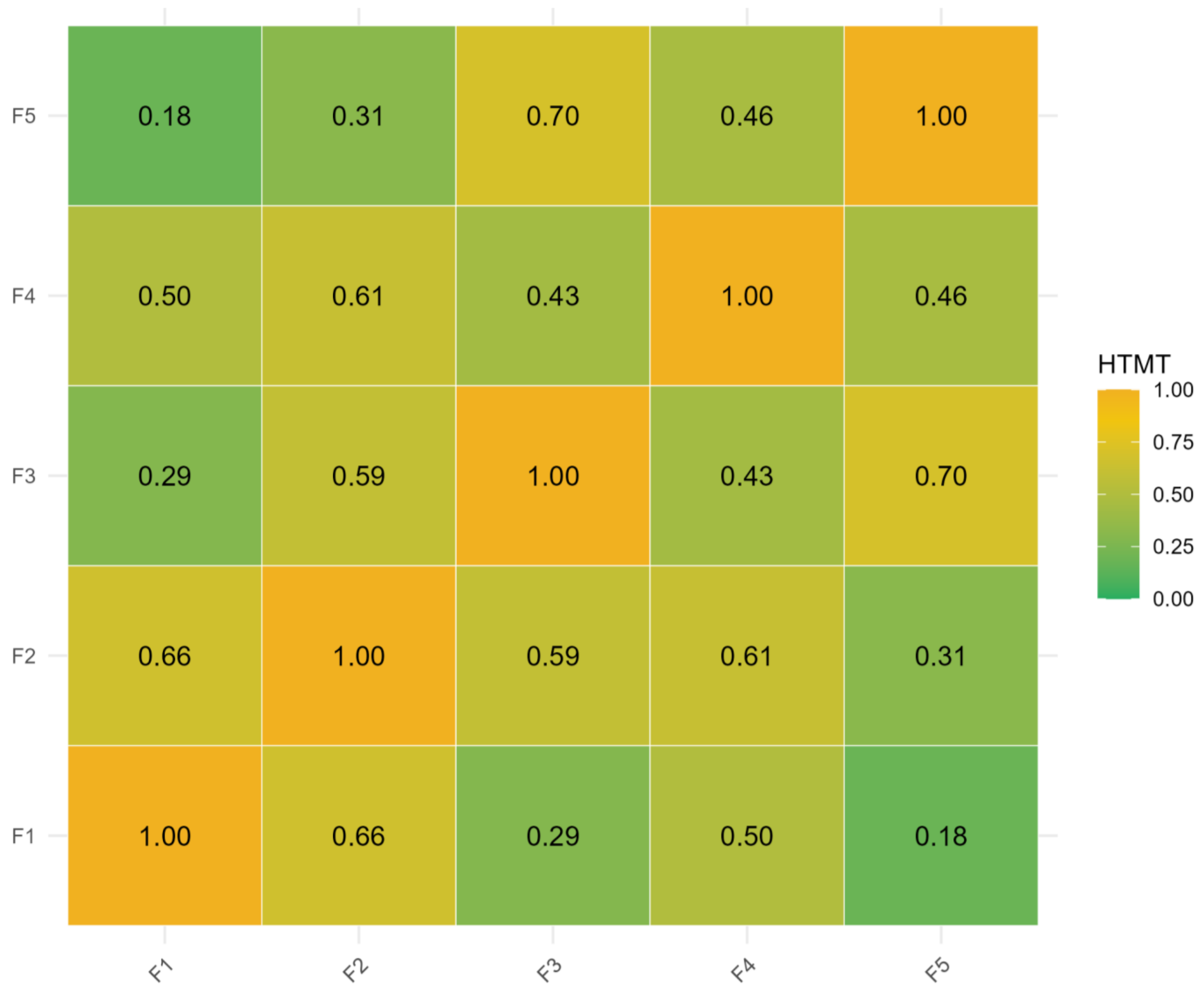


**Figure S11.7.** HTMT matrix heatmap (Model B).

**S11.7 ESEM-Target Analysis: Cross-Loading Profile**

**Table S11.9.** ESEM-Target vs. CFA-5 fit comparison: Model A and Model B (validation subsample)

| Model | k | $\chi^2$ | df | CFI | TLI | RMSEA [90% CI] | SRMR |
|---|---|---|---|---|---|---|---|
| CFA-5 (Model A) | 31 | 1138.41 | 424 | .949 | .944 | .075 [.070, .080] | .075 |
| ESEM-Target (Model A) | 31 | 984.75 | 320 | .953 | .931 | .083 [.077, .089] | .045 |
| CFA-5 (Model B) | 28 | 927.73 | 340 | .954 | .949 | .076 [.070, .082] | .072 |
| ESEM-Target (Model B) | 28 | 750.67 | 248 | .960 | .940 | .082 [.075, .089] | .040 |

*Note.* ESEM-Target used target rotation with item-specific theoretical factor as the target (free estimation) and near-zero targets for non-theoretical factors. The ESEM-Geomin sensitivity model was planned but could not be estimated due to a software constraint (lavaan "epsilon" argument error in this version); this is noted as a technical limitation. ESEM-Target improved CFI and SRMR over CFA-5 in both models; TLI and RMSEA were slightly worse, reflecting the parsimony penalty for additional cross-loading parameters.

**Table S11.10.** ESEM-Target cross-loading summary: Model A vs. Model B

| | Model A (31 items) | Model B (28 items) |
|---|---|---|
| Items with secondary loading ≥ .20 (n, %) | 17 (54.8%) | 11 (39.3%) |
| Items with secondary loading ≥ .30 (n, %) | 8 (25.8%) | 2 (7.1%) |
| Maximum secondary loading | .441 (asd12 → IS) | .378 (asd26 → SES) |

*Note.* Removing asd4, asd5, and asd6 reduced the cross-loading burden substantially (from 54.8% to 39.3% above the .20 threshold, and from 25.8% to 7.1% above .30), providing independent ESEM-based confirmation that these three items introduced cross-loading complexity into the measurement model. FRS = Family–Relative Support; SES = Social Environment Support; IS = Institutional Support; ES = Emotional Support; IIS = Instrumental–Informational Support.

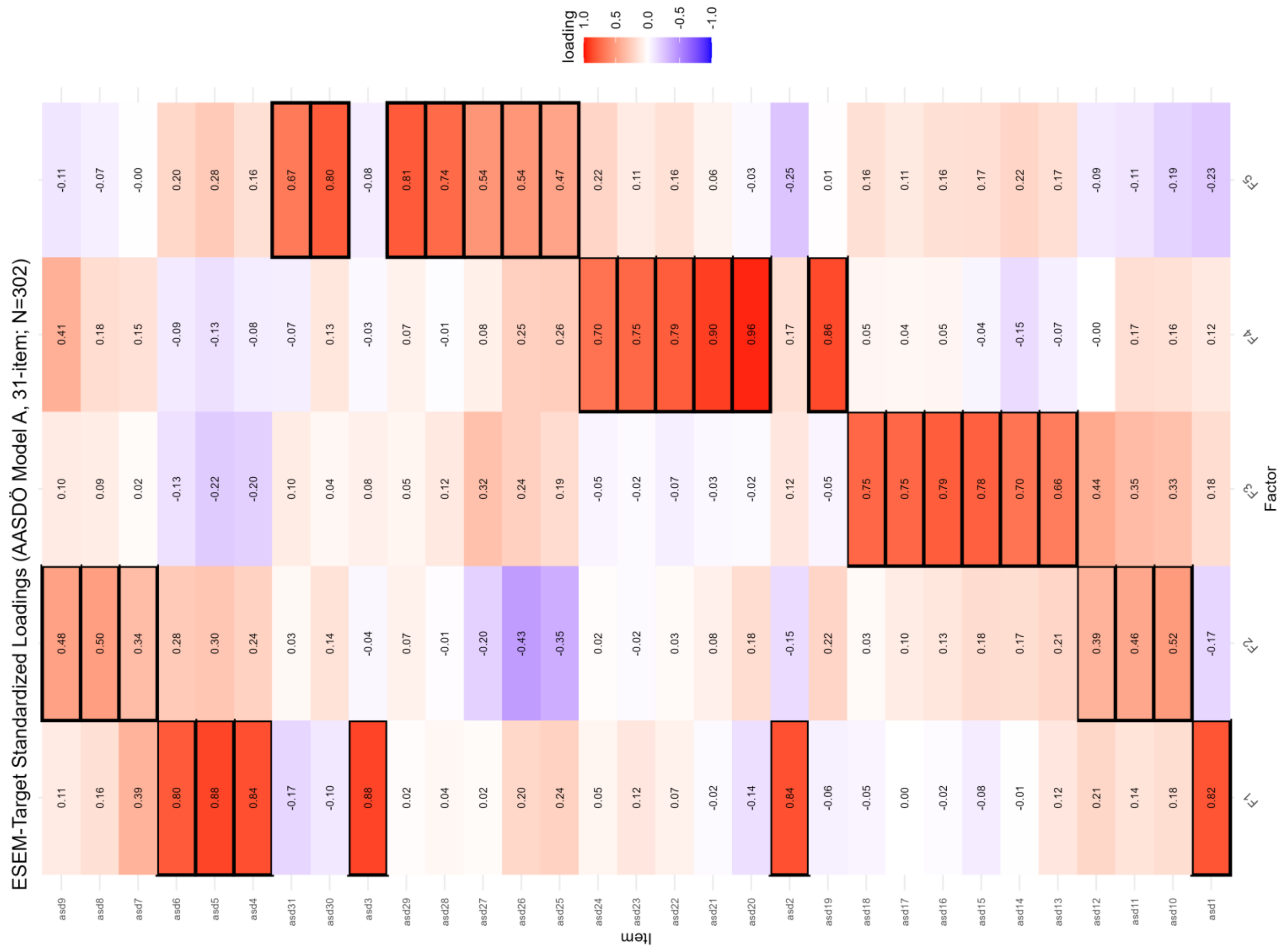


**Figure S11.8.** ESEM-Target loading heatmap (Model A; 31 items; validation subsample).

*Note.* Cells outlined in bold indicate the item's theoretical (target) factor. Colored values outside the diagonal blocks indicate cross-loadings.

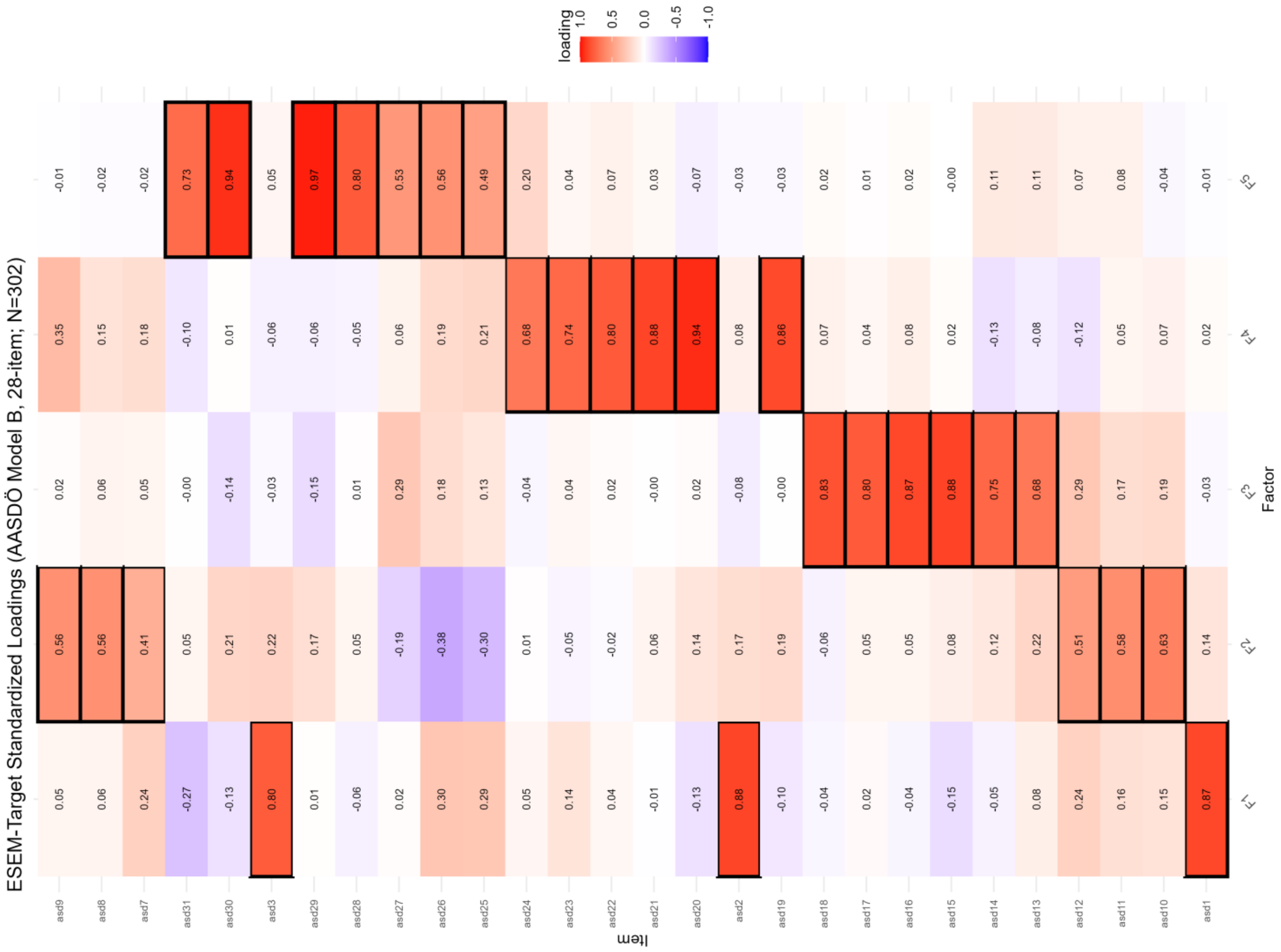


**Figure S11.9.** ESEM-Target loading heatmap (Model B; 28 items; validation subsample).

*Note.* Same format as Figure S11.8. Comparison with Figure S11.8 shows reduced off-diagonal coloration, reflecting fewer and smaller cross-loadings in the 28-item form.

### S11.8 Bootstrap CFA Stability

**Table S11.11.** Bootstrap CFA stability (B = 200): Model A and Model B (validation subsample)

| Model | Convergence rate (%) | CFI (M, SD) | TLI (M, SD) | RMSEA (M, SD) | SRMR (M, SD) |
|---|---|---|---|---|---|
| CFA-5 Model A | 100.0 | .941 (.009) | .939 (.010) | .082 (.004) | .086 (.005) |
| ESEM-Target Model A | 100.0 | .949 (.007) | .930 (.008) | .087 (.005) | .049 (.003) |
| CFA-5 Model B | 97.5 | .946 (.009) | .943 (.010) | .083 (.005) | .083 (.006) |
| ESEM-Target Model B | 99.0 | .956 (.007) | .939 (.007) | .088 (.006) | .045 (.003) |

*Note.* Bootstrap convergence was complete (100%) for Model A and nearly complete for Model B (CFA-5: 97.5%; ESEM-Target: 99.0%). The slight convergence reduction in Model B CFA-5 is attributable to the FRS block having only three items (just-identified at the factor level), which may produce estimation difficulties in some bootstrap resamples. Bootstrap fit distributions were narrow (small SDs), confirming that the observed single-run fit indices are representative of the resampling distribution.

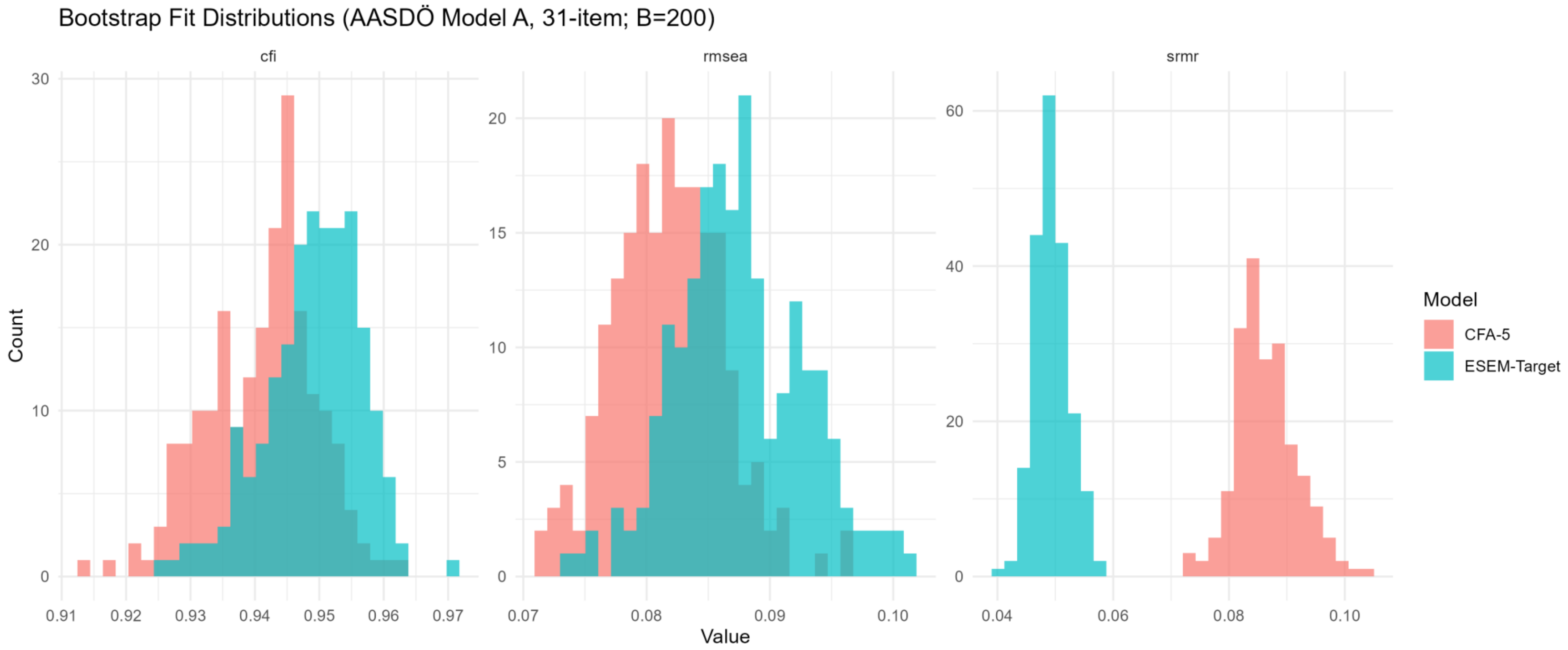


**Figure S11.10.** Bootstrap CFA and ESEM fit distributions (B = 200): CFI, RMSEA, and SRMR; Model A (validation subsample).

*Note.* Distributions of CFI (top), RMSEA (middle), and SRMR (bottom) across 200 bootstrap resamples for CFA-5 (left panels) and ESEM-Target (right panels).

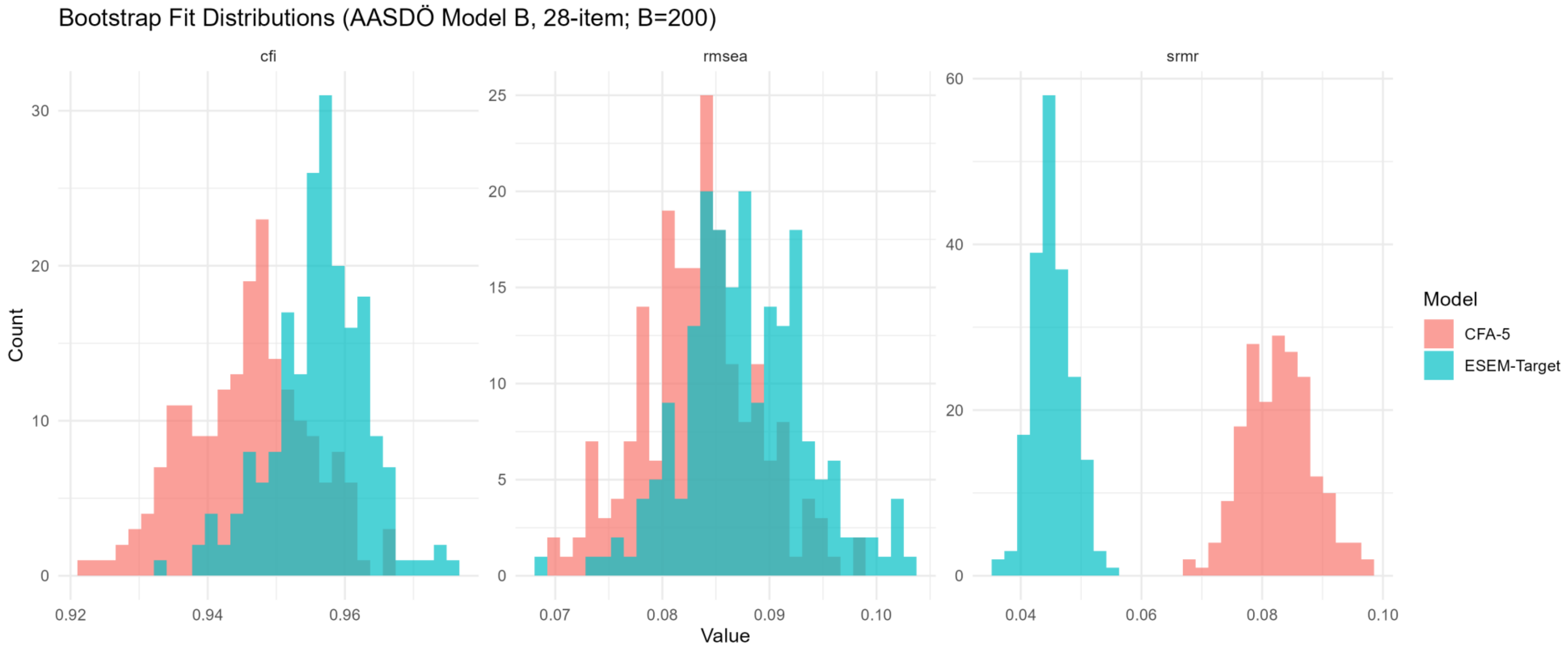


**Figure S11.11.** Bootstrap CFA and ESEM fit distributions (B = 200): CFI, RMSEA, and SRMR; Model B (validation subsample).

*Note.* Same format as Figure S11.10 but for the 28-item model. ESEM-Target shows a narrower, lower SRMR distribution compared to CFA-5.

## PART IV: MEASUREMENT INVARIANCE, RELIABILITY, VALIDITY, IRT, NETWORK PSYCHOMETRICS, AND COMMON METHOD VARIANCE

### S12. Measurement Invariance (PSSDS-28; Full Sample, N = 607)

### S12.1 Grouping Variables and A Priori Categorizations

**Table S12.1.** Group sizes for measurement invariance analyses (PSSDS-28; full sample, N = 607)

| Grouping variable | Group | n |
|---|---|---|
| **Age group** | 18–40 | 432 |
| | 41+ | 175 |
| **Gender** | Men | 333 |
| | Women | 274 |
| **Marital status** | Single | 290 |
| | Married | 271 |
| | Widowed/Divorced | 46 |
| **Education** | University and above | 233 |
| | High school | 201 |
| | Primary or below | 89 |
| | Middle school | 84 |
| **Employment** | Employed | 231 |
| | Student | 174 |
| | Homemaker | 109 |
| | Unemployed/Retired | 93 |
| **Monthly income** | No regular income | 204 |
| | Minimum wage | 127 |
| | 2× minimum wage | 119 |
| | 3× minimum wage | 62 |
| | Below minimum wage | 54 |
| | 4× minimum wage or above | 41 |
| **Loss type** | No major loss | 275 |
| | Bereavement | 139 |
| | Shelter loss | 76 |
| | Community loss | 46 |
| | Property loss | 37 |
| **Housing status** | Permanent housing | 351 |

| | | |
|---|---|---|
| | Rental | 147 |
| | Temporary housing | 57 |
| | With relatives/Other | 52 |
| **Psychological support** | No | 540 |
| | Yes | 67 |

*Note.* A priori category merging was applied before analysis: Age 61+ merged with 41–60 to form "41+"; Divorced merged with Widowed; Postgraduate merged with University; Unemployed merged with Retired. Housing: With relatives (n = 25) merged with Other (n = 27) = "With relatives/Other." Loss: Health loss (n = 9) was excluded a priori because n < 30 and no theoretically adequate merge target existed (cases set to NA); Livelihood loss (n = 25) was dropped under the same n < 30 rule. The analysed loss-type grouping therefore comprises N = 573. For monthly income, loss type, and housing, configural models failed due to empty response categories in small subgroups (see S12.3).

### S12.2 Invariance Results (Six Testable Variables)

All analyses used WLSMV with theta parameterization; prespecified Δ-fit decision rules were: for metric (metric − configural): ΔCFI ≥ −.010, ΔRMSEA ≤ +.015, ΔSRMR ≤ +.030; for scalar (scalar − metric): ΔCFI ≥ −.010, ΔRMSEA ≤ +.015, ΔSRMR ≤ +.010.

**Table S12.2.** Measurement invariance: model fit by step and grouping variable

| Grouping variable | Step | $\chi^2$ | df | CFI | TLI | RMSEA | RMSEA 90% CI | SRMR |
|---|---|---|---|---|---|---|---|---|
| **Age group** | Configural | 1697.79 | 680 | .961 | .956 | .070 | [.066, .075] | .067 |
| | Metric | 1621.59 | 703 | .965 | .962 | .066 | [.062, .070] | .069 |
| | Scalar | 1776.05 | 759 | .961 | .961 | .067 | [.063, .071] | .067 |
| **Gender** | Configural | 1709.36 | 680 | .961 | .957 | .071 | [.067, .075] | .067 |
| | Metric | 1622.47 | 703 | .965 | .963 | .066 | [.062, .070] | .069 |
| | Scalar | 1812.39 | 759 | .960 | .960 | .068 | [.064, .072] | .067 |
| **Marital status** | Configural | 1938.99 | 1020 | .966 | .962 | .067 | [.062, .071] | .073 |
| | Metric | 1895.11 | 1066 | .969 | .967 | .062 | [.058, .067] | .075 |
| | Scalar | 2067.96 | 1178 | .967 | .968 | .061 | [.057, .066] | .073 |
| **Education** | Configural | 2399.80 | 1360 | .963 | .958 | .071 | [.067, .076] | .080 |
| | Metric | 2413.53 | 1429 | .965 | .963 | .068 | [.063, .072] | .084 |
| | Scalar | 2658.57 | 1597 | .962 | .964 | .066 | [.062, .071] | .080 |
| **Employment** | Configural | 2466.03 | 1360 | .962 | .957 | .073 | [.069, .078] | .080 |
| | Metric | 2369.09 | 1429 | .967 | .966 | .066 | [.061, .071] | .082 |
| | Scalar | 2685.68 | 1597 | .962 | .964 | .067 | [.063, .072] | .080 |
| **Psychological support** | Configural | 1504.83 | 680 | .966 | .962 | .063 | [.059, .068] | .067 |
| | Metric | 1422.23 | 703 | .971 | .968 | .058 | [.054, .063] | .069 |
| | Scalar | 1552.61 | 759 | .967 | .968 | .059 | [.055, .063] | .067 |

*Note.* WLSMV with theta parameterization. Configural = same factor structure, no equality constraints; Metric = equal loadings; Scalar = equal loadings and thresholds.

**Table S12.3.** Nested Δ-fit changes and invariance decisions

| Grouping variable | Comparison | ΔCFI | ΔTLI | ΔRMSEA | ΔSRMR | Decision |
|---|---|---|---|---|---|---|
| Age group | Metric − Configural | +.004 | +.006 | −.005 | +.001 | Supported ✓ |
| | Scalar − Metric | −.004 | −.001 | +.001 | −.001 | Supported ✓ |
| Gender | Metric − Configural | +.004 | +.006 | −.005 | +.001 | Supported ✓ |
| | Scalar − Metric | −.005 | −.002 | +.002 | −.001 | Supported ✓ |
| Marital status | Metric − Configural | +.003 | +.005 | −.005 | +.002 | Supported ✓ |
| | Scalar − Metric | −.002 | +.001 | −.001 | −.002 | Supported ✓ |
| Education | Metric − Configural | +.002 | +.004 | −.004 | +.004 | Supported ✓ |
| | Scalar − Metric | −.003 | +.001 | −.001 | −.004 | Supported ✓ |
| Employment | Metric − Configural | +.006 | +.008 | −.007 | +.002 | Supported ✓ |
| | Scalar − Metric | −.005 | −.001 | +.001 | −.002 | Supported ✓ |
| Psychological support | Metric − Configural | +.004 | +.006 | −.005 | +.002 | Supported ✓ |
| | Scalar − Metric | −.003 | −.001 | +.001 | −.002 | Supported ✓ |

*Note.* All six variables supported metric and scalar/strong invariance under prespecified practical Δ-fit criteria. The largest deterioration observed was employment/scalar − metric: ΔCFI = −.005, ΔRMSEA = +.001, ΔSRMR = −.002 — well within all thresholds. Positive ΔCFI indicates improvement (i.e., the more constrained model fit better); negative ΔRMSEA indicates improvement.

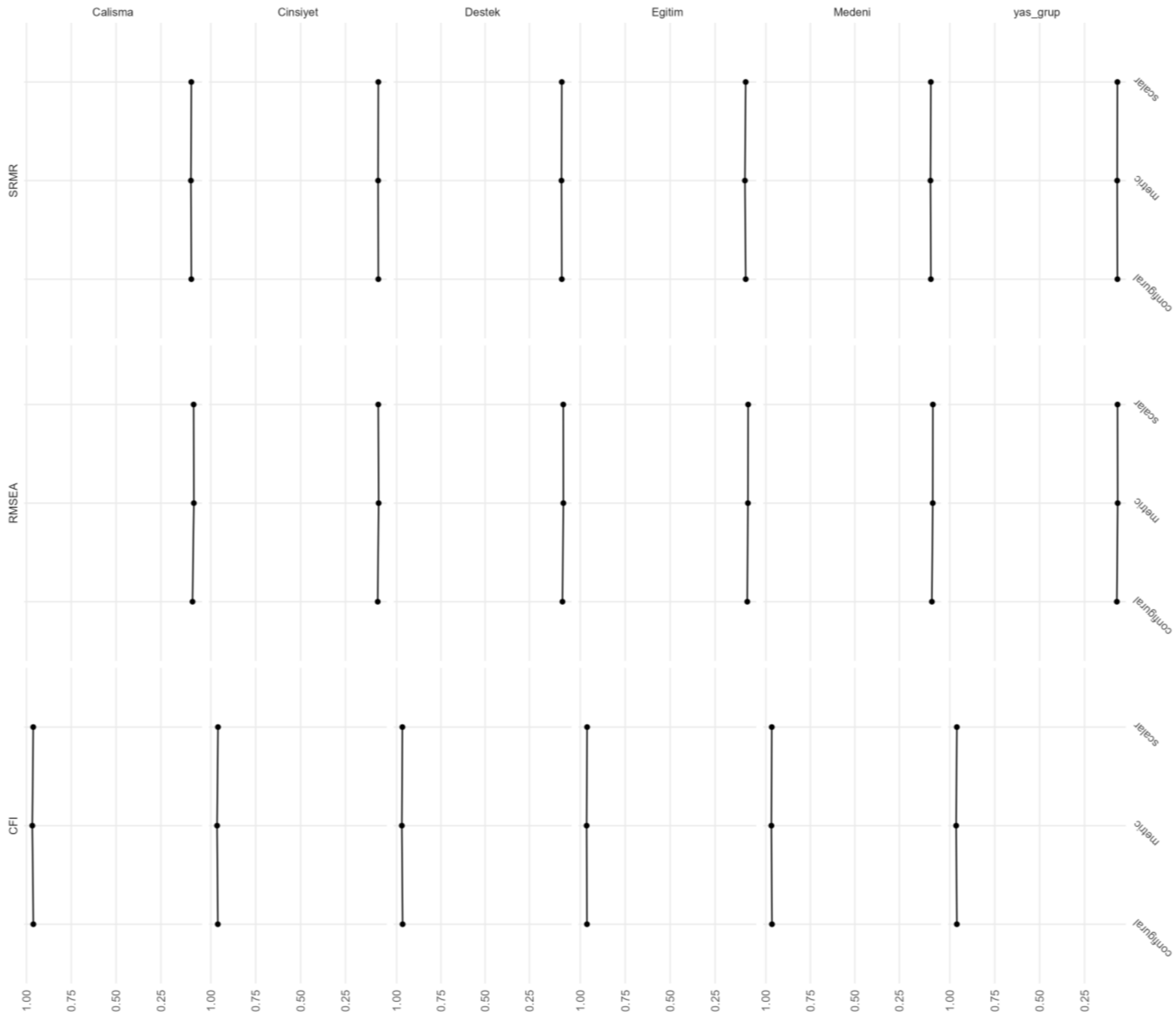


**Figure S12.1.** Measurement invariance fit traces (CFI, RMSEA, SRMR) across configural → metric → scalar steps, by grouping variable.

*Note.* Lines connect fit index values at each step. Stability across steps is visible for all six tested variables.

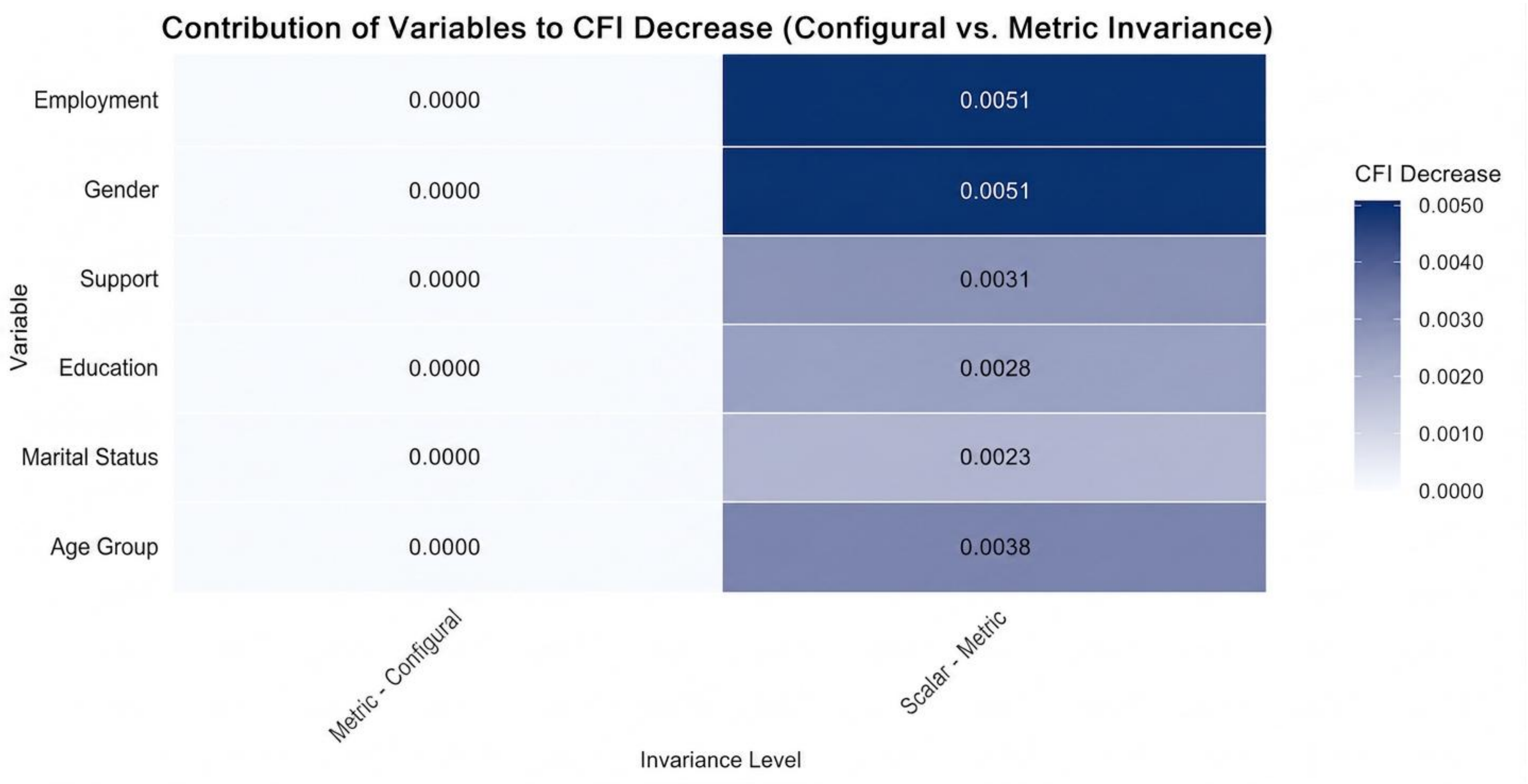


**Figure S12.2.** CFI deterioration heatmap by grouping variable × invariance comparison.

*Note.* Values show max(0, −ΔCFI); improvements are shown as 0. The largest CFI deterioration (employment, scalar − metric = .005) is visible in the heatmap.

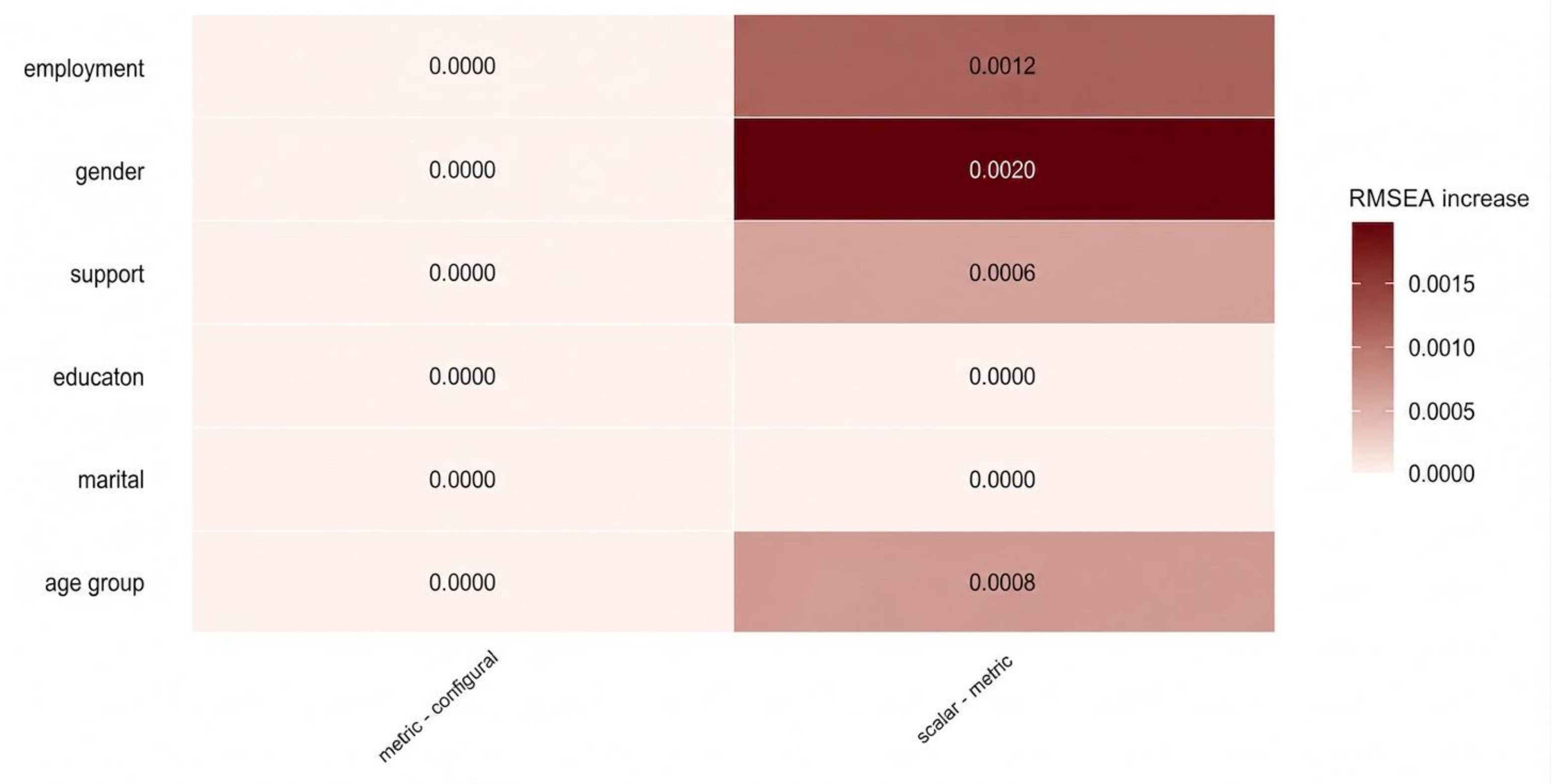


**Figure S12.3.** RMSEA deterioration heatmap by grouping variable × invariance comparison.

*Note.* Values show max(0, ΔRMSEA); improvements shown as 0.

**S12.3 Variables Where Invariance Testing Was Not Feasible**

For monthly income, loss type, and housing status, the configural model was not estimable because small subgroups produced empty response categories in specific item-by-category combinations (EMPTY_CATEGORY_FAIL in lavaan's lav_samplestats_step1()). Specific empty cells identified:

- **Monthly income:** "3× minimum wage" group (n = 62): asd22 category 1 not observed; "4× minimum wage +" group (n = 41): asd1 and asd2 category 2 not observed.
- **Loss type:** "Property loss" group (n = 37): asd9 and asd31 category 3 not observed; asd20 and asd21 category 2 not observed.

- **Housing status:** "With relatives/Other" group (n = 52): asd18 category 2 not observed.

These are sampling-distribution constraints rather than scale deficiencies. Measurement invariance for these variables will require larger or more category-balanced subsamples in future studies.

### S13. Reliability Analyses (PSSDS-28; Full Sample, N = 607)

#### S13.1 Overview

Reliability of the PSSDS-28 (Model B, five-factor structure: F1-Family–Relative Support, F2-Social Environment Support, F3-Institutional Support, F4-Emotional Support, F5-Instrumental–Informational Support) was evaluated in the full sample (N = 607) using a multi-source framework. Because PSSDS-28 is supported as a five-factor instrument, internal consistency was reported primarily at the factor level (F1–F5). CFA-based composite reliability (CR) and average variance extracted (AVE) were presented as model-based factor-level indices. Test–retest reliability was examined using retest data collected approximately 21 days after initial administration (n = 89).

Estimation approach: ordinal alpha (Cronbach alpha computed from the polychoric correlation matrix, with 95% bootstrap confidence intervals, B = 500), omega-total (polychoric-based McDonald omega), corrected item–total correlation (r.drop, polychoric-based), and alpha-if-item-deleted. All analyses were performed without imputation, using observed responses and methods appropriate for ordinal-categorical indicators.

#### S13.2 Internal Consistency — PSSDS-28 Factors (Ordinal/Polychoric)

**Table S13.1.** PSSDS-28 factors: internal consistency (ordinal α with bootstrap CI; ω_t; supporting indices)

| Factor | k | Ordinal α | α CI-lower | α CI-upper | ω_t | Mean IIC | r.drop min | r.drop max |
|---|---|---|---|---|---|---|---|---|
| FRS | 3 | .949 | .935 | .962 | .950 | .860 | .858 | .927 |
| SES | 6 | .852 | .828 | .875 | .854 | .490 | .551 | .714 |
| IS | 6 | .937 | .926 | .946 | .938 | .714 | .774 | .874 |
| ES | 6 | .939 | .927 | .948 | .939 | .720 | .768 | .870 |
| IIS | 7 | .900 | .884 | .913 | .901 | .561 | .605 | .761 |

*Note.* α and ω_t computed from polychoric correlations. Bootstrap valid replications = 500/500 for all factors (target B = 500). Mean IIC = mean polychoric inter-item correlation within the factor. FRS = Family–Relative Support; SES = Social Environment Support; IS = Institutional Support; ES = Emotional Support; IIS = Instrumental–Informational Support.

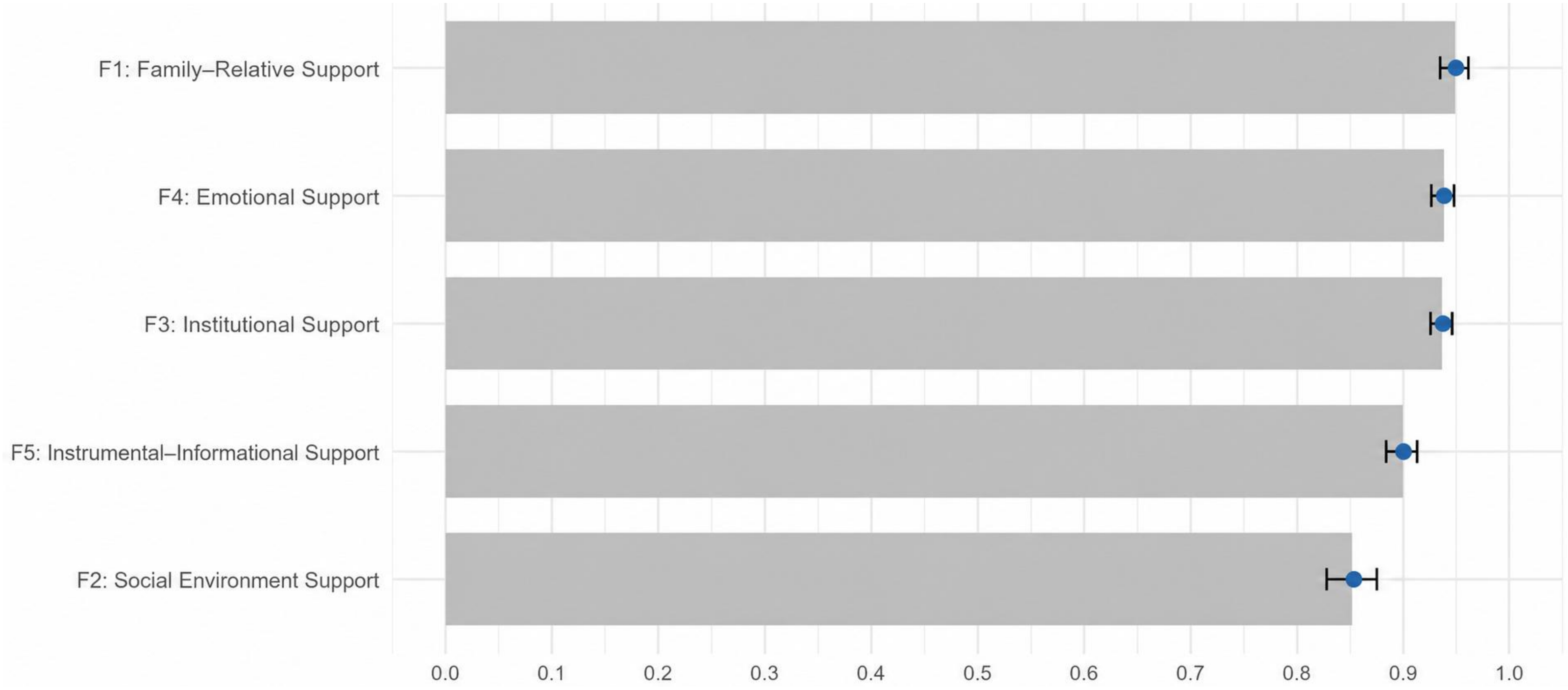


**Figure S13.1.** PSSDS-28 factors: ordinal alpha (95% bootstrap CI) and omega-total.

*Note.* Bars = ordinal alpha (polychoric) with 95% bootstrap CI (B = 500); blue dots = omega-total.

All five factors demonstrated high internal consistency by the ordinal alpha criterion ($\alpha = .852–.949$), supported by 95% bootstrap confidence intervals. Omega-total values closely paralleled alpha in every factor ($\omega\_t = .854–.950$), indicating that reliability estimation was not sensitive to the choice of coefficient. FRS, despite its short three-item structure, showed the highest internal consistency ($\alpha = .949$), consistent with its highest mean inter-item correlation ($r = .860$). SES showed the lowest (but still acceptably high) internal consistency ($\alpha = .852$) and the lowest mean inter-item correlation ($r = .490$) among the five factors. Corrected item–total correlations (r.drop) were uniformly high across all factors (minimum values .551–.858; maximum values .714–.927).

**Table S13.2.** PSSDS-28 item analysis (M, SD, corrected item–total r, alpha-if-deleted)

| Item | M | SD | CITC | α (if deleted) | Block |
|---|---|---|---|---|---|
| asd1 | 4.243 | 1.169 | .892 | .925 | FRS |
| asd2 | 4.198 | 1.179 | .927 | .898 | FRS |
| asd3 | 4.087 | 1.225 | .858 | .951 | FRS |
| asd7 | 3.748 | 1.296 | .551 | .844 | SES |
| asd8 | 3.565 | 1.320 | .636 | .828 | SES |
| asd9 | 3.838 | 1.218 | .693 | .817 | SES |
| asd10 | 3.842 | 1.215 | .714 | .813 | SES |
| asd11 | 3.768 | 1.272 | .658 | .824 | SES |
| asd12 | 3.847 | 1.259 | .575 | .839 | SES |
| asd13 | 3.443 | 1.376 | .774 | .931 | IS |
| asd14 | 3.392 | 1.326 | .791 | .929 | IS |
| asd15 | 3.137 | 1.369 | .833 | .923 | IS |
| asd16 | 3.152 | 1.374 | .874 | .918 | IS |
| asd17 | 3.316 | 1.409 | .794 | .928 | IS |
| asd18 | 3.321 | 1.433 | .812 | .926 | IS |
| asd19 | 3.650 | 1.377 | .812 | .928 | ES |
| asd20 | 3.805 | 1.215 | .844 | .924 | ES |
| asd21 | 3.802 | 1.239 | .870 | .921 | ES |
| asd22 | 3.847 | 1.165 | .806 | .929 | ES |
| asd23 | 3.871 | 1.153 | .803 | .929 | ES |
| asd24 | 3.690 | 1.213 | .768 | .934 | ES |
| asd25 | 3.434 | 1.393 | .605 | .896 | IIS |
| asd26 | 3.343 | 1.362 | .674 | .888 | IIS |
| asd27 | 2.960 | 1.431 | .750 | .879 | IIS |
| asd28 | 2.772 | 1.407 | .758 | .879 | IIS |
| asd29 | 3.015 | 1.373 | .761 | .878 | IIS |
| asd30 | 2.935 | 1.379 | .756 | .879 | IIS |
| asd31 | 2.460 | 1.432 | .633 | .893 | IIS |

*Note.* M/SD computed descriptively from numerical coding. CITC and alpha-if-deleted based on polychoric correlations (ordinal approach). Within blocks, only asd3 showed an alpha-if-deleted value above its block coefficient (.951 against an FRS alpha of .949, a margin of +.002 arising from the just-identified three-item structure); for asd31 the value was .893 against an IIS alpha of .900. Neither margin justifies deletion, and all 28 items were retained. Table S9.2 reports

alpha-if-deleted against the 31-item administered pool rather than against the block, which is why asd31 is flagged there and asd3 is not. FRS = Family–Relative Support; SES = Social Environment Support; IS = Institutional Support; ES = Emotional Support; IIS = Instrumental–Informational Support.

**S13.3 CFA-Based Reliability — Composite Reliability (CR) and AVE**

CR and AVE were computed from the full-sample ordinal CFA-5 model (WLSMV, theta parameterization).

**Table S13.3.** PSSDS-28 factors: composite reliability (CR) and AVE from ordinal CFA-5

| Factor | k | CR | AVE | Min. loading | Max. loading |
|---|---|---|---|---|---|
| FRS: Family–Relative Support | 3 | .950 | .863 | .881 | .981 |
| SES: Social Environment Support | 6 | .865 | .518 | .663 | .778 |
| IS: Institutional Support | 6 | .940 | .725 | .810 | .913 |
| ES: Emotional Support | 6 | .943 | .735 | .813 | .899 |
| IIS: Instrumental–Informational Support | 7 | .915 | .607 | .654 | .861 |

*Note.* CR and AVE computed from the full-sample ordinal CFA-5 model (WLSMV; theta parameterization; std.lv = TRUE; missing = pairwise). These are the CR values reported in the main text; Table S18.9 reports CR from a separately specified construct-replicability model and differs in the third decimal (SES .867 there versus .865 here). All factors exceeded the CR > .80 threshold. AVE exceeded the .50 criterion for all factors; SES showed the lowest AVE (.518), consistent with its broader content domain. Min./max. loadings are from this full-sample model and therefore differ from the validation-subsample loadings in Table S11.2 (e.g., FRS .881–.981 here versus .868–.963 there).

**S13.4 Test–Retest Reliability (T1–T2; PSSDS-28 Factors)**

Retest data were collected approximately 21 days after initial administration (M = 20.8 days, SD = 2.3; n = 89).

**Table S13.4.** PSSDS-28 test–retest reliability (factor scores)

| Factor | ICC (2,1) | ICC CI | Pearson r | r CI | SEM | MDC_95 | mean_diff | bias p |
|---|---|---|---|---|---|---|---|---|
| FRS | .542 | [.225, .726] | .627 | [.482, .739] | .727 | 2.015 | −.588 | < .001 |
| SES | .607 | [.316, .767] | .689 | [.561, .785] | .454 | 1.259 | −.348 | < .001 |
| IS | .859 | [.789, .907] | .880 | [.823, .920] | .318 | .880 | −.114 | .016 |
| ES | .909 | [.865, .940] | .914 | [.871, .942] | .241 | .668 | −.025 | .498 |
| IIS | .892 | [.840, .927] | .902 | [.855, .935] | .251 | .696 | −.030 | .434 |

*Note.* ICC = ICC(2,1) two-way random effects, absolute agreement. SEM and MDC_95 derived from ICC. mean_diff = T2 − T1. No imputation applied; factor scores computed as available-item means (with minimum-item rule). N = 89 (retest-eligible subsample). IS, ES, and IIS demonstrated high temporal stability (ICC = .859–.909); FRS and SES demonstrated moderate stability (ICC = .542 and .607). FRS, SES, and IS showed statistically significant small-to-moderate negative mean differences (T2 < T1; p ≤ .016); ES and IIS showed no significant difference. The SEM and MDC_95 values in this table are retest-based and are the appropriate reference for change interpretation; the alpha-based values in Table S18.5 describe the precision of a single administration.

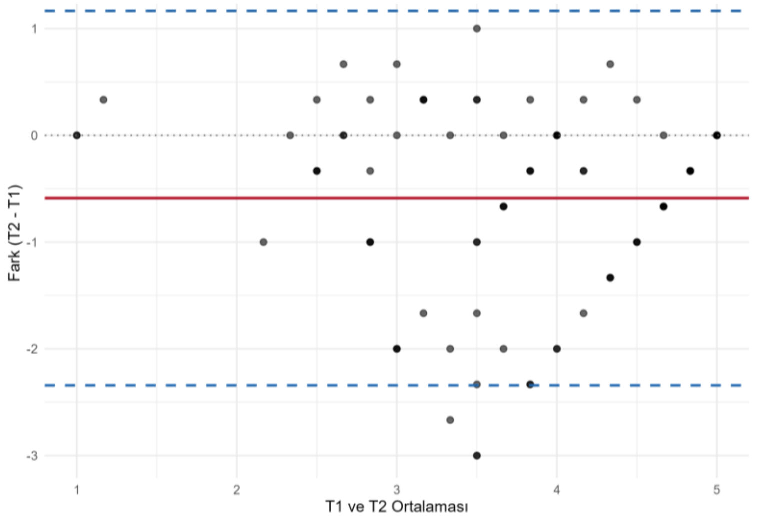


**Figure S13.2.** Bland–Altman plot: FRS (Family–Relative Support).

*Note.* Solid line = mean difference (T2 − T1); dashed lines = 95% limits of agreement (±1.96 SD).

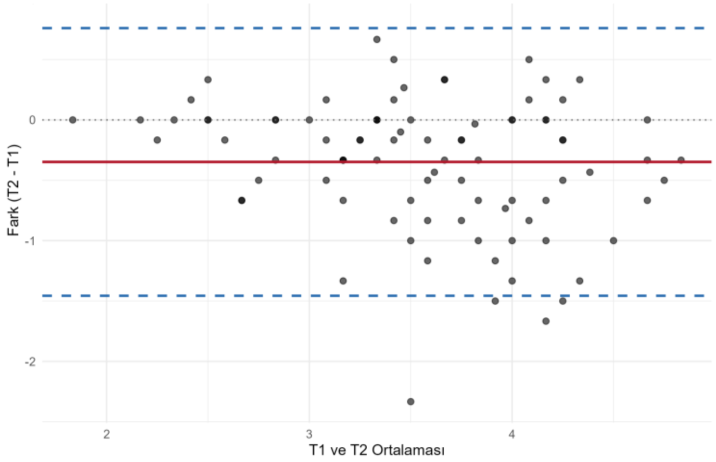


**Figure S13.3.** Bland–Altman plot: SES (Social Environment Support).

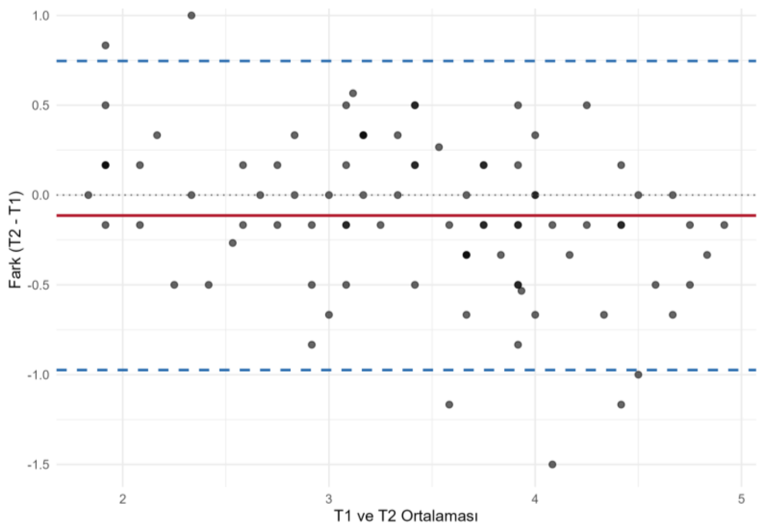


**Figure S13.4.** Bland–Altman plot: IS (Institutional Support).

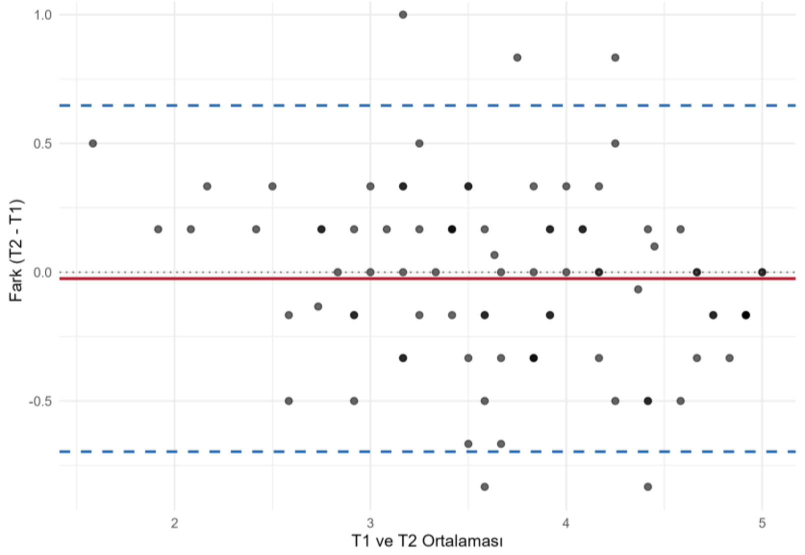


**Figure S13.5.** Bland–Altman plot: ES (Emotional Support).

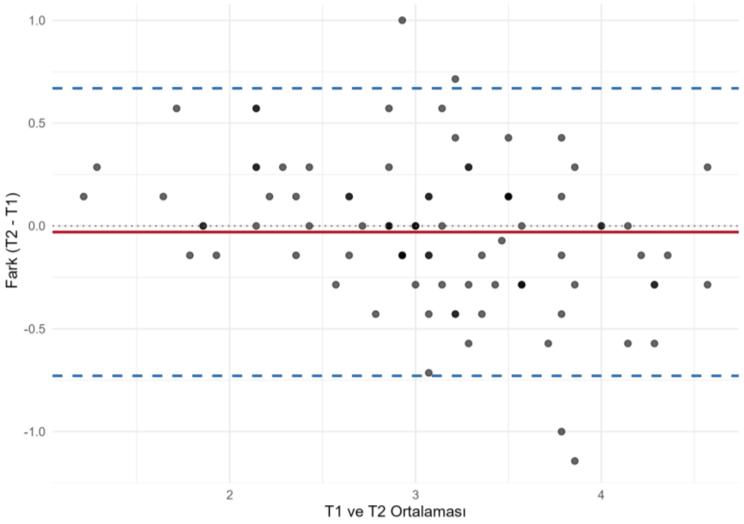


**Figure S13.6.** Bland–Altman plot: IIS (Instrumental–Informational Support).

### S13.5 External Scales — Internal Consistency (Ordinal/Polychoric; N = 607)

**Table S13.5.** External scales: internal consistency (ordinal α with bootstrap CI; ω_t)

| Scale | k | Ordinal α | α CI-lower | α CI-upper | ω_t | Mean IIC |
|---|---|---|---|---|---|---|
| F-SozU K-3 (SD1–SD3) | 3 | .867 | .843 | .891 | .868 | .685 |
| UCLA-6 (UCLA1–UCLA6) | 6 | .808 | .780 | .834 | .809 | .413 |

*Note.* ACI scale not included in this analysis. Bootstrap valid replications = 500/500 for both scales. F-SozU K-3 showed high internal consistency (α = .867); UCLA-6 showed acceptable-to-good internal consistency (α = .808). These results confirm that subsequent convergent validity findings are not attenuated by low external-scale reliability.

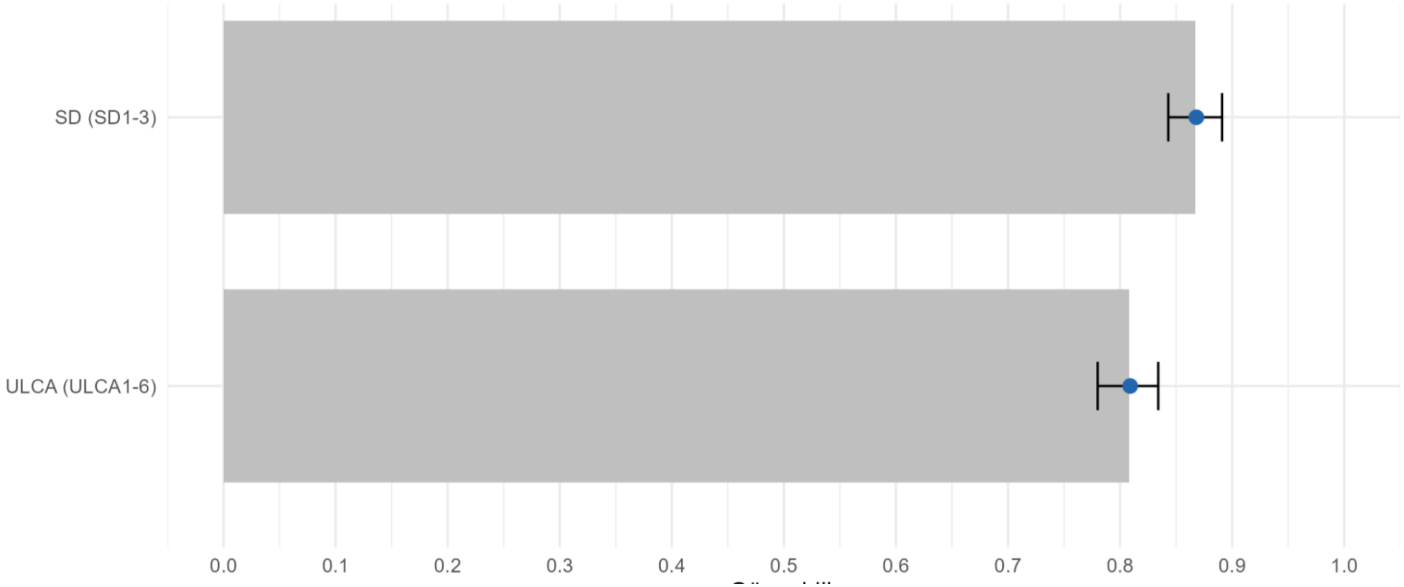


**Figure S13.7.** External scales: ordinal alpha (95% bootstrap CI) and omega-total.

*Note.* Bars = ordinal alpha (polychoric) with 95% bootstrap CI (B = 500); blue dots = omega-total.

## S14. Validity Evidence (PSSDS-28; Full Sample, N = 607)

### S14.1 Overview

Validity evidence for the PSSDS-28 was evaluated within a multiple-evidence framework consistent with the Standards for Educational and Psychological Testing (AERA/APA/NCME, 2014), using the full sample (N = 607). Item indicators were treated as ordinal-categorical; models were estimated with WLSMV and theta parameterization. Validity evidence was organized into three components: (1) construct validity (convergent/discriminant), (2) criterion-related validity with external variables (convergent), and (3) known-groups validity.

## S14.2 Construct Validity (Convergent/Discriminant)

**Table S14.1.** AVE and √AVE (PSSDS-28 factors; full sample; ordinal CFA-5)

| Factor | AVE | √AVE |
|---|---|---|
| FRS | .863 | .929 |
| SES | .518 | .720 |
| IS | .725 | .851 |
| ES | .735 | .858 |
| IIS | .607 | .779 |

**Table S14.2.** Latent inter-factor correlations (CFA-5; full sample)

| | FRS | SES | IS | ES | IIS |
|---|---|---|---|---|---|
| FRS | 1.000 | | | | |
| SES | .574 | 1.000 | | | |
| IS | .220 | .603 | 1.000 | | |
| ES | .507 | .598 | .368 | 1.000 | |
| IIS | .137 | .352 | .611 | .471 | 1.000 |

**Table S14.3.** HTMT matrix (polychoric-based; full sample)

| | FRS | SES | IS | ES | IIS |
|---|---|---|---|---|---|
| FRS | — | .581 | .221 | .509 | .153 |
| SES | | — | .612 | .613 | .364 |
| IS | | | — | .372 | .631 |
| ES | | | | — | .478 |
| IIS | | | | | — |

**Table S14.4.** Construct validity matrix (diagonal = √AVE; lower triangle = latent correlations; upper triangle = HTMT)

| Factor | FRS | SES | IS | ES | IIS |
|---|---|---|---|---|---|
| FRS | .929 | .581 | .221 | .509 | .153 |
| SES | .574 | .720 | .612 | .613 | .364 |
| IS | .220 | .603 | .851 | .372 | .631 |
| ES | .507 | .598 | .368 | .858 | .478 |
| IIS | .137 | .352 | .611 | .471 | .779 |

*Note.* All factors met the AVE > .50 convergent validity criterion. All HTMT values fell below the commonly used .85/.90 discriminant validity thresholds. The three highest HTMT pairs were SES–IS (.612), SES–ES (.613), and IS–IIS (.631), representing the least well-separated block combinations. FRS = Family–Relative Support; SES = Social Environment Support; IS = Institutional Support; ES = Emotional Support; IIS = Instrumental–Informational Support.

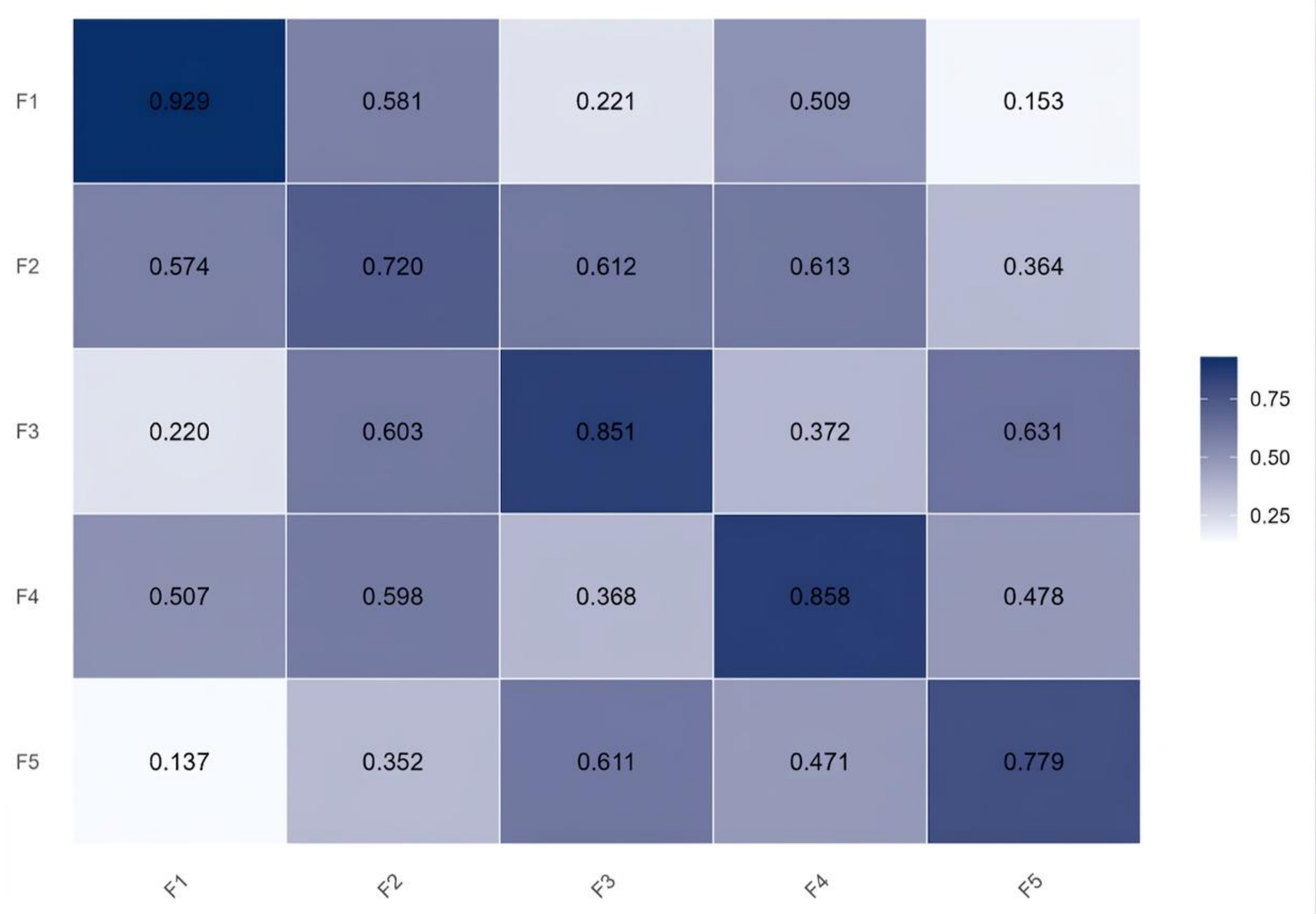


**Figure S14.1.** Construct validity matrix heatmap (PSSDS-28 F1–F5).

*Note.* Diagonal = √AVE; lower triangle = latent correlations; upper triangle = HTMT.

### S14.3 Convergent Validity with External Variables (Full Latent)

Convergent validity was tested against two external scales: F-SozU K-3 (3 items, measuring general perceived social support; higher scores = higher perceived support; expected positive association with PSSDS factors) and UCLA-6 (6 items, measuring loneliness; higher scores = higher loneliness; expected negative association with PSSDS factors). Both scales were considered conceptually proximal to the PSSDS construct and served as convergent validity evidence.

Latent correlations were obtained from the model-implied covariance structure, with corresponding standard errors and z-tests. Multiple testing was controlled using the Benjamini–Hochberg (BH) FDR procedure across the factor × external variable correlation table.

**Table S14.5.** Latent correlations between PSSDS-28 factors and external variables (full latent model; FDR-corrected p values)

| Factor | External | r | SE | z | p | 95% CI | p_fdr |
|---|---|---|---|---|---|---|---|
| FRS | F-SozU K-3 | .673 | .029 | 23.12 | < .001 | [.616, .730] | < .001 |
| SES | F-SozU K-3 | .515 | .036 | 14.33 | < .001 | [.445, .586] | < .001 |
| IS | F-SozU K-3 | .248 | .042 | 5.97 | < .001 | [.167, .330] | < .001 |
| ES | F-SozU K-3 | .665 | .027 | 24.73 | < .001 | [.612, .718] | < .001 |
| IIS | F-SozU K-3 | .216 | .041 | 5.25 | < .001 | [.136, .297] | < .001 |
| FRS | UCLA-6 | −.409 | .047 | −8.68 | < .001 | [−.501, −.317] | < .001 |
| SES | UCLA-6 | −.517 | .041 | −12.57 | < .001 | [−.597, −.436] | < .001 |
| IS | UCLA-6 | −.498 | .039 | −12.93 | < .001 | [−.574, −.423] | < .001 |
| ES | UCLA-6 | −.439 | .041 | −10.65 | < .001 | [−.520, −.358] | < .001 |
| IIS | UCLA-6 | −.460 | .039 | −11.77 | < .001 | [−.536, −.383] | < .001 |

*Note.* All factor × external variable correlations were statistically significant after FDR correction (all p_fdr < .001). PSSDS factors showed positive associations with F-SozU K-3 (r = .216–.673) and negative associations with UCLA-6 (r = −.409 to −.517), both in the theoretically predicted directions. FRS and ES showed the strongest associations with F-SozU K-3 (r = .673 and .665); IS and IIS showed more modest associations (r = .248 and .216). UCLA-6 correlations were more uniform across factors (r = −.409 to −.517). FRS = Family–Relative Support; SES = Social Environment Support; IS = Institutional Support; ES = Emotional Support; IIS = Instrumental–Informational Support. These associations are convergent rather than criterion-related: both external measures are conceptually proximal to perceived social support. Criterion-related validity against clinical, functional, or behavioural outcomes has not been established and is identified in the main text as the most consequential outstanding validity gap.

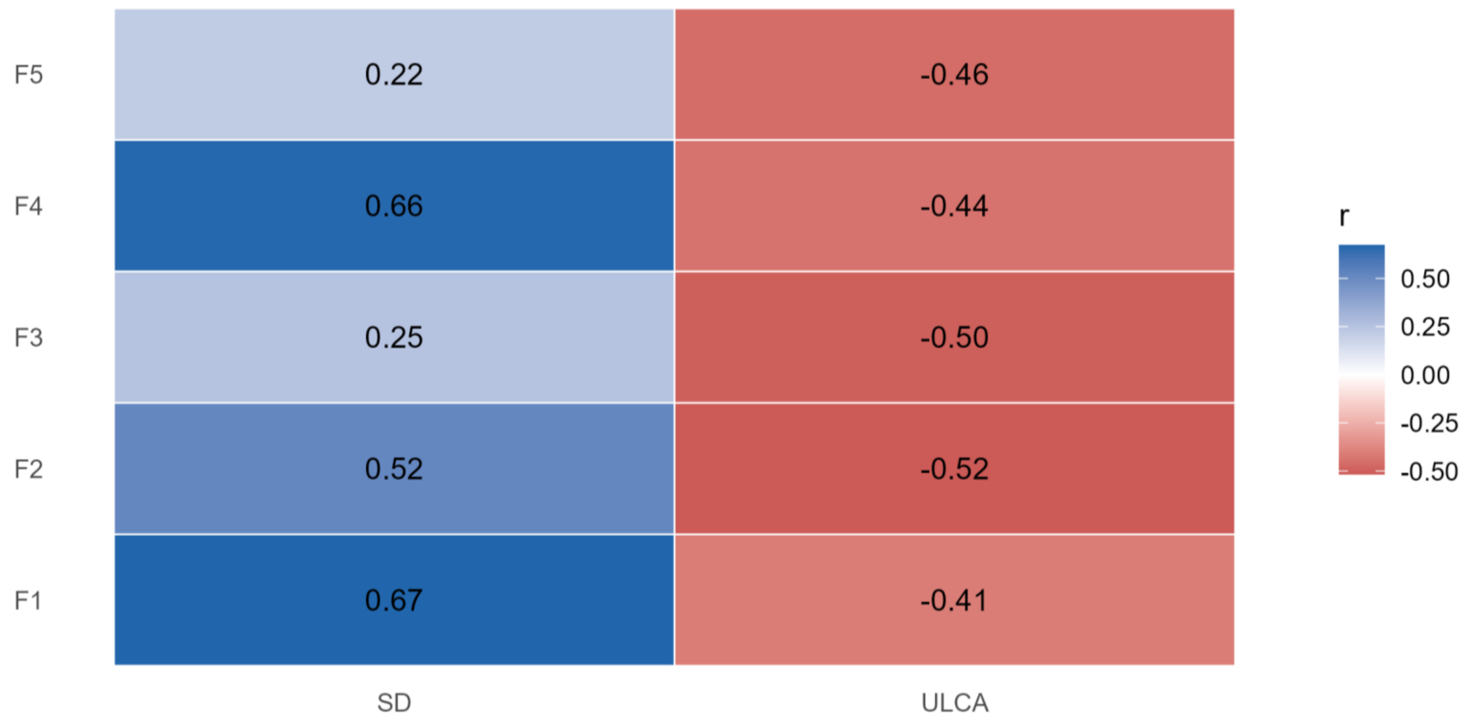


**Figure S14.2.** Latent correlations: PSSDS-28 factors and external variables (F-SozU K-3, UCLA-6).

*Note.* Forest plot showing latent r with 95% CI for each factor × external variable pair. Positive correlations (F-SozU K-3) are shown in the upper panel; negative correlations (UCLA-6) in the lower panel.

**S14.4 Known-Groups Validity (Nine Grouping Variables)**

Known-groups validity was tested a priori across all nine grouping variables defined in S12. Latent mean comparisons were conducted under scalar/strong ordinal invariance (equal loadings + equal thresholds). Three variables (monthly income, loss type, housing status) could not be tested because the scalar model was not estimable (consistent with the configural-level failure reported in S12.3).

**Table S14.6.** Known-groups analysis status by grouping variable

| Grouping variable | Status |
|---|---|
| Age group | Successful |
| Gender | Successful |
| Marital status | Successful |
| Education | Successful |
| Employment | Successful |
| Monthly income | Failed (empty category) |
| Loss type | Failed (empty category) |
| Housing status | Failed (empty category) |
| Psychological support | Successful |

**Table S14.7.** Known-groups latent mean differences (all comparisons; FDR-corrected)

| Grouping variable | Reference | Comparison | Factor | b | SE | z | p | p_fdr |
|---|---|---|---|---|---|---|---|---|
| Age group | 18–40 | 41+ | FRS | +.222 | .117 | 1.895 | .058 | .290 |
| | | | SES | +.056 | .098 | 0.569 | .569 | .712 |
| | | | IS | −.071 | .094 | −0.752 | .452 | .712 |
| | | | ES | +.001 | .096 | 0.006 | .995 | .995 |
| | | | IIS | +.078 | .097 | 0.806 | .420 | .712 |
| Gender | Women | Men | FRS | +.076 | .109 | 0.695 | .487 | .878 |
| | | | SES | +.060 | .095 | 0.633 | .527 | .878 |
| | | | IS | −.003 | .085 | −0.039 | .969 | .969 |
| | | | ES | −.092 | .087 | −1.057 | .290 | .878 |
| | | | IIS | +.022 | .086 | 0.261 | .794 | .969 |
| Marital status | Single | Widowed/Divorced | FRS | −.412 | .235 | −1.757 | .079 | .296 |
| | | | SES | −.370 | .134 | −2.767 | .006 | .057 |
| | | | IS | −.225 | .135 | −1.663 | .096 | .296 |
| | | | ES | +.008 | .166 | 0.051 | .959 | .959 |
| | | | IIS | +.111 | .141 | 0.790 | .430 | .532 |
| Marital status | Single | Married | FRS | +.085 | .119 | 0.709 | .478 | .532 |
| | | | SES | −.108 | .085 | −1.272 | .203 | .296 |
| | | | IS | −.111 | .088 | −1.261 | .207 | .296 |
| | | | ES | +.116 | .091 | 1.276 | .202 | .296 |
| | | | IIS | +.120 | .086 | 1.402 | .161 | .296 |
| Education | High school | Middle school | FRS | +.380 | .148 | 2.572 | .010 | .152 |

| | | | | | | | | |
|---|---|---|---|---|---|---|---|---|
| | | | SES | −.033 | .112 | −0.296 | .767 | .779 |
| | | | IS | +.136 | .096 | 1.406 | .160 | .479 |
| | | | ES | +.211 | .114 | 1.852 | .064 | .435 |
| | | | IIS | +.101 | .101 | 0.999 | .318 | .596 |
| Education | High school | University+ | FRS | +.052 | .186 | 0.281 | .779 | .779 |
| | | | SES | −.215 | .192 | −1.120 | .263 | .596 |
| | | | IS | +.094 | .141 | 0.670 | .503 | .754 |
| | | | ES | +.295 | .181 | 1.627 | .104 | .435 |
| | | | IIS | −.040 | .140 | −0.286 | .775 | .779 |
| Education | High school | Primary or below | FRS | +.309 | .197 | 1.572 | .116 | .435 |
| | | | SES | −.066 | .173 | −0.382 | .702 | .779 |
| | | | IS | +.045 | .149 | 0.303 | .762 | .779 |
| | | | ES | +.183 | .173 | 1.058 | .290 | .596 |
| | | | IIS | +.112 | .150 | 0.745 | .456 | .754 |
| Employment | Homemaker | Employed | FRS | +.179 | .150 | 1.190 | .234 | .319 |
| | | | SES | +.377 | .142 | 2.660 | .008 | .117 |
| | | | IS | +.142 | .135 | 1.048 | .294 | .342 |
| | | | ES | +.172 | .128 | 1.347 | .178 | .267 |
| | | | IIS | +.016 | .133 | 0.119 | .905 | .905 |
| Employment | Homemaker | Student | FRS | +.190 | .182 | 1.043 | .297 | .342 |
| | | | SES | +.332 | .163 | 2.030 | .042 | .132 |
| | | | IS | +.299 | .160 | 1.864 | .062 | .156 |
| | | | ES | +.239 | .145 | 1.643 | .100 | .188 |
| | | | IIS | +.076 | .149 | 0.512 | .609 | .652 |
| Employment | Homemaker | Unemp./Retired | FRS | +.362 | .180 | 2.014 | .044 | .132 |
| | | | SES | +.271 | .134 | 2.016 | .044 | .132 |
| | | | IS | +.190 | .137 | 1.385 | .166 | .267 |
| | | | ES | +.276 | .133 | 2.075 | .038 | .132 |
| | | | IIS | +.235 | .134 | 1.745 | .081 | .173 |
| Psych. support | Yes | No | FRS | +.533 | .189 | 2.826 | .005 | .010† |
| | | | SES | +.609 | .186 | 3.284 | .001 | .005† |
| | | | IS | +.222 | .138 | 1.607 | .108 | .135 |
| | | | ES | +.409 | .148 | 2.754 | .006 | .010† |
| | | | IIS | +.116 | .145 | 0.802 | .422 | .422 |

*Note.* b = latent mean difference (SD units); SE = standard error; z = Wald statistic. Positive b indicates the comparison group had a higher latent mean than the reference group. p_fdr = Benjamini–Hochberg FDR-corrected p value; five factors tested per comparison. †p_fdr < .05. Only the psychological support variable yielded FDR-corrected significant differences: participants not receiving psychological support reported significantly higher latent means on FRS, SES, and ES (b = +.533, +.609, +.409 respectively; all p_fdr ≤ .010). No FDR-corrected significant differences were found for age, gender, marital status, education, or employment. FRS = Family–Relative Support; SES = Social Environment Support; IS = Institutional Support; ES = Emotional Support; IIS = Instrumental–Informational Support.

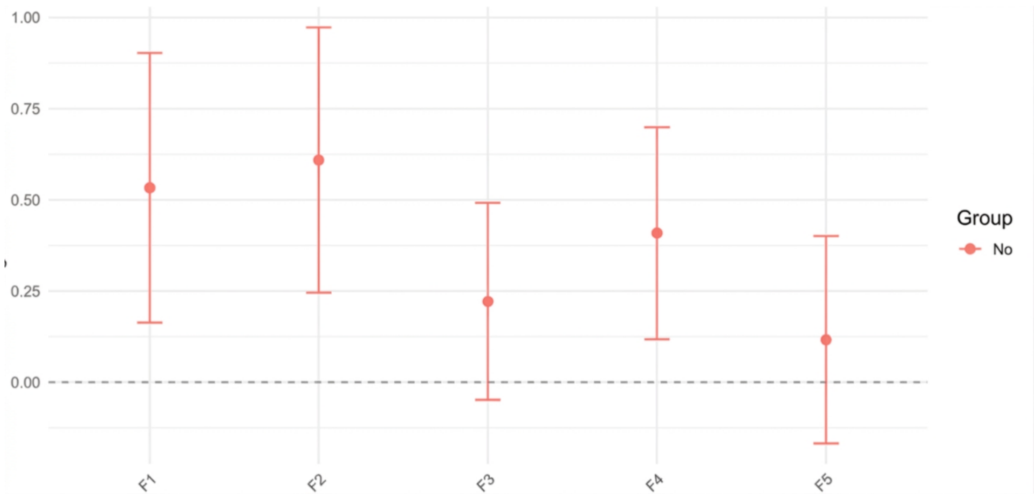


**Figure S14.3.** Known-groups latent mean differences: psychological support (Yes vs. No).

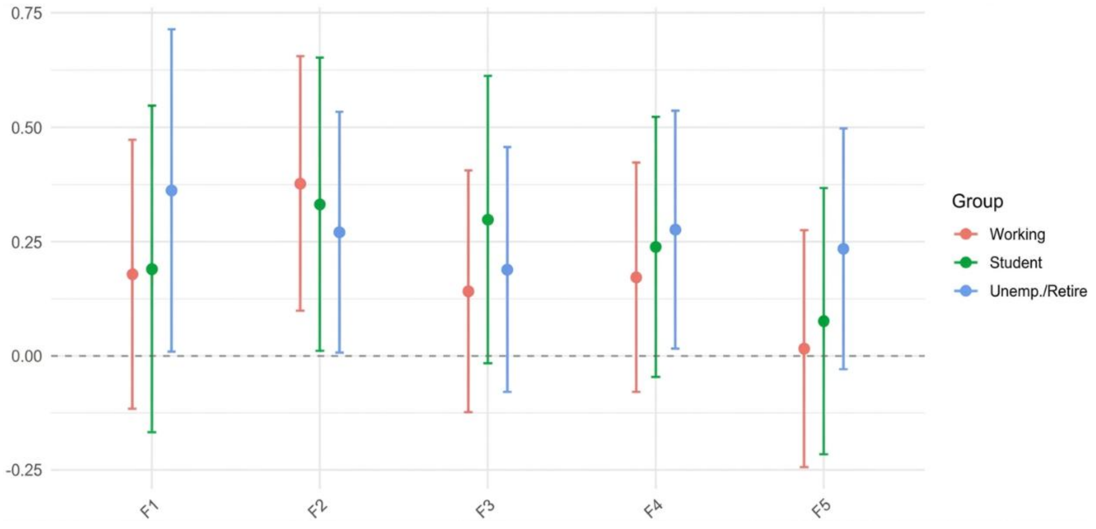


**Figure S14.4.** Known-groups latent mean differences: employment status.

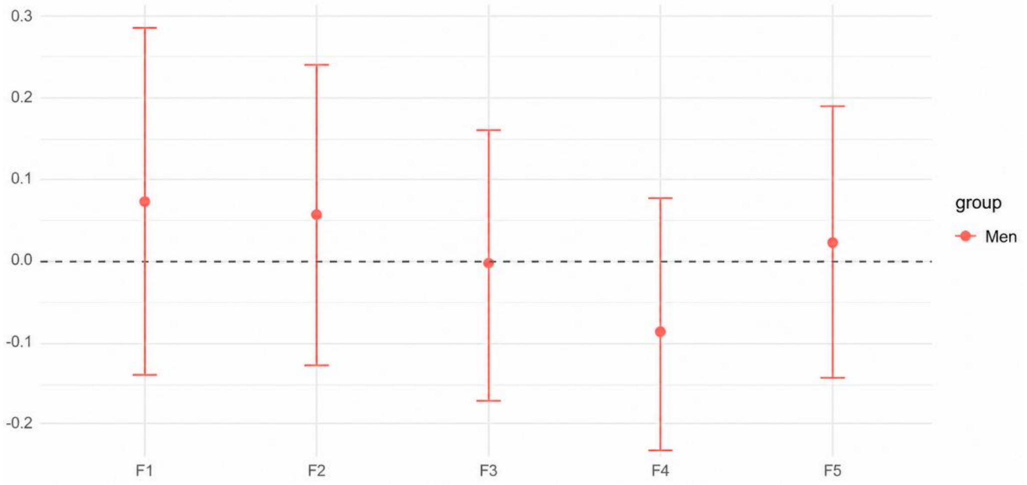


**Figure S14.5.** Known-groups latent mean differences: gender.

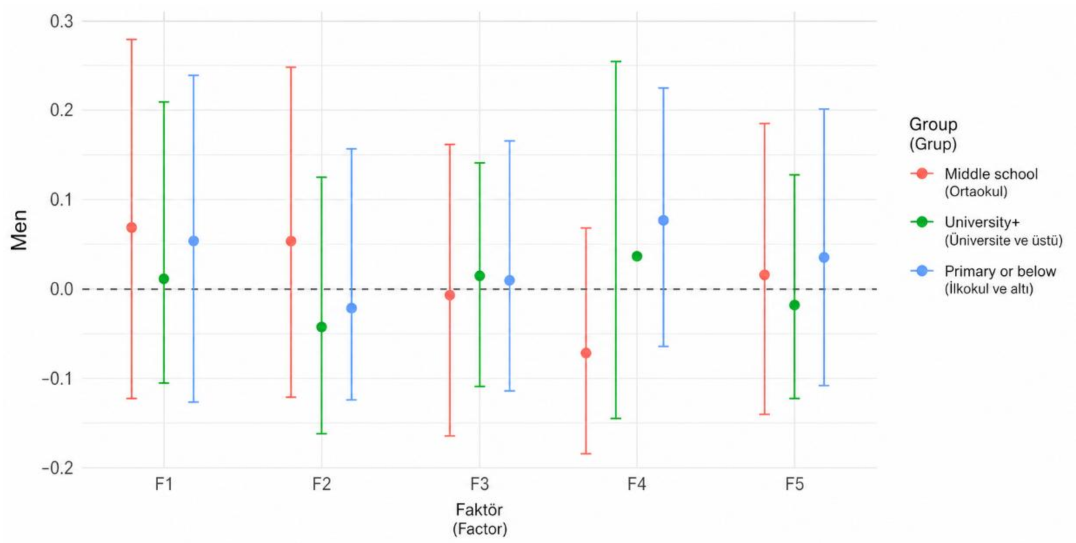


**Figure S14.6.** Known-groups latent mean differences: education level.

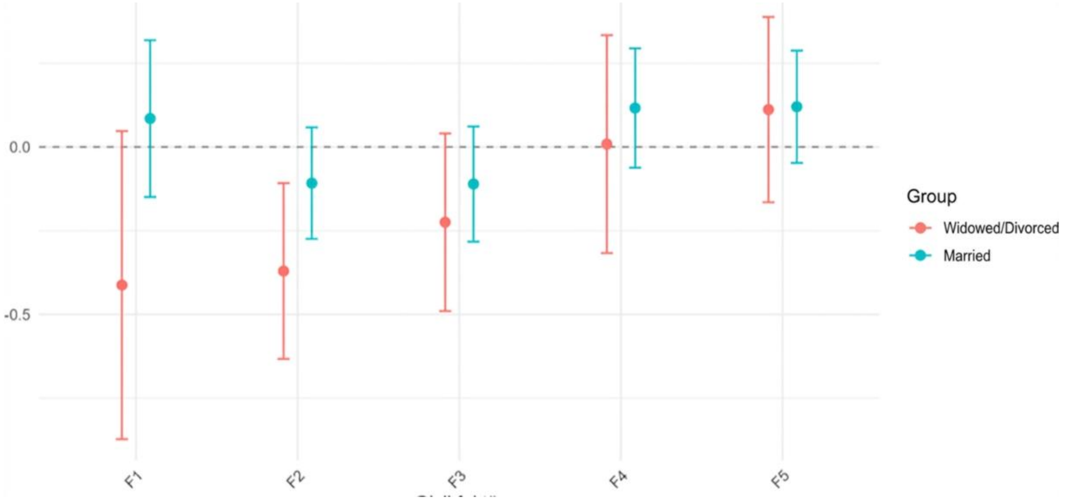


**Figure S14.7.** Known-groups latent mean differences: marital status.

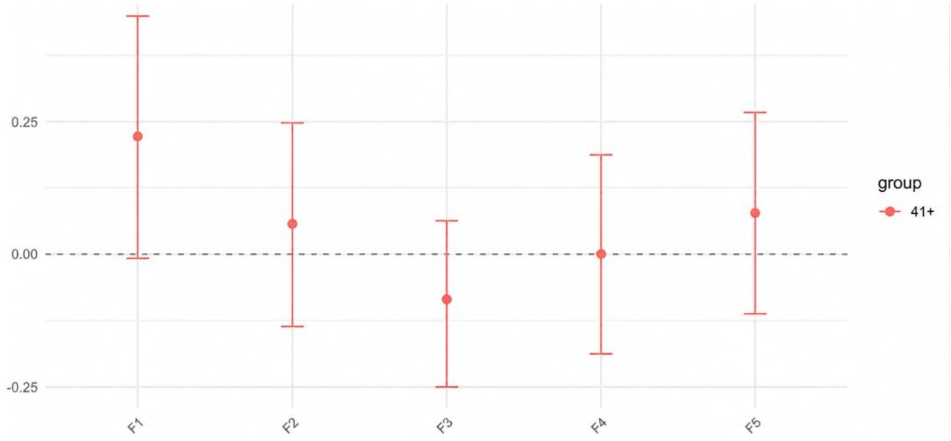


**Figure S14.8.** Known-groups latent mean differences: age group.

## S15. Item Response Theory (IRT) Analyses (PSSDS-28; Full Sample, N = 607)

### S15.1 Missing Data for IRT

**Table S15.1.** Missing data overview (overall)

| N | Complete cases | % Complete | Missing rows | % Missing rows | Total cells | Missing cells | % Missing cells |
|---|---|---|---|---|---|---|---|
| 607 | 548 | 90.28 | 59 | 9.72 | 16,996 | 62 | 0.36 |

**Table S15.2.** Missing data by block

| Block | Items | N | Complete | % Complete | Missing rows | % Missing rows |
|---|---|---|---|---|---|---|
| FRS | 3 | 607 | 605 | 99.67 | 2 | 0.33 |
| SES | 6 | 607 | 598 | 98.52 | 9 | 1.48 |
| IS | 6 | 607 | 596 | 98.19 | 11 | 1.81 |
| ES | 6 | 607 | 591 | 97.36 | 16 | 2.64 |
| IIS | 7 | 607 | 584 | 96.21 | 23 | 3.79 |

*Note.* Missingness was negligible and proportional to block size. Full-information marginal maximum likelihood (EM algorithm) in mirt handled missing data within-model; no imputation was applied.

**S15.2 Assumption Testing**

**Table S15.3.** IRT assumption checks (PSSDS-28 blocks)

| Block | CFI | RMSEA | Var. explained % | Unidimensionality | Mean Q3 | Max Q3 | Local independence | H | Mono. violations | Q3 pairs > .20 |
|---|---|---|---|---|---|---|---|---|---|---|
| FRS | 1.000 | 0.000 | 86.3 | MET | 0.397 | 0.649 | NOT MET | 0.798 | 0 | 2 |
| SES | 0.976 | 0.138 | 51.6 | NOT MET | 0.203 | 0.373 | NOT MET | 0.427 | 0 | 7 |
| IS | 0.998 | 0.089 | 72.5 | NOT MET | 0.205 | 0.303 | NOT MET | 0.677 | 0 | 9 |
| ES | 0.997 | 0.109 | 73.3 | NOT MET | 0.212 | 0.380 | NOT MET | 0.672 | 0 | 9 |
| IIS | 0.975 | 0.192 | 60.7 | NOT MET | 0.222 | 0.438 | NOT MET | 0.534 | 0 | 13 |

*Note.* Unidimensionality evaluated by CFI ≥ .95 and RMSEA ≤ .08. Local independence criterion: |Q3| < 0.20 for all pairs. H = Mokken scale-level scalability coefficient. Monotonicity was perfectly satisfied in all blocks (0 violations). The local independence criterion was not fully met in any block, a known pattern in short, content-homogeneous subscales (Yen, 1993).

**S15.3 Model Comparison: GRM vs. GPCM**

**Table S15.4.** Model comparison (GRM vs. GPCM)

| Block | k | AIC GRM | BIC GRM | AIC GPCM | BIC GPCM | ΔAIC | ΔBIC | Selected |
|---|---|---|---|---|---|---|---|---|
| FRS | 3 | 3284.9 | 3351.1 | 3367.8 | 3433.9 | −82.9 | −82.9 | GRM |
| SES | 6 | 9478.5 | 9610.8 | 9601.0 | 9733.3 | −122.5 | −122.5 | GRM |
| IS | 6 | 9008.8 | 9141.1 | 9137.4 | 9269.6 | −128.6 | −128.6 | GRM |
| ES | 6 | 8038.8 | 8171.1 | 8176.9 | 8309.1 | −138.0 | −138.0 | GRM |
| IIS | 7 | 11477.3 | 11631.6 | 11566.0 | 11720.3 | −88.7 | −88.7 | GRM |

*Note.* GRM was preferred in all five blocks based on lower AIC and BIC (ΔAIC = −82.9 to −138.0).

**S15.4 Item Parameters**

**Table S15.5.** IRT item parameters (GRM; sandwich SE)

| Block | Item | a | SE(a) | b1 | SE | b2 | SE | b3 | SE | b4 | SE |
|---|---|---|---|---|---|---|---|---|---|---|---|
| FRS | asd1 | 4.711 | 0.640 | −1.601 | 0.255 | −1.301 | 0.229 | −0.921 | 0.192 | −0.303 | 0.138 |
| FRS | asd2 | 17.554 | 8.952 | −1.573 | 0.262 | −1.165 | 0.212 | −0.794 | 0.169 | −0.240 | 0.129 |
| FRS | asd3 | 3.420 | 0.454 | −1.617 | 0.262 | −1.295 | 0.233 | −0.785 | 0.183 | −0.095 | 0.126 |
| SES | asd7 | 1.373 | 0.154 | −2.084 | 0.190 | −1.477 | 0.142 | −0.609 | 0.089 | 0.479 | 0.091 |
| SES | asd8 | 1.675 | 0.170 | −1.790 | 0.141 | −1.115 | 0.098 | −0.344 | 0.071 | 0.657 | 0.090 |

| | | | | | | | | | | | |
|---|---|---|---|---|---|---|---|---|---|---|---|
| SES | asd9 | 2.269 | 0.243 | −1.833 | 0.136 | −1.254 | 0.100 | −0.590 | 0.069 | 0.347 | 0.068 |
| SES | asd10 | 2.702 | 0.286 | −1.759 | 0.126 | −1.189 | 0.089 | −0.489 | 0.062 | 0.285 | 0.063 |
| SES | asd11 | 1.997 | 0.226 | −1.844 | 0.147 | −1.213 | 0.106 | −0.444 | 0.070 | 0.345 | 0.072 |
| SES | asd12 | 1.545 | 0.176 | −2.035 | 0.177 | −1.498 | 0.139 | −0.742 | 0.091 | 0.353 | 0.081 |
| IS | asd13 | 2.380 | 0.194 | −1.335 | 0.100 | −0.744 | 0.076 | −0.090 | 0.061 | 0.671 | 0.069 |
| IS | asd14 | 2.623 | 0.211 | −1.352 | 0.096 | −0.781 | 0.075 | 0.007 | 0.058 | 0.777 | 0.069 |
| IS | asd15 | 3.409 | 0.281 | −1.020 | 0.077 | −0.450 | 0.060 | 0.216 | 0.054 | 0.882 | 0.067 |
| IS | asd16 | 4.615 | 0.407 | −0.920 | 0.071 | −0.454 | 0.058 | 0.157 | 0.051 | 0.848 | 0.063 |
| IS | asd17 | 2.924 | 0.238 | −1.126 | 0.084 | −0.597 | 0.066 | 0.008 | 0.056 | 0.692 | 0.065 |
| IS | asd18 | 3.132 | 0.269 | −1.021 | 0.079 | −0.599 | 0.066 | 0.044 | 0.055 | 0.658 | 0.062 |
| ES | asd19 | 3.215 | 0.304 | −1.257 | 0.085 | −0.917 | 0.070 | −0.330 | 0.056 | 0.343 | 0.059 |
| ES | asd20 | 4.064 | 0.421 | −1.453 | 0.091 | −1.123 | 0.077 | −0.485 | 0.057 | 0.396 | 0.058 |
| ES | asd21 | 4.389 | 0.419 | −1.447 | 0.089 | −1.007 | 0.070 | −0.495 | 0.056 | 0.347 | 0.056 |
| ES | asd22 | 2.880 | 0.249 | −1.770 | 0.116 | −1.214 | 0.088 | −0.548 | 0.063 | 0.419 | 0.062 |
| ES | asd23 | 2.708 | 0.234 | −1.793 | 0.119 | −1.309 | 0.092 | −0.596 | 0.065 | 0.417 | 0.063 |
| ES | asd24 | 2.329 | 0.198 | −1.768 | 0.122 | −1.228 | 0.092 | −0.313 | 0.062 | 0.566 | 0.069 |
| IIS | asd25 | 1.376 | 0.153 | −1.601 | 0.171 | −0.923 | 0.122 | −0.129 | 0.082 | 0.906 | 0.098 |
| IIS | asd26 | 1.679 | 0.174 | −1.473 | 0.139 | −0.761 | 0.100 | 0.048 | 0.070 | 0.998 | 0.090 |
| IIS | asd27 | 2.121 | 0.209 | −0.850 | 0.087 | −0.377 | 0.071 | 0.351 | 0.063 | 1.201 | 0.092 |
| IIS | asd28 | 2.594 | 0.238 | −0.680 | 0.075 | −0.107 | 0.060 | 0.519 | 0.059 | 1.273 | 0.083 |
| IIS | asd29 | 2.988 | 0.321 | −0.916 | 0.083 | −0.324 | 0.065 | 0.290 | 0.057 | 1.109 | 0.077 |
| IIS | asd30 | 2.970 | 0.322 | −0.799 | 0.078 | −0.293 | 0.063 | 0.327 | 0.058 | 1.211 | 0.084 |
| IIS | asd31 | 1.875 | 0.185 | −0.395 | 0.074 | 0.164 | 0.066 | 0.794 | 0.080 | 1.560 | 0.118 |

*Note.* Threshold (b) parameters are cumulative thresholds; higher b values indicate higher trait levels (θ) required to endorse the corresponding category. SE = sandwich (robust) standard error. Item asd2 showed an extremely high discrimination point estimate (a = 17.554) with a correspondingly large standard error (SE = 8.952); this estimate is statistically imprecise (a/SE ≈ 1.96) and likely reflects an artifact of unmodeled local dependence in the three-item FRS block (see S15.2, Table S15.3, for Q3 values).

**S15.5 Model Fit**

**Table S15.6.** Model fit summary (M2/S-X²/LD)

| **Block** | **Model** | **M2** | **df** | **p** | **RMSEA (M2)** | **SRMR** | **S-X² misfit items** | **LD > 10 pairs** |
|---|---|---|---|---|---|---|---|---|
| FRS | GRM | — | — | — | — | — | 0 | 3 |
| SES | GRM | 131.62 | 9 | < .001 | 0.150 | 0.091 | 2 | 15 |
| IS | GRM | 90.27 | 9 | < .001 | 0.122 | 0.055 | 0 | 15 |

| | | | | | | | | |
|---|---|---|---|---|---|---|---|---|
| ES | GRM | 133.29 | 9 | < .001 | 0.151 | 0.059 | 0 | 15 |
| IIS | GRM | 392.86 | 14 | < .001 | 0.211 | 0.101 | 0 | 21 |

*Note.* M2 could not be computed for FRS (insufficient df for 3-item block). M2 was statistically significant for SES–IIS (all $p < .001$) with RMSEA(M2) values exceeding the .08 threshold (.122–.211). S-$X^2$ (Bonferroni-corrected within block) flagged two items in SES (asd10: S-$X^2$ = 67.58, $p = .004$; asd11: S-$X^2$ = 78.79, $p = .003$); all other items showed acceptable fit.

**Table S15.7.** S-$X^2$ item fit detail (within-block Bonferroni-corrected)

| **Block** | **Item** | **S-$X^2$** | **df** | **p** | **Result** |
|---|---|---|---|---|---|
| FRS | asd1 | 8.26 | 8 | .409 | OK |
| FRS | asd2 | 12.53 | 5 | .028 | OK |
| FRS | asd3 | 10.99 | 9 | .277 | OK |
| SES | asd7 | 68.97 | 51 | .048 | OK |
| SES | asd8 | 49.55 | 48 | .411 | OK |
| SES | asd9 | 53.15 | 43 | .138 | OK |
| SES | asd10 | 67.58 | 40 | .004 | MISFIT |
| SES | asd11 | 78.79 | 47 | .003 | MISFIT |
| SES | asd12 | 47.44 | 52 | .654 | OK |
| IS | asd13 | 44.14 | 48 | .632 | OK |
| IS | asd14 | 57.47 | 39 | .029 | OK |
| IS | asd15 | 54.01 | 38 | .044 | OK |
| IS | asd16 | 42.30 | 32 | .105 | OK |
| IS | asd17 | 48.99 | 42 | .213 | OK |
| IS | asd18 | 50.35 | 40 | .127 | OK |
| ES | asd19 | 50.77 | 38 | .080 | OK |
| ES | asd20 | 25.28 | 29 | .663 | OK |
| ES | asd21 | 29.01 | 29 | .465 | OK |
| ES | asd22 | 51.17 | 35 | .038 | OK |
| ES | asd23 | 44.93 | 36 | .146 | OK |
| ES | asd24 | 63.12 | 39 | .009 | OK |
| IIS | asd25 | 97.98 | 68 | .010 | OK |
| IIS | asd26 | 87.40 | 66 | .040 | OK |
| IIS | asd27 | 84.97 | 58 | .012 | OK |
| IIS | asd28 | 71.55 | 52 | .037 | OK |
| IIS | asd29 | 53.62 | 50 | .337 | OK |
| IIS | asd30 | 51.87 | 49 | .363 | OK |

| | | | | | |
|---|---|---|---|---|---|
| IIS | asd31 | 79.65 | 63 | .077 | OK |

*Note.* Bonferroni-corrected alpha levels: FRS α = .0167; SES/IS/ES α = .0083; IIS α = .0071. "MISFIT" = p below corrected threshold.

### S15.6 Test Information and Measurement Precision

**Table S15.8.** Test information function summary (peak information and reliability > .70 range)

| **Block** | **Peak θ** | **Peak information** | **Reliability > .70 lower** | **Reliability > .70 upper** |
|---|---|---|---|---|
| FRS | −0.8 | 86.40 | −2.1 | 0.2 |
| SES | −1.2 | 7.27 | −2.5 | 1.1 |
| IS | −0.6 | 18.85 | −1.9 | 1.6 |
| ES | −1.2 | 19.96 | −2.4 | 1.2 |
| IIS | −0.2 | 11.35 | −1.7 | 2.1 |

*Note.* FRS's extreme peak information (86.40) is a mathematical consequence of asd2's inflated discrimination estimate and should not be interpreted as genuine measurement precision superiority. IIS showed the widest reliable θ range (−1.7 to 2.1), consistent with its larger item count (k = 7).

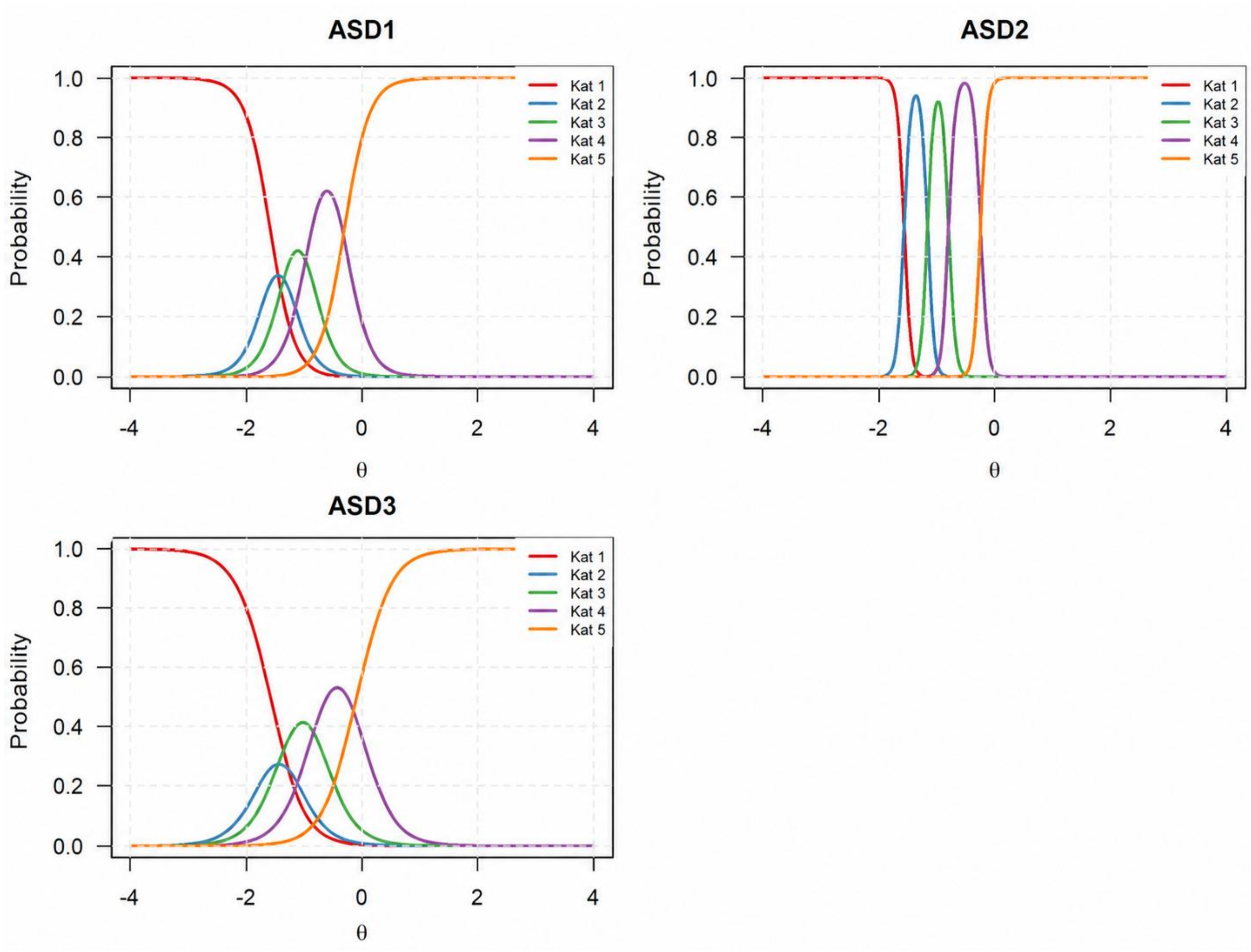


**Figure S15.1.** Item characteristic curves (ICC) — FRS: Family–Relative Support.

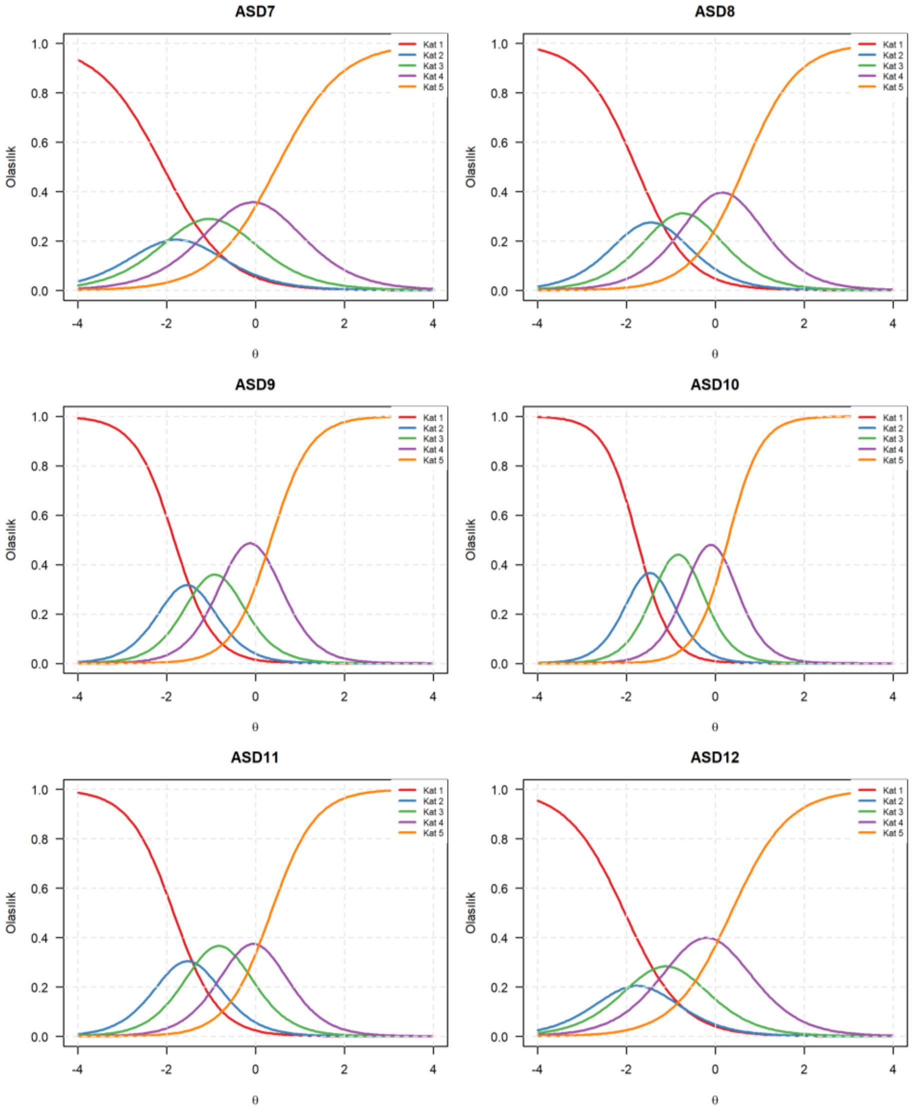


**Figure S15.2.** Item characteristic curves (ICC) — SES: Social Environment Support.

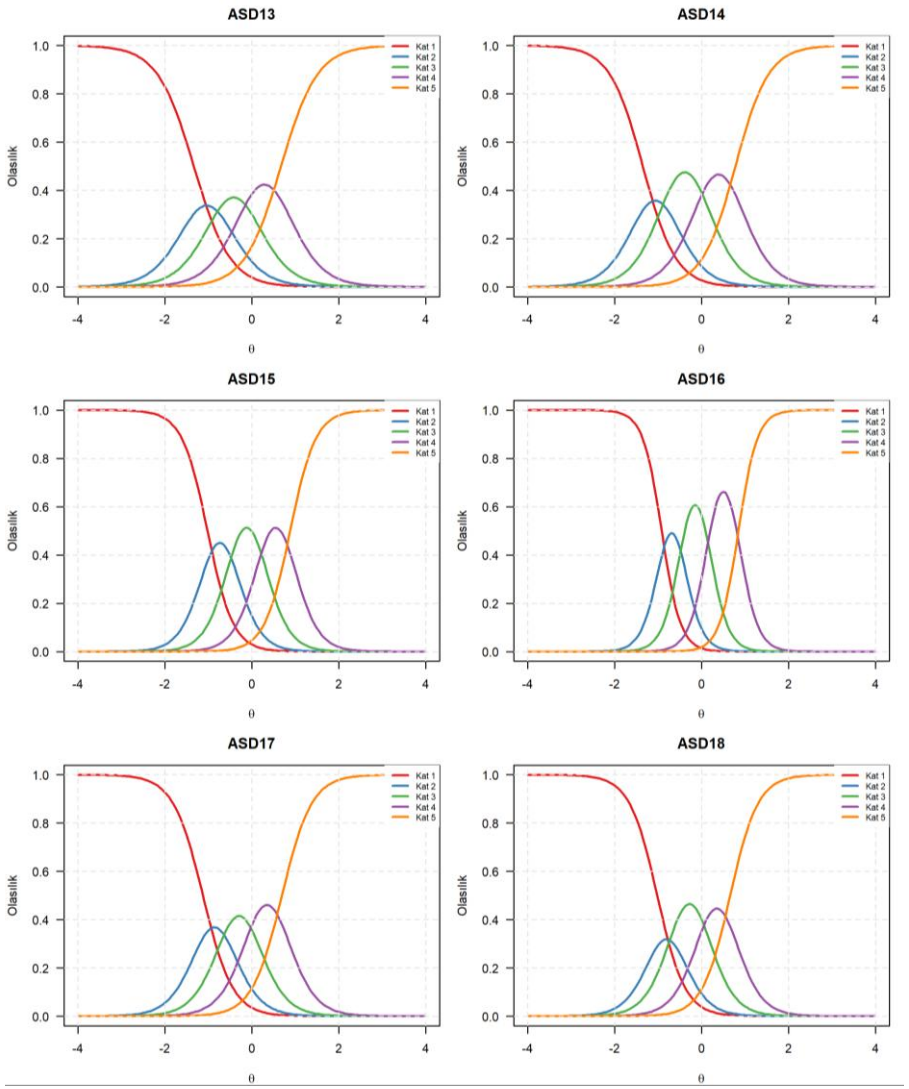


**Figure S15.3.** Item characteristic curves (ICC) — IS: Institutional Support.

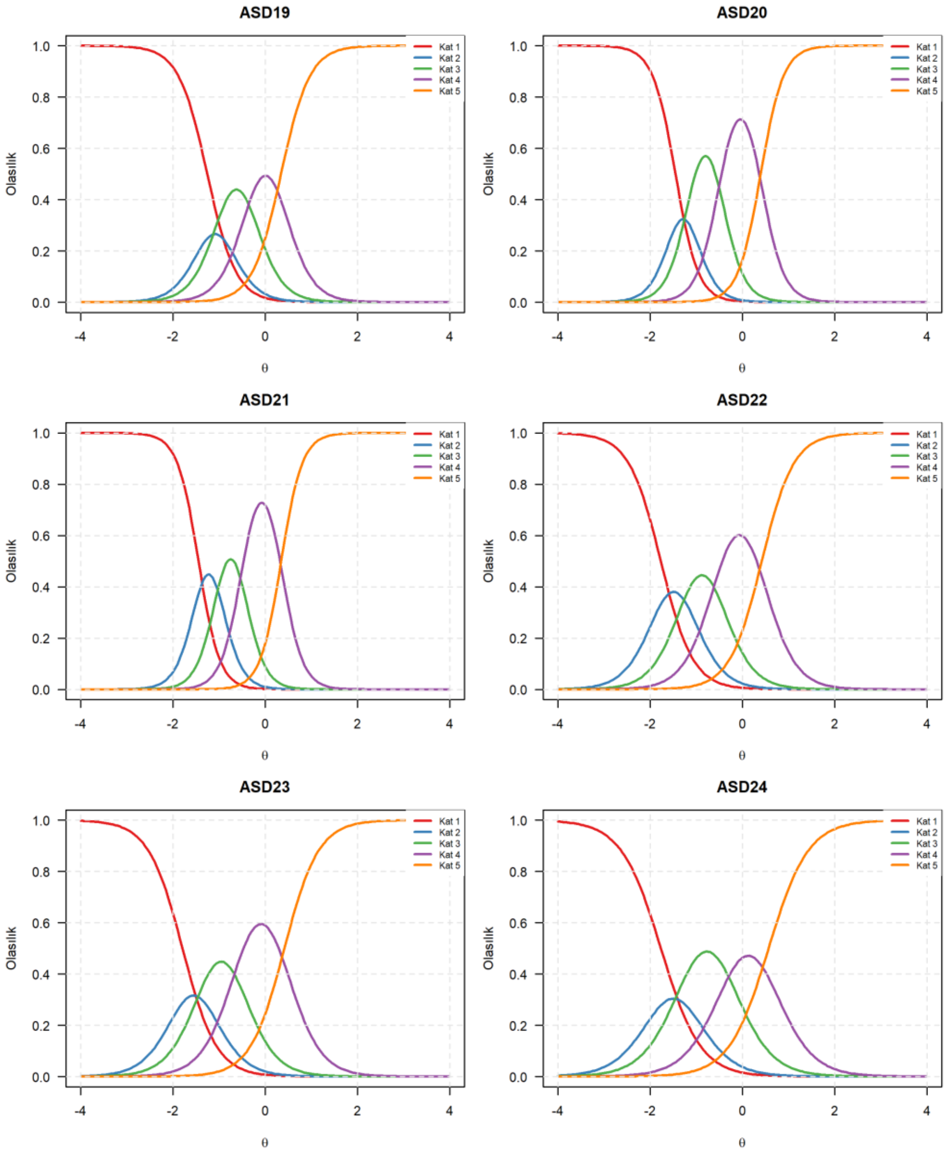


**Figure S15.4.** Item characteristic curves (ICC) — ES: Emotional Support.

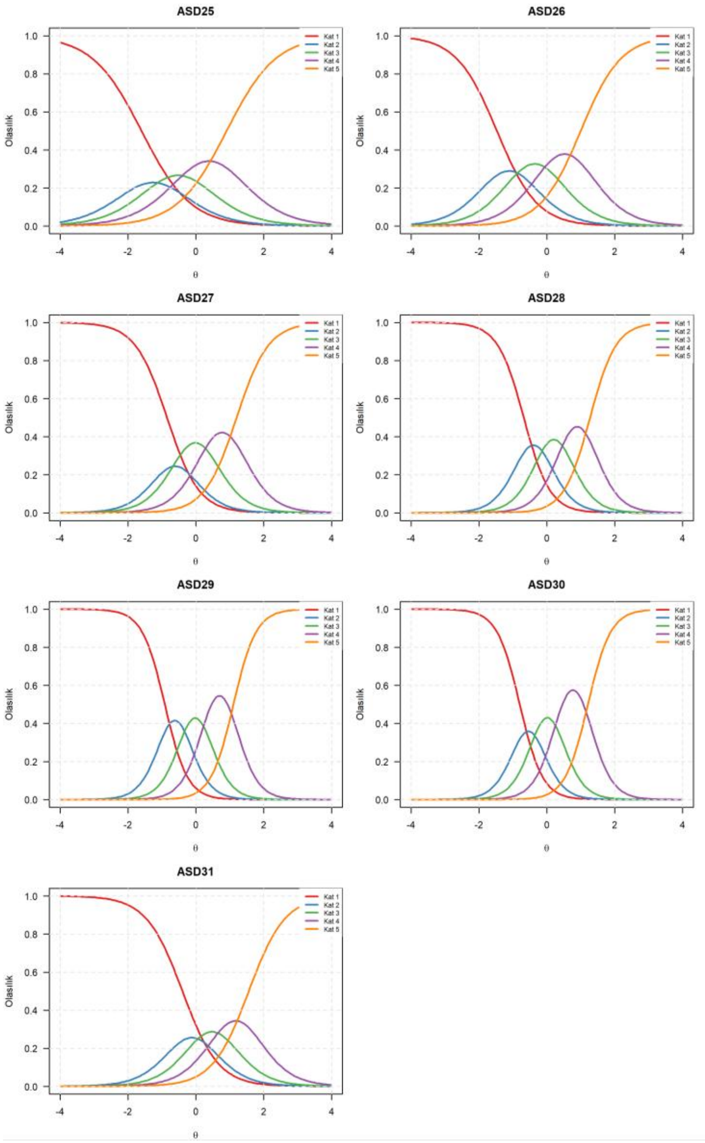


**Figure S15.5.** Item characteristic curves (ICC) — IIS: Instrumental–Informational Support.

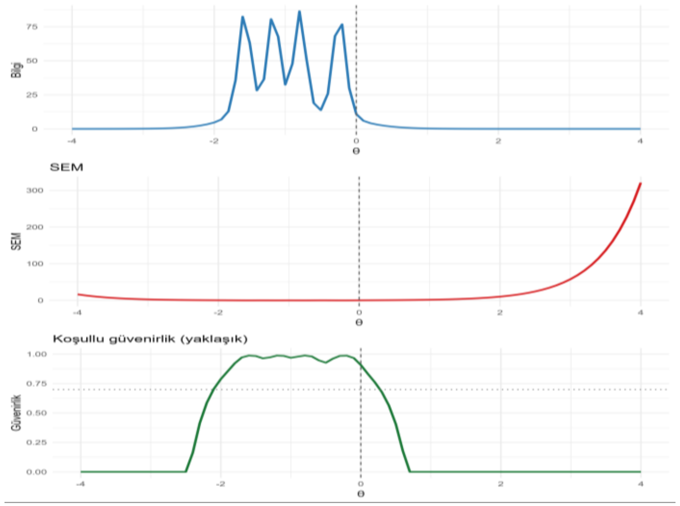


**Figure S15.6.** Test information function (TIF), conditional SEM, and approximate conditional reliability — FRS.

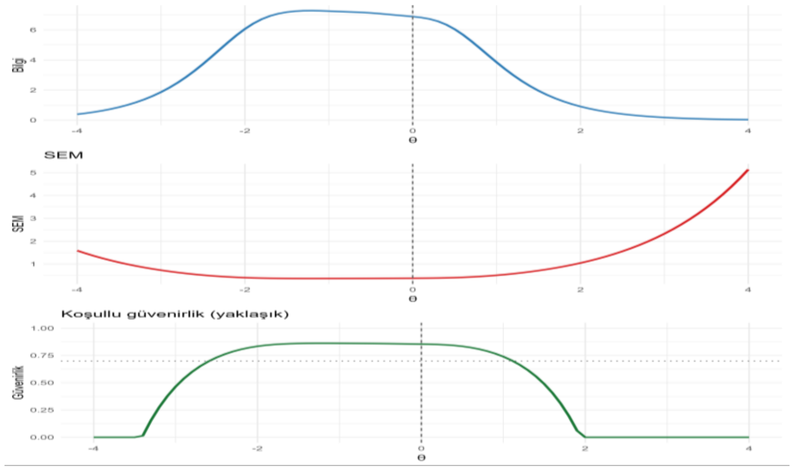


**Figure S15.7.** Test information function (TIF), conditional SEM, and approximate conditional reliability — SES.

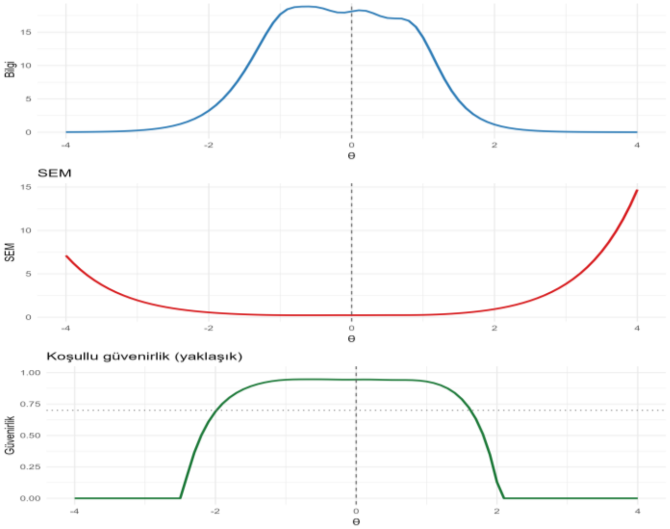


**Figure S15.8.** Test information function (TIF), conditional SEM, and approximate conditional reliability — IS.

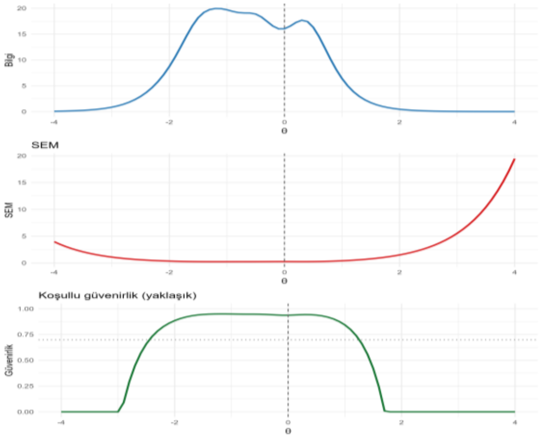


**Figure S15.9.** Test information function (TIF), conditional SEM, and approximate conditional reliability — ES.

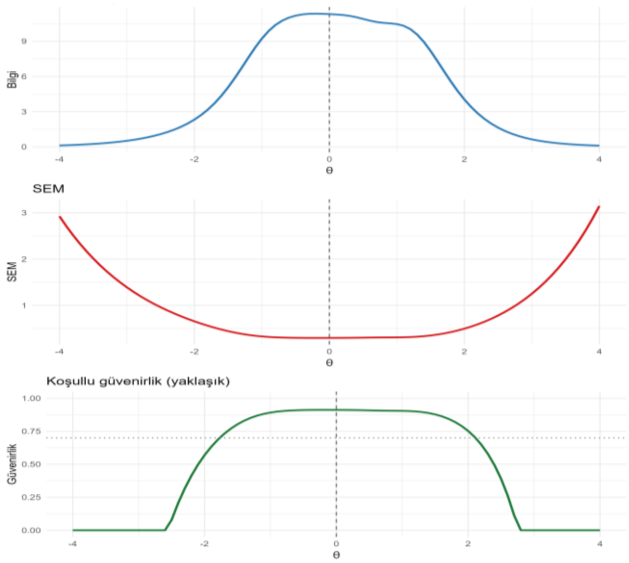


**Figure S15.10.** Test information function (TIF), conditional SEM, and approximate conditional reliability — IIS.

**S15.7 Mokken Scale Analysis**

**Table S15.9.** Mokken scale analysis results (by block)

| Block | H | SE(H) | Min Hi | Max Hi | H quality | AISP scales | IIO violations | Mono. violations |
|---|---|---|---|---|---|---|---|---|
| FRS | 0.798 | 0.025 | 0.773 | 0.827 | Strong | 1 | 0 | 0 |
| SES | 0.427 | 0.025 | 0.368 | 0.472 | Good | 1 | 0 | 0 |
| IS | 0.677 | 0.019 | 0.647 | 0.730 | Strong | 1 | 0 | 0 |
| ES | 0.672 | 0.022 | 0.643 | 0.708 | Strong | 1 | 0 | 0 |
| IIS | 0.534 | 0.020 | 0.456 | 0.572 | Strong | 1 | 0 | 0 |

*Note.* All blocks formed the expected single scale (AISP = 1) with zero IIO and monotonicity violations. Scalability ranged from "Good" (SES) to "Strong" (FRS, IS, ES, IIS). The H_Quality labels are those produced by the analysis script. Under the conventional Mokken classification (weak .30–.40; moderate .40–.50; strong ≥ .50) the SES coefficient of .427 corresponds to moderate scalability and the remaining four blocks to strong scalability.

## S15.8 Wright Maps

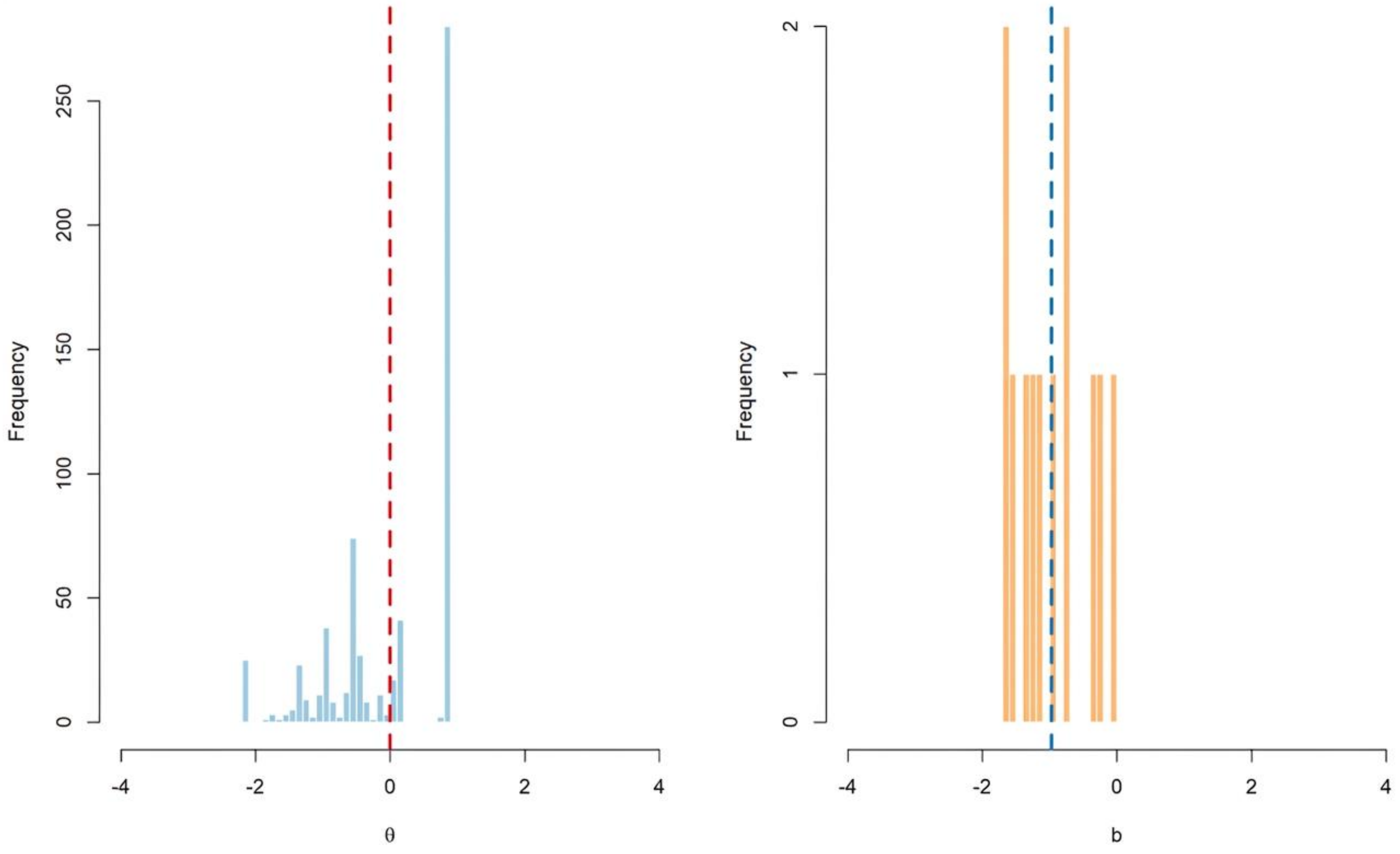


**Figure S15.11.** Wright map (person–item targeting) — FRS.

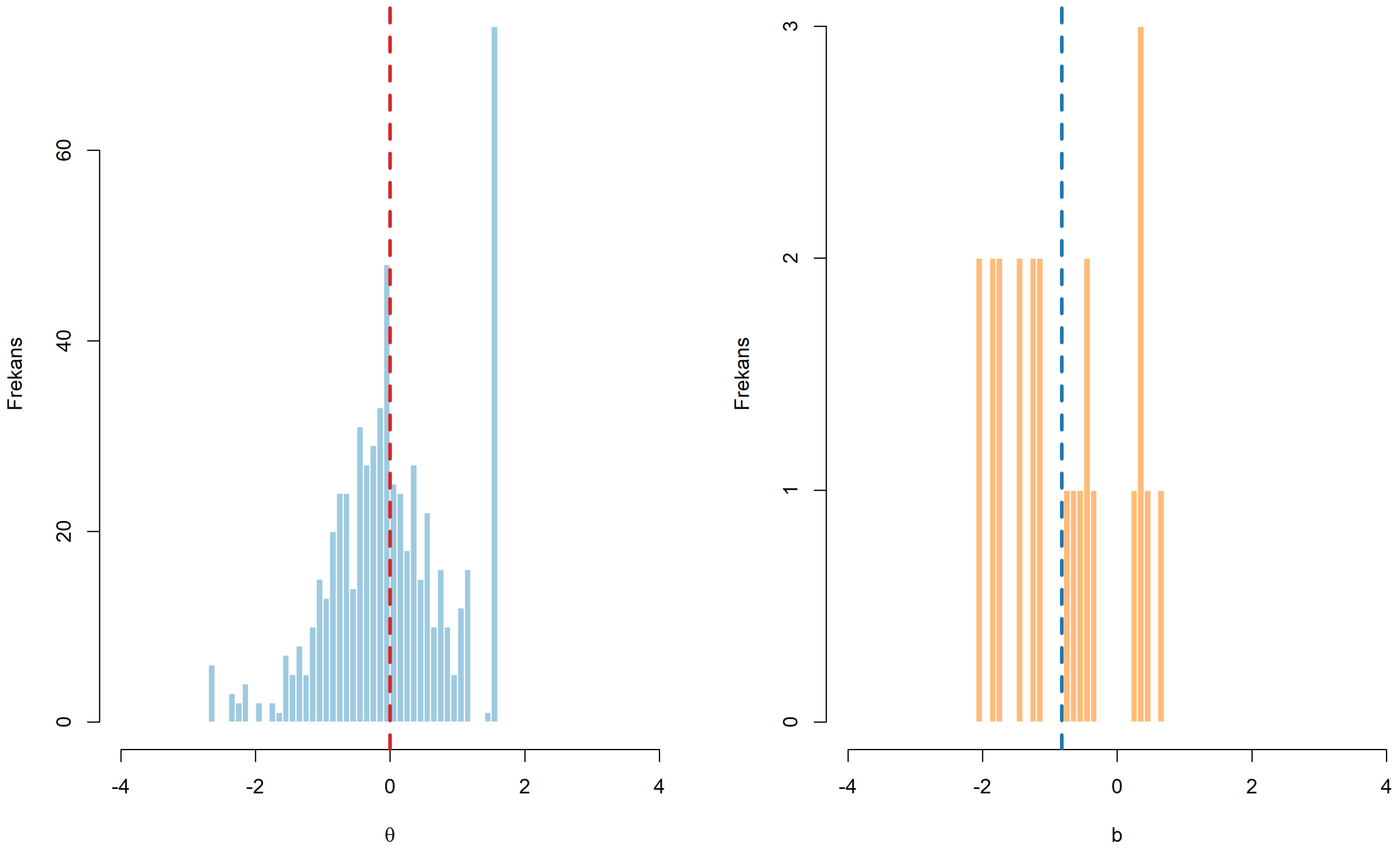


**Figure S15.12.** Wright map (person–item targeting) — SES.

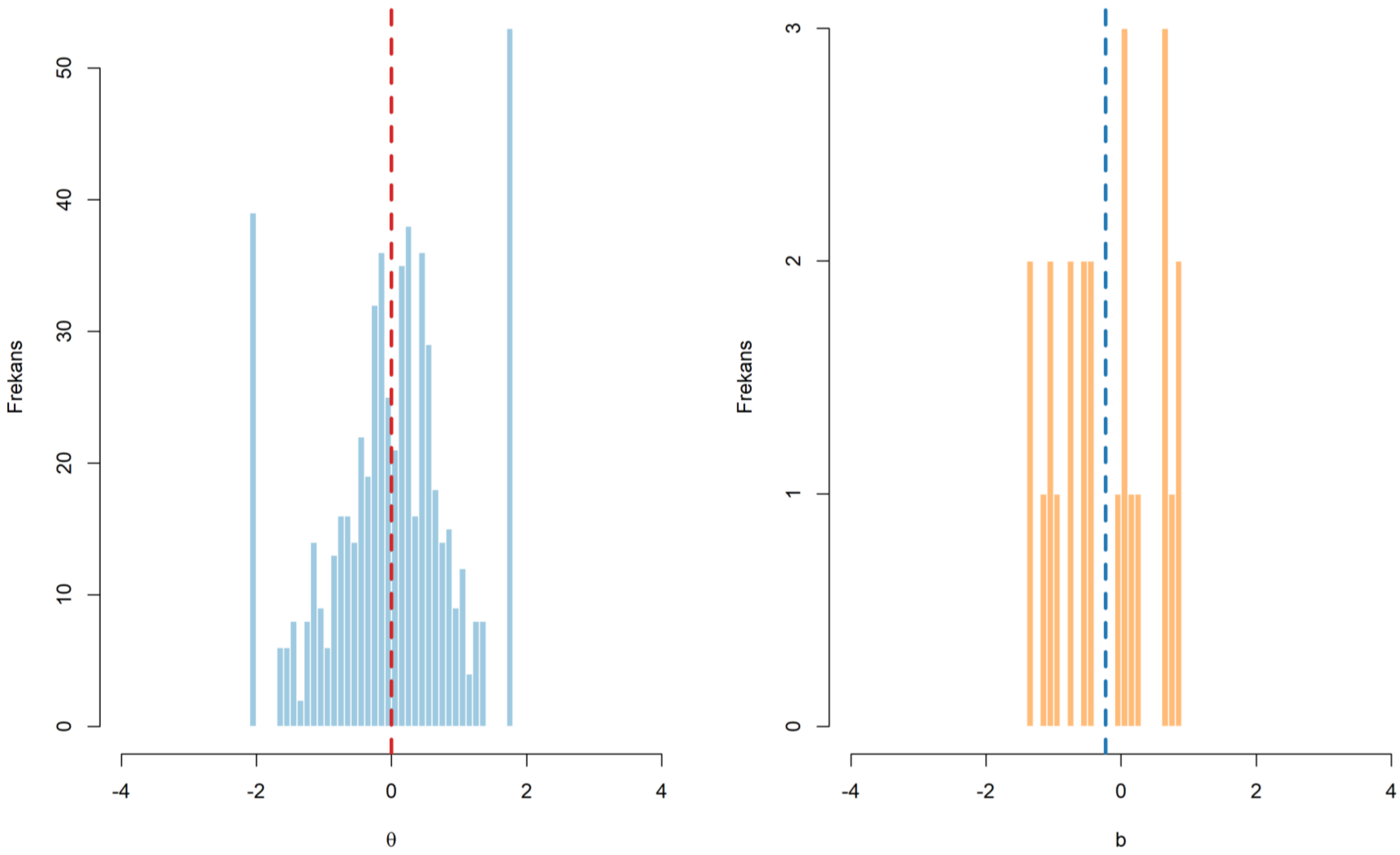


**Figure S15.13.** Wright map (person–item targeting) — IS.

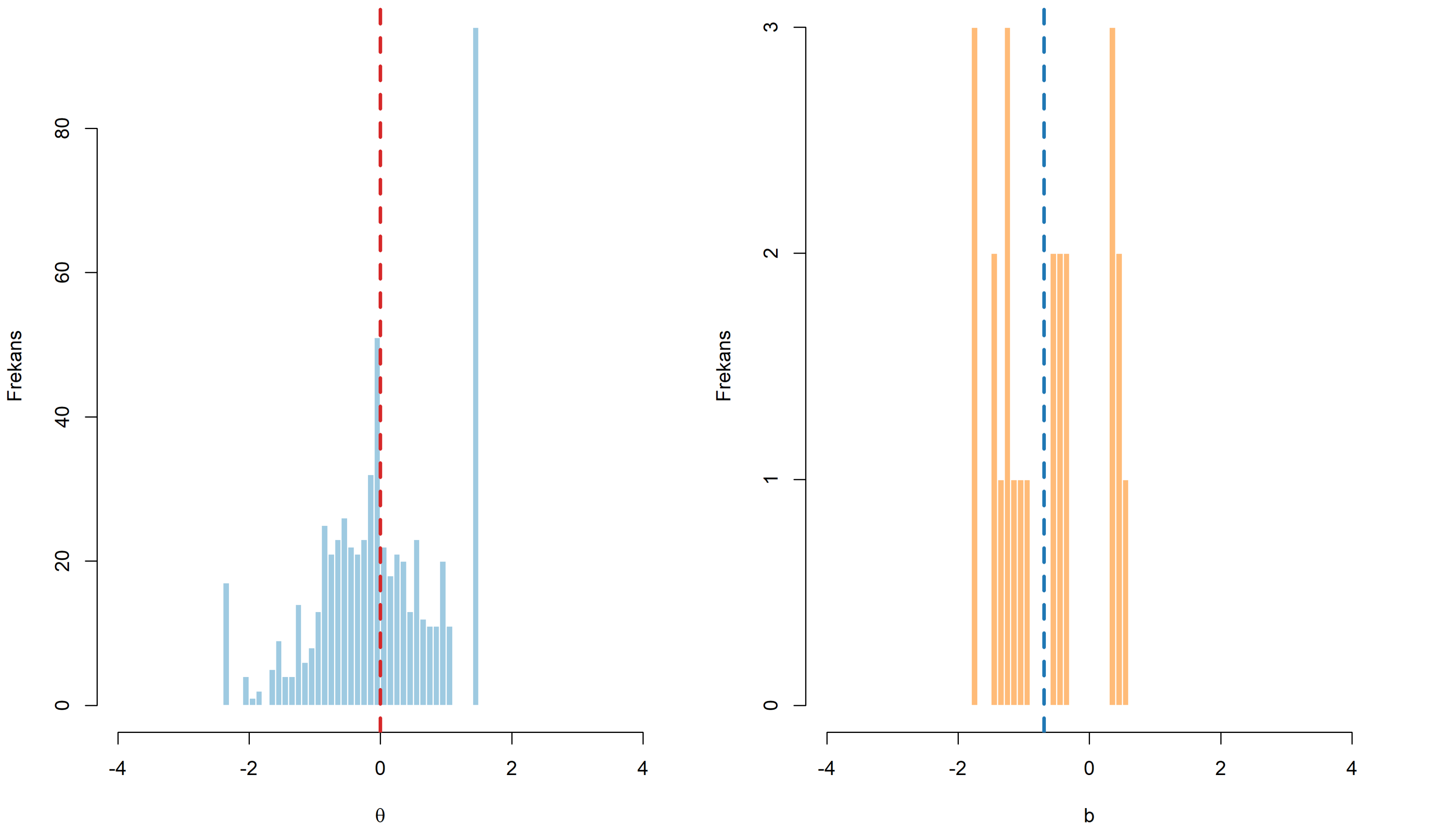


**Figure S15.14.** Wright map (person–item targeting) — ES.

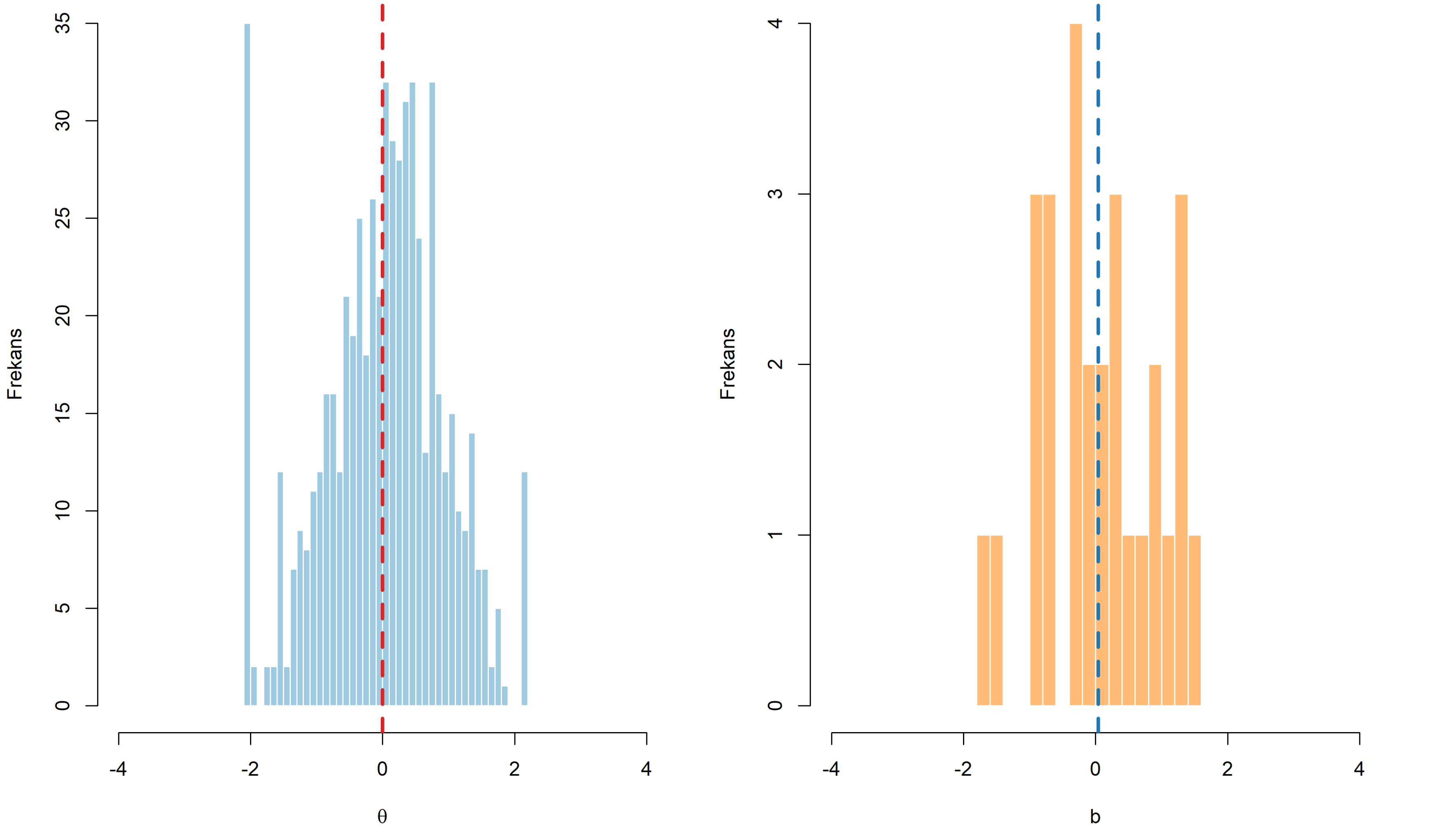


**Figure S15.15**. Wright map (person–item targeting) — IIS.

*Note.* Wright maps for FRS, SES, IS, and ES showed the person ability distribution concentrated near the upper bound of the item threshold distribution, indicating that the majority of participants endorsed relatively high support levels. IIS showed the broadest person-θ spread.

## S16. Network Psychometrics (PSSDS-28; Full Sample, N = 607)

### S16.1 Primary Network Estimation (EBICglasso GGM)

The primary network was estimated from the polychoric correlation matrix using EBICglasso regularization ($\gamma = 0.50$; threshold = FALSE). All five CFA blocks were modeled within a single 28-node network; CFA block labels (F1–F5) were used as community labels.

**Table S16.1.** Network structure summary (primary analysis; threshold = FALSE)

| Items | Nonzero edges | Possible edges | Sparsity | Components |
|---|---|---|---|---|
| 28 | 172 | 378 | 0.545 | 1 |

*Note.* Sparsity = 1 − (nonzero edges / possible edges). A single connected component confirmed that all blocks remained interconnected.

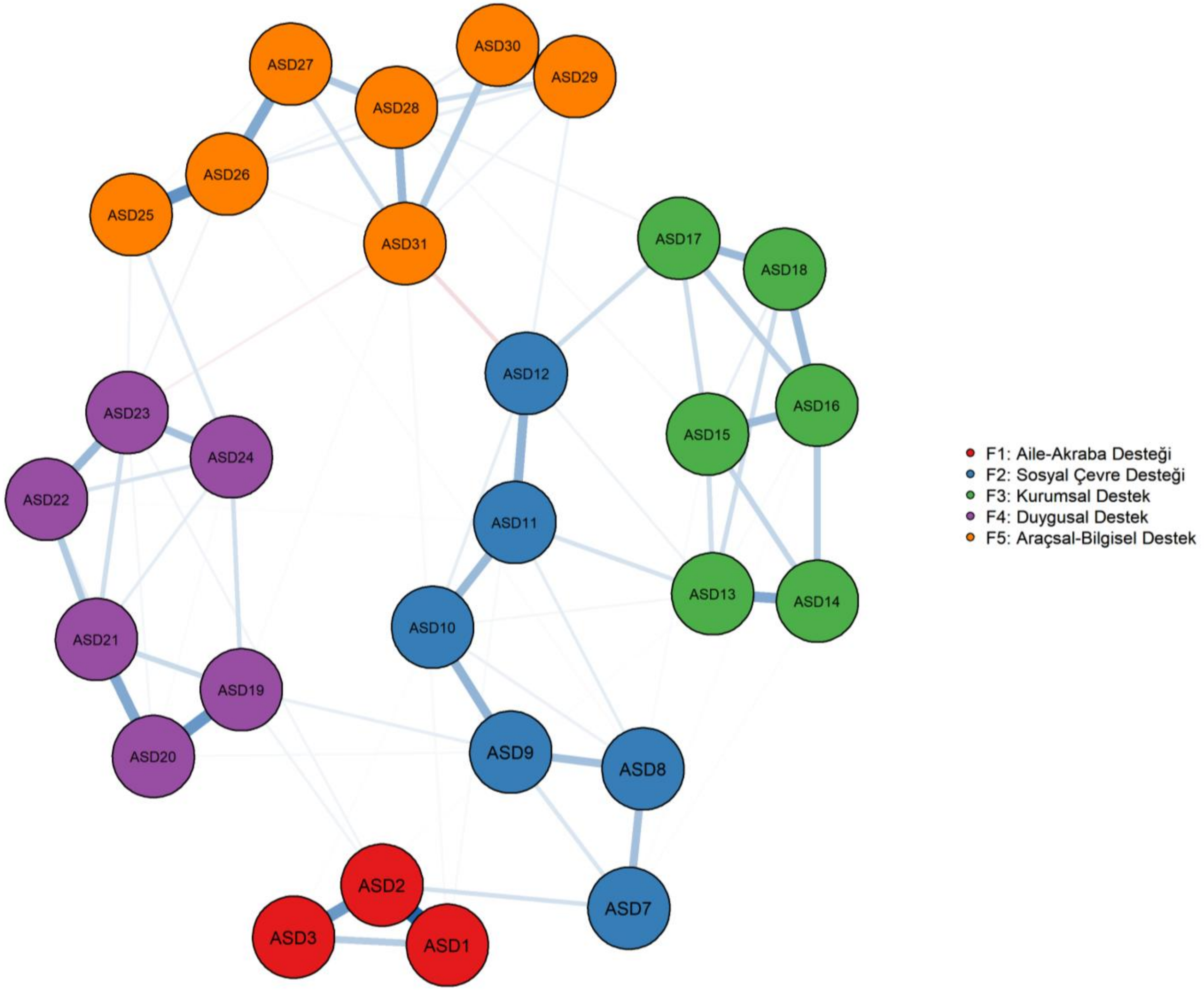


**Figure S16.1.** PSSDS-28 primary network (EBICglasso; γ = 0.50; threshold = FALSE; N_cc = 548).

*Note.* Node colors represent CFA block membership (F1–F5). Edge widths are proportional to partial correlation strength.

## S16.2 Centrality and Bridge Centrality

**Table S16.2.** Node centrality indices (selected items)

| Item | Block | Strength | Closeness | Betweenness | Expected Influence |
|---|---|---|---|---|---|
| asd2 | FRS | 1.378 | 0.0017 | 37 | 1.286 |
| asd31 | IIS | 1.247 | 0.0023 | 80 | 0.594 |
| asd16 | IS | 1.202 | 0.0017 | 6 | 1.202 |
| asd26 | IIS | 1.178 | 0.0019 | 32 | 0.993 |
| asd15 | IS | 1.135 | 0.0016 | 0 | 1.032 |
| asd25 | IIS | 0.791 | 0.0019 | 22 | 0.791 |
| asd3 | FRS | 0.763 | 0.0016 | 0 | 0.763 |
| asd7 | SES | 0.707 | 0.0018 | 17 | 0.707 |

**Table S16.3.** Bridge centrality indices (top 5 items)

| Item | Block | Bridge Strength | Bridge Betweenness | Bridge Expected Influence |
|---|---|---|---|---|
| asd12 | SES | 0.564 | 76 | 0.280 |
| asd31 | IIS | 0.429 | 2 | −0.122 |
| asd13 | IS | 0.353 | 27 | 0.295 |
| asd23 | ES | 0.339 | 31 | 0.169 |
| asd2 | FRS | 0.336 | 39 | 0.245 |

*Note.* Each of the five highest bridge-strength items came from a different block, indicating that cross-block connectivity was distributed across the scale rather than concentrated in any single block.

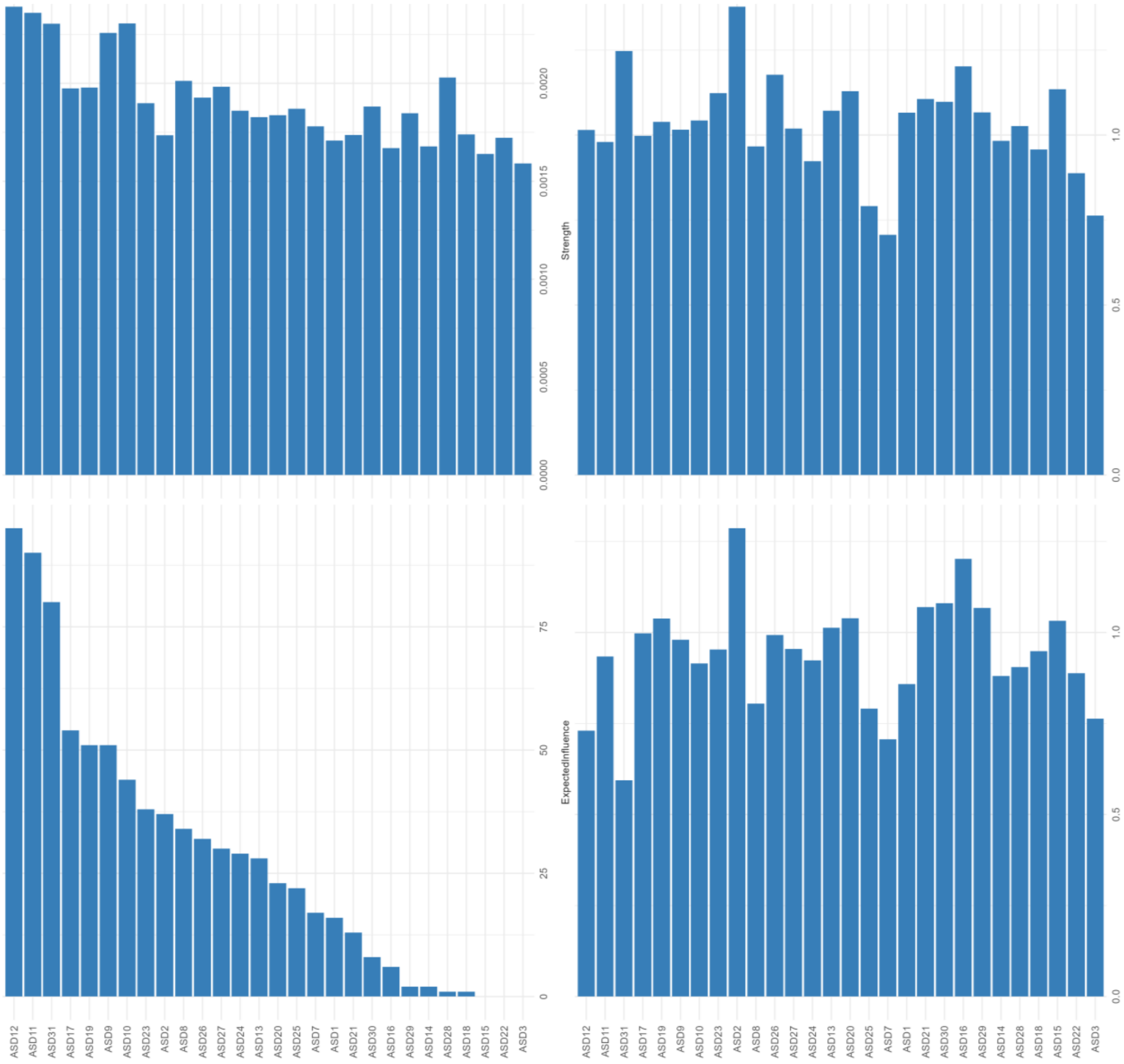


**Figure S16.2.** Centrality indices (Strength, Closeness, Betweenness, Expected Influence).

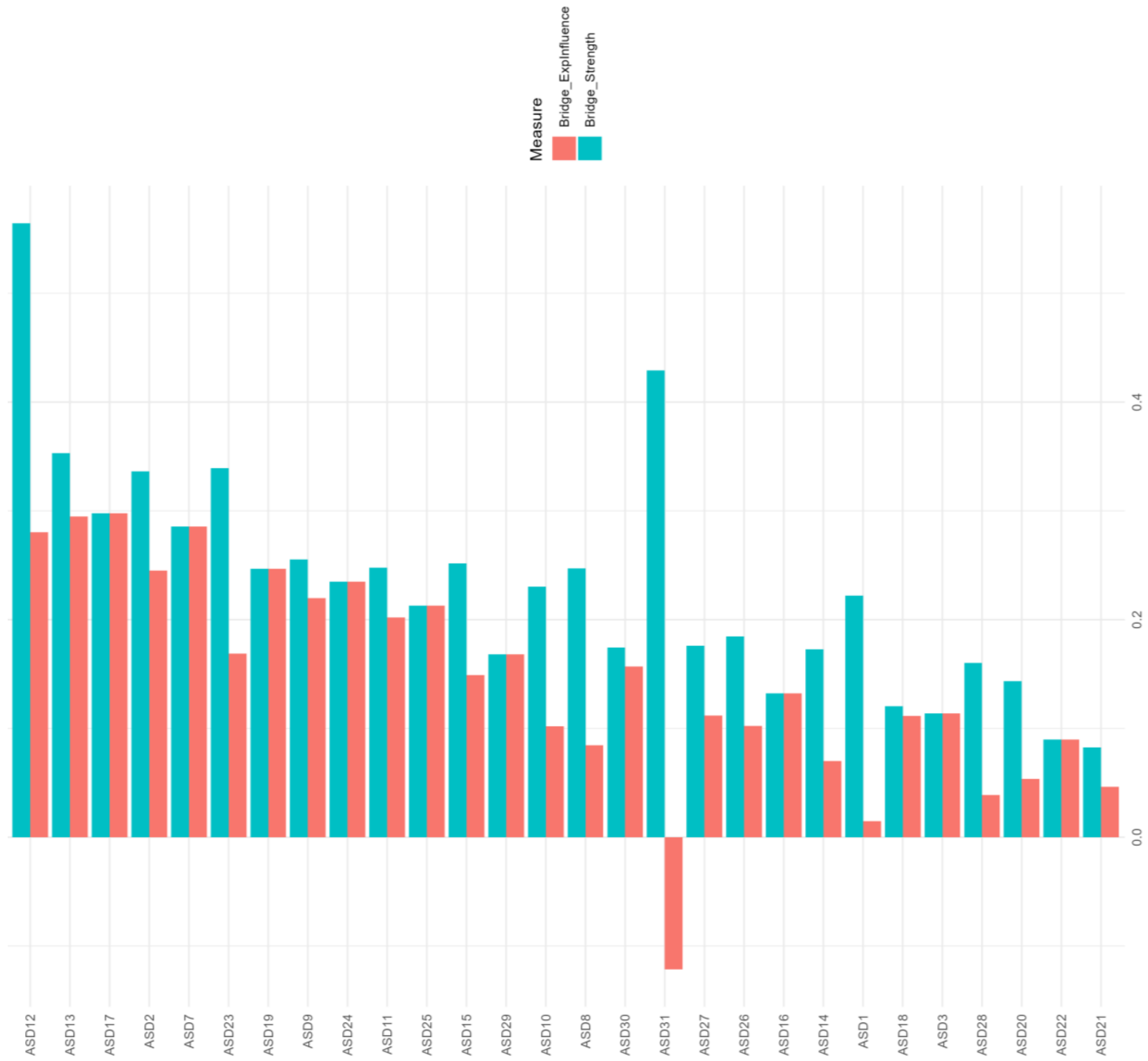


**Figure S16.3.** Bridge centrality indices.

### S16.3 Bootstrap Accuracy and Stability

**Table S16.4.** Centrality stability coefficients (CS; threshold = FALSE)

| Measure | CS coefficient | ≥ .25 (stable) | ≥ .50 (preferred) |
|---|---|---|---|
| Strength | 0.440 | YES | NO |
| Closeness | 0.206 | NO | NO |
| Betweenness | 0.049 | NO | NO |
| Expected Influence | 0.595 | YES | YES |

*Note.* CS coefficients evaluated against the conventional thresholds shown (≥ .25 = stable; ≥ .50 = preferred). Strength and Expected Influence rankings were acceptably stable; Closeness and especially Betweenness should be interpreted with caution.

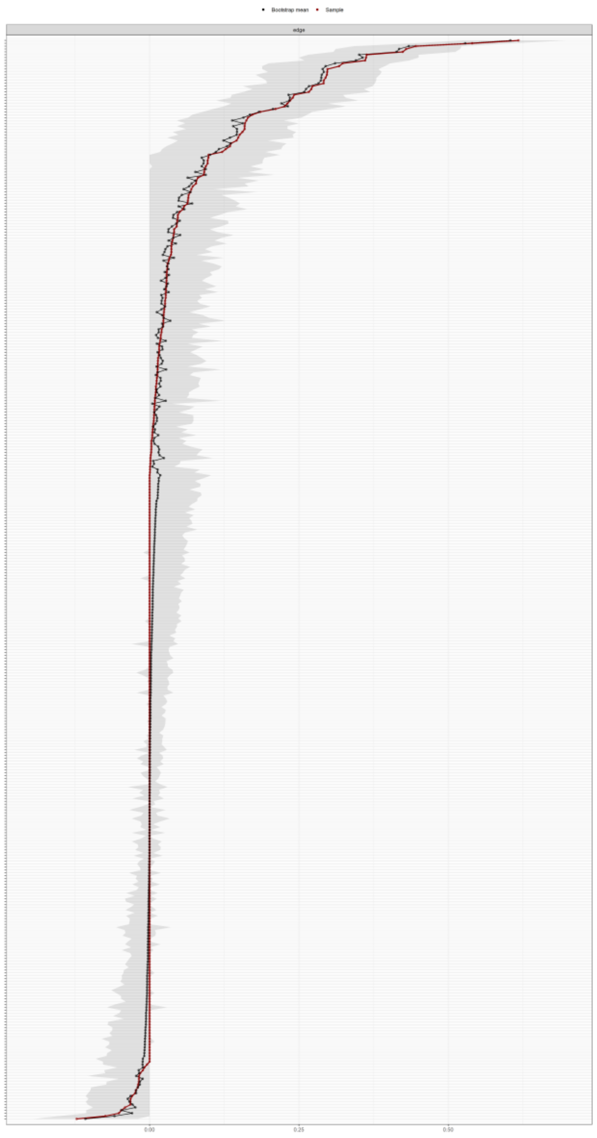


**Figure S16.4.** Edge weight bootstrap accuracy (confidence intervals; B = 500).

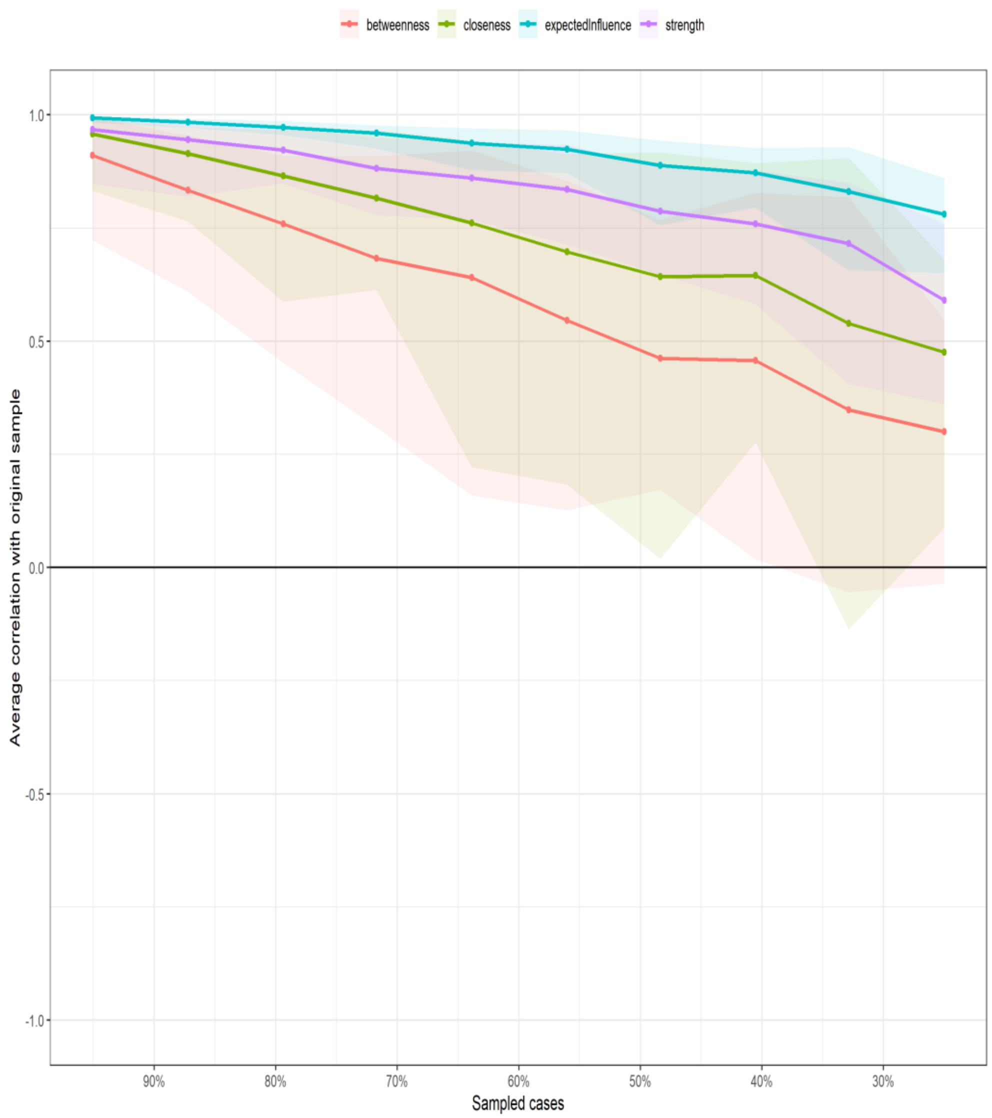


**Figure S16.5.** Centrality stability (case-dropping bootstrap).

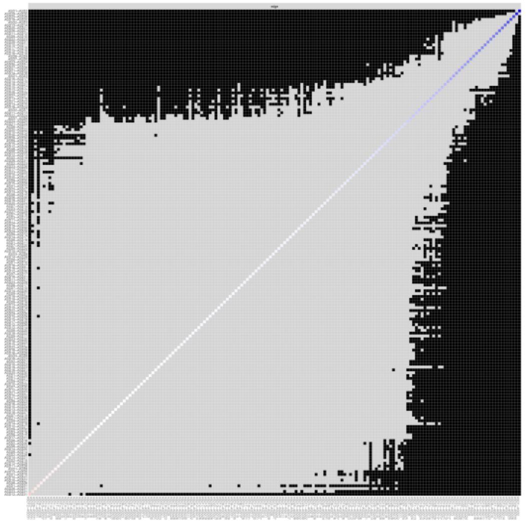

**Figure S16.6.** Bootstrapped edge difference test.

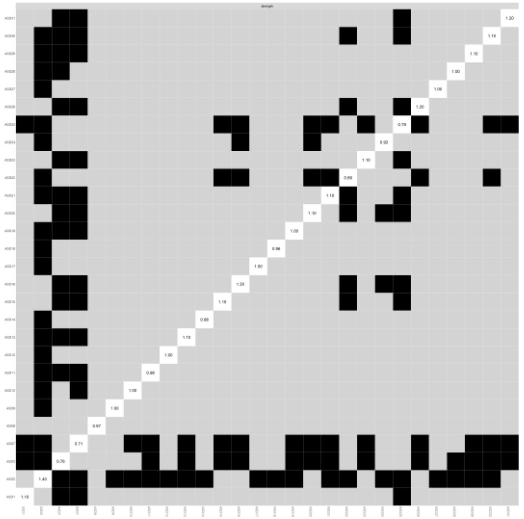

**Figure S16.7.** Bootstrapped centrality (Strength) difference test.

### S16.4 Exploratory Graph Analysis (EGA) and bootEGA

**Table S16.5.** EGA community assignment and CFA block correspondence (full overlap)

| CFA block | Items | EGA community | Overlap |
|---|---|---|---|
| FRS | 3 | 1 | Full (3/3) |
| SES | 6 | 2 | Full (6/6) |
| IS | 6 | 3 | Full (6/6) |
| ES | 6 | 4 | Full (6/6) |
| IIS | 7 | 5 | Full (7/7) |

**Table S16.6.** Network–CFA structural agreement

| Metric | Value |
|---|---|
| Adjusted Rand Index (ARI) | 1.000 |
| Normalized Mutual Information (NMI) | 1.000 |
| CFA dimensions | 5 |
| EGA dimensions | 5 |

**Table S16.7.** bootEGA dimension frequency (B = 500)

| Dimensions | Frequency | Percentage |
|---|---|---|
| 5 | 500 | 100.0 |

*Note.* The five-dimensional solution was reproduced in 100% of 500 bootstrap resamples.

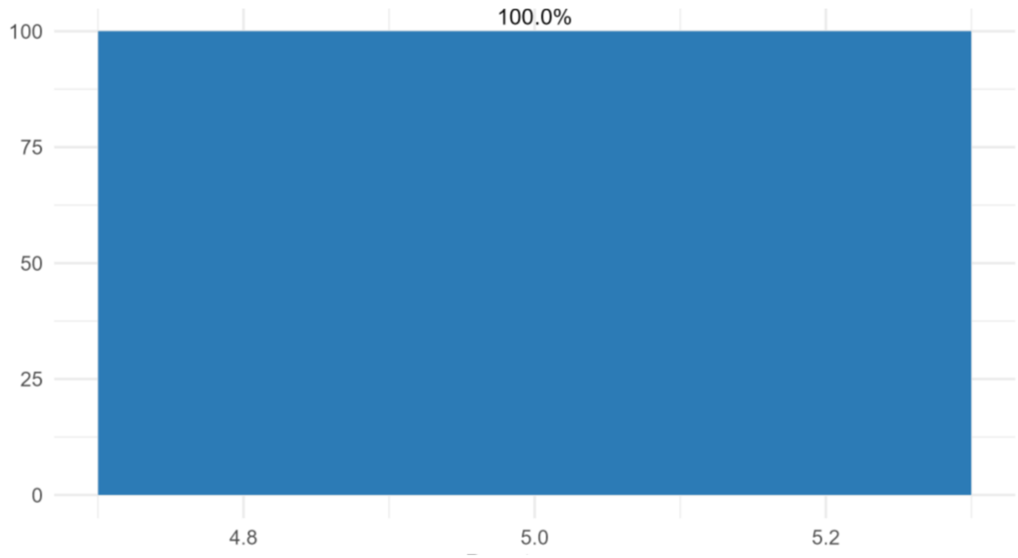


**Figure S16.8.** bootEGA dimension frequency (B = 500).

**S16.5 Sensitivity Analyses: EBIC γ Grid and Split-Half Replication**

**Table S16.8.** EBIC γ sensitivity analysis

| γ | Nonzero edges | Sparsity | Correlation with γ = 0.50 |
|---|---|---|---|
| 0.25 | 172 | 0.545 | 1.000 |
| 0.50 | 172 | 0.545 | 1.000 |
| 1.00 | 147 | 0.611 | 0.994 |

**Table S16.9.** Split-half replication

| Comparison | N | Nonzero edges | Edge weight correlation |
|---|---|---|---|
| Split 1 | 274 | 143 | — |
| Split 2 | 274 | 132 | — |
| Correlation | — | — | 0.884 |

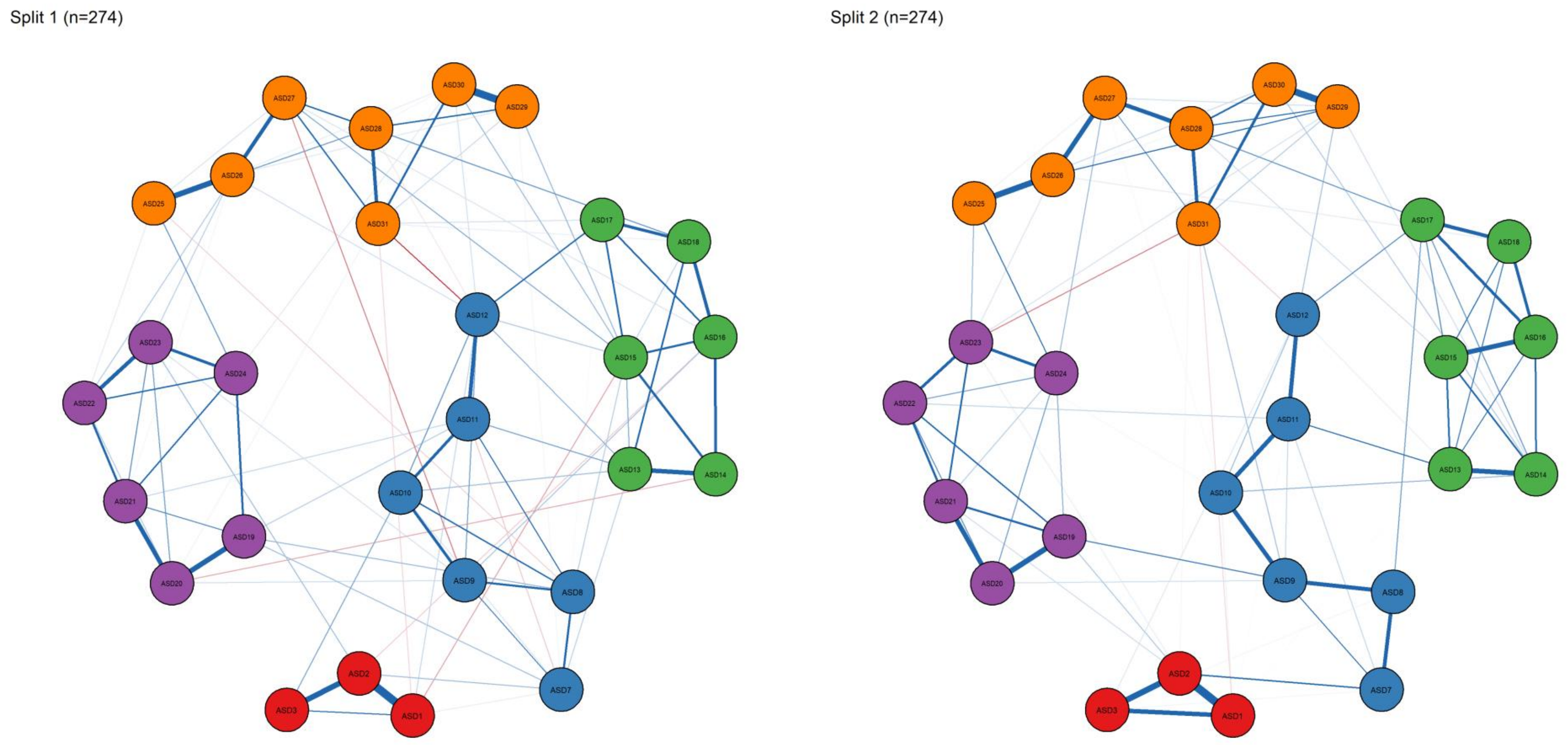


**Figure S16.9.** Split-half network comparison.

**S16.6 Predictability (Nodewise R²)**

**Table S16.10.** Predictability summary by block (mean R²)

| Block | Items | Min R² | Max R² | Mean R² |
|---|---|---|---|---|
| FRS | 3 | 0.588 | 0.744 | 0.678 |
| SES | 6 | 0.339 | 0.434 | 0.375 |

| | | | | |
|---|---|---|---|---|
| IS | 6 | 0.562 | 0.695 | 0.607 |
| ES | 6 | 0.534 | 0.673 | 0.595 |
| IIS | 7 | 0.408 | 0.601 | 0.512 |

*Note.* Overall mean $R^2 = 0.538$ (28 items; unweighted mean of the item-level values above). Predictability values are invariant to the threshold setting because mgm applies its own independent regularization.

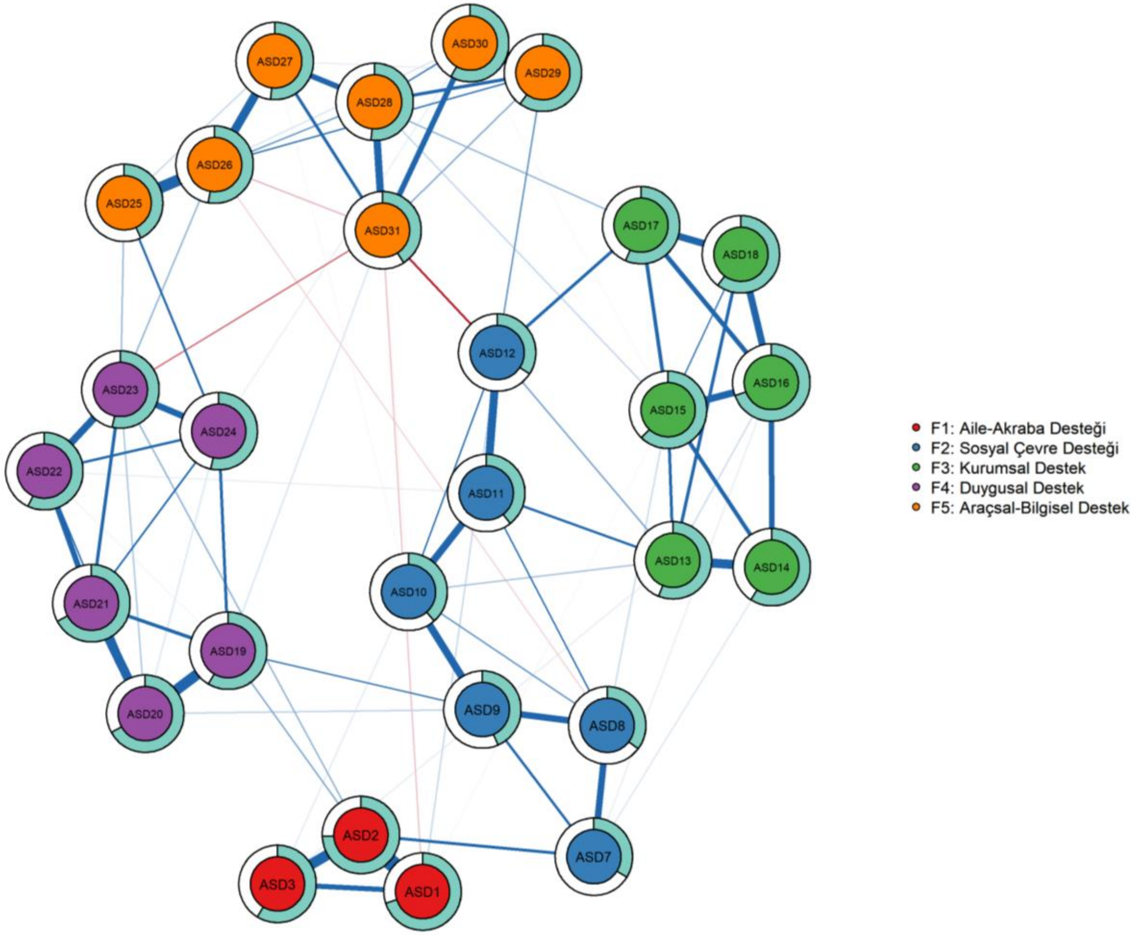


**Figure S16.10.** PSSDS-28 network: predictability (nodewise $R^2$).

### S16.7 Small-World Properties

**Table S16.11.** Small-world diagnostics (binary method; threshold = FALSE)

| Metric | Value |
|---|---|
| Clustering (C) | 0.471 |
| Path length (L) | 1.548 |
| Random C | 0.451 |
| Random L | 1.546 |
| Gamma | 1.043 |
| Lambda | 1.001 |
| Small-World Index (SWI) | 1.041 |
| Components | 1 |

*Note.* SWI ≈ 1.04 indicates no strong small-world property; the observed dense, well-connected topology was close to random-graph expectation, consistent with the relatively low sparsity (0.545).

### S16.8 Redundancy Screening

Goldbricker redundancy screening (correlation threshold = 0.50; difference threshold = 0.25; hittner2003 method) returned no redundant item pair. The archived output does not distinguish a genuine null result from a failure of the procedure to complete, so this is reported as an absence of evidence for item redundancy rather than as positive evidence of its absence.

### S16.9 Supplementary Sensitivity Analysis: threshold = TRUE

**Table S16.12.** threshold = FALSE (primary) vs. threshold = TRUE (sensitivity) comparison

| Metric | threshold = FALSE | threshold = TRUE |
|---|---|---|
| Nonzero edges / possible | 172 / 378 | 63 / 378 |
| Sparsity | 0.545 | 0.833 |
| Components | 1 | 1 |
| SWI | 1.041 | 1.842 |
| CS: Strength | 0.440 (stable) | 0.128 (unstable) |
| CS: Closeness | 0.206 (unstable) | 0.000 (unstable) |
| CS: Betweenness | 0.049 (unstable) | 0.000 (unstable) |
| CS: Expected Influence | 0.595 (stable) | 0.128 (unstable) |
| EGA dimensions | 5 | 5 |
| ARI / NMI | 1.000 / 1.000 | 1.000 / 1.000 |
| bootEGA stability | 100% (5 dim.) | 100% (5 dim.) |

| | | |
|---|---|---|
| Split-half edge correlation | 0.884 | 0.746 |
| Mean predictability $R^2$ | 0.538 | 0.538 (invariant) |

*Note.* Dimensionality-related findings (EGA, ARI/NMI, bootEGA, predictability) were completely invariant to the threshold setting. Centrality stability and structural density indicators were stronger under threshold = FALSE, supporting it as the primary analysis.

## S17. Common Method Variance (CMV) Assessment (Full Sample, N = 607)

### S17.1 Procedural Remedies

Data collection procedures included: (a) emphasis on anonymity/confidentiality, (b) explicit statement that there are no right or wrong answers, (c) encouragement of honest responding, (d) permission to skip items, and (e) rigorous data quality screening (attention checks, missingness thresholds, straightlining detection, speeding rules).

### S17.2 Effective N by Test

**Table S17.1.** CMV test effective N summary

| Test | Items | Missing cells | Complete cases | N total | % Complete |
|---|---|---|---|---|---|
| Test 1 — Harman (37 items) | 37 | 103 | 515 | 607 | 84.84 |
| Tests 2–3 — CFA/CLF (PSSDS-28) | 28 | 62 | 548 | 607 | 90.28 |
| Test 4 — HTMT (PSSDS-28) | 28 | 62 | 548 | 607 | 90.28 |

### S17.3 Test 1: Harman Single-Factor Test (PAF; 37 Items)

**Table S17.2.** Harman single-factor test results (PAF; 37 items)

| Statistic | Value |
|---|---|
| PAF Factor 1 variance explained (%) | 32.04 |
| Polychoric matrix first eigenvalue | 12.508 |
| Eigenvalue / number of items (%) | 33.81 |

*Note.* The first unrotated PAF factor explained 32.04% of variance, well below the 50% heuristic threshold, suggesting no dominant single method factor.

### S17.4 Test 2: CFA Comparison (PSSDS-28; 1-Factor vs. CFA-5)

**Table S17.3.** CFA fit indices (PSSDS-28; WLSMV theta)

| Model | $\chi^2$ | df | p | CFI | TLI | RMSEA | SRMR |
|---|---|---|---|---|---|---|---|
| PSSDS-28 1-factor | 6969.734 | 350 | < .001 | 0.714 | 0.691 | 0.186 | 0.198 |
| PSSDS-28 CFA-5 | 1288.200 | 340 | < .001 | 0.959 | 0.954 | 0.071 | 0.060 |

**Table S17.4.** CFA fit improvement (CFA-5 vs. 1-factor)

| Statistic | Value |
|---|---|
| ΔCFI (CFA-5 − 1F) [scaled] | 0.2451 |
| RMSEA decrease (1F − CFA-5) [scaled] | 0.1145 |

**S17.5 Test 3: Common Latent Factor (CLF) Sensitivity Analysis**

**Table S17.5.** CLF summary (PSSDS-28; CLF vs. CFA-5)

| Statistic | Value |
|---|---|
| ΔCFI (CLF − CFA-5) [scaled] | 0.00090 |
| ΔRMSEA (CLF − CFA-5) [scaled] | −0.00068 |
| Mean Δλ | 0.0200 |
| Max Δλ | 0.0393 |
| m (unstandardized) | 0.300 |
| m p-value | < .001 |
| Mean CLF loading (std.all) [descriptive] | 0.169 |

*Note.* The CLF added negligible explanatory power beyond CFA-5 (ΔCFI ≈ .001; mean |Δλ| = .020). This test is treated as a sensitivity diagnostic because a common factor within a single instrument's item set cannot conceptually distinguish method variance from general severity variance (Podsakoff et al., 2003; Williams et al., 2010).

**Table S17.6.** CLF loading differences by block (summary)

| Block | Items | Min \|Δλ\| | Max \|Δλ\| | Mean \|Δλ\| |
|---|---|---|---|---|
| FRS | 3 | 0.0089 | 0.0171 | 0.0120 |
| SES | 6 | 0.0224 | 0.0370 | 0.0299 |
| IS | 6 | 0.0049 | 0.0219 | 0.0147 |
| ES | 6 | 0.0064 | 0.0254 | 0.0145 |
| IIS | 7 | 0.0103 | 0.0393 | 0.0240 |

**S17.6 Test 4: HTMT (PSSDS-28 Blocks)**

**Table S17.7.** HTMT matrix (PSSDS-28 blocks)

| | FRS | SES | IS | ES | IIS |
|---|---|---|---|---|---|
| FRS | — | 0.572 | 0.232 | 0.503 | 0.152 |
| SES | | — | 0.616 | 0.606 | 0.352 |
| IS | | | — | 0.379 | 0.627 |

| | | |
|---|---|---|
| ES | — | 0.474 |
| IIS | | — |

*Note.* All HTMT values are well below the .85/.90 thresholds, supporting discriminant validity and mitigating concerns that block relationships are dominated by a single method factor. These ratios were computed listwise on the complete-case subset (N_cc = 548), whereas Table S14.3 uses the full sample with pairwise polychoric estimation and positive-definite smoothing (N = 607); the two sets therefore differ by up to .012 (SES–IIS: .352 here versus .364 there). The main text reports the Table S14.3 values throughout.

## PART V: ADDITIONAL AND SENSITIVITY ANALYSES

## S18. Additional Analyses

### S18.1 Differential Item Functioning (DIF)

DIF was tested using proportional-odds ordinal logistic regression (clm, logit link) for each PSSDS-28 item across the six grouping variables for which measurement invariance was supported (age group, gender, marital status, education, employment, psychological support). Each item used the mean of the remaining items in its CFA block (excluding the focal item) as the matching criterion. Three nested models were estimated per item × variable: M0 (item ~ matching score), M1 (+ group; uniform DIF), M2 (+ group × matching score; non-uniform DIF). Decision criteria combined Holm-adjusted p values and practical DIF magnitude ($\Delta$McFadden $R^2 \geq .010$; Crane et al., 2006).

**Table S18.1.** DIF results summary by grouping variable

| Grouping variable | Items tested | Practical flags | Max $\Delta R^2$ (uniform) | Max $\Delta R^2$ (non-uniform) | Min p_Holm (uniform) | Min p_Holm (non-uniform) |
|---|---|---|---|---|---|---|
| Age group | 28 | 0 | 0.00359 | 0.00095 | 0.346 | 1.000 |
| Gender | 28 | 0 | 0.00451 | 0.00437 | 0.119 | 0.267 |
| Marital status | 28 | 0 | 0.00635 | 0.00386 | 0.127 | 1.000 |
| Education | 28 | 0 | 0.00844 | 0.00654 | 0.041 | 0.377 |
| Employment | 28 | 0 | 0.00933 | 0.00712 | 0.130 | 0.336 |
| Psychological support | 28 | 0 | 0.00373 | 0.00509 | 0.360 | 0.067 |

*Note.* Item-specific complete-case rule applied (n = 584–605). No item × group combination exceeded the $\Delta R^2 \geq .010$ practical threshold. One item (asd26, education) reached Holm-corrected statistical significance (p_Holm = .041), but its $\Delta R^2$ (.00844) remained below the practical threshold—a pattern expected in large-sample settings where statistical power exceeds practical effect size.

**Table S18.2.** Largest DIF magnitudes by grouping variable (diagnostic scan)

| Grouping variable | Item | Block | n | $\Delta R^2$ (uniform) | $\Delta R^2$ (non-uniform) | p_Holm (uniform) | Signal type |
|---|---|---|---|---|---|---|---|
| Age group | asd7 | SES | 598 | 0.00359 | 0.00081 | 0.346 | Max uniform |
| Age group | asd29 | IIS | 584 | 0.00000 | 0.00095 | 1.000 | Max non-uniform |
| Gender | asd25 | IIS | 584 | 0.00451 | 0.00124 | 0.119 | Max uniform |
| Gender | asd3 | FRS | 605 | 0.00021 | 0.00437 | 1.000 | Max non-uniform |
| Marital status | asd10 | SES | 598 | 0.00635 | 0.00002 | 0.127 | Max uniform |
| Marital status | asd1 | FRS | 605 | 0.00387 | 0.00386 | 1.000 | Max non-uniform |
| Education | asd26 | IIS | 584 | 0.00844 | 0.00263 | 0.041 | Max uniform |
| Education | asd23 | ES | 591 | 0.00200 | 0.00654 | 1.000 | Max non-uniform |
| Employment | asd1 | FRS | 605 | 0.00933 | 0.00658 | 0.130 | Max uniform |
| Employment | asd3 | FRS | 605 | 0.00174 | 0.00712 | 1.000 | Max non-uniform |
| Psych. support | asd22 | ES | 591 | 0.00372 | 0.00024 | 0.360 | Max uniform |
| Psych. support | asd25 | IIS | 584 | 0.00012 | 0.00509 | 1.000 | Max non-uniform |

### S18.2 Score Distributions and Percentile Values

**Table S18.3.** Score distributions and percentile values (mean-score metric; range 1–5)

| Scale | N | Mean | SD | Median | Q1 | Q3 | P10 | P50 | P90 | Min | Max |
|---|---|---|---|---|---|---|---|---|---|---|---|
| Total | 607 | 3.518 | 0.760 | 3.607 | 3.000 | 4.073 | 2.464 | 3.607 | 4.500 | 1.143 | 4.893 |
| FRS | 605 | 4.174 | 1.097 | 4.667 | 3.667 | 5.000 | 2.667 | 4.667 | 5.000 | 1.000 | 5.000 |
| SES | 607 | 3.769 | 0.902 | 3.833 | 3.333 | 4.500 | 2.600 | 3.833 | 5.000 | 1.000 | 5.000 |
| IS | 607 | 3.294 | 1.165 | 3.500 | 2.500 | 4.167 | 1.500 | 3.500 | 4.833 | 1.000 | 5.000 |
| ES | 607 | 3.777 | 1.031 | 4.000 | 3.167 | 4.667 | 2.333 | 4.000 | 5.000 | 1.000 | 5.000 |
| IIS | 607 | 2.989 | 1.056 | 3.000 | 2.143 | 3.857 | 1.571 | 3.000 | 4.286 | 1.000 | 5.000 |

**Table S18.4.** Score distributions and percentile values (sum-score metric)

| Scale | N | Mean | SD | Median | Q1 | Q3 | P10 | P50 | P90 | Min | Max |
|---|---|---|---|---|---|---|---|---|---|---|---|
| Total | 607 | 98.132 | 21.143 | 101 | 84.0 | 114.0 | 69.0 | 101 | 125 | 32 | 137 |
| FRS | 605 | 12.522 | 3.292 | 14 | 11.0 | 15.0 | 8.0 | 14 | 15 | 3 | 15 |
| SES | 607 | 22.552 | 5.401 | 23 | 19.5 | 26.5 | 15.6 | 23 | 30 | 6 | 30 |
| IS | 607 | 19.700 | 6.974 | 20 | 15.0 | 25.0 | 9.0 | 20 | 29 | 6 | 30 |
| ES | 607 | 22.565 | 6.200 | 24 | 19.0 | 28.0 | 14.0 | 24 | 30 | 6 | 30 |
| IIS | 607 | 20.801 | 7.373 | 21 | 15.0 | 27.0 | 10.6 | 21 | 30 | 6 | 35 |

*Note.* Sum-score ranges: FRS (3–15), SES (6–30), IS (6–30), ES (6–30), IIS (7–35), Total (28–140).

**Table S18.5**. Measurement error (SEM) and minimal detectable change (MDC_95; mean-score metric; α-based)

| Scale | Items | α | SD (mean metric) | SEM | MDC_95 |
|---|---|---|---|---|---|
| FRS | 3 | 0.949 | 1.097 | 0.249 | 0.689 |
| SES | 6 | 0.852 | 0.902 | 0.347 | 0.961 |
| IS | 6 | 0.937 | 1.165 | 0.291 | 0.808 |
| ES | 6 | 0.939 | 1.031 | 0.255 | 0.706 |
| IIS | 7 | 0.900 | 1.056 | 0.335 | 0.928 |
| Total | 28 | 0.940 | 0.760 | 0.186 | 0.516 |

*Note.* SEM = SD × √(1 − α); MDC_95 = 1.96 × √2 × SEM. The total-score coefficient (α = .940) is the 28-item ordinal alpha from the score-distribution run and is reported here only to derive the total SEM and MDC_95; these totals are a practical aid for users computing an overall mean score and do not imply strict unidimensionality. Primary interpretation is intended at the block (factor) level. These values are alpha-based and describe the precision of a single administration; for judging whether an observed change over time exceeds measurement error, use the retest-based SEM and MDC_95 reported in Table S13.4.

### S18.3 Estimator Sensitivity: WLSMV vs. MLR (Validation Subsample)

**Table S18.6.** Estimator sensitivity: CFA-5 model fit under WLSMV and MLR (validation subsample; N = 302)

| Estimator | N | $\chi^2$ | df | p | CFI | TLI | RMSEA | SRMR |
|---|---|---|---|---|---|---|---|---|
| WLSMV (ordinal; theta) | 302 | 927.725 | 340 | < .001 | 0.954 | 0.949 | 0.076 | 0.072 |
| MLR (continuous; robust) | 302 | 948.582 | 340 | < .001 | 0.899 | 0.888 | 0.072 | 0.062 |

*Note.* Incremental indices (CFI, TLI) were markedly higher under WLSMV; absolute indices (RMSEA, SRMR) were similar across estimators. This pattern reflects the known statistical consequence of treating ordinal items as continuous under MLR (Rhemtulla et al., 2012).

**Table S18.7.** Standardized loading stability summary (WLSMV vs. MLR; validation subsample)

| Items | Mean \|Δλ\| | Median \|Δλ\| | Max \|Δλ\| | \|Δλ\| > .05 (*n*) | \|Δλ\| > .10 (*n*) | \|Δλ\| > .20 (*n*) | *r* (λ_WLSMV, λ_MLR) |
|---|---|---|---|---|---|---|---|
| 28 | 0.0602 | 0.0556 | 0.1565 | 17 | 3 | 0 | 0.9377 |

**Table S18.8.** Loading differences by block (WLSMV vs. MLR)

| Block | Items | Min \|Δλ\| | Max \|Δλ\| | Mean \|Δλ\| |
|---|---|---|---|---|
| FRS | 3 | 0.0346 | 0.0657 | 0.0527 |
| SES | 6 | 0.0248 | 0.1124 | 0.0748 |
| IS | 6 | 0.0137 | 0.0740 | 0.0401 |

| | | | | |
|---|---|---|---|---|
| ES | 6 | 0.0259 | 0.0898 | 0.0592 |
| IIS | 7 | 0.0071 | 0.1565 | 0.0689 |

*Note.* All Δλ values were negative (MLR < WLSMV), indicating systematic attenuation under continuous treatment. Largest shifts concentrated in SES and IIS blocks.

### S18.4 Construct Replicability and Factor Score Quality (CR / H / Factor Determinacy)

**Table S18.9.** Construct replicability and factor score quality (PSSDS-28; full sample, N = 607)

| Factor | Subscale | Items | CR | H | Factor determinacy (MLR) |
|---|---|---|---|---|---|
| FRS | Family–Relative Support (asd1–asd3) | 3 | 0.9499 | 0.9716 | 0.9700 |
| SES | Social Environment Support (asd7–asd12) | 6 | 0.8673 | 0.8709 | 0.9230 |
| IS | Institutional Support (asd13–asd18) | 6 | 0.9409 | 0.9443 | 0.9643 |
| ES | Emotional Support (asd19–asd24) | 6 | 0.9420 | 0.9446 | 0.9627 |
| IIS | Instrumental–Informational Support (asd25–asd31) | 7 | 0.9121 | 0.9217 | 0.9468 |

*Note.* CR and H computed from ordinal CFA-5 (WLSMV; theta) within the construct-replicability run, which does not set missing = pairwise; the CR values therefore differ in the third decimal from Table S13.3 (SES .867 here versus .865 there). The main text reports the Table S13.3 values. Factor determinacy (MLR) computed from continuous-treatment CFA-5 (FIML) as primary; WLSMV-based determinacy used for sensitivity triangulation (see Table S18.10).

**Table S18.10.** Sensitivity triangulation: determinacy (WLSMV) and IRT EAP reliability

| Factor | Items | Factor determinacy (WLSMV) | n | EAP reliability |
|---|---|---|---|---|
| FRS | 3 | 0.9859 | 607 | 0.7350 |
| SES | 6 | 0.9442 | 607 | 0.7934 |
| IS | 6 | 0.9733 | 607 | 0.9032 |
| ES | 6 | 0.9732 | 607 | 0.8858 |
| IIS | 7 | 0.9624 | 607 | 0.8736 |

*Note.* The lowest EAP reliability was observed for FRS (.735), consistent with its short block length (k = 3); this does not contradict FRS's highest CR (.950) and H (.972), as CFA-based and IRT-based indices measure different aspects of reliability.

### S18.5 Local Dependence / Residual Covariance Sensitivity (CFA; WLSMV-Theta)

**Table S18.11.** Within-block residual correlation scan — pairs exceeding threshold (|residual r| ≥ .10)

| Item i | Item j | Block | Residual *r* | \|Residual *r*\| |
|---|---|---|---|---|
| asd26 | asd31 | IIS | −0.1934 | 0.1934 |
| asd25 | asd29 | IIS | −0.1889 | 0.1889 |
| asd25 | asd30 | IIS | −0.1849 | 0.1849 |

| | | | | |
|---|---|---|---|---|
| asd28 | asd31 | IIS | 0.1676 | 0.1676 |
| asd25 | asd31 | IIS | −0.1637 | 0.1637 |
| asd26 | asd30 | IIS | −0.1630 | 0.1630 |
| asd7 | asd11 | SES | −0.1557 | 0.1557 |
| asd7 | asd12 | SES | −0.1514 | 0.1514 |
| asd25 | asd26 | IIS | 0.1451 | 0.1451 |
| asd27 | asd30 | IIS | −0.1311 | 0.1311 |
| asd26 | asd29 | IIS | −0.1304 | 0.1304 |
| asd9 | asd12 | SES | −0.1275 | 0.1275 |
| asd7 | asd8 | SES | 0.1041 | 0.1041 |
| asd27 | asd29 | IIS | −0.1022 | 0.1022 |
| asd8 | asd12 | SES | −0.1018 | 0.1018 |

*Note.* 15 of 69 within-block pairs exceeded the |residual r| ≥ .10 threshold; 10 were in IIS, 5 in SES; 0 in FRS, IS, or ES. Bold rows indicate the three pairs freed in the sensitivity model (greedy no-item-repeat rule): asd26–asd31, asd25–asd29, and asd7–asd11.

**Table S18.12.** Baseline vs. residual covariance sensitivity model comparison

| Model | N | $\chi^2$ | df | CFI | TLI | RMSEA | SRMR |
|---|---|---|---|---|---|---|---|
| Baseline CFA-5 | 548 | 1288.200 | 340 | 0.959 | 0.954 | 0.071 | 0.060 |
| CFA-5 + residual covariance (k = 3) | 548 | 1215.393 | 337 | 0.962 | 0.957 | 0.069 | 0.059 |

*Note.* ΔCFI = +.003; ΔRMSEA = −.002; ΔSRMR = −.001 (sensitivity − baseline). Improvement was small and the baseline model already showed good fit.

**Table S18.13.** Loading stability summary (baseline vs. sensitivity model)

| Items | Mean \|Δλ\| | Median \|Δλ\| | Max \|Δλ\| | \|Δλ\| > .05 (*n*) | \|Δλ\| > .10 (*n*) | *r* (λ_baseline, λ_sensitivity) |
|---|---|---|---|---|---|---|
| 28 | 0.0041 | 0.0000 | 0.0243 | 0 | 0 | 0.9964 |

**Table S18.14.** Loading differences by block (baseline vs. sensitivity model)

| Block | Items | Max \|Δλ\| | Mean \|Δλ\| |
|---|---|---|---|
| FRS | 3 | 0.0000 | 0.0000 |
| SES | 6 | 0.0189 | 0.0060 |
| IS | 6 | 0.0000 | 0.0000 |
| ES | 6 | 0.0000 | 0.0000 |
| IIS | 7 | 0.0243 | 0.0112 |

*Note.* Loading shifts were negligible (mean |Δλ| = .004; max = .024); no item exceeded |Δλ| > .05. Changes were confined to blocks with freed residual covariances (SES and IIS).

### S18.6 Item 31 Sensitivity: Seven-Item versus Six-Item Instrumental–Informational Support

The Discussion recommends that item 31 (asd31, "I was able to obtain legal counselling") be reported as a standalone descriptive indicator in applied needs-assessment settings, in addition to its contribution to the instrumental–informational support (IIS) composite. Because an earlier formulation of that recommendation could be read as proposing that the item be excluded from the composite, and because all psychometric properties reported in this work were estimated on the seven-item block, this section reports what would follow if the item were in fact removed. The analysis was requested during peer review and is provided so that prospective users can evaluate the scoring rule against evidence rather than assertion.

Estimation followed the settings used throughout this work. Internal consistency was computed as ordinal alpha from the polychoric correlation matrix, with 95% confidence intervals from 500 case-resampling bootstrap replications; omega total was obtained from a one-factor polychoric solution; corrected item–rest correlations were computed within block from the polychoric matrix. Test–retest estimates used ICC(2,1) with two-way random effects and absolute agreement on the retest subsample, with block scores computed as item means over available responses. Heterotrait–monotrait ratios were computed from polychoric correlations. A fixed seed (20240315) was used for all resampling. Every seven-item value was re-estimated within this run before the six-item values were computed, so that the two columns rest on the same estimation and the same retest subsample. The re-estimated values reproduced those in Table S13.2, Table S14.3 and Table S18.2 exactly. The two retest quantities did not: the retest analysis in this run applies listwise deletion to the paired block scores and therefore uses n = 87, whereas the main reliability analysis in Table S13.4 computes block scores as means over available responses and uses n = 89. Both columns of Table S18.15 are therefore reported from the present run; the corresponding Table S13.4 values for the seven-item block are SEM = .251 and $MDC_{95}$ = .696.

Table S18.15 reports the comparison. Removing item 31 lowered internal consistency and temporal stability, widened the standard error of measurement and the minimal detectable change, left discriminant separation from the institutional block essentially unchanged, and raised the block mean. The mean shift is the substantively important result: item 31 has the highest floor rate of any item in the instrument (38.35%; Table S9.1), so removing it from the composite raises the block score by removing the clearest available indicator of unmet need. No criterion examined here favours the six-item composite. All seven items are therefore retained.

**Table S18.15.** Sensitivity of the instrumental–informational support block to the exclusion of item 31 (full sample, N = 607; retest subsample n = 87).

| Indicator | IIS as reported (7 items) | IIS without item 31 (6 items) | Change |
|---|---|---|---|
| Ordinal α [95% CI] | .900 [.884, .913] | .893 [.873, .908] | −.007 |
| ω total | .901 | .893 | −.008 |
| Mean inter-item correlation (polychoric) | .561 | .581 | +.020 |
| Corrected item–rest correlation (range) | .605–.761 | .632–.745 | — |
| Test–retest ICC(2,1) | .892 | .871 | −.021 |
| Retest SEM | .255 | .293 | +.038 |

| | | | |
|---|---|---|---|
| Retest $MDC_{95}$ | .706 | .813 | +.107 |
| HTMT with the institutional block | .631 | .634 | +.003 |
| Block mean (1–5) | 2.989 | 3.077 | +0.088 |
| Block SD | 1.056 | 1.080 | +0.024 |

*Note.* SEM = standard error of measurement; $MDC_{95}$ = minimal detectable change at the 95% confidence level; HTMT = heterotrait–monotrait ratio. Ordinal α, ω total, corrected item–rest correlations and HTMT ratios are polychoric-based (full sample, N = 607). Test–retest estimates are ICC(2,1), two-way random effects, absolute agreement, computed within the present run with listwise deletion on the paired block scores (n = 87; interval M ≈ 21 days). The main reliability analysis in Table S13.4 uses block scores averaged over available responses (n = 89) and therefore reports SEM = .251 and $MDC_{95}$ = .696 for the seven-item block; both columns here are reported from the present run so that they rest on the same subsample. Confidence intervals for ordinal α are 95% bootstrap intervals (B = 500). Apart from the two retest quantities noted above, the seven-item column reproduces the values reported in Table S13.2, Table S14.3 and Table S18.2. IIS = instrumental–informational support.

**Table S18.16.** Psychometric profile of item 31 (asd31) as a standalone indicator (full sample, N = 607).

| **Indicator** | **Value** | **Source** |
|---|---|---|
| Item wording (English) | "I was able to obtain legal counselling." | S20, A5.1 |
| N with valid response | 605 | Table S9.1 |
| Mean (SD) | 2.460 (1.432) | Table S9.1 |
| Median | 2 | Table S9.1 |
| Skewness / kurtosis | 0.463 / −1.179 | Table S9.1 |
| Floor rate (response = 1) | 38.35% | Table S9.1 |
| Ceiling rate (response = 5) | 12.07% | Table S9.1 |
| Corrected item–rest r (within IIS block) | .633 | Table S13.2 |
| Alpha-if-deleted (IIS block) | .893 (block α = .900) | Table S13.2 |
| Corrected item–total r (against the 31-item pool) | .345 | Table S9.2 |
| Standardized CFA loading (Model B) | .581 | Table S11.2 |
| GRM discrimination (a) | 1.875 | Table S15.4 |
| GRM thresholds ($b_1$–$b_4$) | −0.395, 0.164, 0.794, 1.560 | Table S15.4 |

*Note.* Two corrected-correlation coefficients are reported because they are defined against different reference sets. The within-block coefficient (.633) correlates the item with the remaining six IIS items and is the relevant index for block-level reliability; the pool-level coefficient (.345) correlates the item with the remaining 30 items of the 31-item administered pool and is reported in Table S9.2 for completeness. Item 31 is the only item whose removal would raise the

alpha of the full 31-item pool, and the margin is +.0005 (Table S9.2). At the block level the direction reverses: removing item 31 lowers the IIS coefficient from .900 to .893 (Table S13.2), which is the result reported in Table S18.15. GRM = graded response model.

Read together, the two tables support the scoring rule stated in the main text. Item 31 is the weakest item in the instrument on loading, discrimination and item–rest correlation, and it is the only item whose removal marginally raises its block's alpha. It is nonetheless retained, because the margin is negligible, because every block-level index deteriorates when it is removed, and because it is the only item in any existing perceived-support instrument that addresses access to legal aid following displacement and bereavement. Its high floor rate is not a defect to be corrected by deletion; it is the substantive finding that the item exists to detect.

**S19. Consolidated Robustness Summary (Cross-Method Triangulation Map)**

**Table S19.1.** Consolidated robustness check summary (cross-method triangulation; PSSDS-28)

| Domain | Analysis | What was varied/checked | Robustness outcome | Primary evidence (existing) |
|---|---|---|---|---|
| Data integrity | QC screening and exclusions | Attention checks; missingness; straightlining; speed | High integrity; 9.67% excluded; N = 607 retained | S5, Tables S5.2–S5.3 |
| Missingness | Missingness overview + MCAR | Item pool missingness; Little's MCAR; predictors | Negligible missingness; MCAR not rejected | S7, Tables S7.1–S7.4 |
| Dimensionality | Polychoric EFA retention (31 items) | PA, MAP, scree, Kaiser; fit comparisons | PA/MAP/Kaiser suggested k = 7; scree + theory supported k = 5 | S10.3, Tables S10.3–S10.4 |
| Dimensionality | EGA dimensionality + bootstrap (31 → 28 items) | GLASSO; Walktrap vs. Leiden; bootstrap | 31 items: asd4/5/6/7 low stability (.390–.645); 28 items: full alignment, stability .965–1.000 | S10.7–S10.8 and S10.16–S10.17, Tables S10.14–S10.18 and S10A.16–S10A.21 |
| Convergence | EFA ↔ EGA alignment (31 → 28 items) | Item membership agreement | 31 items: 83.9% agreement; 28 items: 67.9% (SES/IS EFA merger) | S10.18–S10.19, Tables S10A.22–S10A.24 and S10B.1 |
| Item set | Calibration-based item reduction | Pilot + calibration exploratory signals | asd4/asd5/asd6 removed based on convergent triangulation | S10.9, Table S10.19 |
| Cross-validation | Stratified split-sample | Stratified 50/50 split + equivalence tests | Subsamples equivalent; all Bonferroni-corrected comparisons non-significant | S8, Tables S8.1–S8.3 |
| Fit stability | Bootstrap CFA stability | Fit index and loading distributions | Model A: 100% convergence; Model B: 97.5% (CFA-5) / 99.0% (ESEM) | S11.8, Table S11.11 |

| | | | | |
|---|---|---|---|---|
| Parameter stability | Bootstrap EFA stability | EFA loading congruence | Moderate-to-high stability (mean congruence = .859, SD = .082) | S10.6, Table S10.13 |
| Measurement models | Competing CFA models | 1-factor vs. CFA-5 vs. second-order vs. bifactor | CFA-5 best fit (Model B: CFI = .954); alternatives weaker | S11.1, Table S11.1 |
| Cross-loadings | ESEM triangulation | Target-rotation ESEM (Geomin not converged) | Cross-loading burden reduced from 54.8% to 39.3% (≥ .20 secondary) after item removal | S11.7, Tables S11.9–S11.10 |
| Invariance | Ordinal MGCFA (theta) | 9 grouping variables; configural → metric → scalar | 6/9 fully invariant (metric + scalar); 3/9 configural EMPTY_CATEGORY_FAIL | S12, Tables S12.2–S12.3 |
| Item-level bias | DIF | Uniform/non-uniform; Holm + $\Delta R^2 \geq .010$ | No practical DIF flag in any item × group combination (168/168 tested) | S18.1, Tables S18.1–S18.2 |
| CMV | Harman + CFA + CLF + HTMT | Single-factor scans; CLF; HTMT | No dominant method factor; HTMT < .85 | S17, Tables S17.2–S17.7 |
| Complementary | IRT / Mokken (block-level) | GRM vs. GPCM; M2/S-$X^2$; local dependence | High discrimination (asd2 excepted); within-block local dependence (short-block limitation) | S15, Tables S15.3–S15.9 |
| Network psychometrics | Network structure | EBICglasso; γ sensitivity; EGA stability | Block-clustered structure, stable EGA communities (ARI = NMI = 1.000); centrality partially stable (CS = .049–.595) | S16, Tables S16.1–S16.12 |
| Estimator | WLSMV vs. MLR | CFA-5 fit + loading stability | Notable incremental-index divergence (ΔCFI = −.055); loading pattern moderately-to-highly stable (r = .938) | S18.3, Tables S18.6–S18.8 |
| Score quality | CR / H / determinacy | Composite reliability; replicability; EAP | CR/H good-to-excellent (.867–.950 / .871–.972); determinacy excellent (.923–.970) | S18.4, Tables S18.9–S18.10 |
| Local dependence | Residual correlation scan | Within-block residual r scan (threshold .10) | 15/69 pairs exceeded threshold (10 in IIS, 5 in SES); freeing improved fit minimally (ΔCFI = +.003); loadings stable (r = .996) | S18.5, Tables S18.11–S18.14 |
| Interpretability | Percentiles + SEM/MDC_95 | Score distributions; measurement error | Reference percentiles and MDC_95 provided | S18.2, Tables S18.3–S18.5 |
| Scoring rule | Item 31 sensitivity (IIS composite) | IIS scored with 7 items (asd25–asd31) vs. 6 items (asd25–asd30) | Every block-level index deteriorated without item 31 (α −.007; ICC −.021; $MDC_{95}$ +.107) and the block mean rose by 0.088; seven-item composite retained | S18.6, Tables S18.15–S18.16 |

*Note.* This table consolidates robustness evidence across methods; it points to existing tables and figures rather than reproducing them. The table is organized to enable readers to locate the primary evidence for each robustness check directly.

**S20. PSSDS-28 User Guide and Scoring**

**(PSSDS-28 Final Scoring/Reporting Form; PSSDS-31 Administered Archive Form)**

**A1. Purpose**

The Perceived Social Support in Disasters Scale (PSSDS) was developed to measure source-based and function-based dimensions of perceived social support (family/relative, social environment, institutional, emotional, and instrumental–informational support) in adults who have experienced an earthquake. The instrument is designed for use in the context of rapid-onset natural disasters such as earthquakes, and is suitable for research, needs assessment, and evaluation/monitoring of post-disaster psychosocial support programs.

**Validation context and scope of generalizability.** The psychometric validation of the PSSDS-28 was conducted in a sample of adults affected by the February 6, 2023 Kahramanmaraş-centered earthquakes. Accordingly, the psychometric evidence presented (factor structure, reliability, validity, measurement invariance) is directly and empirically supported only for the earthquake context. Because the item content (family/relative, social environment, institutional, emotional, and instrumental–informational support sources) does not employ earthquake-specific language, the PSSDS-28 is considered conceptually adaptable to other rapid-onset natural disaster types such as floods, hurricanes, tornadoes, avalanches, and landslides. However, this generalization is entirely a theoretical projection and has not been empirically tested. Prior to use in a disaster type other than an earthquake (or in slow-onset disaster/crisis types, e.g., drought, pandemic, economic crisis), it is strongly recommended that the factor structure, reliability, and validity of the scale be re-examined in an independent sample specific to that disaster type (psychometric re-validation). Until such validation is completed, scores obtained with the PSSDS-28 in non-earthquake disaster contexts should be interpreted only for theoretical/exploratory purposes, and with caution.

**Not a diagnostic instrument.** The PSSDS does not diagnose any mental disorder. It provides dimensional severity/intensity-level ratings of perceived social support.

**Validated structure used in this study.** The recommended form (PSSDS-28) is a five-factor (F1–F5) instrument supported by calibration-subsample exploratory analyses (EFA/EGA), validation-subsample confirmatory analyses (CFA/ESEM), and complementary IRT/Mokken and network psychometric evidence (S10, S11, S15 and S16).

**A2. Target Population and Eligibility**

**Recommended eligible population**

- Adults (aged 18 and older).
- Individuals directly affected by an earthquake disaster (e.g., loss of shelter, bereavement, injury, property loss).

**Use for non-earthquake rapid-onset disaster types** (e.g., flood, hurricane): As noted in A1, the instrument has not yet been independently validated for these contexts; psychometric re-examination in a sample specific to the relevant disaster type is recommended before use.

**Validation context (for comparability)**

The primary validation was carried out in Türkiye among adults aged 18 and older who, at the time of the February 6, 2023 Kahramanmaraş-centered earthquakes, were residents of the eleven provinces declared affected (Kahramanmaraş, Hatay, Adıyaman, Gaziantep, Malatya, Şanlıurfa, Diyarbakır, Adana, Osmaniye, Kilis, and Elazığ), who were Turkish citizens, and who had Turkish literacy. Fieldwork was conducted in eight of these provinces — Adana, Adıyaman, Diyarbakır, Gaziantep, Hatay, Kahramanmaraş, Malatya and Osmaniye — and the realized sample is described in S6. Administration was in Turkish, using the dual-anchor response frame described in A3 (support experienced since the dated event, with particular emphasis on the past month). These sample characteristics define the specific context to which the presented psychometric evidence is directly generalizable; use in a different earthquake event (different country/region, different time-since-event window, different cultural context) may require re-testing of measurement invariance.

**Recommended screening/eligibility questions (not part of the scale)**

1. Were you living in the disaster zone (the 11 provinces) at the time of the earthquake? (Yes/No)
2. Were you directly affected by the earthquake (e.g., loss of shelter, bereavement, injury, property loss)? (Yes/No)
3. Are you 18 years of age or older? (Yes/No)
4. Are you a Turkish citizen? (Yes/No)
5. Do you have Turkish literacy? (Yes/No)

If the answer to any of the required screening questions is "No," the PSSDS should not be administered (or a skip logic consistent with the study objectives should be applied).

**Cut-off scores**

Because the validation did not include a clinical gold standard, no clinical cut-off scores are recommended. Distributional references/percentiles derived from the fully QC-approved main study sample (N = 607) should be used (see Section A8).

**A3. Administration Format**

- Self-report (online or paper-and-pencil) or interviewer-assisted (neutral read-aloud is permitted).
- **Time frame:** a dual anchor — support experienced since the disaster event, with particular emphasis on the past month. In the validation study the administered wording was "since the 6 February 2023 earthquakes, with particular emphasis on the past month." Users administering the scale after a different event should substitute that event and its date while preserving both components of the anchor, since the psychometric evidence reported here was obtained under the dual-window instruction.
- **Response format (1–5):** 1 = Strongly disagree; 2 = Disagree; 3 = Neither agree nor disagree; 4 = Agree; 5 = Strongly agree.

- **Administration time:** The anticipated completion time for the questionnaire form is approximately 10–15 minutes. In the main study the observed mean completion time was 11.97 minutes (SD = 3.84); this value is reported in S5.2, where it also defines the speeding-exclusion cut-off of 3.99 minutes (mean/3).

### A4. Instructions (Recommended Wording)

**Instructions for participants**

*"The following statements are intended to help you evaluate the social support you have received since the earthquake. Please read each statement and, based on your experience since [event name and date] and with particular emphasis on the past month, select one of the following options that best reflects your own experience. If you do not wish to answer a question, you may leave it blank."*

*Note.* The statement above is a generalized version of the instructions administered in the validation study, which read "since the 6 February 2023 earthquakes, with particular emphasis on the past month"; users referring to a specific disaster event should substitute the name and date of that event while retaining both components of the anchor.

**Distress note (recommended)**

Because disaster- and loss-related items may cause distress, participants should be given permission to take a break, and local support resources should be provided. The form used in this study informed participants that *"If you feel very distressed, you may call the ALO 183 Social Support Hotline, which operates 24/7"* and explicitly stated that participation was entirely voluntary and that participants could take a break or discontinue the study at any time.

### A5. Item Sets and Wordings (PSSDS-28 Final vs. PSSDS-31 Archive)

#### A5.1. PSSDS-28 (Final psychometric form used in CFA/MI/IRT/network analyses)

The PSSDS-28 is the recommended form for scoring, reporting, and psychometric analyses within the scope of this project. In this study, the retained PSSDS-28 items were administered not as a separate, independent 28-item questionnaire, but as part of the 31-item PSSDS-31 form. The following 28-item presentation is provided as the final retained scoring/reporting form for future use and for traceability to the validated item set.

- **Retained items (PSSDS-28):** asd1–asd3, asd7–asd31
- **Items removed prior to CFA during the item reduction step in the calibration phase:** asd4, asd5, asd6 (see S10.9, Table S10.19, and S11.2, Table S11.2)

**Fixed blocks (reporting convention)**

- F1 — Family–Relative Support: asd1, asd2, asd3
- F2 — Social Environment Support: asd7, asd8, asd9, asd10, asd11, asd12
- F3 — Institutional Support: asd13, asd14, asd15, asd16, asd17, asd18

- F4 — Emotional Support: asd19, asd20, asd21, asd22, asd23, asd24
- F5 — Instrumental–Informational Support: asd25, asd26, asd27, asd28, asd29, asd30, asd31

**Table S20.1.** PSSDS-28 Items (English; administration language; dataset codes)

| Block | Item Code | Item (English) |
|---|---|---|
| F1 | asd1 | My family was there for me when I needed them. |
| F1 | asd2 | I relied on my family during difficult times. |
| F1 | asd3 | My family supported me throughout my long-term recovery. |
| F2 | asd7 | My friends were there for me. |
| F2 | asd8 | My neighbors looked out for me. |
| F2 | asd9 | I found people around me from whom I could seek support. |
| F2 | asd10 | Our community acted in solidarity. |
| F2 | asd11 | There was mutual aid in our neighborhood/village. |
| F2 | asd12 | I connected with others who had experienced the disaster. |
| F3 | asd13 | Government institutions provided support. |
| F3 | asd14 | NGOs and aid organizations provided help. |
| F3 | asd15 | I was able to reach institutions when needed. |
| F3 | asd16 | The services institutions provided met my needs. |
| F3 | asd17 | I was able to access health services. |
| F3 | asd18 | Institutions acted in a trustworthy manner. |
| F4 | asd19 | I was able to share my feelings. |
| F4 | asd20 | There were people who listened to me. |
| F4 | asd21 | I found people who understood what I was going through. |
| F4 | asd22 | I encountered people who showed empathy. |
| F4 | asd23 | I felt that there were people beside me. |
| F4 | asd24 | I was helped to achieve emotional relief. |
| F5 | asd25 | My shelter needs were met. |
| F5 | asd26 | My basic needs such as food and clothing were met. |
| F5 | asd27 | My transportation needs were met. |
| F5 | asd28 | I was able to obtain financial support. |
| F5 | asd29 | I obtained information about where to apply. |
| F5 | asd30 | I was guided about the resources available. |
| F5 | asd31 | I was able to obtain legal counseling. |

### A5.2. PSSDS-31 (Administered form; archive reference)

The PSSDS-31 is the administered version used during data collection (the final item pool following the Delphi panel and cognitive interviews). It is included for archival traceability, as item reduction from PSSDS-31 to PSSDS-28 took place after the calibration-phase psychometric evaluation and before the validation-phase confirmatory analyses.

**Table S20.2.** PSSDS-31 Items (English; and PSSDS-28 Status)

| No. | Dataset Item | Item (English) | PSSDS-28 Status |
|---|---|---|---|
| 1 | asd1 | My family was there for me when I needed them. | Retained |
| 2 | asd2 | I relied on my family during difficult times. | Retained |
| 3 | asd3 | My family supported me throughout my long-term recovery. | Retained |
| 4 | asd4 | I shared my experiences with my relatives. | Removed |
| 5 | asd5 | My relatives helped me. | Removed |
| 6 | asd6 | I stayed in contact with my relatives. | Removed |
| 7 | asd7 | My friends were there for me. | Retained |
| 8 | asd8 | My neighbors looked out for me. | Retained |
| 9 | asd9 | I found people around me from whom I could seek support. | Retained |
| 10 | asd10 | Our community acted in solidarity. | Retained |
| 11 | asd11 | There was mutual aid in our neighborhood/village. | Retained |
| 12 | asd12 | I connected with others who had experienced the disaster. | Retained |
| 13 | asd13 | Government institutions provided support. | Retained |
| 14 | asd14 | NGOs and aid organizations provided help. | Retained |
| 15 | asd15 | I was able to reach institutions when needed. | Retained |
| 16 | asd16 | The services institutions provided met my needs. | Retained |
| 17 | asd17 | I was able to access health services. | Retained |
| 18 | asd18 | Institutions acted in a trustworthy manner. | Retained |
| 19 | asd19 | I was able to share my feelings. | Retained |
| 20 | asd20 | There were people who listened to me. | Retained |
| 21 | asd21 | I found people who understood what I was going through. | Retained |
| 22 | asd22 | I encountered people who showed empathy. | Retained |
| 23 | asd23 | I felt that there were people beside me. | Retained |
| 24 | asd24 | I was helped to achieve emotional relief. | Retained |
| 25 | asd25 | My shelter needs were met. | Retained |
| 26 | asd26 | My basic needs such as food and clothing were met. | Retained |
| 27 | asd27 | My transportation needs were met. | Retained |
| 28 | asd28 | I was able to obtain financial support. | Retained |

| | | | |
|---|---|---|---|
| 29 | asd29 | I obtained information about where to apply. | Retained |
| 30 | asd30 | I was guided about the resources available. | Retained |
| 31 | asd31 | I was able to obtain legal counseling. | Retained |

*Note*. The reduction from PSSDS-31 to PSSDS-28 was determined exclusively on the basis of calibration-subsample evidence (EFA theoretical–empirical misalignment, low EGA bootstrap stability) and pilot monitoring findings (S10.9). The questionnaire also included three instructed-response attention-check items, each specifying a different correct response (e.g., "Please mark this item as 'Strongly disagree'"); these items are not part of the PSSDS-31/28 item set and were used for data quality screening, with exclusion applied when two or more were failed (S5.1–S5.2). FRS = Family–Relative Support; SES = Social Environment Support; IS = Institutional Support; ES = Emotional Support; IIS = Instrumental–Informational Support.

**A6. Scoring**

**A6.1. Response coding**

- Each item is scored from 1 to 5 (Strongly disagree – Strongly agree).
- Higher scores reflect higher levels of perceived social support.

**A6.2. Recommended scoring (PSSDS-28; primary)**

**Block mean scores (1–5; recommended):**

- F1 mean = mean(asd1, asd2, asd3)
- F2 mean = mean(asd7, asd8, asd9, asd10, asd11, asd12)
- F3 mean = mean(asd13, asd14, asd15, asd16, asd17, asd18)
- F4 mean = mean(asd19, asd20, asd21, asd22, asd23, asd24)
- F5 mean = mean(asd25, asd26, asd27, asd28, asd29, asd30, asd31)

**Block sum scores (optional):**

- F1 sum range: 3–15
- F2 sum range: 6–30
- F3 sum range: 6–30
- F4 sum range: 6–30
- F5 sum range: 7–35

*Reporting item 31.* Item 31 (asd31) contributes to the F5 composite in all applications, and every psychometric property reported for F5 in this work refers to the seven-item block. In applied needs-assessment settings, item 31 should additionally be reported on its own, alongside the F5 composite, as a descriptive indicator of unmet legal-aid need. This is an addition to the composite score and not a substitute for it: the composite is the scored unit, and the standalone value is supplementary information. The rationale is that item 31 has the highest floor rate in the instrument (38.35%), so a composite that averages it with six higher-scoring items necessarily attenuates the signal it carries. Sensitivity evidence for this rule is reported in S18.6; removing item 31 from the composite lowers reliability and temporal stability, widens the minimal detectable change, and raises the F5 score.

### A6.3. Total score (optional; descriptive use only)

Because the PSSDS-28 is supported as a five-block instrument, primary interpretation should be based on the block profile. If a global index is required, compute and report TOTAL28 for descriptive purposes only and interpret with caution (multidimensional structure); this does not imply strict unidimensionality:

- TOTAL28 mean (1–5) = mean(all 28 retained items)
- TOTAL28 sum (28–140) = sum(all 28 retained items)

### A6.4. Reverse-scored items

- The PSSDS-28 contains no reverse-scored items.

## A7. Missing Responses (Recommended Rule for Applied Use)

Apply the minimum-item rule set out below: all items must be answered in the three-item block, at least two thirds of the items in the six- and seven-item blocks, and at least 22 of 28 items (78.6%) for the total score. Unless a separate imputation protocol is planned, no imputation should be performed by default in field applications.

**PSSDS-28:**

- F1 (3 items): compute if all 3 items are answered (strict rule; due to small block size)
- F2 (6 items): compute if ≥ 4 items are answered
- F3 (6 items): compute if ≥ 4 items are answered
- F4 (6 items): compute if ≥ 4 items are answered
- F5 (7 items): compute if ≥ 5 items are answered
- TOTAL28 (28 items): compute if ≥ 22 items are answered

If the rule is met, compute the mean using available items.

### A8. Interpretation Guide (Distributional References and Change Interpretation)

#### A8.1. Distributional references (main study sample; mean metric 1–5)

The following are descriptive references (not cut-off scores) derived from the QC-approved sample (N = 607; S18.2, Table S18.3):

- **TOTAL28 mean:** M = 3.518; SD = 0.760; P10 = 2.464; P50 = 3.607; P90 = 4.500
- **F1 mean:** M = 4.174; SD = 1.097; P10 = 2.667; P90 = 5.000
- **F2 mean:** M = 3.769; SD = 0.902; P10 = 2.600; P90 = 5.000
- **F3 mean:** M = 3.294; SD = 1.165; P10 = 1.500; P90 = 4.833
- **F4 mean:** M = 3.777; SD = 1.031; P10 = 2.333; P90 = 5.000
- **F5 mean:** M = 2.989; SD = 1.056; P10 = 1.571; P90 = 4.286

#### A8.2. Change interpretation (SEM/$MDC_{95}$; mean metric)

Two sets of SEM and $MDC_{95}$ values are available, and they answer different questions. The alpha-based values (S18.2, Table S18.5) describe the precision of a single administration. The retest-based values (S13.4, Table S13.4), derived from the test–retest ICCs, are the appropriate reference for judging whether an observed within-person change exceeds measurement error, because the alpha-based figures treat all non-error variance as stable over time. For repeated-measure monitoring use the retest-based value; the alpha-based value is given after it for comparison:

- F1 (FRS) $MDC_{95}$ = 2.015 retest-based; 0.689 alpha-based
- F2 (SES) $MDC_{95}$ = 1.259 retest-based; 0.961 alpha-based
- F3 (IS) $MDC_{95}$ = 0.880 retest-based; 0.808 alpha-based
- F4 (ES) $MDC_{95}$ = 0.668 retest-based; 0.706 alpha-based
- F5 (IIS) $MDC_{95}$ = 0.696 retest-based; 0.928 alpha-based
- TOTAL28 $MDC_{95} \approx 0.516$ (alpha-based; reported as a practical aid; does not imply strict unidimensionality). No retest-based total-score value is reported, because test–retest reliability was estimated at block level (Table S13.4). The retest-based value exceeds the alpha-based value in F1, F2 and F3, where temporal stability is lower, and falls below it in F4 and F5, where stability is high; in every block it remains the reference for change.

### A9. Group Comparisons: Measurement Invariance and DIF Notes (Recommended Reporting)

- Ordinal measurement invariance (scalar/strong) was supported for six of the nine grouping variables tested in this study (age group, gender, marital status, education level, employment status, and psychological support receipt) under prespecified practical Δ-fit criteria (see S12). Accordingly, latent

mean comparisons are supported for these six variables within the current validation context. For three variables (monthly income, type of loss experienced, and housing status), the configural model could not be estimated due to empty response categories in small subgroups; this is noted as a statistical constraint (S12.3).

- The DIF scan showed no robust, practically significant item bias for any of the six tested grouping variables (ΔMcFadden $R^2 < .010$ across all 168 item-by-group combinations; see S18.1).
- As a general reporting preference, latent-variable-based group comparisons should be preferred over item-level comparisons wherever possible.

**A10. Recommended Reporting Checklist**

At minimum, report the following:

- Disaster type and key event characteristics (e.g., earthquake; date; geographic region), and whether this disaster type corresponds to the context in which the PSSDS-28 was validated (earthquake)
- Sampling frame and eligibility criteria (disaster zone residency requirement; direct exposure requirement; retrospective response window)
- Administration language and mode (self-report vs. interviewer-assisted)
- Block means/standard deviations (and N), and total score descriptives if used
- Reliability per block (ordinal α/ω and, where relevant, model-based CR/AVE; S13.2–S13.3)
- Missing-response scoring rule applied (minimum-item rule per block; mean of available items)
- MI/DIF context notes when comparing groups (S12 and S18.1)
- If used in a non-earthquake disaster type, it should be explicitly stated that this constitutes an exploratory/preliminary application and that independent psychometric validation is required

**A11. Computation Example (R; Mean Scores with the Minimum-Item Rule)**

```
R
mean_na <- function(x) mean(x, na.rm = TRUE)
min_answered <- function(x, min_n) sum(!is.na(x)) >= min_n

df$F1_mean <- apply(df[, c("asd1","asd2","asd3")], 1, function(x)
  if (min_answered(x, 3)) mean_na(x) else NA_real_
)
df$F2_mean <- apply(df[, c("asd7","asd8","asd9","asd10","asd11","asd12")], 1, function(x)
  if (min_answered(x, 4)) mean_na(x) else NA_real_
)
```

```
df$F3_mean <- apply(df[, c("asd13","asd14","asd15","asd16","asd17","asd18")], 1, function(x)
  if (min_answered(x, 4)) mean_na(x) else NA_real_
)
df$F4_mean <- apply(df[, c("asd19","asd20","asd21","asd22","asd23","asd24")], 1, function(x)
  if (min_answered(x, 4)) mean_na(x) else NA_real_
)
df$F5_mean <- apply(df[, c("asd25","asd26","asd27","asd28","asd29","asd30","asd31")], 1, function(x)
  if (min_answered(x, 5)) mean_na(x) else NA_real_
)

items28 <- c("asd1","asd2","asd3","asd7","asd8","asd9","asd10","asd11","asd12",
             "asd13","asd14","asd15","asd16","asd17","asd18",
             "asd19","asd20","asd21","asd22","asd23","asd24",
             "asd25","asd26","asd27","asd28","asd29","asd30","asd31")
df$TOTAL28_mean <- apply(df[, items28], 1, function(x)
  if (min_answered(x, 22)) mean_na(x) else NA_real_
)
```

**A12. Citation and Permitted Use**

If you use the PSSDS-28, please cite the primary source that introduced and validated the scale (the article reporting this development and validation study).

**Recommended permission statement:**

- Non-commercial use for academic research and program evaluation is permitted, provided that users (i) cite the original source and (ii) administer the items as written when reporting results as "PSSDS-28."
- For translations/adaptations, follow cross-cultural adaptation guidelines and report psychometric evidence in the new context.
- The same principle applies to adaptation for non-earthquake disaster types (flood, hurricane, tornado, avalanche, landslide, etc.): although the item wording does not contain disaster-type-specific language and may appear directly applicable, the factor structure, reliability, and validity should be re-tested and reported in an independent sample specific to that disaster type before use (see A1).

## S21. MAPPING TO REPORTING AND MEASUREMENT STANDARDS (PSSDS-28)

This section provides alignment ("mapping") tables summarizing how the PSSDS-28 scale-development and validation study corresponds to major reporting and measurement frameworks. The purpose is transparency and auditability, not formal "certification." Evidence locations are referenced to the online Supplementary Material (SM; Sections S1–S21), to the main manuscript (Method, Results and Discussion; Tables 1–9, Figures 1–2), and to the OSF project archive (anonymized data, analysis scripts, codebooks, search documentation, and study instruments; link provided in the Data Availability Statement).

**Frameworks mapped**

1. AERA–APA–NCME Standards for Educational and Psychological Testing (2014) (validity evidence, intended use, fairness)
2. APA JARS–Quant (Appelbaum et al., 2018) (quantitative reporting)
3. TOP Guidelines (Nosek et al., 2015) (openness/transparency)
4. STROBE (cross-sectional reporting)
5. COSMIN (measurement properties of self-report instruments; Mokkink et al., 2010)

**Core-criteria approach**

Because the AERA Standards, JARS–Quant, TOP, and COSMIN frameworks are broad, we defined core criteria most relevant to this study's workflow: theory/construct specification (S1) → systematic review, initial item pool, and advisor-panel restructuring (S2) → Delphi content validity and cognitive interviews (S3) → pilot psychometrics and target-N rationale (S4) → main-study data-quality control (S5) → sample characteristics (S6) → missing-data analyses (S7) → split-sample cross-validation architecture (S8) → item-level diagnostics (S9) → exploratory structure (EFA/EGA; S10) → confirmatory structure (CFA/ESEM; competing models; bootstrap; S11) → measurement invariance (S12) → reliability (S13) → validity evidence (S14) → IRT and Mokken scaling (S15) → network psychometrics (S16) → CMV diagnostics (S17) → additional analyses (DIF; score distributions/percentiles; estimator sensitivity; construct replicability; local dependence; S18) → integrated robustness synthesis (S19). For STROBE, all 22 checklist items were considered.

**Scoring (descriptive)**

Each criterion was coded as: Yes (1), Partial (0.5), No (0). Compliance % = (∑ points / number of criteria) × 100.

**Table S21.1.** AERA–APA–NCME Standards (2014): Core mapping and compliance (validity evidence + intended use + fairness; k = 14)

| No. | Core criterion | Why it matters | Evidence in this study (SM / manuscript) | Status | Score |
|---|---|---|---|---|---|
| A1 | Intended use and purpose explicit | Frames validity argument | S1.1–S1.2 (conceptual rationale; intended use) + manuscript Introduction (aims) | Yes | 1 |
| A2 | Construct definition + theoretical boundaries | Clarifies what is measured | S1.2 (construct definition across interpersonal, communal, institutional and functional channels, with the narrower operational boundary stated in the main text: retrospective appraisal at the perceived–received interface, excluding prospective availability) | Yes | 1 |
| A3 | Blueprint / domain representation | Ensures content coverage | S1.3, Table S1.3 (initial seven-dimension model) + S2.2 (evidence density by dimension) + S2.3 (43-item pool; advisor-panel restructuring to five-factor hybrid model) | Yes | 1 |
| A4 | Content validity evidence (experts) | Item relevance/coverage | S3.2–S3.4 (two-round Delphi, n = 8; I-CVI ≥ .78 rule; S-CVI/Average .946–1.000) | Yes | 1 |

| | | | | | |
|---|---|---|---|---|---|
| A5 | Response-process evidence | Interpretability for respondents | S3.5 (cognitive interviews, n = 12; think-aloud, paraphrasing, probing) | Yes | 1 |
| A6 | Internal structure evidence | Dimensionality/structure support | S10 (polychoric EFA + EGA with bootstrap stability) + S11 (CFA/ESEM in independent validation subsample) | Yes | 1 |
| A7 | Alternative models / robustness | Strengthens structural argument | S11.1–S11.4 (one-factor vs. correlated five-factor vs. second-order vs. bifactor) + S11.7 (ESEM) + S11.8 (bootstrap CFA) + S19 (triangulation map) | Yes | 1 |
| A8 | Relations to other variables | External validity evidence | S14.2–S14.3 (fully latent relations with F-SozU K-3 and UCLA-6; FDR-corrected) | Yes | 1 |
| A9 | Reliability evidence | Precision/consistency | S13.2 (ordinal α/ω with bootstrap CIs) + S13.3 (CR/AVE) + S13.4 (test–retest ICC, n = 89) | Yes | 1 |
| A10 | Measurement error / interpretability | Supports change interpretation | S13.4 (Bland–Altman) + S18.2 (percentiles; SEM/MDC95) + manuscript Score Distributions | Yes | 1 |
| A11 | Invariance / fairness across groups | Enables group comparisons | S12 (ordinal MG-CFA; configural→metric→scalar supported for 6 of 9 groupings; income, loss type, and housing not estimable due to sparse cells) | Partial | 0.5 |
| A12 | DIF (item-level bias) | Item functioning across groups | S18.1 (ordinal logistic DIF; Holm + ΔMcFadden $R^2 \geq .010$; no meaningful DIF across 168 item × group combinations; limited to the six invariance-supported variables) | Partial | 0.5 |
| A13 | Scoring and reporting rules | Standardized use | Manuscript Measures (item set, response format, keying) + Score Distributions (block means; SEM/MDC95) + Table 3 (items) + S20 (stand-alone user guide: scoring rules, block composition, keying, missing-item rule, percentile references) | Yes | 1 |
| A14 | Consequences of testing/use | Intended/unintended outcomes | Manuscript Practical Implications (non-diagnostic status; no cut-off scores; triage use flagged as hypothesis only) | Yes | 1 |

**Total: 13.0 / 14 — Compliance: 92.9%**

**Table S21.2.** APA JARS–Quant (Appelbaum et al., 2018): Core mapping and compliance (reporting completeness; k = 20)

| No. | Core criterion | Evidence in this study (SM / manuscript) | Status | Score |
|---|---|---|---|---|
| J1 | Clear aims and design | Manuscript Overview and Design + S1–S4 (development stages) | Yes | 1 |
| J2 | Participant characteristics | S6 (main-sample demographics) + manuscript Participants | Yes | 1 |
| J3 | Inclusion/exclusion criteria | Manuscript Participants (eligibility) + S5.2 (QC exclusion protocol) | Yes | 1 |
| J4 | Participant flow and final N | S5.2 (65 of 672 excluded, 9.67%; N = 607) | Yes | 1 |
| J5 | Measures/operationalization | Manuscript Measures + Table 3 (PSSDS-28 items) + S1.2 (construct) | Yes | 1 |
| J6 | Scale development steps | S1–S4 (construct → pool → Delphi → cognitive interviews → pilot → final form) | Yes | 1 |

| | | | | |
|---|---|---|---|---|
| J7 | Data quality procedures | S5.1–S5.2 (range/format checks; three instructed-response attention checks; straightlining; speeding) | Yes | 1 |
| J8 | Missingness rates/patterns | S7.1 (0.48% overall; item-level maximum 1.32%) | Yes | 1 |
| J9 | Missingness mechanism diagnostics | S7.2 (Little's MCAR; diagnostic logistic regression) | Yes | 1 |
| J10 | Missing-data handling rationale | S7 (no-imputation rationale; analysis-specific effective N) | Yes | 1 |
| J11 | Sample size justification | S4.3 (a priori target N = 800); realized N = 607 transparently reported, with the post-hoc power curve in S4.3, Figure S4.2 and the observation-to-parameter caveat in manuscript Limitations | Yes | 1 |
| J12 | Rationale for analytic choices | Manuscript Data Analysis Strategy (ordinal treatment; WLSMV) + S9–S19 | Yes | 1 |
| J13 | Model specification detail | S10 (EFA/EGA specifications) + S11 (CFA/ESEM) + S12.1–S12.2 (MI steps; Δ-fit criteria) | Yes | 1 |
| J14 | Competing models/comparisons | S11.1 (model comparison incl. 31- vs. 28-item forms; four competing CFA models) | Yes | 1 |
| J15 | Robustness/sensitivity analyses | S10 (bootstrap EFA/EGA) + S11.8 (bootstrap CFA) + S15–S17 + S18.3 (WLSMV vs. MLR) + S18.5 (local dependence) + S19 | Yes | 1 |
| J16 | Effect sizes/uncertainty | Bootstrap CIs (S13); FDR-corrected latent correlations (S14); ICC CIs and Bland–Altman (S13.4) | Yes | 1 |
| J17 | Software and versions | Manuscript Data Analysis Strategy (R 4.5.2; lavaan, psych, EGAnet, mirt, mokken, bootnet, qgraph, networktools, mgm); fixed seed reported | Yes | 1 |
| J18 | Setting/context detail | Manuscript Procedure (face-to-face, paper-and-pencil; eight provinces; quiet private settings; trained field researchers; completion time) | Yes | 1 |
| J19 | Data/code/material availability | OSF archive (anonymized data, scripts, codebooks, instruments); public access upon article acceptance (Data Availability Statement) | Partial | 0.5 |
| J20 | Transparency of exclusions/analytic Ns | S5.2 (exclusion counts by rule) + S8 (subsample equivalence) + effective N reported per analysis (e.g., network N_cc = 548) | Yes | 1 |

**Total: 19.5 / 20 — Compliance: 97.5%**

Partial note. J19 is coded partial because the OSF archive becomes publicly accessible upon article acceptance; at submission it is available to editors/reviewers via the Data Availability Statement.

**Table S21.3.** TOP Guidelines (Nosek et al., 2015): Core mapping and compliance (openness/transparency; k = 10)

| No. | TOP component | Evidence in this study | Status | Score |
|---|---|---|---|---|
| T1 | Citation standards | Standard scholarly citation throughout; all instruments and analytic methods cited | Yes | 1 |
| T2 | Data availability | Anonymized dataset archived on OSF; public upon acceptance (Data Availability Statement) | Yes | 1 |
| T3 | Code availability | Complete analysis scripts archived on OSF (fixed seed; session information) | Yes | 1 |
| T4 | Materials availability | Study instruments and codebooks on OSF + full item set in manuscript Table 3 | Yes | 1 |

| | | | | |
|---|---|---|---|---|
| T5 | Design/analysis transparency | SM S1–S19 document the full pipeline and prespecified decision rules | Yes | 1 |
| T6 | Transparent exclusion/QC rules | S5.2 (prespecified QC rules; exclusion counts by rule) | Yes | 1 |
| T7 | Transparent missing-data reporting | S7 (rates; MCAR diagnostics; handling rationale) | Yes | 1 |
| T8 | Study preregistration | Not preregistered | No | 0 |
| T9 | Analysis preregistration | Not preregistered; analytic decision rules were prespecified and documented in the SM, but not registered independently | No | 0 |
| T10 | Replication facilitation | Split-sample cross-validation (S8) + MI/DIF evidence (S12, S18.1) + OSF materials + robustness synthesis (S19) | Yes | 1 |

**Total: 8.0 / 10 — Compliance: 80.0%**

**Table S21.4.** STROBE (cross-sectional) mapping and compliance (k = 22; full checklist)

| **No.** | **STROBE item (short)** | **Evidence in this study (SM / manuscript)** | **Status** | **Score** |
|---|---|---|---|---|
| ST1 | Title/abstract indicate design | Title/abstract report development-and-validation design; the term “cross-sectional” is not used explicitly | Partial | 0.5 |
| ST2 | Background/rationale | Manuscript Introduction (theoretical frameworks; instrument-coverage analysis; Table 1) + S1 | Yes | 1 |
| ST3 | Objectives | Manuscript Introduction (aims paragraph) | Yes | 1 |
| ST4 | Study design | Manuscript Overview and Design + S8 (calibration/validation architecture) | Yes | 1 |
| ST5 | Setting | Manuscript Procedure (eight provinces; face-to-face; May–June 2026) + S4.4, Table S4.5 (phase-by-phase fieldwork dates) | Yes | 1 |
| ST6 | Participants | Manuscript Participants (eligibility) + S5.2 (QC flow) | Yes | 1 |
| ST7 | Variables | Manuscript Measures + S1.2 (construct) + demographic/disaster-context form | Yes | 1 |
| ST8 | Data sources/measurement | Manuscript Measures + Table 3 (items) + S20, Table A2 (administered 31-item form) | Yes | 1 |
| ST9 | Bias | S5 (QC), S17 (CMV diagnostics), S12/S18.1 (MI/DIF) | Yes | 1 |
| ST10 | Study size | S4.3 (target N) + manuscript Participants (achieved N = 607) | Yes | 1 |
| ST11 | Quantitative variables | Manuscript Measures (Likert scaling) + Score Distributions (SEM/MDC95) | Yes | 1 |
| ST12 | Statistical methods | Manuscript Data Analysis Strategy + S9–S19 | Yes | 1 |
| ST13 | Participants (flow) | S5.2 (exclusions by rule; retained N) | Yes | 1 |
| ST14 | Descriptive data | S6 + manuscript Participants (demographics; loss profile) | Yes | 1 |
| ST15 | Outcome data | Manuscript Results + S13–S18 | Yes | 1 |
| ST16 | Main results | Manuscript Results (Tables 2–7; Figures 1–2) | Yes | 1 |
| ST17 | Other analyses | S15–S19 (IRT/Mokken; network; CMV; DIF; sensitivity) | Yes | 1 |
| ST18 | Key results summary | Manuscript Discussion/Conclusion | Yes | 1 |

| | | | | |
|---|---|---|---|---|
| ST19 | Limitations | Manuscript Limitations and Future Directions + caveats in S11–S18 | Yes | 1 |
| ST20 | Interpretation | Manuscript Discussion (theoretical interpretation; methodological tensions) | Yes | 1 |
| ST21 | Generalisability | Manuscript Limitations (single event type; single culture; explicit replication agenda) | Yes | 1 |
| ST22 | Funding | Manuscript Funding statement | Yes | 1 |

**Total: 21.5 / 22 — Compliance: 97.7%**

**Table S21.5.** COSMIN (measurement properties): Core mapping and compliance (self-report instruments; k = 12)

| No. | COSMIN core domain | Evidence in this study (SM / manuscript) | Status | Score |
|---|---|---|---|---|
| C1 | Construct + target population defined | S1.2 (construct definition) + manuscript Participants (disaster-affected adults) | Yes | 1 |
| C2 | Content validity (experts) | S3.2–S3.4 (two-round Delphi; I-CVI/S-CVI with prespecified thresholds; universal agreement) | Yes | 1 |
| C3 | Content validity (response process) | S3.5 (cognitive interviews, n = 12) | Yes | 1 |
| C4 | Structural validity | S10 (EFA/EGA) + S11 (CFA/ESEM) + S16.4 (EGA/bootEGA 100% structural replication) | Yes | 1 |
| C5 | Internal consistency | S13.2 (ordinal α/ω with bootstrap CIs) + S13.3 (CR/AVE) | Yes | 1 |
| C6 | Reliability (test–retest) | S13.4 (ICC[2,1], n = 89, ≈21 days; Bland–Altman) | Yes | 1 |
| C7 | Measurement error | S13/S18.2 (SEM; MDC95 per block) | Yes | 1 |
| C8 | Cross-cultural validity / MI | Demographic and disaster-context MI established for six groupings (S12); cross-cultural validity not evaluated (single-language sample) | Partial | 0.5 |
| C9 | Criterion validity | No clinical gold standard administered | No | 0 |
| C10 | Hypothesis testing / relations to other variables | S14.2–S14.3 (directional hypotheses for F-SozU K-3 and UCLA-6 confirmed; FDR-corrected) | Yes | 1 |
| C11 | Known-groups validity | S14.4 (latent mean comparisons under scalar invariance; psychological-support groups) | Yes | 1 |
| C12 | Responsiveness | Not evaluated (no longitudinal/intervention design) | No | 0 |

**Total: 9.5 / 12 — Compliance: 79.2%**

**Summary**

Across frameworks, compliance was 92.9% (AERA–APA–NCME), 97.5% (JARS–Quant), 80.0% (TOP), 97.7% (STROBE), and 79.2% (COSMIN). Unmet or partially met elements—preregistration (T8–T9), criterion validity against a clinical gold standard (C9), responsiveness (C12), cross-cultural validity (C8), and invariance across income, loss type, and housing (A11)—reflect design-stage boundaries of a first validation study rather than reporting omissions; each is identified in the manuscript as a priority for the future research agenda.

## References


American Educational Research Association, American Psychological Association, & National Council on Measurement in Education. (2014). *Standards for educational and psychological testing*. American Educational Research Association.

Appelbaum, M., Cooper, H., Kline, R. B., Mayo-Wilson, E., Nezu, A. M., & Rao, S. M. (2018). Journal article reporting standards for quantitative research in psychology: The APA Publications and Communications Board task force report. *American Psychologist, 73*(1), 3–25. https://doi.org/10.1037/amp0000191

Boateng, G. O., Neilands, T. B., Frongillo, E. A., Melgar-Quiñonez, H. R., & Young, S. L. (2018). Best practices for developing and validating scales for health, social, and behavioral research: A primer. *Frontiers in Public Health, 6*, 149. https://doi.org/10.3389/fpubh.2018.00149

Brown, T. A. (2015). *Confirmatory factor analysis for applied research* (2nd ed.). Guilford Press.

Cohen, S., & Wills, T. A. (1985). Stress, social support, and the buffering hypothesis. *Psychological Bulletin, 98*(2), 310–357. https://doi.org/10.1037/0033-2909.98.2.310

Crane, P. K., Gibbons, L. E., Jolley, L., & van Belle, G. (2006). Differential item functioning analysis with ordinal logistic regression techniques. *Medical Care, 44*(11), 115–123. https://doi.org/10.1097/01.mlr.0000245183.28384.ed

Cutrona, C. E., & Russell, D. W. (1990). Type of social support and specific stress: Toward a theory of optimal matching. In B. R. Sarason, I. G. Sarason, & G. R. Pierce (Eds.), *Social support: An interactional view* (pp. 319–366). Wiley.

DeVellis, R. F., & Thorpe, C. T. (2022). *Scale development: Theory and applications* (5th ed.). Sage.

Haber, M. G., Cohen, J. L., Lucas, T., & Baltes, B. B. (2007). The relationship between self-reported received and perceived social support: A meta-analytic review. *American Journal of Community Psychology, 39*(1–2), 133–144. https://doi.org/10.1007/s10464-007-9100-9

Kaniasty, K., & Norris, F. H. (2008). Longitudinal linkages between perceived social support and posttraumatic stress symptoms. *Journal of Traumatic Stress, 21*(3), 274–281. https://doi.org/10.1002/jts.20334

Lakey, B., & Orehek, E. (2011). Relational regulation theory: A new approach to explain the link between perceived social support and mental health. *Psychological Review, 118*(3), 482–495. https://doi.org/10.1037/a0023477

Laksmita, O. D., Chung, M.-H., Liao, Y.-M., & Chang, P.-C. (2020). Multidimensional scale of perceived social support in Indonesian adolescent disaster survivors: A psychometric evaluation. *PLOS ONE, 15*(3), e0229958. https://doi.org/10.1371/journal.pone.0229958

Lam, P. H. (2024). An extension to the stress-buffering model: Timing of support across the lifecourse. *Brain, Behavior, and Immunity – Health, 42*, 100876. https://doi.org/10.1016/j.bbih.2024.100876

Leykin, D., Lahad, M., Cohen, O., Goldberg, A., & Aharonson-Daniel, L. (2013). Conjoint Community Resiliency Assessment Measure-28/10 items (CCRAM28 and CCRAM10): A self-report tool for assessing community resilience. *American Journal of Community Psychology, 52*(3–4), 313–323. https://doi.org/10.1007/s10464-013-9596-0

Lynn, M. R. (1986). Determination and quantification of content validity. *Nursing Research, 35*(6), 382–385. https://doi.org/10.1097/00006199-198611000-00017

Ma, C., Smith, T. E., & Culhane, D. P. (2025). The stress-buffering effects of received social support on post-traumatic stress disorder among Hurricane Ike survivors. *Traumatology, 31*(2), 276–284. https://doi.org/10.1037/trm0000526

Mokkink, L. B., Terwee, C. B., Patrick, D. L., Alonso, J., Stratford, P. W., Knol, D. L., Bouter, L. M., & de Vet, H. C. W. (2010). The COSMIN study reached international consensus on taxonomy, terminology, and definitions of measurement properties. *Journal of Clinical Epidemiology, 63*(7), 737–745. https://doi.org/10.1016/j.jclinepi.2010.02.006

Norris, F. H., & Kaniasty, K. (1996). Received and perceived social support in times of stress: A test of the social support deterioration deterrence model. *Journal of Personality and Social Psychology, 71*(3), 498–511. https://doi.org/10.1037/0022-3514.71.3.498

Norris, F. H., Friedman, M. J., Watson, P. J., Byrne, C. M., Diaz, E., & Kaniasty, K. (2002). 60,000 disaster victims speak: Part I. An empirical review of the empirical literature, 1981–2001. *Psychiatry, 65*(3), 207–239. https://doi.org/10.1521/psyc.65.3.207.20173

Nosek, B. A., Alter, G., Banks, G. C., Borsboom, D., Bowman, S. D., Breckler, S. J., Buck, S., Chambers, C. D., Chin, G., Christensen, G., Contestabile, M., Dafoe, A., Eich, E., Freese, J., Glennerster, R., Goroff, D., Green, D. P., Hesse, B., Humphreys, M., … Yarkoni, T. (2015). Promoting an open research culture. *Science, 348*(6242), 1422–1425. https://doi.org/10.1126/science.aab2374

Page, M. J., McKenzie, J. E., Bossuyt, P. M., Boutron, I., Hoffmann, T. C., Mulrow, C. D., & Moher, D. (2021). The PRISMA 2020 statement: An updated guideline for reporting systematic reviews. *BMJ, 372*, n71. https://doi.org/10.1136/bmj.n71

Podsakoff, P. M., MacKenzie, S. B., Lee, J.-Y., & Podsakoff, N. P. (2003). Common method biases in behavioral research: A critical review of the literature and recommended remedies. *Journal of Applied Psychology, 88*(5), 879–903. https://doi.org/10.1037/0021-9010.88.5.879

Rhemtulla, M., Brosseau-Liard, P. É., & Savalei, V. (2012). When can categorical variables be treated as continuous? A comparison of robust continuous and categorical SEM estimation methods under suboptimal conditions. *Psychological Methods, 17*(3), 354–373. https://doi.org/10.1037/a0029315

Thoresen, S., Birkeland, M. S., Wentzel-Larsen, T., & Blix, I. (2018). Loss of trust may never heal. Institutional trust in disaster victims in a long-term perspective: Associations with social support and mental health. *Frontiers in Psychology, 9*, 1204. https://doi.org/10.3389/fpsyg.2018.01204

Wheaton, B. (1985). Models for the stress-buffering functions of coping resources. *Journal of Health and Social Behavior, 26*(4), 352–364. https://doi.org/10.2307/2136658

Williams, L. J., Hartman, N., & Cavazotte, F. (2010). Method variance and marker variables: A review and comprehensive CFA marker technique. *Organizational Research Methods, 13*(3), 477–514. https://doi.org/10.1177/1094428110366036

Yang, Y.-S., & Bae, S.-M. (2022). Association between resilience, social support, and institutional trust and post-traumatic stress disorder after natural disasters. *Archives of Psychiatric Nursing, 37*, 39–44. https://doi.org/10.1016/j.apnu.2022.01.001

Yen, W. M. (1993). Scaling performance assessments: Strategies for managing local item dependence. *Journal of Educational Measurement, 30*(3), 187–213. https://doi.org/10.1111/j.1745-3984.1993.tb00423.x

Zimet, G. D., Dahlem, N. W., Zimet, S. G., & Farley, G. K. (1988). The Multidimensional Scale of Perceived Social Support. *Journal of Personality Assessment, 52*(1), 30–41. https://doi.org/10.1207/s15327752jpa5201_2
